# A living library concept to capture the dynamics and reactivity of mixed-metal clusters for catalysis

**DOI:** 10.1038/s41557-024-01726-3

**Published:** 2025-01-23

**Authors:** Raphael Bühler, Max Schütz, Karla F. Andriani, Marcos G. Quiles, João Paulo A. de Mendonça, Vivianne K. Ocampo-Restrepo, Johannes Stephan, Sophia Ling, Samia Kahlal, Jean-Yves Saillard, Christian Gemel, Juarez L. F. Da Silva, Roland A. Fischer

**Affiliations:** 1 TUM School of Natural Sciences, Department of Chemistry, Chair of Inorganic and Metal-Organic Chemistry and Catalysis Research Center, Technical University of Munich, Garching, Germany; 2 São Carlos Institute of Chemistry, University of São Paulo, São Paulo, Brazil; 3 Department of Exact Sciences, State University of Santa Cruz, Ilhéus, Brazil; 4 Department of Science and Technology, Federal University of São Paulo, São José dos Campos, Brazil; 5 Univ Rennes CNRS, ISCR-UMR 6226, Rennes, France

**Keywords:** Synthetic chemistry methodology, Chemical libraries, Catalysis

## Abstract

The exploration of ligated metal clusters’ chemical space is challenging, partly owing to an insufficiently targeted access to reactive clusters. Now, dynamic mixtures of clusters, defined as living libraries, are obtained through organometallic precursor chemistry. The libraries are populated with interrelated clusters, including transient and highly reactive ones, as well as more accessible but less reactive species. Their evolutions upon perturbation with substrate molecules are monitored and chemical information is gained without separation of the clusters. Here we prepared a library of all-hydrocarbon ligated Cu/Zn clusters and developed a bias-free computational framework suited to analyse the full compositional space that yields a reliable structural model for each cluster. This methodology enables efficient searches for structure–reactivity relationships relevant for catalysis with mixed-metal clusters: when treating the library with CO_2_ or 3-hexyne and H_2_, we discovered [Cu_11_Zn_6_](Cp*)_8_(CO_2_)_2_(HCO_2_) bearing a formate species related to CO_2_ reduction and [Cu_9_Zn_7_](Cp*)_6_(Hex)_3_(H)_3_ bearing C_6_ species related to alkyne semi-hydrogenation.

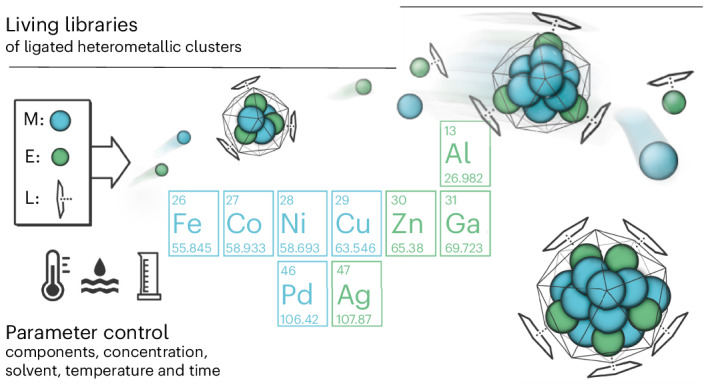

## Abstract (web summary)

Understanding ligated metal clusters’ reactivities is challenging. Now, a methodology has been developed to explore dynamic mixtures of metal clusters. Interrelated cluster populations are constructed through organometallic precursor chemistry. Structural information on mixed-metal systems is obtained with a bias-free computational framework, and reactivities towards CO_2_ and alkynes are investigated in situ.

## Main

Access to sub-nanometre metal clusters is largely based on metal evaporation, ionization and atom-precise size selection by electromagnetic mass filters. This gas-phase synthesis and spectrometry, together with size-conservative deposition to substrates, enable microscopic characterization and reactivity studies of individual, non-ligated clusters^1–5^. Atom-by-atom assembly of clusters in the liquid phase has been demonstrated as well. Dendrimer nano-reactors were designed to coordinatively bind and cage a predefined number of metal ions, which then aggregate to the targeted cluster by a chemical reduction step. Removing the dendrimer yields samples of substrate-supported atom-precise clusters similar to the gas-phase approach^6–8^. Neither concept, selection from polydisperse mixtures nor programmed assembly, can be translated to the synthesis of ligated metal clusters. Here the obstacle of selective synthesis design is rooted in the specific chemistries of the molecular metal precursors and reagents that must be chosen. It relates to entangled phenomena of nucleation and growth modulated by ligation, the intricate kinetics of which are not known with sufficient precision. Nevertheless, the outcome of the synthesis may be rationalized a posteriori in terms of structure and bonding analysis of the obtained clusters^9–13^. Nevertheless, much information is lost as the aforementioned approach is limited to just those few clusters that were stable enough and could be isolated from the reaction solution, eventually in very low yields with uncertain reproducibility^13–15^. These challenges were discussed within a wider context in our recent review article^16^.

Now, we report a novel concept and a methodology for generating and efficiently exploring ligated mixed-metal (heterometallic) clusters in the form of ‘living libraries’. Information is gained by directly dealing with chemical complexity without the separation of clusters. A living library is defined as a dynamic mixture of clusters, including growth species and additives such as ligands and reactants. Portfolios of cluster libraries can be generated for a given metal combination by settings of initial components and library evolution conditions. The libraries are populated with interrelated clusters, including transient and highly reactive ones, as well as more accessible but less reactive ones. Its distribution of clusters is sensitive to perturbation, for example, the interaction with reactants to be trapped or converted at the cluster surface that may also influence cluster structure rearrangement, growth or degradation reactions.

We demonstrate our methodology by using non-aqueous organometallic precursor chemistry under inert gas, tailored for living libraries of all-hydrocarbon ligated Cu/Zn clusters of the formula [Cu_*a*_Zn_*b*_](R)_*k*_ (metal atomicity ***n*** = *a* + *b*, coordination ***k*** with ligands R = H, 2,4,6-C_6_H_2_(CH_3_)_3_ = Mes or C_5_(CH_3_)_5_ = Cp*). Organometallic chemistry offers a unique toolbox for generating living libraries of clusters such as highly reactive metal precursors, prone to releasing metal atoms by reversible and irreversible fundamental reactions, including ligand exchange and transfer^17,18^, protolysis^19–21^, transmetallation^22,23^, oxidative addition/reductive elimination^24–26^, hydrogenolysis^27,28^ and so on.

**Fig. 1:**
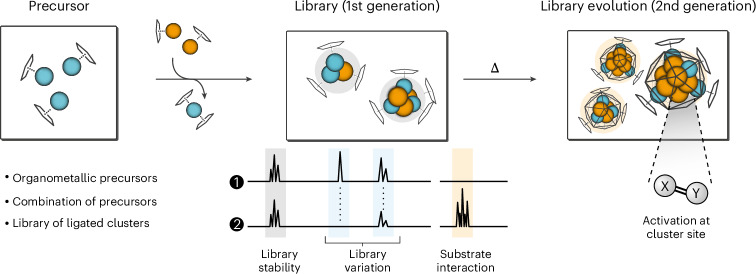
The living library concept. Mixed-metal cluster libraries are generated by combining highly reactive organometallic precursors at a defined set of conditions. Library perturbation is initiated by changing the conditions, for example, by the addition of substrates, and the evolution over time is monitored via mass spectrometry (Cu, orange; Zn, blue; carbon, grey; oxygen, red).

We selected the Cu/Zn system for two reasons. First, we have previously explored Cu/Zn clusters^29–32^. Our ‘embryonic brass’ chemistry proved very suitable for library generation owing to the variety of species formed and its high sensitivity to the reaction parameters. Second, there is interest in experimental and theoretical studies of atom-precise, sub-nanometre Cu_*n*_ clusters^33–36^, including hetero-metal-doped species^37^ in relation to the industrial methanol synthesis using Cu/ZnO/Al_2_O_3_ catalysts^38–41^. Liu et al. have evaluated the catalytic properties of the superatomic clusters Cu_*a*_Zn_*b*_ (*a* + *b* = 14) by a density functional theory (DFT) study^42^. Höltzl et al. explored the size- and charge-dependent CO_2_ and H_2_ activation on small clusters [Cu_*n*_Zn]^0/+^ (*n* = 3–6)^43^. Similarly, Li et al. reported on the electronic structure of a Cu–Zn dual atom catalyst site^44^, being highly selective in Cu-based alkyne semi-hydrogenation^45,46^. Our studies on a wide range of ligand-stabilized Cu/Zn clusters will thus broaden the understanding of Cu/Zn intermetallics at the molecular scale (Fig. 1).

## Results and discussion

### Living library generation

On combining Cu_5_Mes_5_ with decamethyldizincocene, Zn_2_Cp*_2_, as precursors, Zn(I) is oxidized to yield Zn(II) species, while Cu(I) is reduced to Cu(0). Cp* and Mes ligated Cu/Zn clusters are formed by choosing initial excess or specific quantities of either Cu_5_Mes_5_ or Zn_2_Cp*_2_ added after a particular incubation time (Fig. 1). For example, the library {[Cu_*a*_Zn_*b*_](R)_*k*_}, denoted as {**1**}, is reproducibly prepared from Cu_5_Mes_5_ (12.0 µmol) with 3.75 equiv. of Zn_2_Cp*_2_ (45.6 µmol) in dry toluene (0.50 ml) at 25 °C under argon as a dark-red solution within 120 min. Characterization of {**1**} by in situ liquid injection field desorption ionization mass spectrometry (LIFDI-MS) with a set-up coupled to a glove box^47^ gives more than 100 peak patterns up to the detection limit of our instrument (*m*/*z* = 6,000 a.m.u.), each pattern representing a unique species, which either is present in solution or is formed upon ionization and fragmentation. We were able to selectively synthesize, isolate and characterize a few clusters of {**1**}, for example, [**CuZn**_**2**_](Cp*)_3_ (**A**), [**Cu**_**3**_**Zn**_**4**_](Cp*)_5_ (**B**) and [**Cu**_**10**_**Zn**_**2**_](Cp*)_2_(Mes)_6_ (**C**)^29,31,32^. The vast majority of clusters present, however, exhibit previously unknown structures with a diversity of ***n*** and ***k***. This highlights both a rich chemical space to be explored and the challenge of isolation and characterization of individual clusters facing their very labile, air-, moisture- and temperature-sensitive nature. A methodology needs to be developed to efficiently exploit this chemical space towards the identification of those clusters, which hold promise for exciting properties and would justify the effort for in-depth investigations, including iterative size focusing, separation and isolation of specific clusters.

### Mass spectrometric library characterization

Each cluster library is characterized by its LIFDI mass spectrum, which allows for the identification of all ionizable molecular species in the reaction mixture at a given parameter set of library generation and evolution conditions. Two problems must be addressed. First, an exact and unique sum formula [Cu_*a*_Zn_*b*_](R)_*k*_ (R = Cp*_c_, Mes_*d*_, H_*h*_; *c* + *d* + *h* = *k*) has to be assigned to each pattern in the spectrum. This problem is solved by introducing a double labelling strategy with isotopically enriched ^68^Zn_2_Cp*_2_ (Δ*m*/*z* = 2.62) and Cp*^Et^ (Δ*m*/*z* = 14) for library preparation. This labelling strategy can certainly be done with ^2^H, ^13^C, and with other markers for R or reactants with other isotopically enriched metals M, thus matching the organometallic precursor chemistry used with the available MS instrument resolution. Second, a differentiation between molecular and fragment ions must be established to define a unique set of clusters that characterizes the library. This problem is solved by introducing varied collision energy experiments. The approach is akin to energy-dependent electrospray ionization mass spectrometry and stepped collision energy, known from peptide fragmentation analysis. Thus, we were able to reliably assign sum formulas [Cu_*a*_Zn_*b*_](R)_*k*_ to an extensive list of molecular ions for clusters present in the solution (Methods and Supplementary Table 2).

**Fig. 2:**
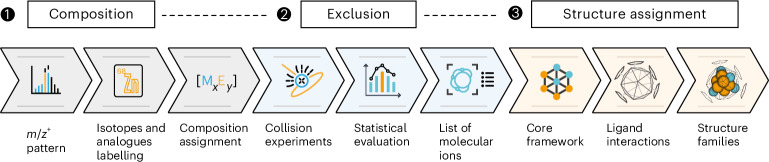
Workflow for the identification (1, 2) and structure assignment (3) of clusters. The libraries are analysed by mass spectrometry, including labelling and varied collision energy experiments, combined with a calculation framework using the obtained composition of the molecular ions of the clusters as the only input.

It is extremely difficult, ineffective or even impossible to separate all or at least the majority of clusters from a library and collect specific analytical data, including experimental structure determination of individual clusters. Furthermore, it is a priori unknown which of the clusters may be particularly interesting, could and should possibly be isolated, rigorously characterized, and justify the effort of scaled-up synthesis for more investigations. Therefore, a novel bias-free computational framework was developed to efficiently obtain well-founded suggestions of structures for any given candidate based on the sum formula derived from the libraries’ MS data analysis. The whole workflow for identification and structure assessment of clusters is depicted in Fig. 2. It guides the experiments with the 1st generation library {**1**}, and it can particularly be applied to reactivity investigations with substrates leading to the 2nd generation libraries {**2**}–{**4**}.

### Calculation framework for structural assignment

The calculation framework is based on the combination of DFT with data mining analysis techniques. This multi-step method is developed to obtain a reliable family of cluster structures without or with little additional information other than the sum formula deduced by the mass spectrometric library characterization (vide supra). The first step involves determining plausible metal core structure configurations without considering ligands, that is, the core’s frame. To obtain the initial structures, we used an internal subroutine, where each atom is iteratively added to the frame with the constraint that each atom must be placed inside defined cube and sphere boxes. Therefore, the input parameters are the total number of metal atoms (*n* = *a* + *b*) from a given Cu_*a*_Zn_*b*_ composition. In addition, the core frames can also be imported from the literature and included in the binary cores design. Then, the binary cores are generated by taking all possible permutations obtained by replacing the original atoms of the frames with *a* atoms of Cu and *b* atoms of Zn.

To attain feasible calculation times, the number of binary cores needs to be reduced using an appropriate filter. To exclude quasi-identical configurations, we encode the structures using the eigenvalues of their Coulomb matrices and apply the *k*-means clustering algorithm to automatically select several representative structures (one from each group). Then, those trial structures are optimized (for example, FHI-aims^48^) through DFT with low-computational self-consistency and geometry optimization parameters. After a set of Cu_*a*_Zn_*b*_ optimized cores are obtained, the ligands (R = Cp*, Mes, Hex, CO_2_, H,…) are distributed around the binary core to yield the desired [Cu_*a*_Zn_*b*_](R)_*k*_ clusters under two steps. First, *n* sites are generated and distributed (using a Fibonacci lattice) over a sphere that circumscribes the binary core, followed by a fine adjustment based on a coarse force-field process. Then, the ligands are added randomly or oriented by a predetermined distance to the given sites.

However, owing to the intrinsically unbiased and random nature of ligand distributions, certain structures may exhibit undesired configurations with overlapping atoms, particularly in the case of large metal cores and large numbers of ligands. Therefore, a covalent-radii-based filter was used as a cut-off to remove those configurations from the set. The resulting configurations are grouped into sets of quasi-identical structures. Then, the representative structures obtained through *k*-means are submitted to low-cost DFT calculations. Finally, as an optional step, the remaining representative structures can be submitted using tighter convergence parameters in the DFT calculations, particularly for properties. This leads to a family of local minimum structures for each Cu/Zn and other miscellaneous clusters.

We chose the two experimentally resolved and previously fully characterized [CuZn_2_](Cp*)_3_ (**A**) and [Cu_3_Zn_4_](Cp*)_5_ (**B**) to assess the validity of the calculation framework^29,31^. Starting with the sum formula only, families of 10 and 15 structures were generated for **A** and **B**, respectively. For both tests, the lowest energy configurations agreed with the experimental structures and DFT calculations, showcasing the great accuracy of the calculation framework with only ligand conformation distinguishing the lowest energy conformers. Nonetheless, clusters with a large number of core atoms and unusual structures become a challenge. However, this can be overcome by repeating the entire framework to guarantee a good sampling of structures. In addition, design-based structures from the computational living library can be incorporated into the final family of clusters to reinforce structures that exhibit specific physical–chemical experimental characteristics. In this way, reliable computational structures can be assigned to all clusters of interest (Supplementary Information).

### Probing cluster reactivity in libraries

To demonstrate the potential of our approach, we probed the perturbation of {**1**} by small molecules such as carbon dioxide, hydrogen and 3-hexyne (Fig. 4). An overview of our experiments is also provided in Supplementary Fig. 1.

**Fig. 3:**
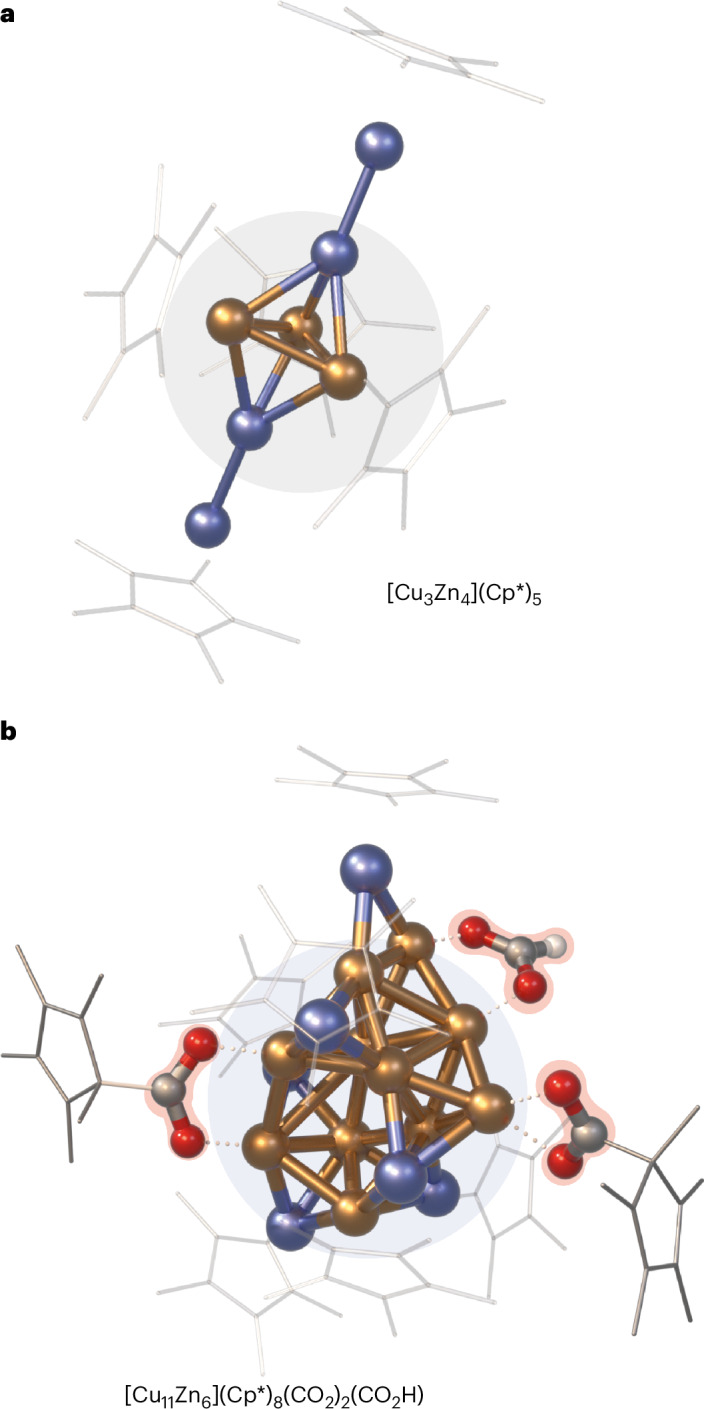
Results of the computational framework ‘*Cluster Assembler*’. **a**, Lowest energy structure of [Cu_3_Zn_4_](Cp*)_5_ (**B**) present in {**1**} showing a perfect match with the previously reported crystal structure^29^. **b**, Lowest energy structure of [Cu_11_Zn_6_](Cp*)_8_(CO_2_)_2_(HCO_2_) (**Z**) present in {**3**}. Colour code: Cu, orange; Zn, blue; C, grey; O, red; H, white; Cp* and ligands are depicted as wireframes for the sake of clarity.

**Fig. 4:**
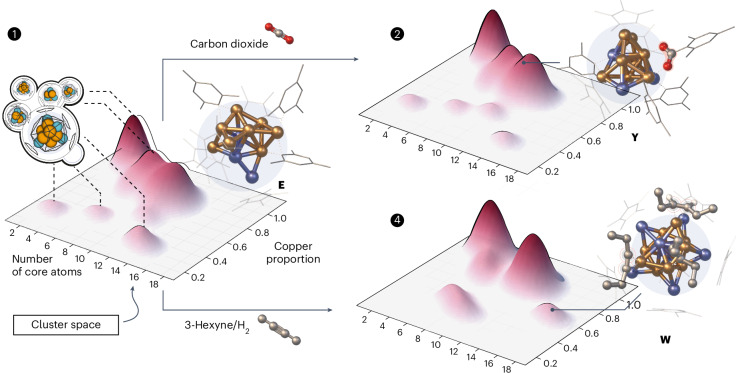
A selection of clusters [Cu_a_Zn_b_](R)_k_ identified in the libraries {1}, {2} and {4} are presented as heat maps based on the number of core metal atoms *n* = a + b and the Cu proportion. The relative abundance of different clusters with similar number *n* of core atoms and proportion of Cu are qualitatively shown by the intensity of the domes. The computed structures of examples of clusters that have not been isolated in pure form but identified by our methodology as important species during weak or strong perturbation of {**1**} and the derived libraries {**2**} and {**4**} are depicted: [HCu_8_Zn_3_](Cp*)_4_(Mes)_3_ (**E**) and the mesityl-carboxylate insertion product upon CO_2_ activation [Cu_8_Zn_3_](Cp*)_3_(Mes)_4_(CO_2_) (**Y**) occurring in {**2**}; computed structure of [Cu_9_Zn_7_](Cp*)_6_(Hex)_3_H_3_ (**W**) that occurs in {**4**} after treatment of {**1**} with 3-hexyne and H_2_. The notations (Hex)_3_ and (H)_3_ refer to 3 x C_6_H_10_ and 3 x H as part of the cluster’s composition deduced from the MS data. Colour code for the displayed structures: Cu, orange; Zn, blue; C, grey; O, red; H, white; Cp* and mesityl ligands are depicted as wireframes for the sake of clarity.

#### Reactivity towards CO_2_ and hydrogen

Treating {**1**} with CO_2_ reveals two new clusters, [Cu_5_Zn_5_](Cp*)_6_(CO_2_)_2_ (**X**, *m*/*z* = 1,543) and [Cu_8_Zn_3_](Cp*)_3_(Mes)_4_(CO_2_) (**Y**, *m*/*z* = 1,630) (Fig. 4). The unambiguous assignment was achieved by labelling with ^68^Zn_2_Cp*_2_ and ^13^CO_2_. The sum formulas for **X** and **Y** would also be in line with a CO_2_ splitting into M-CO and M-O (M = Cu, Zn), which was excluded by the absence of ν(CO) vibrations in the Fourier-transform infrared spectra (FT-IR) of {**2**} (Supplementary Fig. 135). Monitoring this weak perturbation of {**1**} to yield {**2**} via in situ ^1^H-NMR (Supplementary Fig. 131) suggests [H_3_Cu_6_Zn_5_](Cp*)_5_(Mes) (**D**) and [HCu_8_Zn_3_](Cp*)_4_(Mes)_3_ (**E**) as precursors for **X** and **Y** (Supplementary Figs. 12 and 13). Clusters **D** and **E** are the only species of {**1**} to disappear in {**2**}. The release of H_2_ and a formula unit CuMes (via NMR and LIFDI-MS) is assigned to the conversion **D** → **X** (Supplementary Fig. 12). The formation of HCp* and tetramethylfulvene, together with H_2_ evolution, is assigned to the conversion **E** → **Y** (Supplementary Fig. 13). The calculation framework proposes structures for **X** and **Y**. In **X**, both CO_2_ units feature a bent geometry (O–C–O angle of 129°) with monodentate coordination of O to Cu, while C coordinates to Zn and Cu sites in one case and solely to Zn in the other (Supplementary Fig. 14). This is reminiscent of the structures adopted by adsorbed CO_2_ on Fe_13_, Co_13_ and Ni_13_ clusters^49^. By contrast, **Y** shows an insertion of the activated CO_2_ into the hydrocarbon ligand sphere, forming a mesityl-carboxylate bridge to Cu sites (Supplementary Fig. 14).

Subsequent admission of H_2_ to this CO_2_-treated library {**2**} leads to a third library {**3**} revealing a strong perturbation with 14 species of {**2**} reacting off (Supplementary Table 5). However, both CO_2_-containing species **X** and **Y** of {**2**} remained, while a new formate-containing species [Cu_11_Zn_6_](Cp*)_8_(CO_2_)_2_(HCO_2_) (**Z**, *m*/*z* = 2305) was identified in {**3**} (Supplementary Figs. 6 and 15). The origin of **Z** could not be unambiguously traced back to any cluster of the original library {**1**}. Notably, the presence of clusters so far not identified by our methodology but possibly involved in the formation of **Z** cannot be excluded. The computed structure of **Z** (Fig. 3b and Supplementary Fig. 16) suggests an HCO_2_ moiety bridging two Cu atoms and flanked by a Zn atom, as well as two mesityl-carboxylate moieties linked to Cu sites, quite similar to **Y**. The formate species was confirmed via monitoring {**3**} by ^1^H-NMR (peak at 8.32 ppm (refs. ^50,51^); Supplementary Figs. 132 and 133) and FT-IR spectroscopy (bands at 1,380 cm^−1^ and 1,327 cm^−1^ (refs. ^52,53^); Supplementary Figs. 136 and 137). The occurrence of formate in our organometallic Cu/Zn library {**3**} is well in line with in situ FT-IR studies on Cu/ZnO, Cu/SiO_2_ and Cu/Al_2_O_3_ solid-state systems exposed to CO_2_/H_2_ mixtures^54,55^. It also reflects the mechanism for methanol formation on size-selected Cu_4_ clusters supported on Al_2_O_3_ (ref. ^38^).

#### Reactivity towards 3-hexyne and hydrogen

**Fig. 5:**
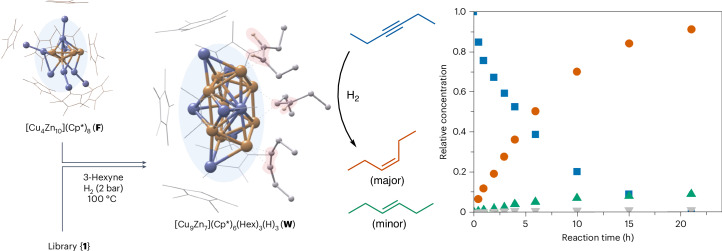
Catalytic semi-hydrogenation of 3-hexyne using either the whole library {4} or the pre-catalyst [Cu_4_Zn_10_](Cp*)_8_ (F) that was isolated from library {1}. Cluster **F** disappears in both cases and [Cu_9_Zn_7_](Cp*)_6_(Hex)_3_(H)_3_ (**W**) emerges while *cis*-3-hexene is formed with high selectivity (*cis*:*trans* = 9:1). The computed structure of **W** features various coordination modes of C_6_ species (Hex + 0, 1 or 2H) such as µ_1_η^2^-3-hexyne, µ_1_η^2^-*cis*-3-hexene and µ_2_η^1^-*cis*-3-hexenyl. The relative concentrations as a function of the reaction time of 3-hexyne (blue squares), *cis-*3-hexene (orange circles) and *trans*-3-hexene (green triangles), as well as *n*-hexane (grey trapezoids), are given for the catalytic conversion of 3-hexyne with 5 mol% **F** under a dihydrogen atmosphere at 100 °C.

Treating {**1**} with an excess of 3-hexyne and H_2_ yields the strongly perturbated {**4**}. In situ ^1^H-NMR reveals free 3-hexene (*cis*:*trans* = 9:1) without *n*-hexane formation. At least one of the species of {**4**} appears catalytically active in alkyne semi-hydrogenation. The cluster [Cu_9_Zn_7_](Cp*)_6_(Hex)_3_(H)_3_ (**W**, *m*/*z* = 2091) is the dominant C_6_-containing species (Hex = C_6_H_10_) of {**4**}, also including three additional H atoms (Supplementary Table 6). The computed structure of **W** (Fig. 5 and Supplementary Fig. 148) shows a distorted Cu_9_Zn pentagonal antiprismatic core with five ZnCp* moieties capping every second of the ten triangular faces and one ZnCp* capping the Cu_5_ pentagonal face. The other Cu_4_Zn pentagonal face contains the one Zn atom that is not ligated to Cp* as to interact with the other four Cu atoms, which bear the Cu-bound species µ_1_η^2^-3-hexyne (Hex + 0H), µ_1_η^2^-*cis*-3-hexene (Hex + 2H) and µ_2_η^1^-*cis*-3-hexenyl (Hex + 1H). These species and their coordination modes are beautifully in line with the alkyne semi-hydrogenation mechanism. Thus, we suggest **W** as a molecular model surface. All Cp* ligands are grouped to form a protective half-sphere around a Cu_5_Zn_6_ sub-surface structure and expose the non-ligated Cu_4_Zn plane for ‘adsorption’ of substrates. Evidently, selective synthesis and full experimental characterization of **W** is a target of our future efforts.

The composition of **W** deviates much from the 10 clusters of {**1**} that are reacting off to yield {**4**}. Identifying a particular cluster or a set of clusters related to the formation of **W** requires narrowing down the chemical space of {**1**}. Careful tweaking the preparation parameters of {**1**} allowed us to isolate and fully characterize the new cluster [Cu_4_Zn_10_](Cp*)_8_ (**F**) as one of the clusters reacting off when treating {**1**} with 3-hexyne and H_2_. Details on the X-ray single-crystal diffraction analysis and the corresponding DFT calculations used to rationalize the structure and bonding of **F** are given in the Supplementary Information.

Treatment of 3-hexyne with H_2_ in the presence of 5 mol% of isolated **F** leads to catalytic alkyne semi-hydrogenation with near-perfect selectivity (Fig. 5 and Supplementary Figs. 151–153). The highest activity was observed at 100 °C with a quantitative conversion and a *cis*:*trans* ratio of 9:1 after 21 h. Monitoring the catalytic test reaction by LIFDI-MS reveals the quantitative consumption of **F** and the formation of **W** (Supplementary Fig. 145) without significant abundance of other clusters. Thus, we assign **F** as a pre-catalyst and regard **W** as likely to be involved in the catalytic cycle. After 4 h, 48% of the 3-hexyne was converted with TOF (turnover frequency) = 0.60 h^−1^ Cu^−1^ compared with TOF = 0.15 h^−1^ Cu^−1^ for library {**4**} that is derived from {**1**} at the same conditions. Notably, {**1**} contains a relatively small amount of **F** besides inactive clusters. The transformation **F** → **W** occurs under the elimination of Zn_2_Cp*_2_ and traces of [Cu_3_Zn_4_](Cp*)_5_ (**B**) and by restructuring towards a Cu-enriched core. The by-product Zn_2_Cp*_2_ decomposes rapidly to elemental zinc under the conditions (Supplementary Fig. 146).

## Conclusion

We introduced the concept of living libraries using Cu/Zn clusters as an example and reported a methodology for probing their chemical space directly without separating and isolating individual clusters. Conclusions regarding the reactive properties of the clusters can be obtained by monitoring the library evolution upon weak and strong perturbations. A weak perturbation is caused by CO_2_ to yield CO_2_-activated species bound at specific clusters while many remain inert. A strong perturbation is caused by 3-hexyne and H_2,_ with many clusters reacting off during alkyne semi-hydrogenation. Here we demonstrated the targeted synthesis, isolation and full characterization of [Cu_4_Zn_10_](Cp*)_8_ (**F**), establishing it as a pre-catalyst. In turn, we discovered [Cu_9_Zn_7_](Cp*)_6_(Hex)_3_H_3_ (**W**) as a molecular surface model likely to be involved in the catalytic cycle. Altogether, the examples showcase the usefulness of the living library approach for efficiently exploring a diverse (hetero)metallic cluster landscape. The concept is ready to be transferred to related metal combinations such as Cu/Al^13^ and Ni/Ga^56^. Our approach holds much promise for cluster science in general as it may enable exploiting organometallic precursor chemistry to systematically identify novel, highly reactive, even catalytically active, all-hydrocarbon ligated mixed metal clusters across the periodic table.

## Methods

### General

All manipulations were carried out using standard Schlenk techniques under inert atmospheres. Solvents were dried using an MBraun solvent purification system. The final H_2_O content of all solvents was measured via Karl Fischer titration and was below 5 ppm. The organometallic precursors Cu_5_Mes_5_ (ref. ^57^) and Zn_2_Cp*_2_ (ref. ^58^) were synthesized and characterized according to literature procedures.

^1^H-NMR spectra were recorded on a Bruker AVIII 400 US spectrometer (400 MHz) in benzene-*d*_6_ or toluene-*d*_8_. Chemical shifts (*δ*, ppm) and referenced to the solvent resonances as internal standards and corrected tetramethylsilane as the standard. LIFDI-MS was measured via sample transfer under an argon atmosphere (glove box) using a Thermo Fisher Scientific Exactive Plus Orbitrap (mass range up to 6,000 a.m.u.; mass accuracy 3 ppm; external calibration) that was equipped with an LIFDI source from Linden CMS. The FT-IR spectra were taken on an ALPHA-T FT-IR spectrometer from Bruker with a transmission cell unit under an argon atmosphere (glove box) with 512 scans per measurement and a resolution of 4 cm^−1^. The spectra were evaluated using the software OPUS.

The computational framework for structure generation is compiled in an iterative Python code, namely *Cluster Assembler*, which is available at 10.5281/zenodo.8136872.

### Library generations and evolutions

The Cu/Zn library {**1**} was prepared by reacting 1.0 equiv. Cu_5_Mes_5_ (for example, 24.0 mol, 22.0 mg) with 3.75 equiv. Zn_2_Cp*_2_ (for example, 90.0 µmol, 36.6 mg) in 1.0 ml dry toluene for 2 h before analysis via LIFDI-MS either using a flame-dried Schlenk tube or in a flame-dried J-Young NMR tube. Labelling was carried out with ^68^Zn_2_Cp*_2_ and Zn_2_Cp*^Et^_2_ accordingly, with quantities of 37.0 mg and 39.6 mg, respectively. The library generation can be scaled when keeping the overall concentration and molar ratio of the reactants constant.

The evolution of library {**1**} to library {**2**}, that is, perturbation with CO_2_, was conducted by pressurizing a sample of 0.5 ml {**1**} with 1 bar of CO_2_ in a flame-dried J-Young NMR tube after freeze–pump–thaw degassing. After 18 h reaction time, the CO_2_ adducts [Cu_5_Zn_5_](Cp*)_6_(CO_2_)_2_ (**X**) and [Cu_8_Zn_3_](Cp*)_3_(Mes)_4_(CO_2_) (**Y**) were observed by LIFDI-MS analysis. Reactions with ^13^CO_2_ are performed accordingly.

The evolution of library {**2**} to library {**3**}, that is, the subsequent perturbation of {**1**} with CO_2_ and then with H_2_, was conducted by pressurizing 0.5 ml {**1**} in a flame-dried J-Young NMR tube with 1 bar of CO_2_ to obtain {**2**} as described earlier. After a reaction time of 18 h at room temperature, CO_2_ was released from the tube (glove box). The tube was then pressurized with 2 bar H_2_ after freeze–pump–thaw degassing. Formate is detected by ^1^H-NMR after 4 h reaction time and [Cu_11_Zn_6_](Cp*)_8_(CO_2_)_2_(HCO_2_) (**Z**) is detected via LIFDI-MS.

The evolution of library {**1**} to library {**4**}, that is, the simultaneous treatment with 3-hexyne and H_2_, was conducted by placing 0.5 ml of {**1**} in a flame-dried J-Young NMR tube together with 5.0 μl 3-hexyne (44.0 μmol, 3.66 equiv. based on Cu) and pressurizing with 2 bar H_2_ after freeze–pump–thaw degassing. After 4 h at 100 °C, 3-hexene is detected by ^1^H-NMR and [Cu_9_Zn_7_](Cp*)_6_(Hex)_3_(H)_3_ (**W**) is detected via LIFDI-MS.

### Mass spectrometric library characterization

The assignment of sum formulas for clusters is based on the *m*/*z* value and the isotopic pattern of the observed molecular ions in the LIFDI-MS experiment. Differences of one H atom are well recognized by a shift of Δ*m*/*z* = 1. However, the difference in atomic masses of Cu and Zn is also only 1. When including the possibility of cluster hydrides, it leads to a very large number of possible compositions for each *m*/*z* value. The mass accuracy of 3 ppm and the instrumental resolution at optimum sensitivity of 35,000 prevents us from using the isotopic pattern alone for unambiguous identification, even for medium-sized clusters (Supplementary Fig. 18). A double labelling strategy was applied to solve this problem by introducing Cp*^Et^ (Δ*m*/*z* = 14) and isotopically enriched ^68^Zn_2_Cp*_2_ (Δ*m*/*z* = 2.62). The clusters [Cu_*a*_Zn_*b*_](R)_*k*_ (R = Cp*_c_, Mes_*d*_, H_*h*_; *c* + *d* + *h* = *k*) are generally ligated by Cp* and Mes, while H may occur as well. The number of Cp* ligands ***c*** of a species can thereafter be determined by scanning the Cp*^Et^ labelled spectrum for peaks with a Δ*m*/*z* shift of ***c*** x 14. The number of matches for each peak is thus reduced to species carrying the same number ***c*** of Cp*. For multiple computed compositions with the same ***c***, matching the experimental patterns and unambiguous peak identification was achieved by the second labelling experiment with ^68^Zn_2_Cp*_2_. Thus, definite sum formulas [Cu_*a*_Zn_*b*_](R)_*k*_ (R_*k*_ = Cp*_c_, Mes_*d*_, H_h_; *c* + *d* + *h* = *k*) were assigned to nearly every observed peak pattern, including the unique deconvolution of overlapping patterns. The results are given in Supplementary Table 1.

Collision experiments enable discrimination between molecular ions and fragments. The collision energy in the higher-energy collisional dissociation cell (HCD) of the ORBITRAP mass spectrometer was increased stepwise, and a spectrum of the original cluster library was recorded for each collision energy. The intensities (*I*) of the peaks of interest were determined for the different collision energies by integrating them relative to the overall integral of the respective spectrum. Molecular ion peaks are associated with a continuous decrease in peak intensity for increasing collision energies. This is due to the enhanced fragmentation of parent ions at higher collision energies. For fragment ions, an increase in peak intensity is expected for increasing collision energies owing to their enhanced formation at higher collision energies. The approach is akin to energy-dependent electrospray ionization mass spectrometry and stepped collision energy known from peptide fragmentation^59,60^. The resulting *I* versus collision energy plots for every ion pattern of {**1**} are shown in Supplementary Information (Supplementary Figs. 73–129). For some species, no significant variation in peak intensity was detected. Hence, discrimination between a fragment and a molecular ion failed in these few cases. However, the method is reliable for the great majority of the peaks and reliably yields the assignment of sum formulas to an extensive list of molecular ions for clusters present in the solution (Supplementary Table 2). This methodology can also be applied to reactivity investigations of cluster libraries (vide supra).

### Synthesis and characterization of the new cluster [Cu_4_Zn_10_](Cp*)_8_ (F)

Samples of Cu_5_Mes_5_ (420 mg, 0.46 mmol, 1.00 equiv.) and Zn_2_Cp*_2_ (876 mg, 2.18 mmol, 4.7 equiv.) were dissolved in toluene (75 ml) and stirred at room temperature for 2 h. After concentrating the solution under reduced pressure to 8 ml, the mixture was filtered and left to crystallize at −30 °C for 6 days. Then, the mother liquor was filtered off at −30 °C, and the deposited crystals were washed thoroughly with *n*-hexane (4 × 10 ml) and filtered off with cooling. The volume of the combined filtrates was further reduced in vacuo and left to crystallize again at −30 °C for a few days to give another batch of crystals. The combined crude product still contains significant quantities of [CuZn_2_]Cp*_3_ (**A**). It was then further purified by careful decantation by suspending the collected crystals in *n*-hexane (3 × 10 ml) and stirring it strongly before letting the yellow-orange [CuZn_2_]Cp*_3_ (**A**) settle for a few seconds and decanting off the black suspension using a narrow Teflon cannula. The resulting black suspension was allowed to settle and decanted, and the remaining crystals were dried in vacuo at room temperature. The remaining impurities of [CuZn_2_]Cp*_3_ (**A**) were removed by crystal picking from that sample under the microscope in the glove box to yield a homogeneous black crystalline sample of [Cu_4_Zn_10_](Cp*)_8_ (**F**, 80 mg, 40.2 µmol, 7% based on Cu). Analytical data of **F**: ^1^H-NMR (400 MHz, toluene-*d*_8_): *δ* (ppm) = 2.27 (s, 45H), 2.21 (s, 75H); ^1^H-NMR (400 MHz, C_6_D_6_): *δ* (ppm) = 2.30 (s, 45H), 2.24 (s, 75H). ^13^C-NMR (101 MHz, Tol-*d*_8_): *δ* (ppm) = 110.4 (s, C_5_Me_5_), 107.6 (s, C_5_Me_5_), 12.6 (s, C_5_Me_5_), 10.6 (s, C_5_Me_5_). LIFDI-MS (*m*/*z*): found [M]^+^ 1989.9273 (calculated 1989.9327). Elemental analysis (C, H, Cu, Zn) yielded non-satisfactory results owing to still not fully removed impurities of **A** (traces detected by ^1^H-NMR).

### Single-crystal X-ray crystallography

A black, plate-shaped crystal of **F**, C_80_H_120_Cu_4_Zn_10_, coated with perfluorinated ether and fixed on top of a Kapton micro sampler was used for X-ray crystallographic analysis. The X-ray intensity data were collected at 100(2) K on a Bruker D8 VENTURE three-angle diffractometer with a TXS rotating anode with Mo K_α_ radiation (*λ* = 0.71073 Å) using APEX4 (ref. ^61^). The diffractometer was equipped with a Helios optic monochromator, a Bruker PHOTON-100 CMOS detector and a low-temperature device. The results of the structure solution and refinement are given in the Supplementary Information.

### Bonding analysis

DFT calculations at the BP86/TZ2P/D3(BJ) level were carried out specifically on [Cu_4_Zn_10_](Cp*)_8_ (**F**) to guide and ascertain the Cu versus Zn nature of the M positions in the experimental structure obtained via single-crystal X-ray diffraction and to provide a rationalization of its electronic structure. The DFT calculations were carried out with the use of the Amsterdam Density Functional code (ADF2020.101)^62^, incorporating scalar relativistic corrections via the ZORA Hamiltonian^63,64^. The BP86 functional^65,66^ was used, with the addition of Grimme’s D3(BJ) empirical corrections^67,68^ to account for dispersion effects. All the geometry optimizations were performed with the all-electron triple-ξ Slater basis set plus two polarization functions (STO-TZ2P)^69^. Natural atomic charges and Wiberg bond indices were computed with the natural bond orbital NBO6.0 programme^70^ implemented in the ADF2020.101 package.

From the electron counting point of view, the number of cluster metal valence electrons (discarding 3d electrons) of [Cu_4_Zn_10_](Cp*)_8_ (**F**) is 4 + (10 × 2) − 8 = 16. Assuming that the Zn–Zn bonds in the four Zn–ZnCp* units are localized 2-electron/2-centre bonds, the number of electrons associated with metal–metal bonding within the coordination sphere of the central Cu atom is 16 − (4 × 2) = 8. Together with the 10 non-bonding 3d(Cu) electrons, this results in a central Cu atom following the 18-electron rule, analogous to a regular ML_*n*_ organometallic complex (Supplementary Fig. 144). A full discussion of the results is given in the Supplementary Information.

### Catalytic test reaction

A sample of [Cu_4_Zn_10_](Cp*)_8_ (**F**) (2.50 mg, 1.26 μmol, 1.00 equiv.) obtained as described earlier and a sample of 3-hexyne (2.90 μl, 25.1 μmol, 20 equiv.) were dissolved in toluene-*d*_8_ (0.50 ml) and placed in a flame-dried J-Young NMR tube. After degassing the solution, the mixture was pressurized with H_2_ (2 bar) and heated to 100 °C for 21 h to obtain quantitative conversion of the 3-hexyne to *cis*-3-hexene and *trans*-3-hexene in a molar ratio of 9:1 obtained by ^1^H-NMR signal integration: ^1^H-NMR (400 MHz, Tol-*d*_8_): *δ* (ppm) = 5.41 (tt, 2H, H_*trans*_), 5.35 (ddd, 2H, H_*cis*_). Treating 0.5 ml of library {**1**} with 3-hexyne and H_2_ under the same conditions yields the same result. Note: Using Cu_5_Mes_5_ or Zn_2_Cp*_2_ or the cluster [CuZn_2_]Cp*_3_ (**A**) as pre-catalysts instead of a sample of **F** did not show any conversion of 3-hexyne. Also, to exclude catalysis induced by any metal nanoparticles (Zn, Cu, Cu/Zn), test reactions were performed in the presence of elemental mercury, known to amalgamate with metals and thus passivate nanoparticles^56^, and no change in the catalytic performance of our system was observed.

## Online content

Any methods, additional references, Nature Portfolio reporting summaries, source data, extended data, supplementary information, acknowledgements, peer review information; details of author contributions and competing interests; and statements of data and code availability are available at 10.1038/s41557-024-01726-3.

## Supplementary information

Supplementary Information Supplementary Discussions, Figs. 1–154, Tables 1–39, LIFDI mass spectra, NMR spectra, IR spectra, SC-XRD data and Computational results.

Supplementary Data 1 XYZ coordinates of the optimized lowest energy isomer of compound **F**.

Supplementary Data 2 XYZ coordinates of the optimized other isomers of compound **F**.

## Data Availability

Crystallographic data for the structures of **A**, **B** and **F** are available at the Cambridge Crystallographic Data Centre under deposition numbers 1013577, 1854852 and 2390361, respectively. Copies of the data can be obtained free of charge via https://www.ccdc.cam.ac.uk/structures/. Full experimental (LIFDI-MS, IR, NMR) and computational datasets are available from the Research Data Service MediaTUM at 10.14459/2023mp1715745.

## Code availability

We provided the procedure for structure generation compiled in an automated Python code, namely *Cluster Assembler* (v.1.2.0), available at 10.5281/zenodo.12819855.
