# Supplementary material for: A living library concept to capture the dynamics and reactivity of mixed-metal clusters for catalysis
Source: Nat Chem. 2025 Jan 23;17(4):525–31. doi: 10.1038/s41557-024-01726-3 (PMC11964927; doi:10.1038/s41557-024-01726-3)
Supplement: Supplementary file 1 — Supplementary Discussions, Figs. 1–154, Tables 1–39, LIFDI mass spectra, NMR spectra, IR spectra, SC-XRD data and Computational results. [file 41557_2024_1726_MOESM1_ESM.pdf]

nature chemistry

Supplementary information

https://doi.org/10.1038/s41557-024-01726-3

A living library concept to capture the dynamics and reactivity of mixed-metal clusters for catalysis

In the format provided by the authors and unedited

Table of Contents

1. Experimental Section 3

1.1. Cu/Zn Libraries 3

1.1.1. Mass Spectrometric Characterization 4

1.1.1.1. LIFDI Mass Spectra 4

1.1.1.2. Composition Tables 9

1.1.1.3. Peak Identification 17

Molecular Ions 18

Fragments 28

1.1.1.4. Fragmentation Analysis 37

1.1.2. NMR Spectra 68

1.1.3. FT-IR Spectra 71

1.2. $[Cu_4Zn_{10}]Cp^*_8$ (F) 73

1.2.1. Experimental Characterization 73

1.2.1.1. Single-Crystal Crystallography 74

1.2.2. DFT Bonding Analysis 77

1.3. Catalytic Semi-Hydrogenation 81

1.4. References 86

2. Computational Framework 87

2.1. Introduction to the Generation of Metal Complexes 87

2.2. Additional Computational Details on the Framework for the Generation of Metal Complexes 89

2.2.1. Generation of Molecular Unary Frames 89

2.2.2. Clustering of Molecular Structures via Coulomb Matrix Representation 90

2.2.3. Generation of the Binary Core Structures 92

2.2.4. Low-cost Geometric Optimizations for the Core 92

2.2.5. Generation of Sites on the Core Surface 93

2.2.6. Addition of Ligands on the Core Surface 93

2.2.7. Complexes Filtering 94

2.2.8. Representative Complexes via Clustering Algorithm 94

2.2.9. Low-cost Geometric Optimization of Metal Complexes 94

2.2.10. Optimization with Tight Criteria 95

2.3. Additional Details on the Theoretical Approach and Computational Details for the Optimization Calculations 95

2.4. Cluster_Assembler-Documentation 96

2.5. Computational Parameters to Generate the Family of Metal Complexes 99

2.6. Additional Results for the A_aB_b Cores 100

2.6.1. $[CuZn_2]$ 100

2.6.2. $[Cu_3Zn_4]$ 102
2.6.3. $[Cu_5Zn_5]$ 111
2.6.4. $[Cu_6Zn_5]$ 119
2.6.5. $[Cu_8Zn_3$ 125
2.6.6. $[Cu_4Zn_{10}]$ 137
2.6.7. $[Cu_{11}Zn_6]$ 148
2.6.8. $[Cu_8Al_6]$ 162
2.6.9. $[Ni_7Ga_6]$ 171
2.7. Additional Results for Metal Complexes 180
2.7.1. $[CuZn_2](Cp^*)_3$ Complexes 181
2.7.2. $[Cu_3Zn_4](Cp^*)_5$ Complexes 182
2.7.3. $[Cu_5Zn_5](Cp^*)_6(CO_2)_2$ Complexes 198
2.7.4. $[Cu_6Zn_5](Cp^*)_5(Mes)H_3$ Complexes 211
2.7.5. $[Cu_8Zn_3](Cp^*)_3(Mes)_4CO_2$ Complexes 223
2.7.6. $[Cu_8Zn_3](Cp^*)_4(Mes)_3H$ Complexes 237
2.7.7. $[Cu_9Zn_7](Cp^*)_6(Hex)_3H_3$ Complexes 247
2.7.8. $[Cu_{11}Zn_6](Cp^*)_8(CO_2)_2(HCO_2)$ Complexes 252
2.7.9. $[Cu_8Al_6](Cp^*)_6$ Complexes 258
2.7.10. $[Ni_7Ga_6](Cp^*)_6$ Complexes 270
2.8. References 280

1. Experimental Section

1.1. Cu/Zn Libraries

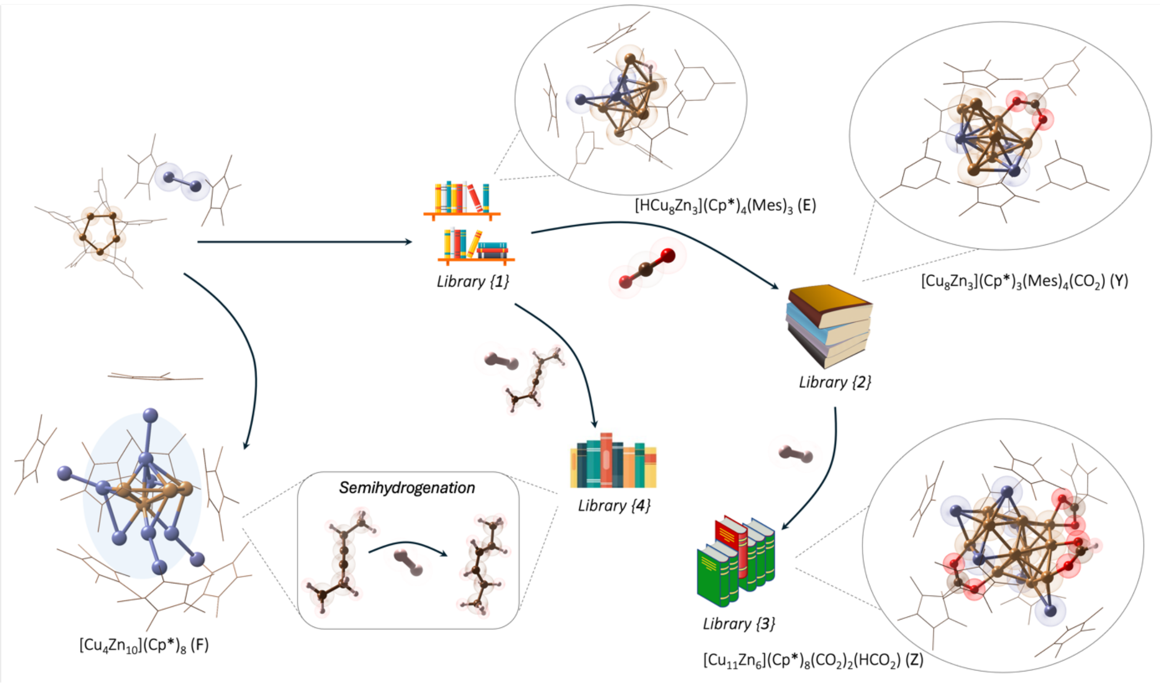

Supplementary Figure 1: Schematic overview of the different reactions investigated. The reaction of copper mesityl with $Zn_2Cp^*_2$ leads to the formation of library {**1**}, that contains, among others, the clusters $[H_3Cu_6Zn_5](Cp^*)_5(Mes)$ (**D**) and $[HCu_8Zn_3](Cp^*)_4(Mes)_3)$ (**E**) and $[Cu_4Zn_{10}](Cp^*)_8$ (**F**). Cluster **F** can be enriched, isolated and obtained in acceptable purity form by targeted optimization of the synthesis parameters of {**1**}. The treatment of {**1**} with CO_2 leads to the formation of library {**2**}, containing the clusters $[Cu_5Zn_5](Cp^*)_6(CO_2)_2$ (**X**) and $[Cu_8Zn_3](Cp^*)_3(Mes)_4(CO_2)$ (**Y**). The treatment of {**2**} with H_2 leads to the formation of library {**3**}, in particular containing the formate bearing cluster $[Cu_{11}Zn_6](Cp^*)_8(CO_2)_2(HCO_2)$ (**Z**). Library {**4**}, obtained from {**1**} under treatment with 3-hexyne and H_2, is catalytically active towards the semi-hydrogenation of 3-hexyne. The isolated $[Cu_4Zn_{10}](Cp^*)_8$ (**F**) acts as a pre-catalyst for the semi-hydrogenation of 3-hexyne to cis-3-hexene.

1.1.1. Mass Spectrometric Characterization

1.1.1.1. LIFDI Mass Spectra

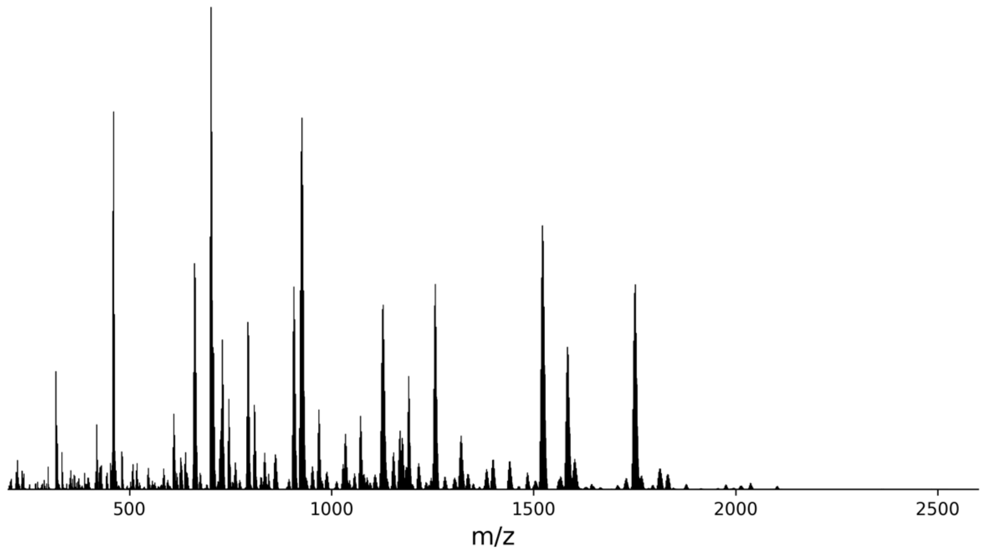

Supplementary Figure 2: Full range LIFDI mass spectrum of the Cu/Zn library {**1**}.

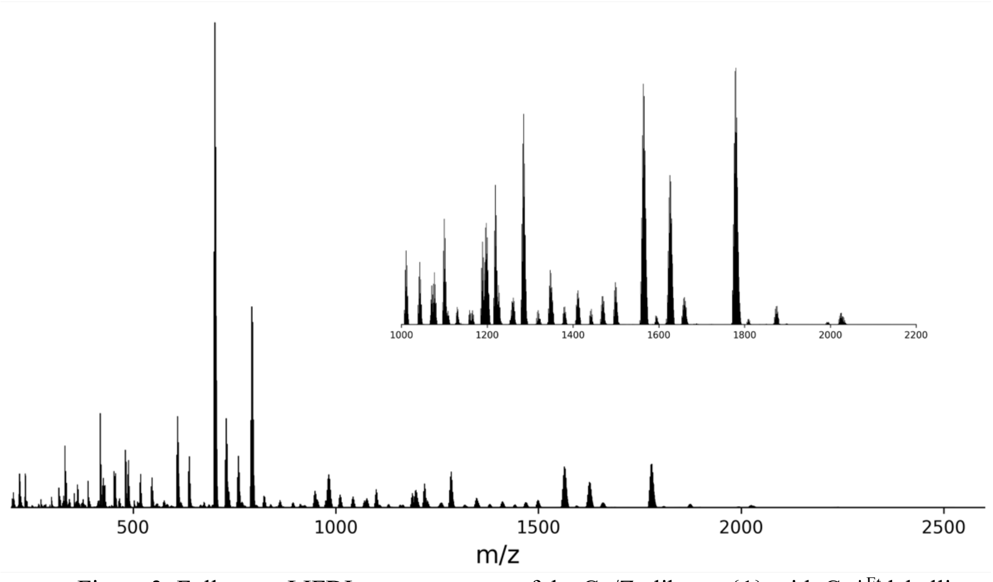

Supplementary Figure 3: Full range LIFDI mass spectrum of the Cu/Zn library {**1**} with Cp*^{Et} labelling.

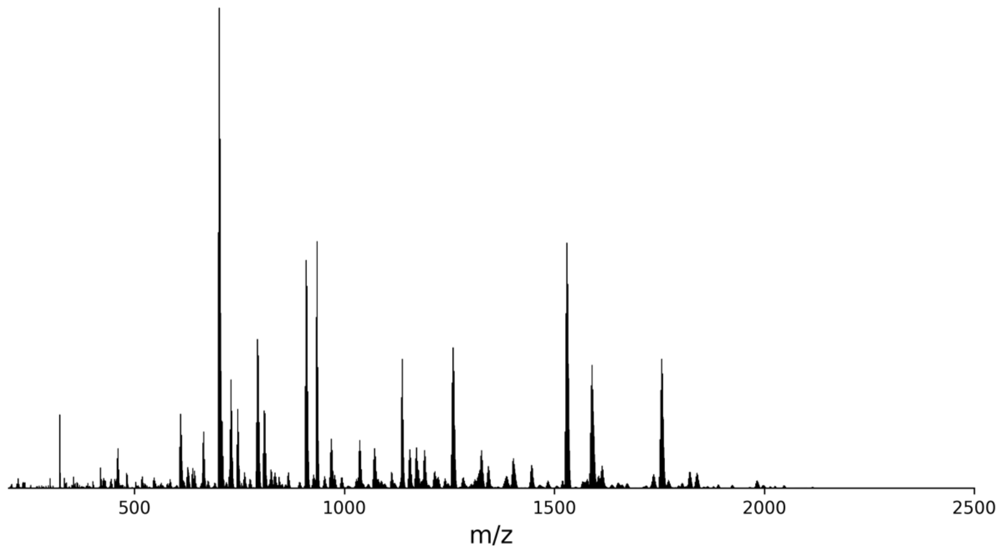

Supplementary Figure 4: Full range LIFDI mass spectrum of the Cu/Zn library {**1**} with ^{68}Zn labelling.

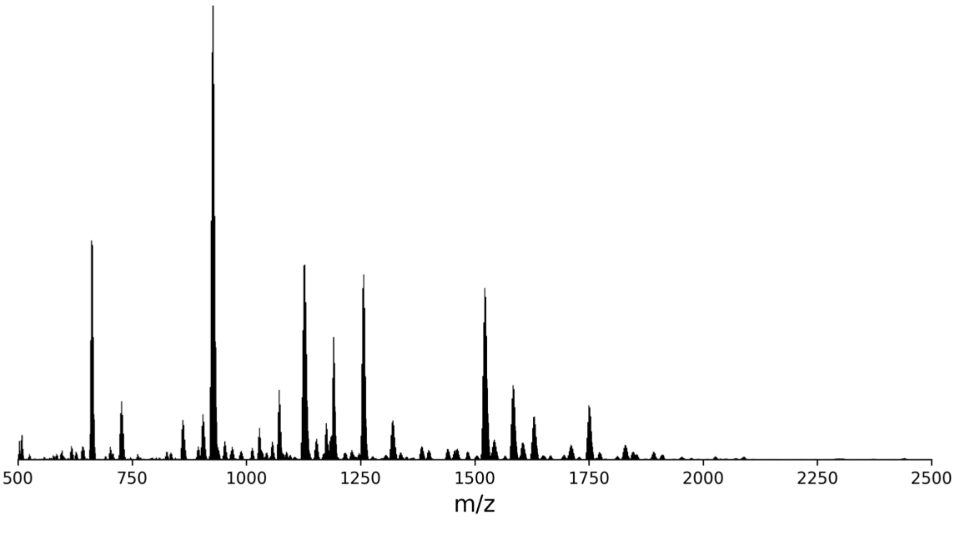

Supplementary Figure 5: Full range LIFDI mass spectrum of the reaction of the Cu/Zn library {**1**} with CO_2 yielding {**2**}.

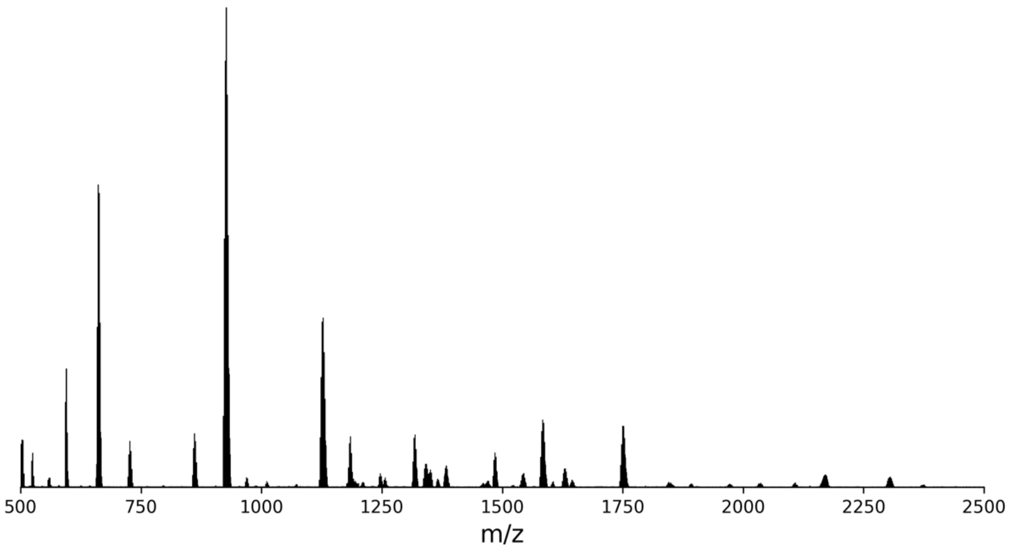

Supplementary Figure 6: Full range LIFDI mass spectrum of the reaction of the Cu/Zn library {**1**} with CO_2 and H_2 yielding library {**3**}.

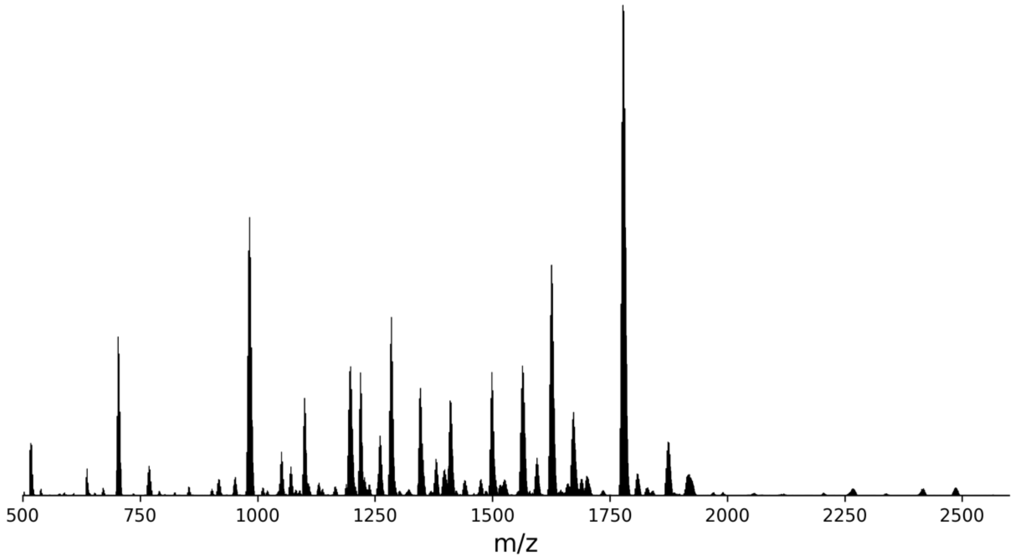

Supplementary Figure 7: Full range LIFDI mass spectrum of the reaction of the Cu/Zn library {**1**} with CO_2 and H_2 with Cp^{*Et} labelling yielding {**3**}.

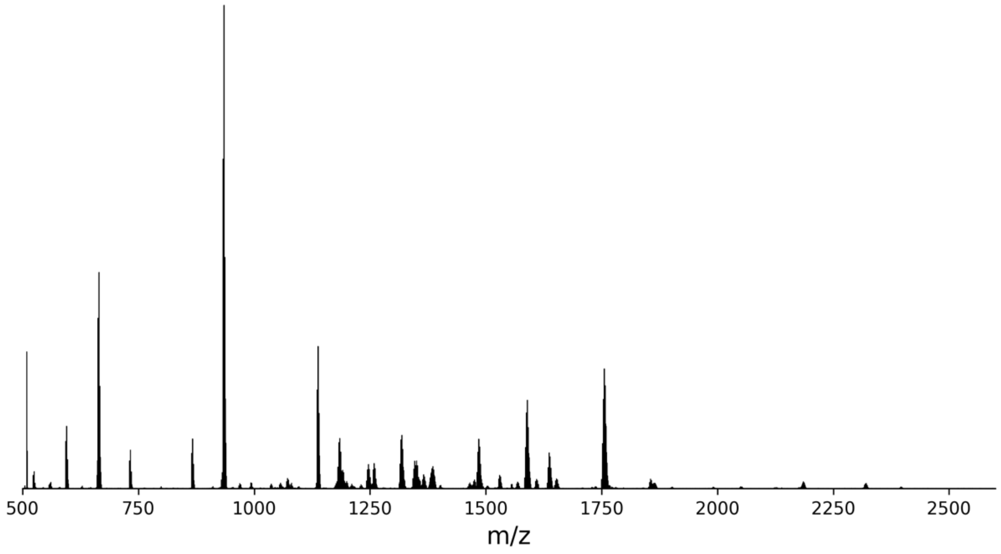

Supplementary Figure 8: Full range LIFDI mass spectrum of the reaction of the Cu/Zn library {**1**} with CO_2 and H_2 with ^{68}Zn labelling yielding {**3**}.

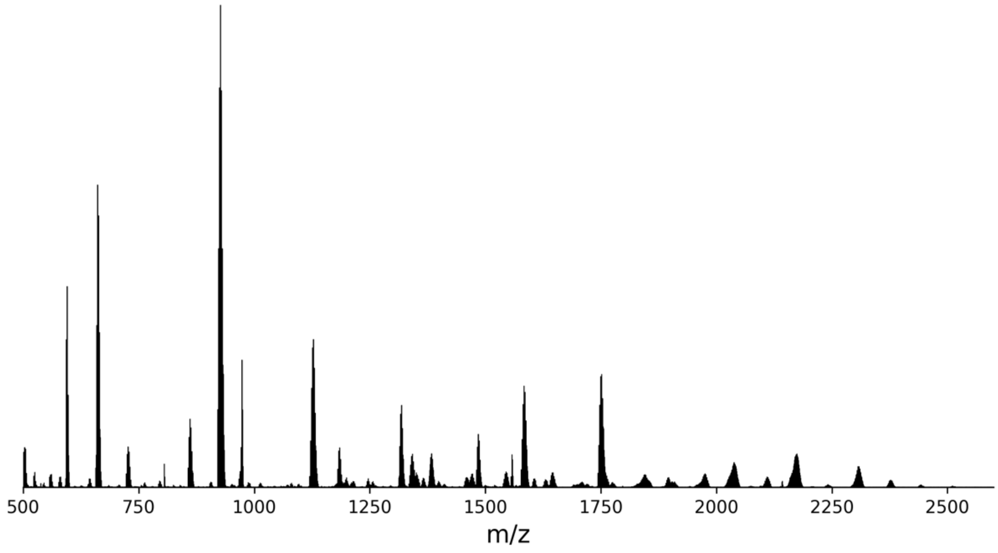

Supplementary Figure 9: Full range LIFDI mass spectrum of the reaction of the Cu/Zn library {**1**} with $^{13}CO_2$ and H_2 yielding library {**3**}.

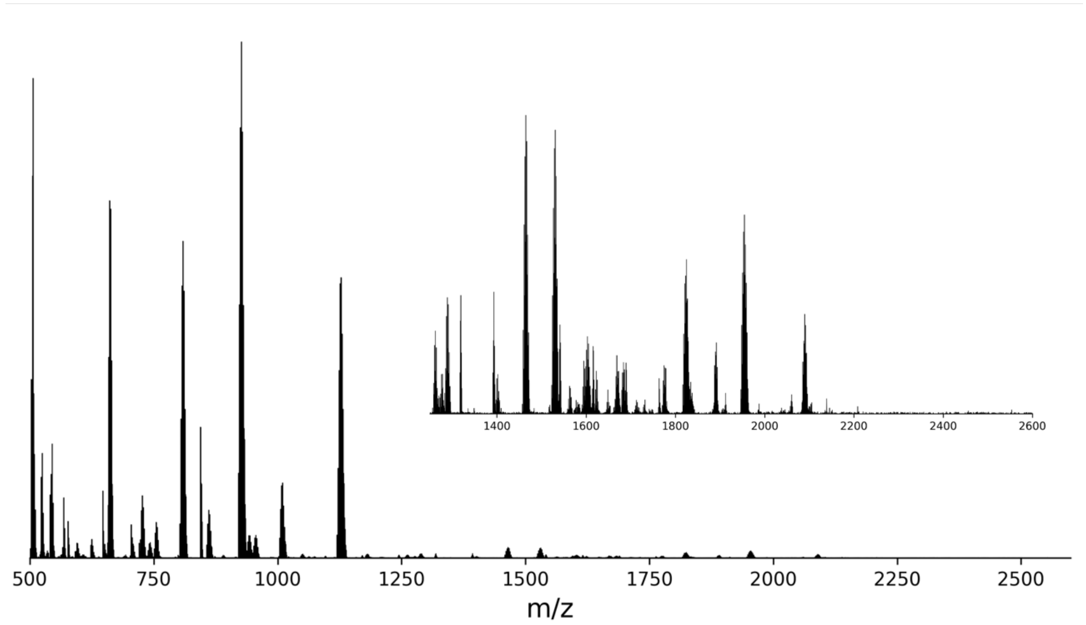

Supplementary Figure 10: Full range LIFDI mass spectrum of the reaction of the Cu/Zn library {**1**} with 3-hexyne and H_2 yielding {**4**}.

1.1.1.2. Composition Tables

Supplementary Table 1:List of all identified patterns in the LIFDI mass spectrum of the Cu/Zn library {**1**} with composition and m/z, as well as indications if a species has been identified by Cp*[Et] labelling, [68]Zn labelling or both.

Composition	*m/z*	*Cp^{*Et} Label*	*^{68}Zn Label*
ZnCp*Mes	318,1289	✓	✓
$[CuZn](Cp^*)_2$	399,0865	✓	✓
$[Cu_3](Cp^*)_2$	461,0168	✓	✓
$[HCu_4](Cp^*)_2$	524,9531	✓	✓
$[H_2Cu_3](Cp^*)_3$	598,1309	✓	✓
$[CuZn_2](Cp^*)_3$ (**A**)	600,1593	✓	✓
$[H_3Cu_2Zn](Cp^*)_3$	600,1593	✓	✓
$[Cu_4](Cp^*)(Mes)_2$	627,0009	✓	✓
$[Cu_4](Cp^*)_2(Mes)$	643,0310	×	✓
$[HCu_3Zn](Cp^*)_3$	661,0653	✓	✓
$[Cu_2Zn_3](Mes)_3$	678,9015	✓	✓
$[HCu_5](Cp^*)_2(Mes)$	706,9671	✓	✓
$[HCu_7](Cp^*)_2$	715,7538	✓	✓
$[HCu_3Zn_2](Cp^*)_3$ {-2H}	724,9912	✓	✓
$[Cu_3Zn](Mes)_4$	731,0599	✓	✓
$[Cu_4](Cp^*)(Mes)_3$	746,0845	✓	✓
$[HCu_5Zn](Cp^*)_3$	788,924	✓	✓
$[Cu_4Zn_2](Cp^*)_3$	788,924	✓	✓
$[Cu_5](Mes)_4$	792,9827	✓	✓
$[Cu_5](Cp^*)(Mes)_3$	809,0139	×	✓
$[Cu_5](Cp^*)_2(Mes)_2$	825,0443	✓	✓
$[HCu_7](Cp^*)_2(Mes)$	834,8236	✓	✓
$[HCu_7](Cp^*)_3$	850,8526	×	✓
$[Cu_3Zn_2](Cp^*)_4$	861,1030	✓	✓
$[Cu_5Zn](Cp^*)_3(Mes)$	907,0021	✓	✓
$[Cu_3Zn_3](Cp^*)_4$	927,0291	✓	✓
$[Cu_7](Cp^*)_2(Mes)_2$	952,9009	✓	✓
$[Cu_7](Cp^*)_3(Mes)$	968,9302	×	✓
$[Cu_5Zn_2](Cp^*)_4$	988,9597	✓	✓
$[H_2Cu_7Zn](Cp^*)_4$	1050,8907	✓	✓
$[Cu_7](Cp^*)_2(Mes)_3$	1071,9873	✓	✓
$[Cu_9](Cp^*)_2(Mes)_2$	1080,7580	✓	✓
$[Cu_7](Cp^*)_3(Mes)_2$	1088,0089	✓	✓
$[Cu_9](Cp^*)_3(Mes)$	1096,7881	✓	✓
$[HCu_5Zn_2](Cp^*)_4(Mes)$	1108,0456	✓	✓
$[HCu_8Zn](Cp^*)_4$	1114,8248	✓	✓
$[Cu_3Zn_4](Cp^*)_5$ (**B**)	1128,0719	✓	✓
$[Cu_7Zn](Cp^*)_3(Mes)_2$	1153,9412	✓	✓
$[Cu_7Zn](Cp^*)_4(Mes)$	1169,9734	×	✓
$[HCu_9]Cp^*(Mes)_4$	1184,8193	✓	✓
$[Cu_7](Cp^*)_2(Mes)_4$	1191,0795	✓	✓
$[Cu_9](Cp^*)_3(Mes)_2$	1215,8734	✓	✓

$[Cu_9Zn_2](Cp^*)_3(Mes)$	1226,6638	✓	✓
$[Cu_7Zn_2](Cp^*)_4(Mes)$	1233,9060	×	✓
$[Cu_7Zn](Cp^*)_2(Mes)_4$	1257,0006	✓	✓
$[Cu_7Zn_2](Cp^*)_3(Mes)_3$	1336,9574	✓	✓
$[Cu_9Zn](Cp^*)_3(Mes)_3$	1400,8855	✓	✓
$[Cu_7Zn_2](Cp^*)_2(Mes)_5$	1442,0074	✓	✓
$[Cu_{10}Zn](Cp^*)_3(Mes)_3$	1461,8229	×	✓
$[H_3Cu_6Zn_5](Cp^*)_5(Mes)$ (**D**)	1504,8750	✓	✓
$[Cu_7Zn_3](Cp^*)_3(Mes)_4$	1521,9694	✓	✓
$[Cu_9Zn_2](Cp^*)_3(Mes)_4$	1583,9421	✓	✓
$[HCu_8Zn_3](Cp^*)_4(Mes)_3$ (**E**)	1603,9283	✓	✓
$[Cu_9Zn_3](Cp^*)_4(Mes)_3$	1665,8539	✓	✓
$[Cu_{10}Zn_2](Cp^*)_2(Mes)_6$	1751,8787	✓	✓
$[Cu_4Zn_9](Cp^*)_7$	1789,4314	✓	✓
$[Cu_{10}Zn_3](Cp^*)_3(Mes)_5$	1831,8334	✓	✓
$[Cu_4Zn_{10}](Cp^*)_7$	1852,8166	×	✓
$\{[Cu_3Zn_4](Cp^*)_6(Mes)_6$ {-H}	1975,6995	✓	✓
$[Cu_4Zn_{10}](Cp^*)_8$ (**F**)	1989,9273	✓	✓

Supplementary Table 2:List of all identified molecular ions in the Cu/Zn library {**1**} with composition and m/z, as well as indications if a species has been identified by Cp^{*Et} labelling, ^{68}Zn labelling or both.

Composition	*m/z*	*Cp^{*Et} Label*	*^{68}Zn Label*
$[CuZn_2](Cp^*)_3$ (**A**)	600,1593	✓	✓
$[Cu_4](Cp^*)(Mes)_2$	627,0009	✓	✓
$[Cu_4](Cp^*)_2(Mes)$	643,0310	×	✓
$[HCu_5](Cp^*)_2(Mes)$	706,9671	✓	✓
$[Cu_4](Cp^*)(Mes)_3$	746,0845	✓	✓
$[Cu_5](Cp^*)(Mes)_3$	809,0139	×	✓
$[Cu_5](Cp^*)_2(Mes)_2$	825,0443	✓	✓
$[Cu_5Zn](Cp^*)_3(Mes)$	907,0021	✓	✓
$[HCu_5Zn_2](Cp^*)_4(Mes)$	1108,0456	✓	✓
$[Cu_3Zn_4](Cp^*)_5$ (**B**)	1128,0719	✓	✓
$[Cu_7Zn](Cp^*)_3(Mes)_2$	1153,9412	✓	✓
$[Cu_7Zn](Cp^*)_4(Mes)$	1169,9734	×	✓
$[Cu_7Zn_2](Cp^*)_4(Mes)$	1233,9060	×	✓
$[Cu_7Zn](Cp^*)_2(Mes)_4$	1257,0006	✓	✓
$[Cu_9Zn](Cp^*)_3(Mes)_3$	1400,8855	✓	✓
$[Cu_{10}Zn](Cp^*)_3(Mes)_3$	1461,8229	×	✓
$[H_3Cu_6Zn_5](Cp^*)_5(Mes)$ (**D**)	1504,8750	✓	✓
$[Cu_7Zn_3](Cp^*)_3(Mes)_4$	1521,9694	✓	✓
$[Cu_9Zn_2](Cp^*)_3(Mes)_4$	1583,9421	✓	✓
$[HCu_8Zn_3](Cp^*)_4(Mes)_3$ (**E**)	1603,9283	✓	✓
$[Cu_9Zn_3](Cp^*)_4(Mes)_3$	1665,8539	✓	✓
$[Cu_{10}Zn_2](Cp^*)_2(Mes)_6$ (**C**)	1751,8787	✓	✓
$[Cu_4Zn_{10}](Cp^*)_8$ (**F**)	1989,9273	✓	✓

Supplementary Table 3: List of all identified fragments in the Cu/Zn library {**1**} with composition and m/z, as well as indications if a species has been identified by Cp*^{Et} labelling, ^{68}Zn labelling or both.

Composition	*m/z*	*Cp*^{Et} Label*	*^{68}Zn Label*
$[CuZn](Cp^*)_2$	399,0865	✓	✓
$[Cu_3](Cp^*)_2$	461,0168	✓	✓
$[HCu_4](Cp^*)_2$	524,9531	✓	✓
$[HCu_3Zn_2](Cp^*)_3$ {-2H}	724,9912	✓	✓
$[HCu_5Zn](Cp^*)_3$	788,924	✓	✓
$[Cu_4Zn_2](Cp^*)_3$	788,924	✓	✓
$[HCu_7](Cp^*)_3$	850,8526	×	✓
$[Cu_3Zn_3](Cp^*)_4$	927,0291	✓	✓
$[Cu_5Zn_2](Cp^*)_4$	988,9597	✓	✓
$[H_2Cu_7Zn](Cp^*)_4$	1050,8907	✓	✓
$[HCu_8Zn](Cp^*)_4$	1114,8248	✓	✓
$[Cu_4Zn_9](Cp^*)_7$	1789,4314	✓	✓

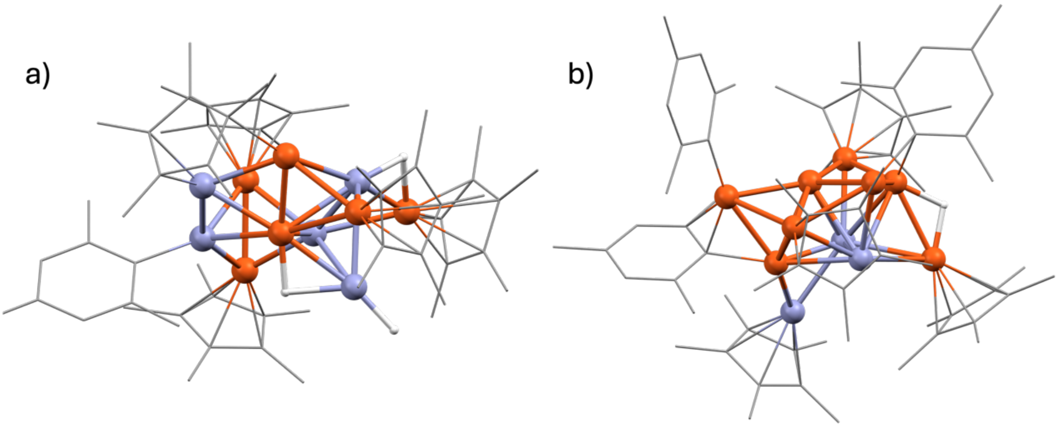

Supplementary Figure 11: Calculated structures of a) $[H_3Cu_6Zn_5](Cp^*)_5(Mes)$ (**D**) and b) $[HCu_8Zn_3](Cp^*)_4(Mes)_3$ (**E**). Color code: orange = copper, blue = zinc, grey = carbon and white = hydrogen.

Supplementary Table 4: List of all identified molecular ions in the Cu/Zn library {**2**} with composition and m/z, species of the substrate library {**1**} reacting off, as well as indications if a new species (marked in bold) has been identified by ^{68}Zn labelling, $^{13}CO_2$ labelling or both.

Composition	*m/z*	*Presence in {1}*	*^{68}Zn Label*	*$^{13}CO_2$ Label*
$[CuZn_2](Cp^*)_3$ (**A**)	600,1593	✓		
$[Cu_4](Cp^*)(Mes)_2$	627,0009	✓		
$[Cu_4](Cp^*)_2(Mes)$	643,0310	✓		
$[HCu_5](Cp^*)_2(Mes)$	706,9671	✓		
$[Cu_4](Cp^*)(Mes)_3$	746,0845	✓		
$[Cu_5](Cp^*)(Mes)_3$	809,0139	✓		
$[Cu_5](Cp^*)_2(Mes)_2$	825,0443	✓		
$[Cu_5Zn](Cp^*)_3(Mes)$	907,0021	✓		

$[HCu_5Zn_2](Cp^*)_4(Mes)$	1108,0456	✓		
$[Cu_3Zn_4](Cp^*)_5$ (**B**)	1128,0719	✓		
$[Cu_7Zn](Cp^*)_3(Mes)_2$	1153,9412	✓		
$[Cu_7Zn](Cp^*)_4(Mes)$	1169,9734	✓		
$[Cu_7Zn_2](Cp^*)_4(Mes)$	1233,9060	✓		
$[Cu_7Zn](Cp^*)_2(Mes)_4$	1257,0006	✓		
$[Cu_9Zn](Cp^*)_3(Mes)_3$	1400,8855	✓		
$[Cu_{10}Zn](Cp^*)_3(Mes)_3$	1461,8229	✓		
$[H_3Cu_6Zn_5](Cp^*)_5(Mes)$ (**D**)	1504,8750	✓		
$[Cu_7Zn_3](Cp^*)_3(Mes)_4$	1521,9694	✓		
**$[Cu_5Zn_5](Cp^*)_6(CO_2)_2$ (X)**	1542,9686		✓	✓
$[Cu_9Zn_2](Cp^*)_3(Mes)_4$	1583,9421	✓		
$[HCu_8Zn_3](Cp^*)_4(Mes)_3$ (**E**)	1603,9283	✓		
**$[Cu_8Zn_3](Cp^*)_3(Mes)_4(CO_2)$ (Y)**	1630,9030		✓	✓
$[Cu_9Zn_3](Cp^*)_4(Mes)_3$	1665,8539	✓		
$[Cu_{10}Zn_2](Cp^*)_2(Mes)_6$ (**C**)	1751,8787	✓		
$[Cu_4Zn_{10}](Cp^*)_8$ (**F**)	1989,9273	✓		

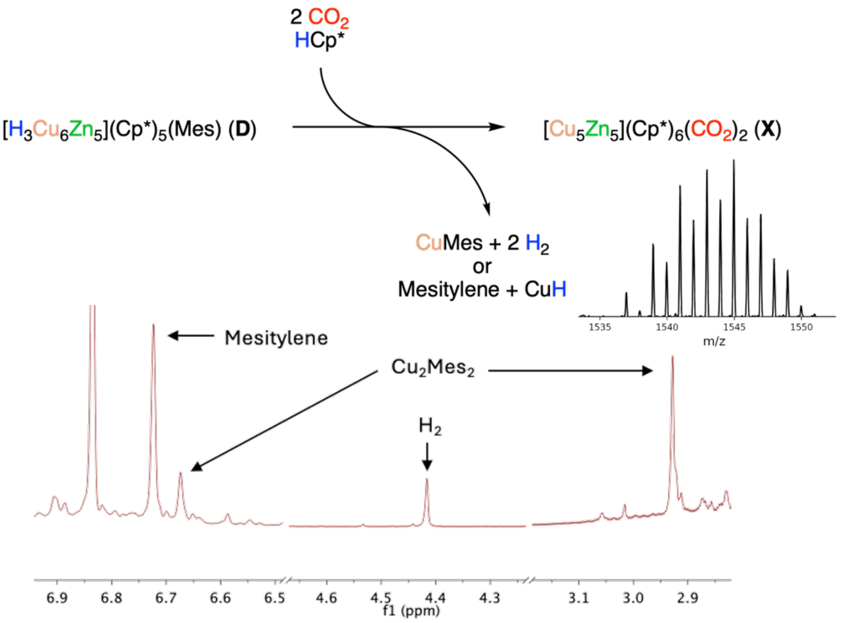

Supplementary Figure 12: Reaction scheme for the formation of species **X** from **D** in the conversion of {**1**} to {**2**} upon addition of CO_2. The side products are identified by ^{1}H-NMR spectroscopy and the pattern of the molecular ion of **X** is shown (m/z = 1542,9686; see also Supplementary Table 4).

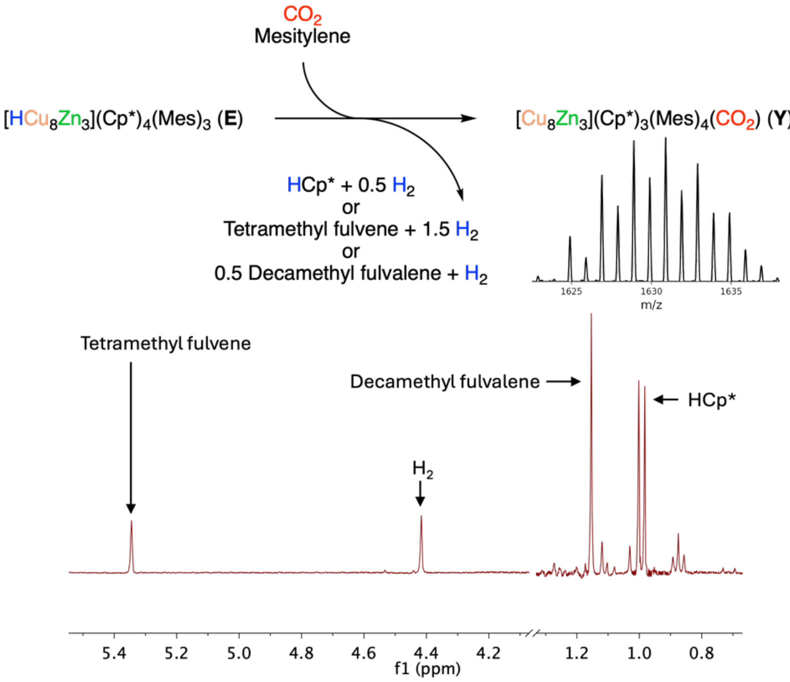

Supplementary Figure 13: Reaction scheme for the formation of species **Y** from **E** in the conversion of {**1**} to {**2**} upon addition of CO_2. The side products are identified by ^{1}H-NMR spectroscopy and the patter of the molecular ion of **Y** is shown (m/z = 1630,9030; see also Supplementary Table 4).

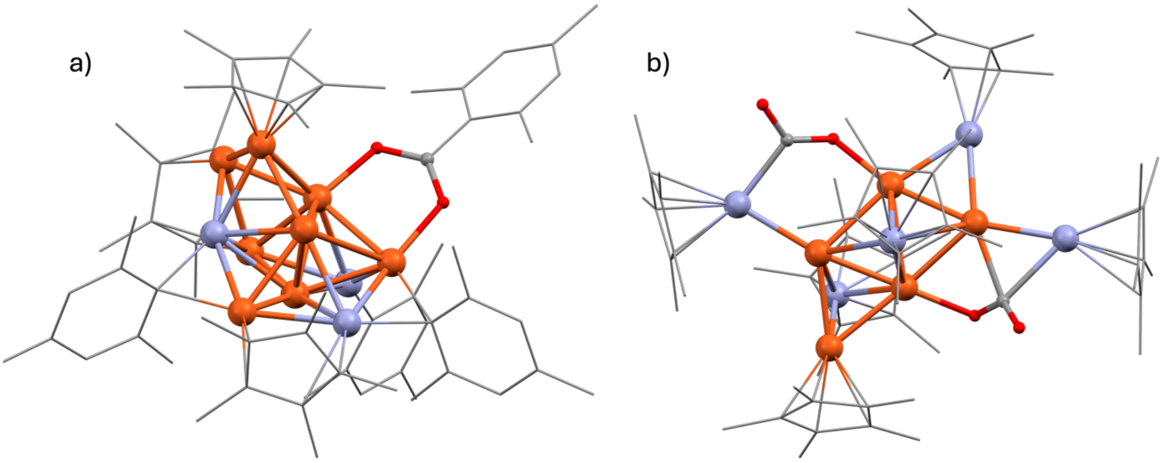

Supplementary Figure 14: Calculated structures of a) $[Cu_8Zn_3](Cp^*)_3(Mes)_4(CO_2)$ (**Y**) and b) $[Cu_5Zn_5](Cp^*)_6(CO_2)_2$ (**X**). Color code: orange = copper, blue = zinc, grey = carbon, red = oxygen and white = hydrogen.

Supplementary Table 5: List of all identified molecular ions in the Cu/Zn library {**3**} with composition and m/z, species of the substrate library {**2**} reacting off, as well as indications if a new species (marked in bold) has been identified by ^{68}Zn labelling, $^{13}CO_2$ labelling or both.

Composition	*m/z*	*Presence in {1} or {2}*	*^{68}Zn Label*	*$^{13}CO_2$ Label*
$[CuZn_2](Cp^*)_3$ (**A**)	600,1593	✓		
$[Cu_4](Cp^*)(Mes)_2$	627,0009	✓		
$[Cu_4](Cp^*)_2(Mes)$	643,0310	✓		
$[HCu_5](Cp^*)_2(Mes)$	706,9671	✓		
$[Cu_4](Cp^*)(Mes)_3$	746,0845	✓		
$[Cu_5](Cp^*)(Mes)_3$	809,0139	✓		
$[Cu_5](Cp^*)_2(Mes)_2$	825,0443	✓		
$[Cu_5Zn](Cp^*)_3(Mes)$	907,0021	✓		
$[HCu_5Zn_2](Cp^*)_4(Mes)$	1108,0456	✓		
$[Cu_3Zn_4](Cp^*)_5$ (**B**)	1128,0719	✓		
$[Cu_7Zn](Cp^*)_3(Mes)_2$	1153,9412	✓		
$[Cu_7Zn](Cp^*)_4(Mes)$	1169,9734	✓		
$[Cu_7Zn_2](Cp^*)_4(Mes)$	1233,9060	✓		
$[Cu_7Zn](Cp^*)_2(Mes)_4$	1257,0006	✓		
$[Cu_9Zn](Cp^*)_3(Mes)_3$	1400,8855	✓		
$[Cu_{10}Zn](Cp^*)_3(Mes)_3$	1461,8229	✓		
$[Cu_7Zn_3](Cp^*)_3(Mes)_4$	1521,9694	✓		
$[Cu_5Zn_5](Cp^*)_6(CO_2)_2$ (**X**)	1542,9686	✓		
$[Cu_9Zn_2](Cp^*)_3(Mes)_4$	1583,9421	✓		
$[Cu_8Zn_3](Cp^*)_3(Mes)_4(CO_2)$ (**Y**)	1630,9030	✓		
$[Cu_9Zn_3](Cp^*)_4(Mes)_3$	1665,8539	✓		
$[Cu_{10}Zn_2](Cp^*)_2(Mes)_6$ (**C**)	1751,8787	✓		
$[Cu_4Zn_{10}](Cp^*)_8$ (**F**)	1989,9173	✓		
**$[Cu_{11}Zn_6](Cp^*)_8(CO_2)_2(HCO_2)$ (Z)**	2305,7034		✓	✓

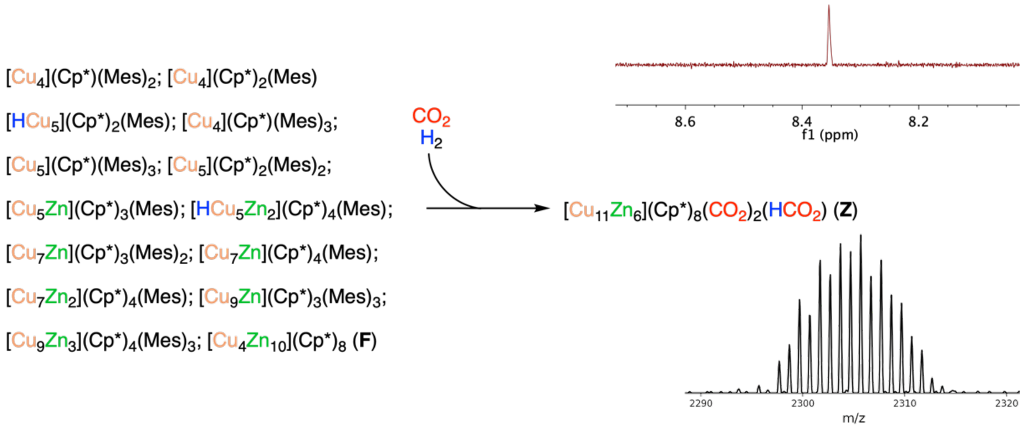

Supplementary Figure 15: Reaction scheme for the formation of species **Z** in the conversion of {**2**} to {**3**} upon addition of H_2. Formate is identified by ^{1}H-NMR spectroscopy and the patter of the molecular ion of **Z** is shown (m/z = 2305,7034; see Supplementary Table 5).

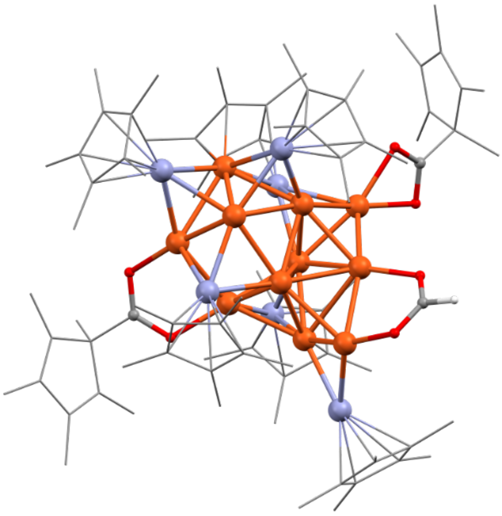

Supplementary Figure 16: Calculated structures of $[Cu_{11}Zn_6](Cp^*)_8(CO_2)_2(HCO_2)$ (**Z**). Color code: orange = copper, blue = zinc, grey = carbon, red = oxygen and white = hydrogen.

Supplementary Table 6: List of all identified molecular ions in the Cu/Zn library {**4**} with composition and m/z, species of the substrate library {**1**} reacting off, as well as indications if a new species (marked in bold) has been identified by Cp^{*Et} labelling, ^{68}Zn labelling or both.

Composition	*m/z*	*Presence in {1}*	*Cp^{*Et} Label*	*^{68}Zn Label*
$[CuZn_2](Cp^*)_3$ (**A**)	600,1593	✓		
$[Cu_4](Cp^*)(Mes)_2$	627,0009	✓		
$[HCu_5](Cp^*)_2(Mes)$	706,9671	✓		
$[HCu_5](Cp^*)_2(Mes)$	706,9671	✓		
$[Cu_4](Cp^*)(Mes)_3$	746,0845	✓		
$[Cu_5](Cp^*)(Mes)_3$	809,0139	✓		
$[Cu_5](Cp^*)_2(Mes)_2$	825,0443	✓		
$[Cu_5Zn](Cp^*)_3(Mes)$	907,0021	✓		
$[HCu_5Zn_2](Cp^*)_4(Mes)$	1108,0456	✓		
$[Cu_3Zn_4](Cp^*)_5$ (**B**)	1128,0719	✓		
$[Cu_7Zn](Cp^*)_3(Mes)_2$	1153,9412	✓		
$[Cu_7Zn](Cp^*)_4(Mes)$	1169,9734	✓		
$[Cu_7Zn_2](Cp^*)_4(Mes)$	1233,9060	✓		
$[Cu_7Zn](Cp^*)_2(Mes)_4$	1257,0006	✓		
**$[Cu_3Zn_6](Cp^*)_4(3\text{-}Hex)_2H_2$**	1288,9970		✓	✓
$[Cu_9Zn](Cp^*)_3(Mes)_3$	1400,8855	✓		
$[Cu_{10}Zn](Cp^*)_3(Mes)_3$	1461,8229	✓		
$[H_3Cu_6Zn_5](Cp^*)_5(Mes)$ (**D**)	1504,8750	✓		
$[Cu_7Zn_3](Cp^*)_3(Mes)_4$	1521,9694	✓		
$[Cu_9Zn_2](Cp^*)_3(Mes)_4$	1583,9421	✓		
$[HCu_8Zn_3](Cp^*)_4(Mes)_3$ (**E**)	1603,9283	✓		
$[Cu_9Zn_3](Cp^*)_4(Mes)_3$	1665,8539	✓		
$[Cu_{10}Zn_2](Cp^*)_2(Mes)_6$ (**C**)	1751,8787	✓		
$[Cu_4Zn_{10}](Cp^*)_8$ (**F**)	1989,9173	✓		
**$[Cu_9Zn_7](Cp^*)_6(3\text{-}Hex)_3H_3$ (W)**	2089,8176		✓	✓

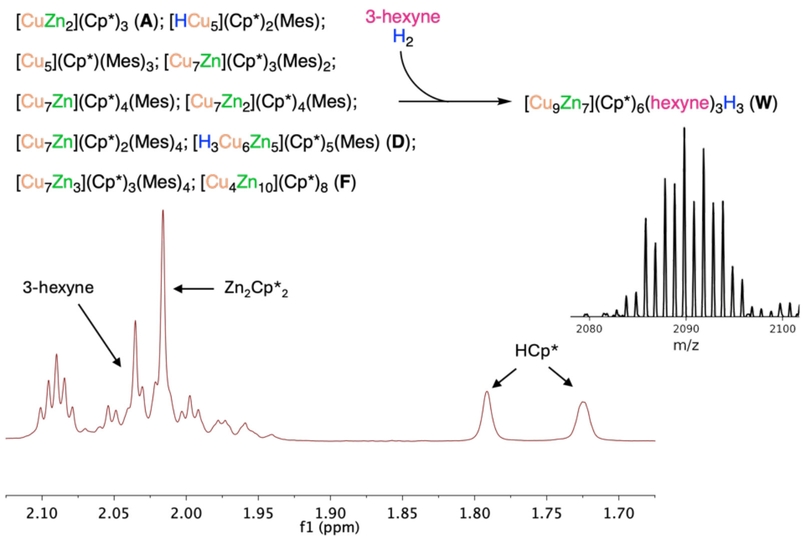

Supplementary Figure 17: Reaction scheme for the formation of species **W** in the conversion of {**1**} to {**4**} upon addition of 3-hexyne and H_2. $Zn_2Cp^*_2$ and HCp* are identified by ^{1}H-NMR spectroscopy and the pattern of the molecular ion of **W** is shown (m/z = 2089,8176; see Supplementary Table 6).

1.1.1.3. Peak Identification

Through the analysis of the *m/z* value and the isotopic pattern of each peak, one or multiple theoretical compositions could be assigned to each peak in the LIFDI mass spectrum. Below the double labelling work flow for peak pattern assignment is explained.

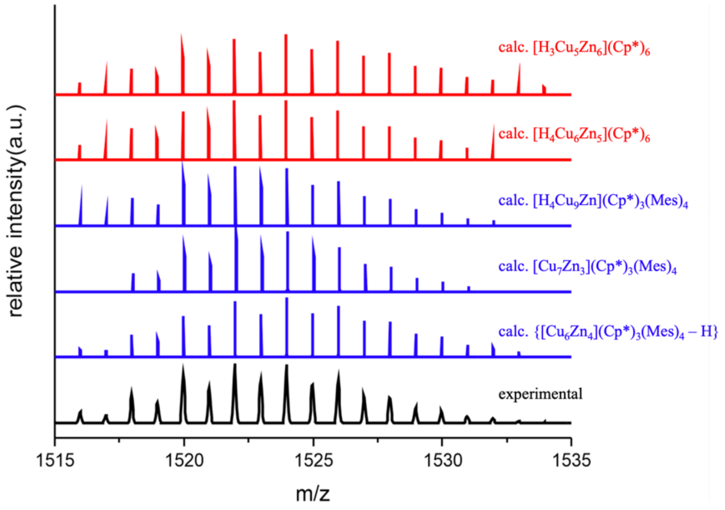

Supplementary Figure 18: For illustration, selected series of several matching isotopic patterns with similar GooF for an experimental mass spectrometric peak pattern of the Cu/Zn cluster library {**1**}. Blue = Matches with 3 Cp* ligands, red = Matches with 6 Cp* ligands.

In order to discriminate between the different possible compositions, labelling experiments with Cp*^{Et} and ^{68}Zn were performed. The procedure is illustrated in Supplementary Figure 19 and must be repeated for every peak in the spectrum. The number of matches for each peak is thereby reduced to species carrying the same number of Cp* ligands. Notably, the procedure did work for most of the peaks and corresponding, mass-shifted envelopes were identified in the Cp*^{Et} labeled spectrum.

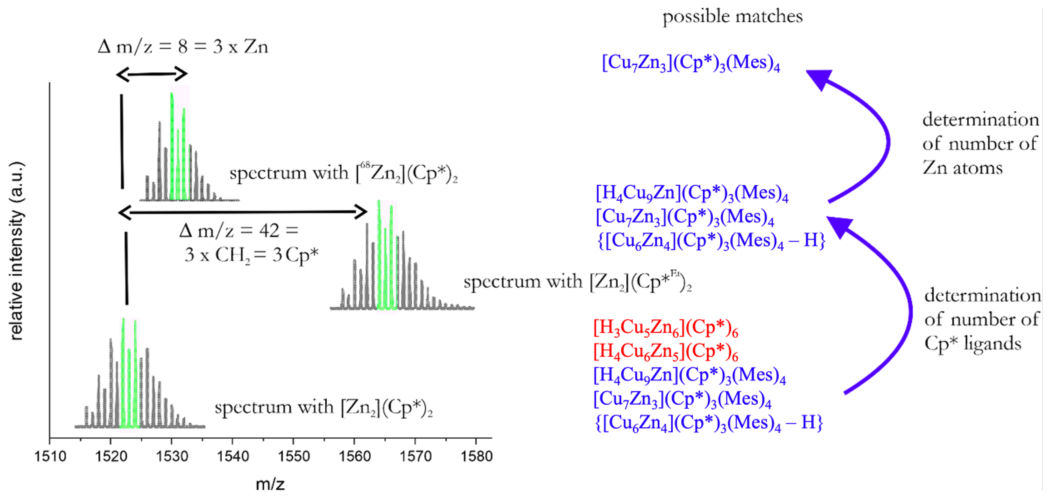

Supplementary Figure 19: Determination of the composition via Cp*^{Et} and ^{68}Zn labeling.

Using the same procedure as for the Cp^{*Et} labeling experiments, the number of Zn atoms and thereby the cluster core composition can be identified. The labeling with ^{68}Zn additionally changes (simplifies!) the isotopic patterns, leading to an easy and straightforward interpretation of most of the peaks. For many peaks, labeling with ^{68}Zn was sufficient to achieve unambiguous identification. Consequently, many of the cases, for which Cp^{*Et} labeling did not yield clear results, could eventually be resolved by using the ^{68}Zn label.

The procedure was repeated for all peaks of the spectrum. A list of more than 50 species is produced, which is shown in Supplementary Table 1. It is also denoted in the table, whether a species was identified solely by its ^{68}Zn pattern or by combination of Cp^{*Et} and ^{68}Zn labeling experiments.

This procedure is described exemplarily for the peak at *m/z* = 1521.986 (Supplementary Figures 18 and 19). The consideration of Cu, Zn, Cp*, mestiyl and hydrides leads to 5 possible combinations with simulated patterns corresponding well to the experimental one. Through the synthesis of {**1**} using $Zn_2Cp^{*Et}_2$, a Δ*m/z* of 42 is observed for this pattern. As the *m/z* difference between Cp* and Cp^{*Et} is 14 (additional CH_2 group in Cp^{*Et}), it is thus determined, that this species bears 3 Cp* ligands. This reduces the number of possible compositions to those only containing 3 Cp* ligands: $[Cu_6Zn_4]Cp^*_3Mes_4$\{-H\}, $[H_4Cu_9Zn]Cp^*_3Mes_4$ and $[Cu_7Zn_3]Cp^*_3Mes_4$. All remaining possible combinations exhibit an identical hydrocarbon ligand shell. As such, isotopic labeling of the metal atoms is required in order to obtain a definite composition for this peak. This is done using $^{68}Zn_2Cp^*_2$ for the synthesis of {**1**}, yielding a library with peaks shifted by $\Delta m/z = n_{Zn} \times 2.62$. In this example, the observed peak shift corresponds to 3 zinc atoms. As only one composition matches the information of both the Cp^{*Et} and ^{68}Zn labeling experiments, namely $[Cu_7Zn_3]Cp^*_3Mes_4$, this is then determined to be the definite sum formula for the peak at *m/z* = 1521.986.

Molecular Ions

Below we list the data of the identified cluster molecular ions present in the libraries {**1**}-{**4**}: comparison of experimental and calculated peak patterns.

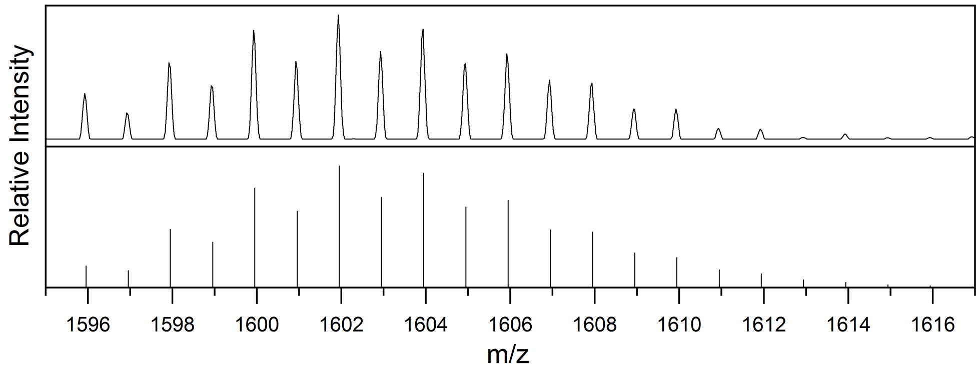

Supplementary Figure 20: Experimental pattern (top) and calculated pattern (bottom) for $[HCu_8Zn_3](Cp^*)_4(Mes)_3$ (**E**).

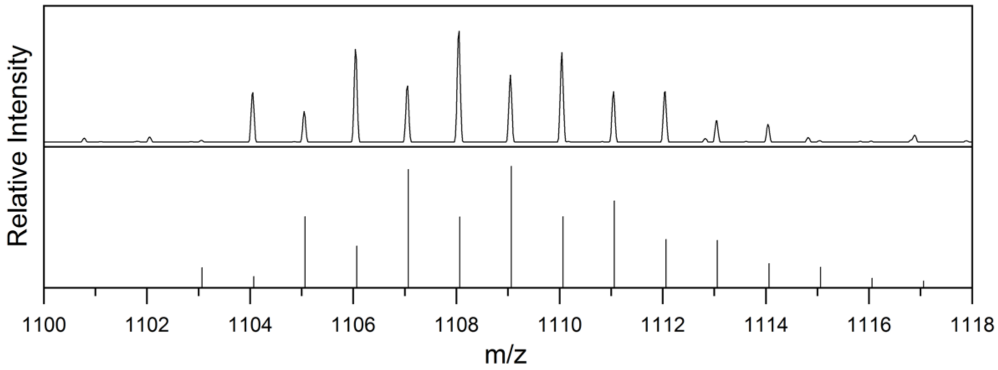

Supplementary Figure 21: Experimental pattern (top) and calculated pattern (bottom) for $[HCu_5Zn_2](Cp^*)_4(Mes)$.

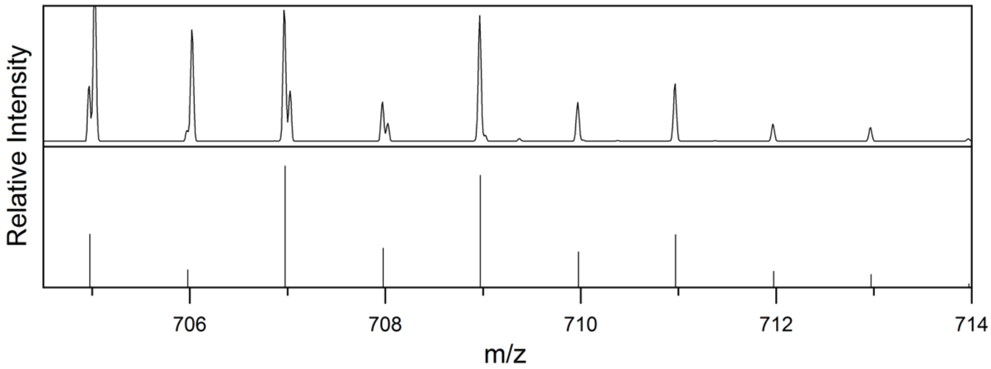

Supplementary Figure 22: Experimental pattern (top) and calculated pattern (bottom) for $[HCu_5](Cp^*)_2(Mes)$.

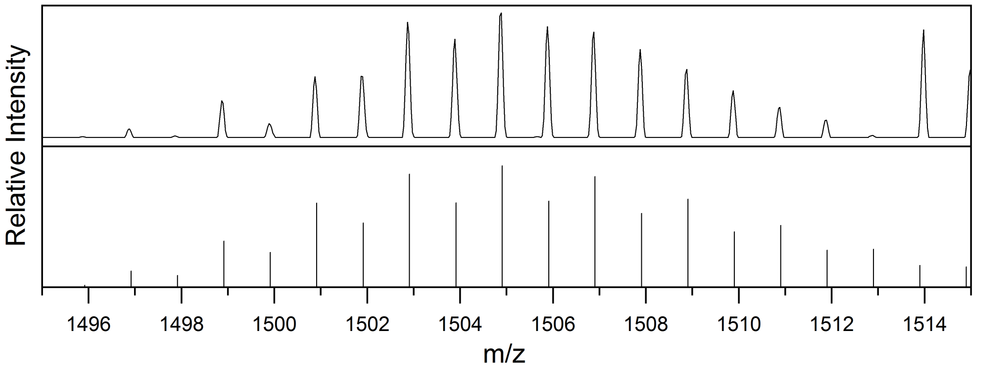

Supplementary Figure 23: Experimental pattern (top) and calculated pattern (bottom) for $[H_3Cu_6Zn_5](Cp^*)_5(Mes)_3$ (**D**).

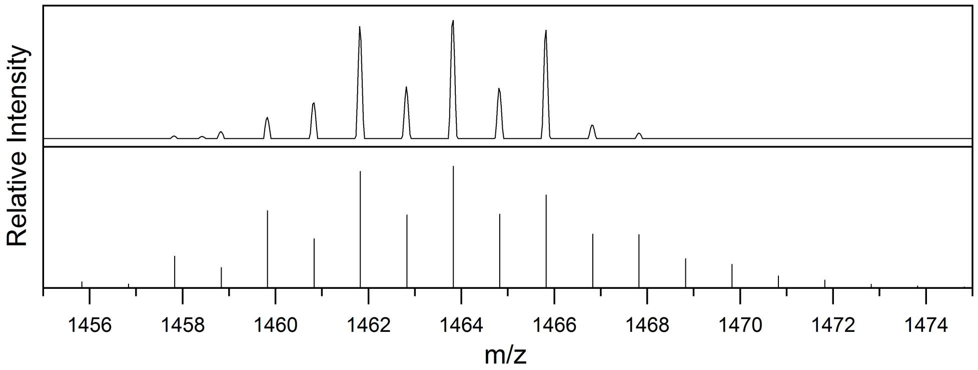

Supplementary Figure 24: Experimental pattern (top) and calculated pattern (bottom) for $[Cu_{10}Zn](Cp^*)_3(Mes)_3$.Low intensity of the pattern gives this "truncated" aspect with peaks at the extremities of the pattern missing.

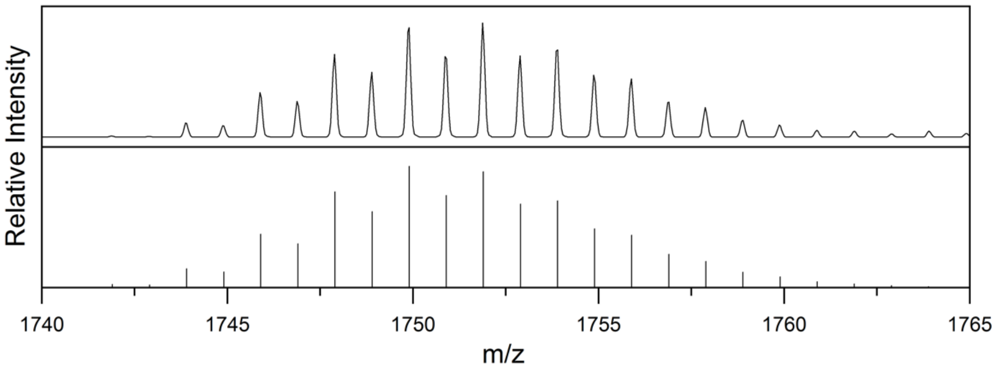

Supplementary Figure 25: Experimental pattern (top) and calculated pattern (bottom) for $[Cu_{10}Zn_2](Cp^*)_2(Mes)_6$.

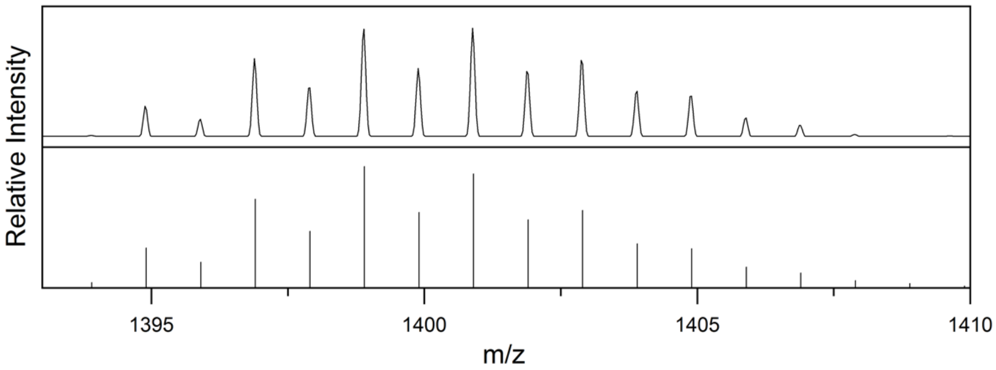

Supplementary Figure 26: Experimental pattern (top) and calculated pattern (bottom) for $[Cu_9Zn](Cp^*)_3(Mes)_3$.

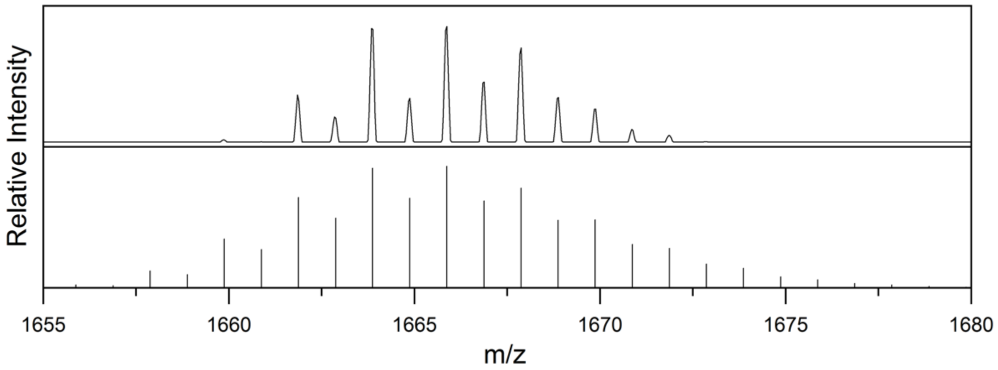

Supplementary Figure 27: Experimental pattern (top) and calculated pattern (bottom) for $[Cu_9Zn_3](Cp^*)_4(Mes)_3$. Low intensity of the pattern gives this "truncated" aspect with peaks at the extremities of the pattern missing.

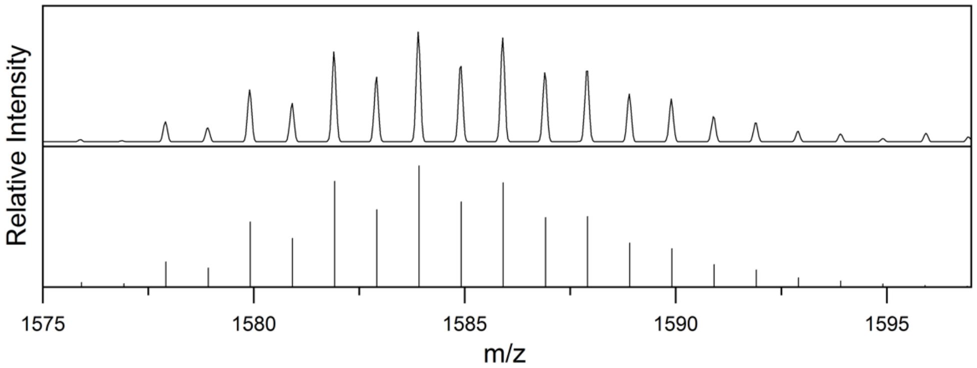

Supplementary Figure 28:Experimental pattern (top) and calculated pattern (bottom) for $[Cu_9Zn_2](Cp^*)_3(Mes)_4$.

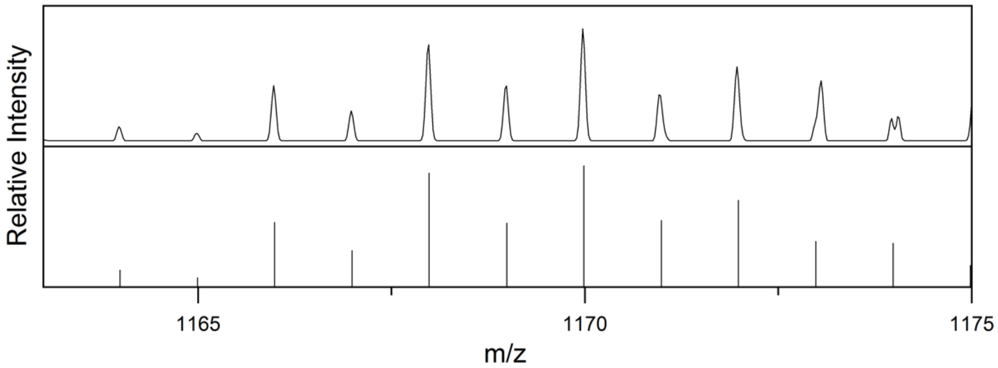

Supplementary Figure 29: Experimental pattern (top) and calculated pattern (bottom) for $[Cu_7Zn](Cp^*)_4(Mes)$.

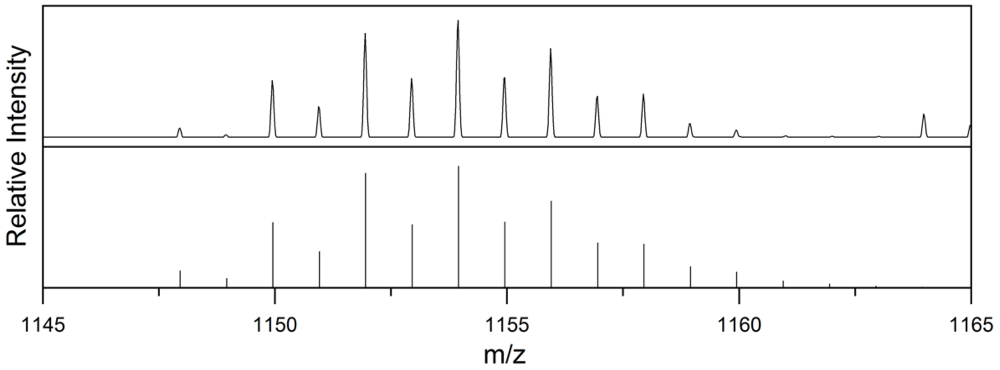

Supplementary Figure 30: Experimental pattern (top) and calculated pattern (bottom) for $[Cu_7Zn](Cp^*)_3(Mes)_2$.

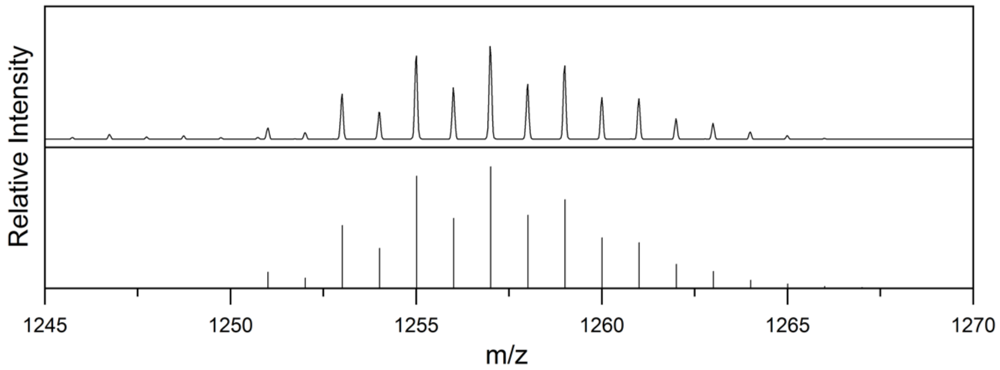

Supplementary Figure 31: Experimental pattern (top) and calculated pattern (bottom) for $[Cu_7Zn](Cp^*)_2(Mes)_4$.

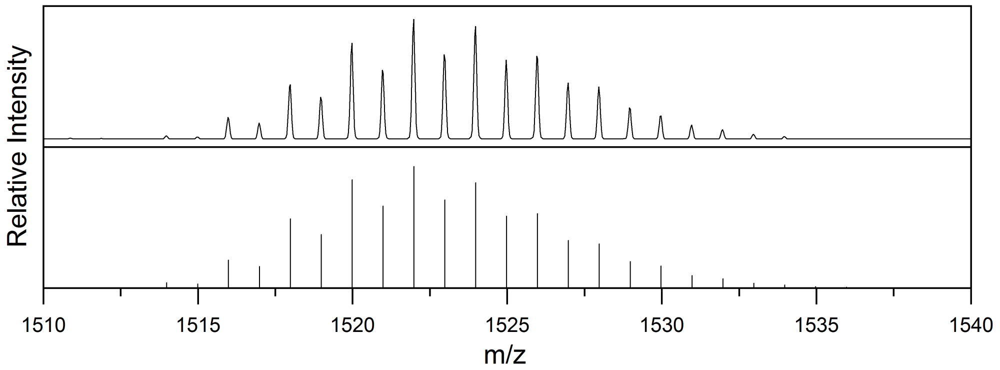

Supplementary Figure 32: Experimental pattern (top) and calculated pattern (bottom) for $[Cu_7Zn_3](Cp^*)_3(Mes)_4$.

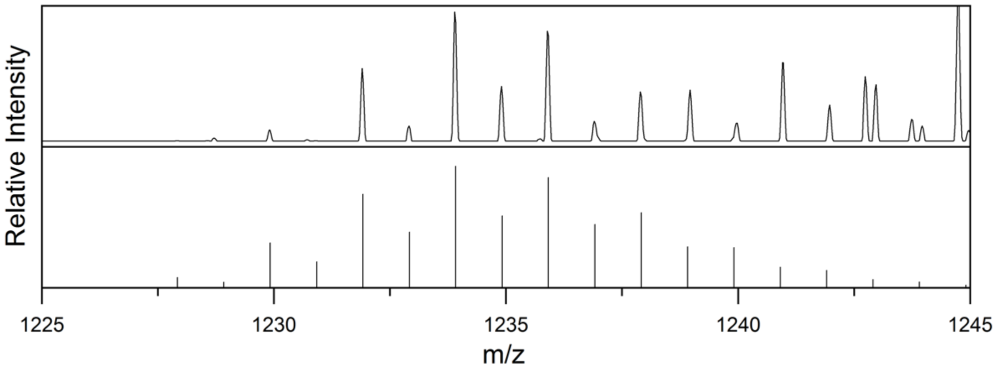

Supplementary Figure 33: Experimental pattern (top) and calculated pattern (bottom) for $[Cu_7Zn_2](Cp^*)_4(Mes)$.

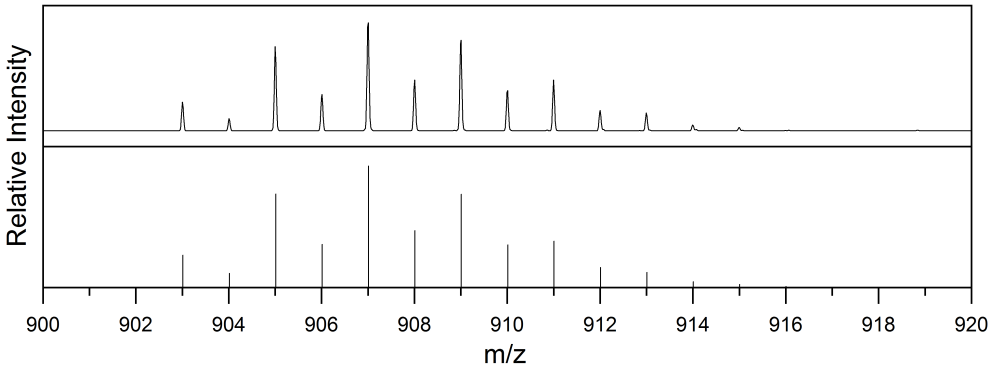

Supplementary Figure 34: Experimental pattern (top) and calculated pattern (bottom) for $[Cu_5Zn](Cp^*)_3(Mes)$.

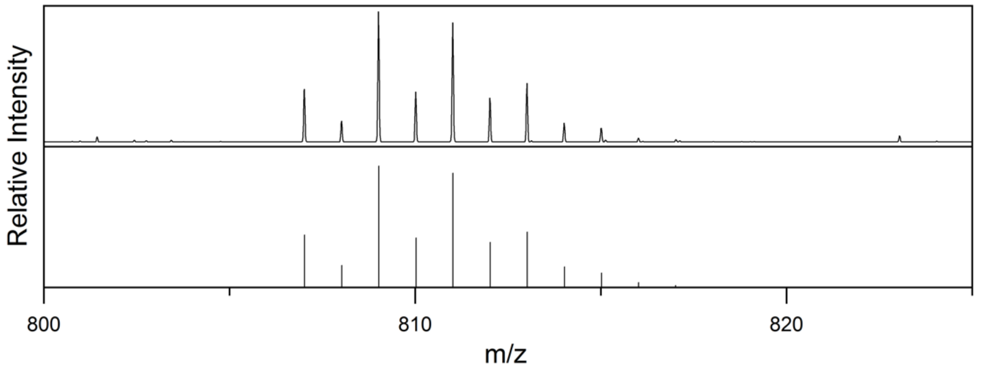

Supplementary Figure 35: Experimental pattern (top) and calculated pattern (bottom) for $[Cu_5](Cp^*)(Mes)_3$.

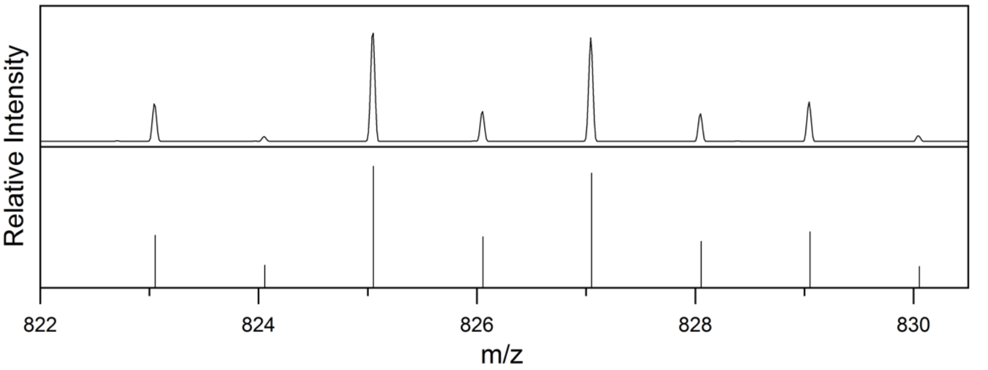

Supplementary Figure 36: Experimental pattern (top) and calculated pattern (bottom) for $[Cu_5](Cp^*)_2(Mes)_2$.

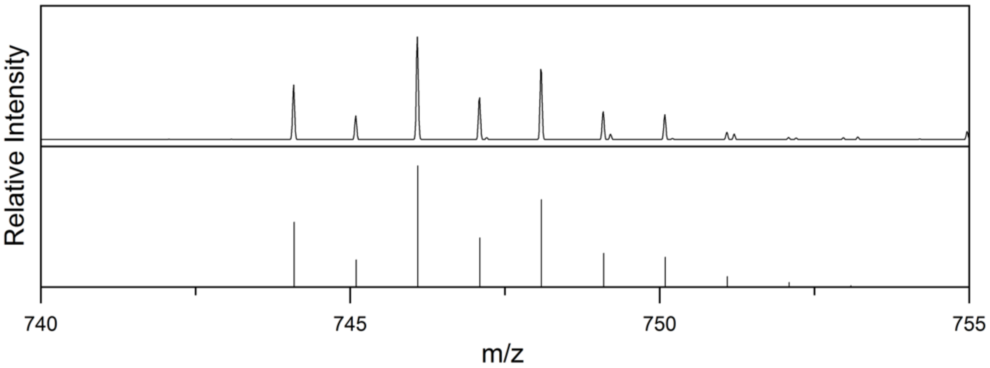

Supplementary Figure 37: Experimental pattern (top) and calculated pattern (bottom) for $[Cu_4](Cp^*)(Mes)_3$.

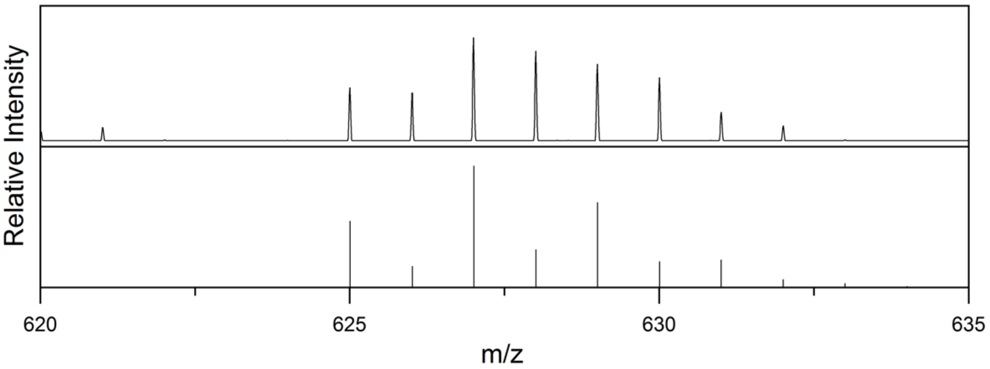

Supplementary Figure 38: Experimental pattern (top) and calculated pattern (bottom) for $[Cu_4](Cp^*)(Mes)_2$.

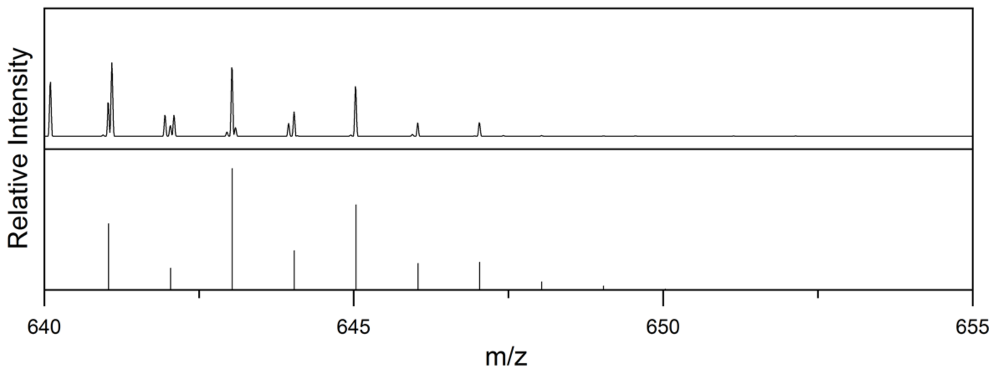

Supplementary Figure 39: Experimental pattern (top) and calculated pattern (bottom) for $[Cu_4](Cp^*)_2(Mes)$.

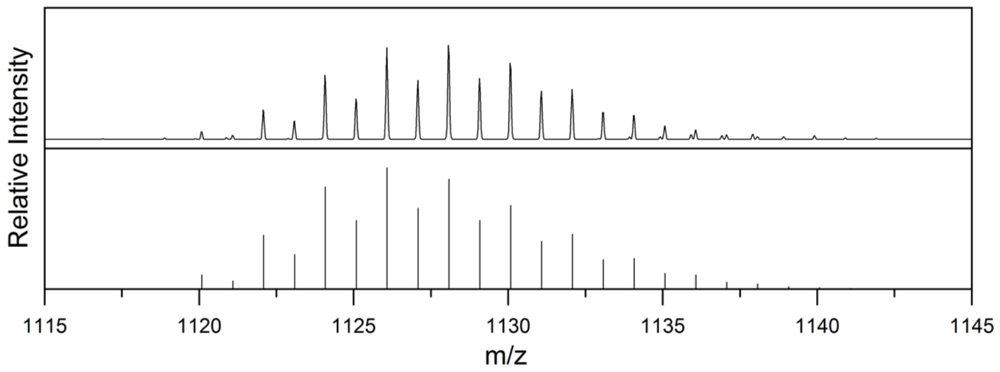

Supplementary Figure 40: Experimental pattern (top) and calculated pattern (bottom) for $[Cu_3Zn_4](Cp^*)_5$ (**B**).

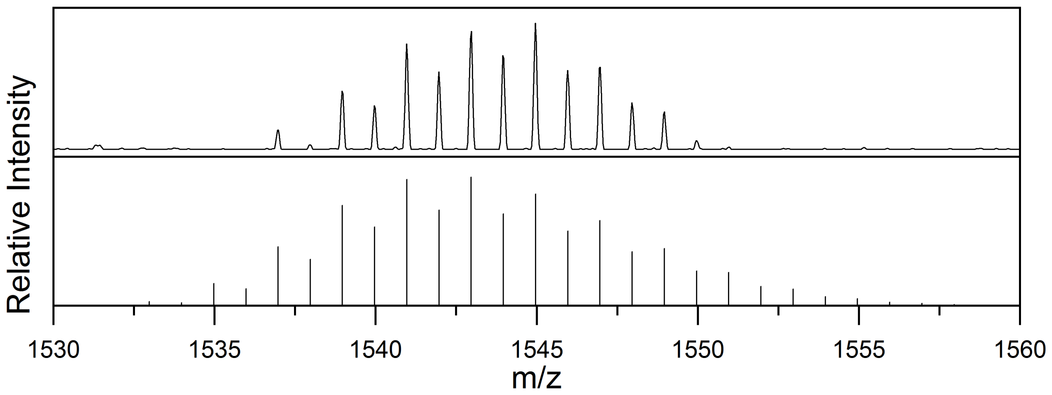

Supplementary Figure 41: Experimental pattern (top) and calculated pattern (bottom) for $[Cu_5Zn_5](Cp^*)_6(CO_2)_2$ (**X**).

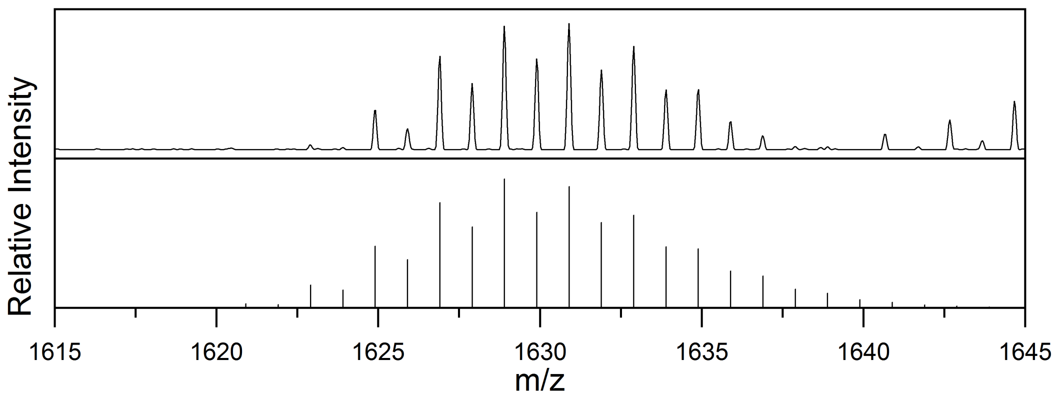

Supplementary Figure 42: Experimental pattern (top) and calculated pattern (bottom) for $[Cu_8Zn_3](Cp^*)_3(Mes)_4(CO_2)$ (**Y**).

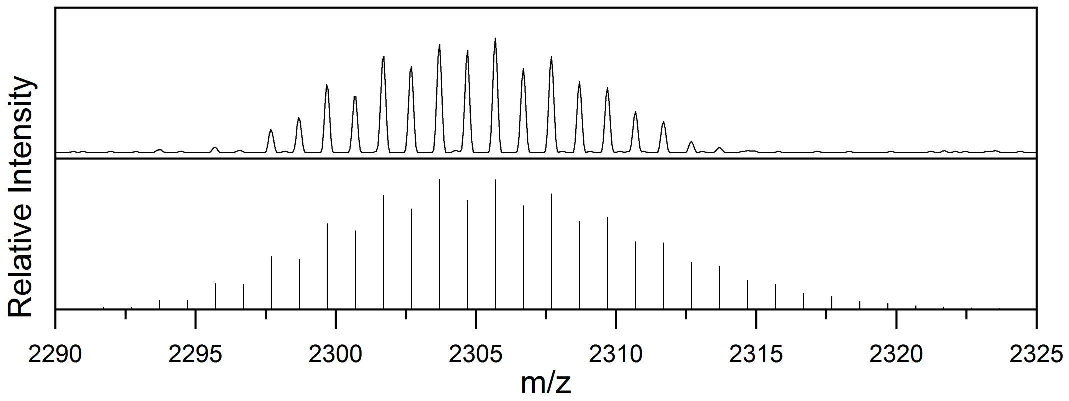

Supplementary Figure 43: Experimental pattern (top) and calculated pattern (bottom) for $[Cu_{11}Zn_6](Cp^*)_8(CO_2)_2(HCO_2)$ (**Z**).

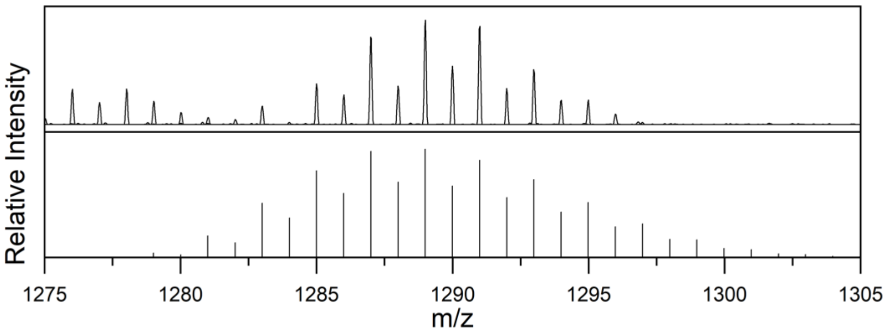

Supplementary Figure 44: Experimental pattern (top) and calculated pattern (bottom) for $[Cu_3Zn_6](Cp^*)_4(Hex)_2H_2$.

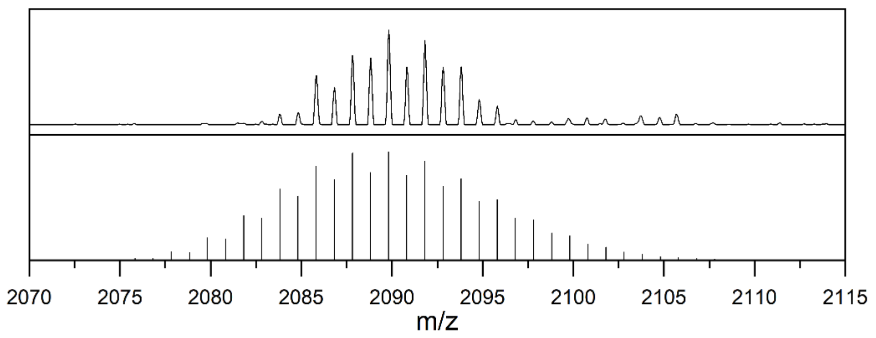

Supplementary Figure 45: Experimental pattern (top) and calculated pattern (bottom) for $[Cu_9Zn_7](Cp^*)_6(Hex)_3H_3$ (**W**).

Fragments

Below we list the data of the identified cluster fragment ions of clusters present in the libraries {**1**}-{**4**}: comparison of experimental and calculated peak patterns.

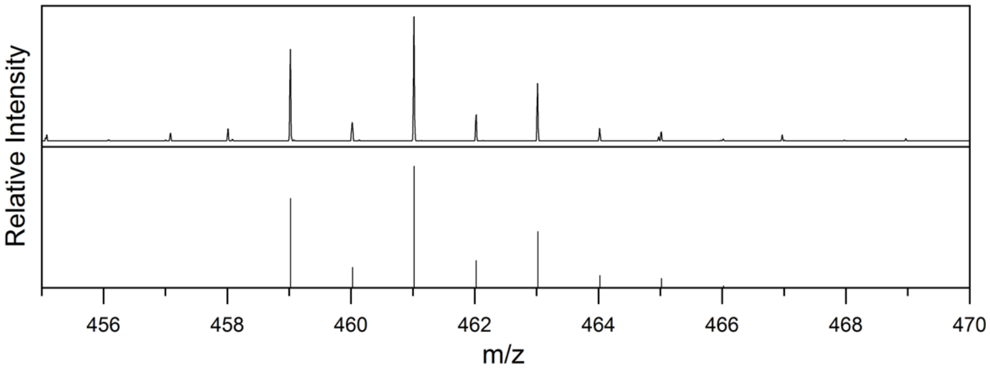

Supplementary Figure 46: Experimental pattern (top) and calculated pattern (bottom) for $[Cu_3](Cp^*)_2$.

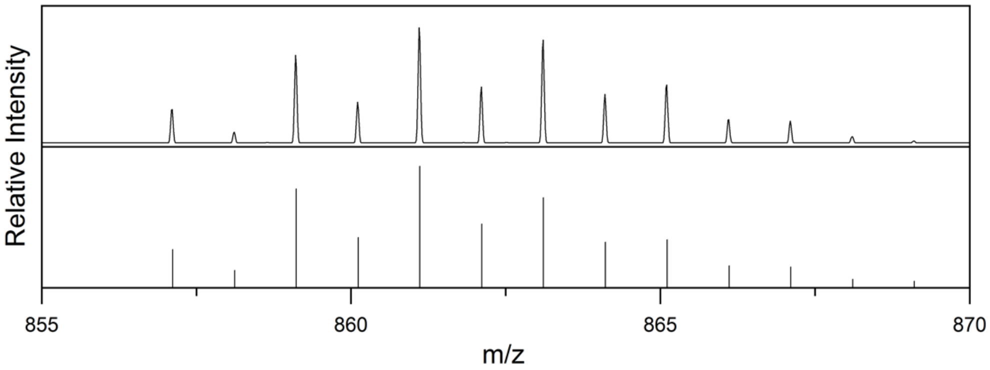

Supplementary Figure 47: Experimental pattern (top) and calculated pattern (bottom) for $[Cu_3Zn_2](Cp^*)_4$.

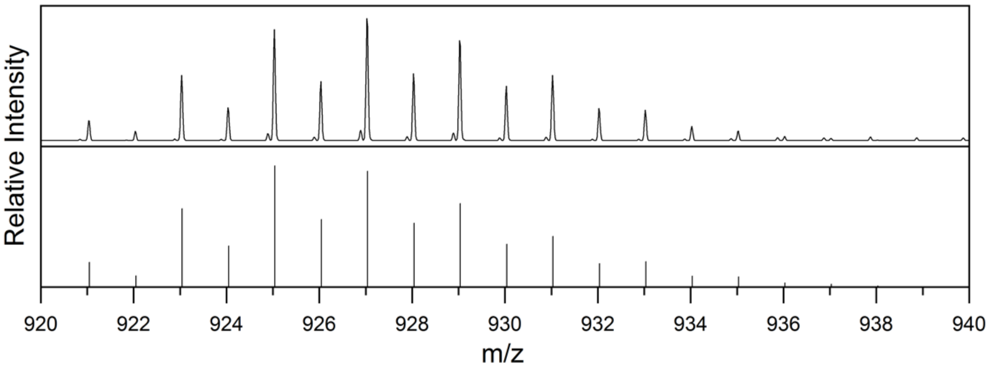

Supplementary Figure 48: Experimental pattern (top) and calculated pattern (bottom) for $[Cu_3Zn_3](Cp^*)_4$.

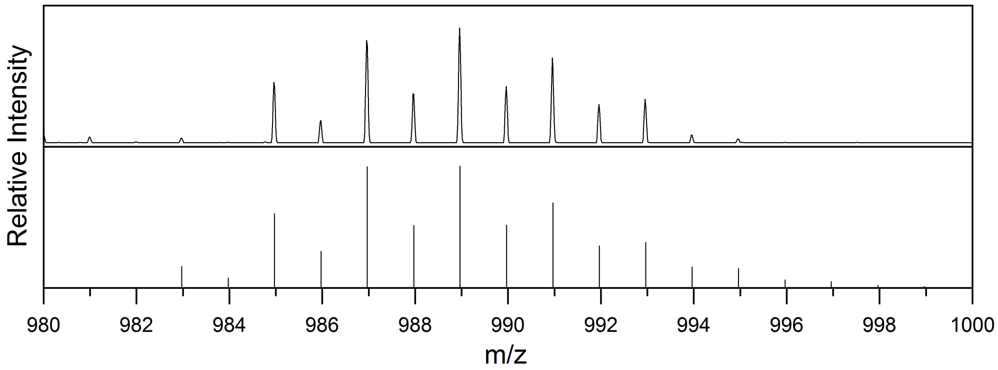

Supplementary Figure 49: Experimental pattern (top) and calculated pattern (bottom) for $[Cu_5Zn_2](Cp^*)_4$.

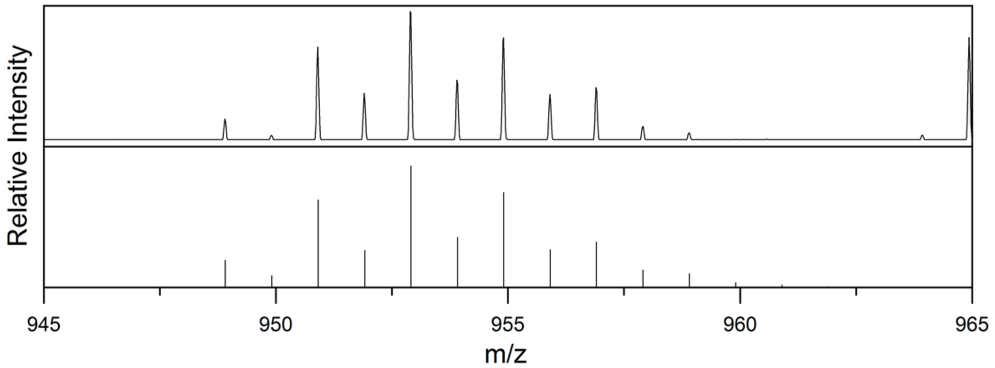

Supplementary Figure 50: Experimental pattern (top) and calculated pattern (bottom) for $[Cu_7](Cp^*)_2(Mes)_2$.

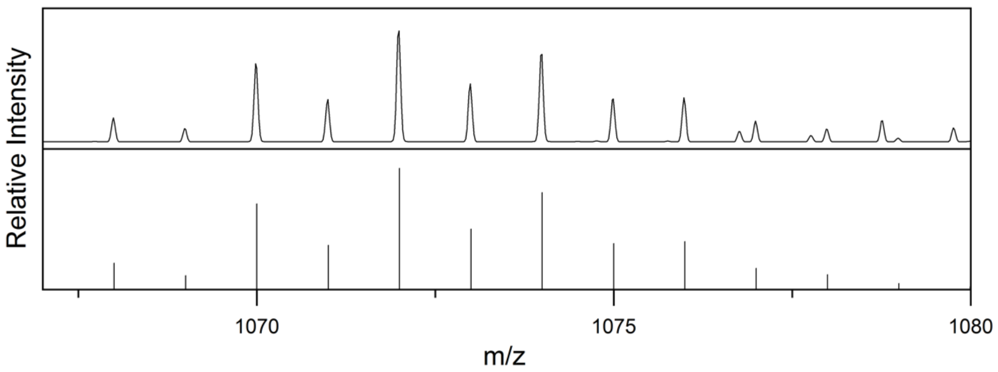

Supplementary Figure 51: Experimental pattern (top) and calculated pattern (bottom) for $[Cu_7](Cp^*)_2(Mes)_3$.

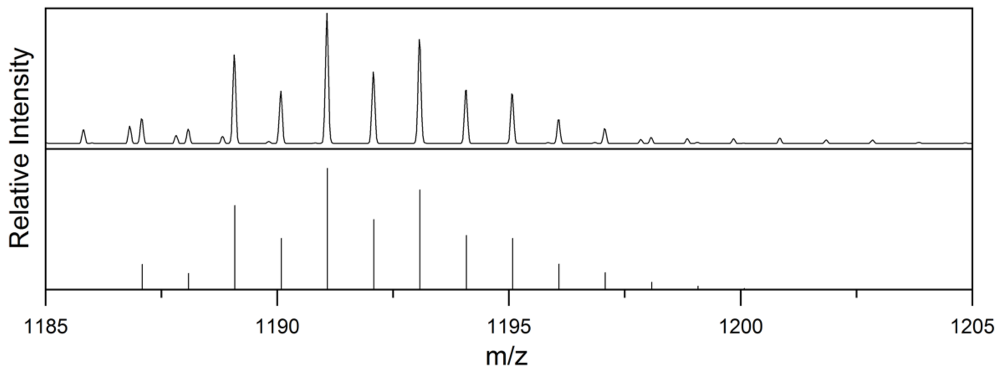

Supplementary Figure 52: Experimental pattern (top) and calculated pattern (bottom) for $[Cu_7](Cp^*)_2(Mes)_4$.

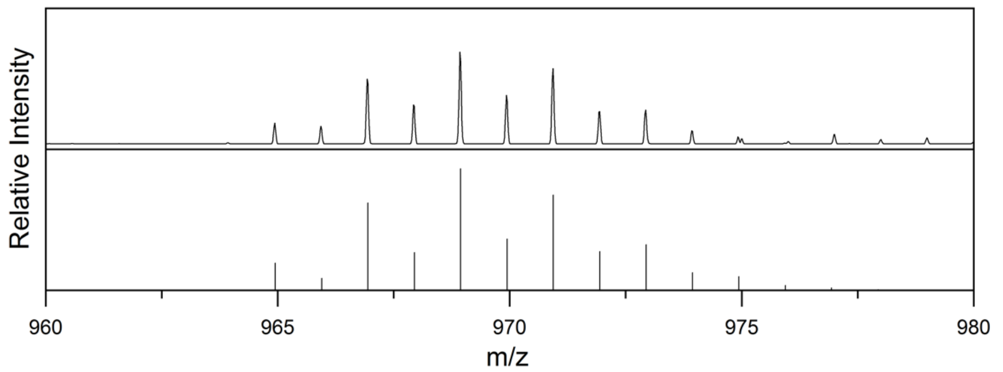

Supplementary Figure 53: Experimental pattern (top) and calculated pattern (bottom) for $[Cu_7](Cp^*)_3(Mes)$.

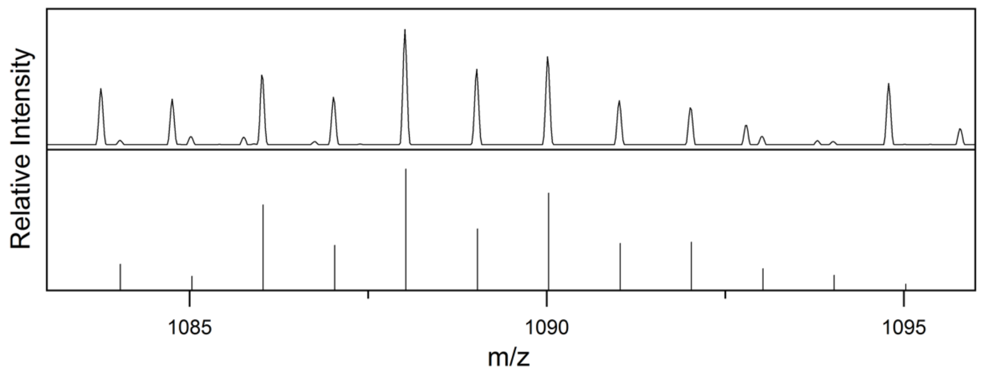

Supplementary Figure 54: Experimental pattern (top) and calculated pattern (bottom) for $[Cu_7](Cp^*)_3(Mes)_2$.

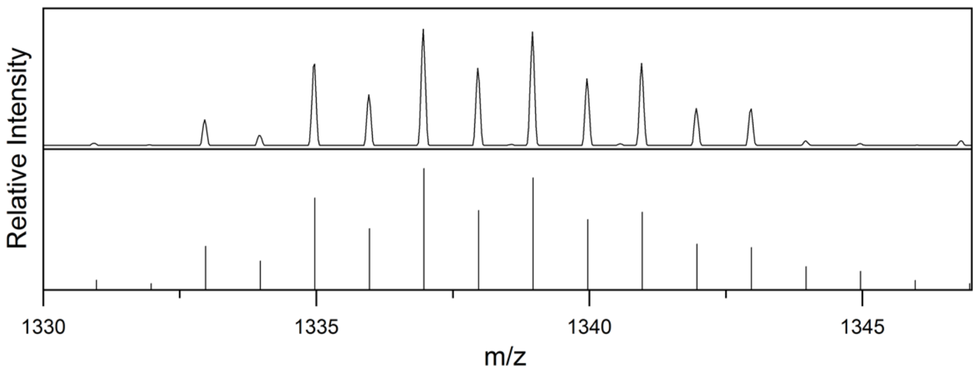

Supplementary Figure 55: Experimental pattern (top) and calculated pattern (bottom) for $[Cu_7Zn_2](Cp^*)_3(Mes)_3$.

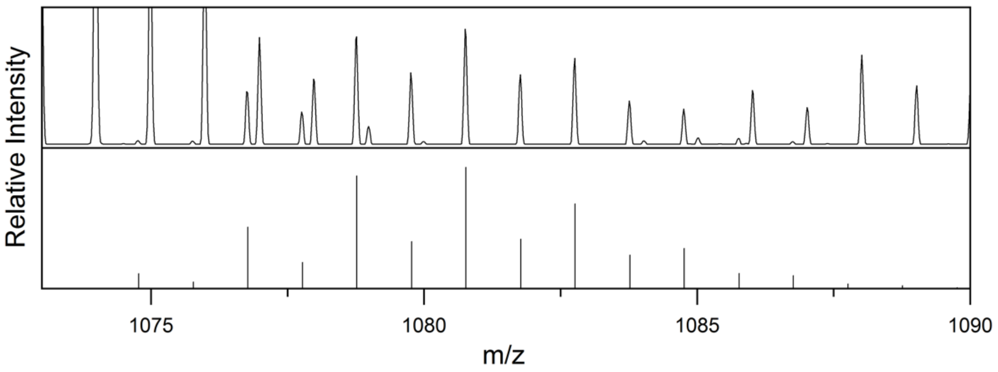

Supplementary Figure 56: Experimental pattern (top) and calculated pattern (bottom) for $[Cu_9](Cp^*)_2(Mes)_2$. Low signal intensity and overlap with $[Cu_7](Cp^*)_2(Mes)_3$ makes the pattern difficult to distinguish.

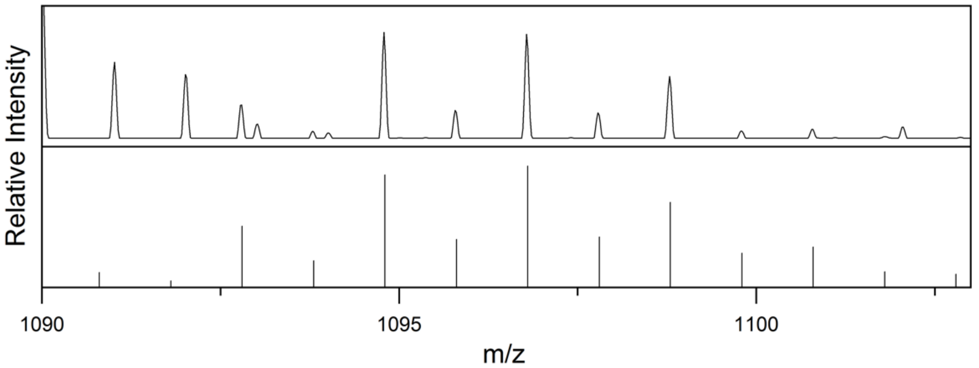

Supplementary Figure 57: Experimental pattern (top) and calculated pattern (bottom) for $[Cu_9](Cp^*)_3(Mes)$. Low signal intensity makes the pattern difficult to distinguish.

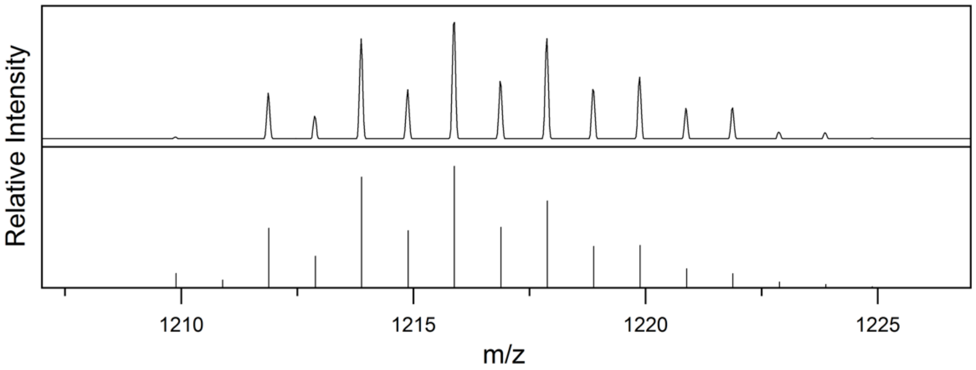

Supplementary Figure 58: Experimental pattern (top) and calculated pattern (bottom) for $[Cu_9](Cp^*)_3(Mes)_2$.

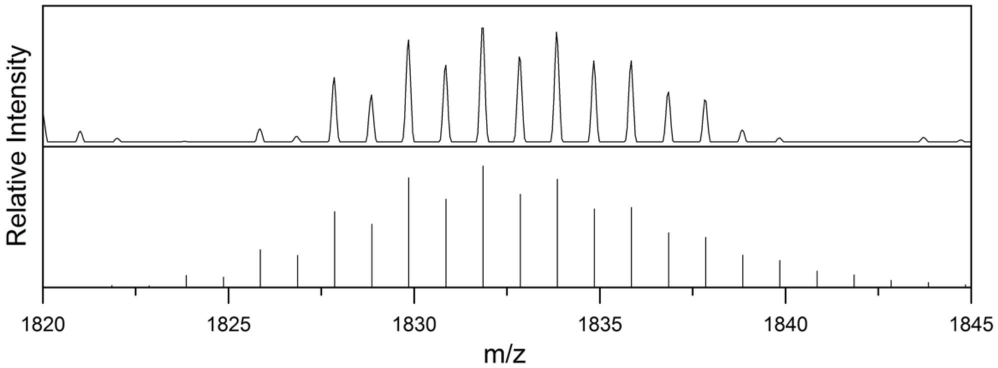

Supplementary Figure 59: Experimental pattern (top) and calculated pattern (bottom) for $[Cu_{10}Zn_3](Cp^*)_3(Mes)_5$.

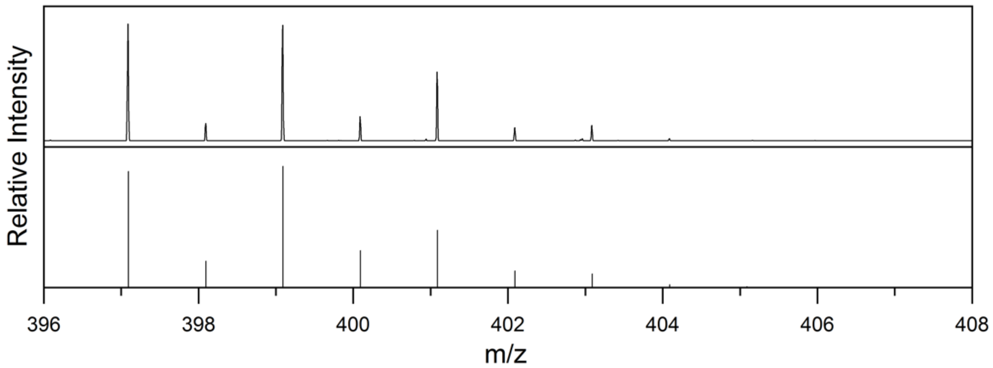

Supplementary Figure 60: Experimental pattern (top) and calculated pattern (bottom) for $[CuZn](Cp^*)_2$.

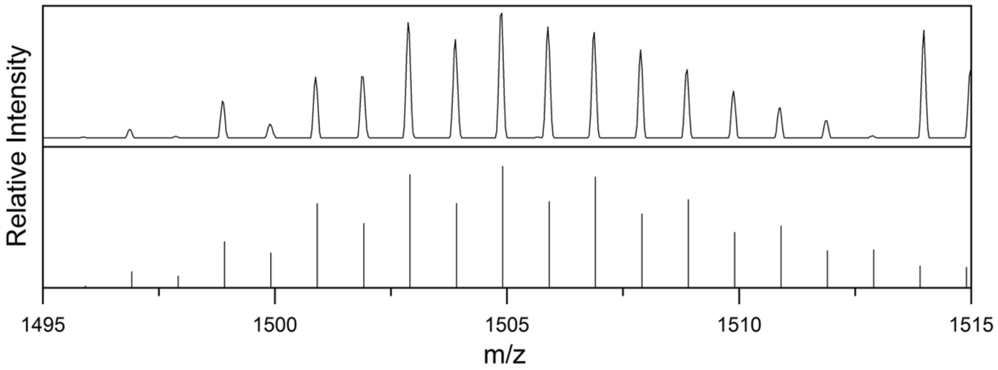

Supplementary Figure 61: Experimental pattern (top) and calculated pattern (bottom) for $[H_3Cu_6Zn_5](Cp^*)_5(Mes)$ (**D**).

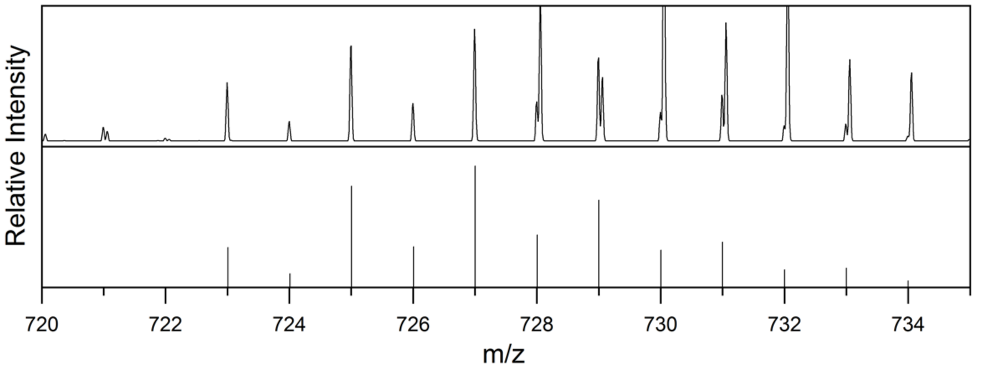

Supplementary Figure 62: Experimental pattern (top) and calculated pattern (bottom) for $[HCu_3Zn_2](Cp^*)_3$. Low signal intensity and overlap with $[Cu_3Zn](Mes)_4$ makes the pattern difficult to distinguish.

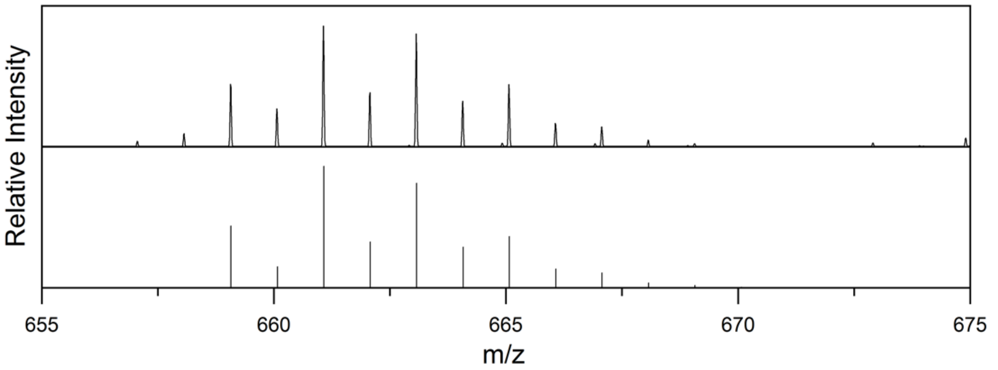

Supplementary Figure 63: Experimental pattern (top) and calculated pattern (bottom) for $[HCu_3Zn](Cp^*)_3$.

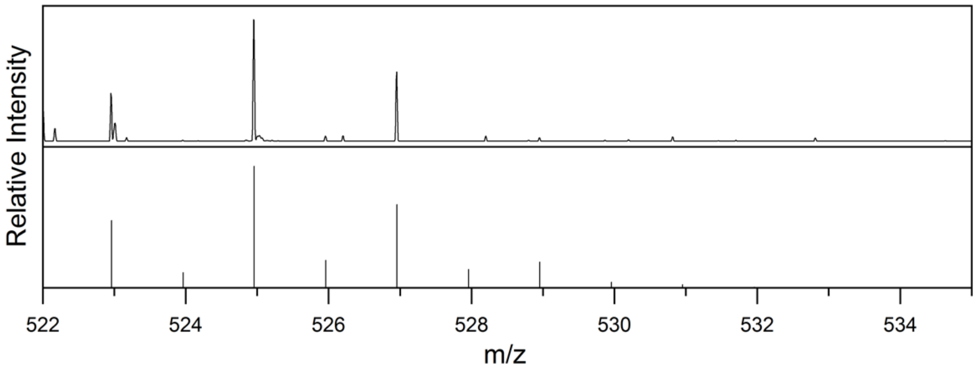

Supplementary Figure 64: Experimental pattern (top) and calculated pattern (bottom) for $[HCu_4](Cp^*)_2$. Low signal intensity makes the pattern difficult to distinguish.

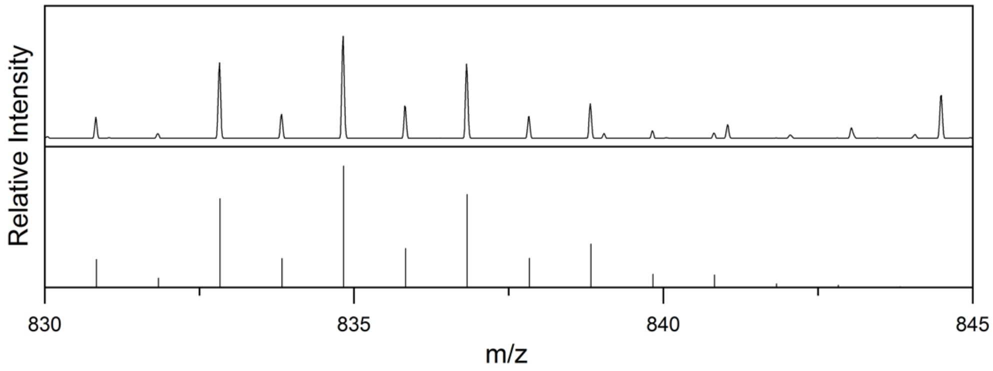

Supplementary Figure 65: Experimental pattern (top) and calculated pattern (bottom) for $[HCu_7](Cp^*)_2(Mes)$.

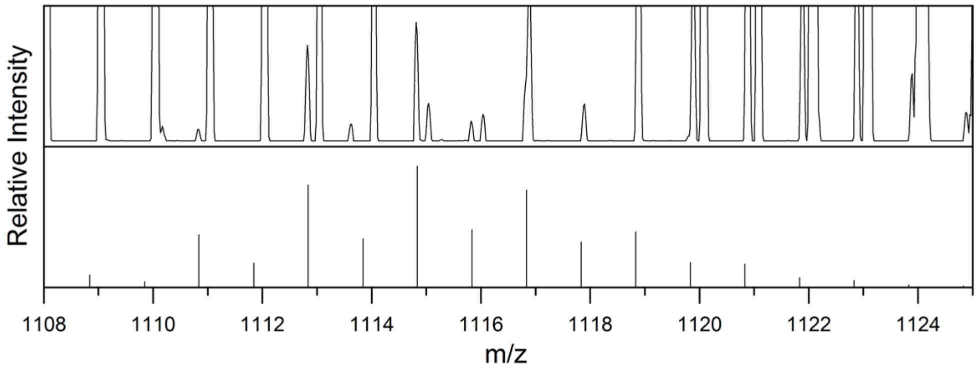

Supplementary Figure 66: Experimental pattern (top) and calculated pattern (bottom) for $[HCu_8Zn](Cp^*)_4$. Low signal intensity and overlap with $[HCu_5Zn_2](Cp^*)_4(Mes)$ makes the pattern difficult to distinguish.

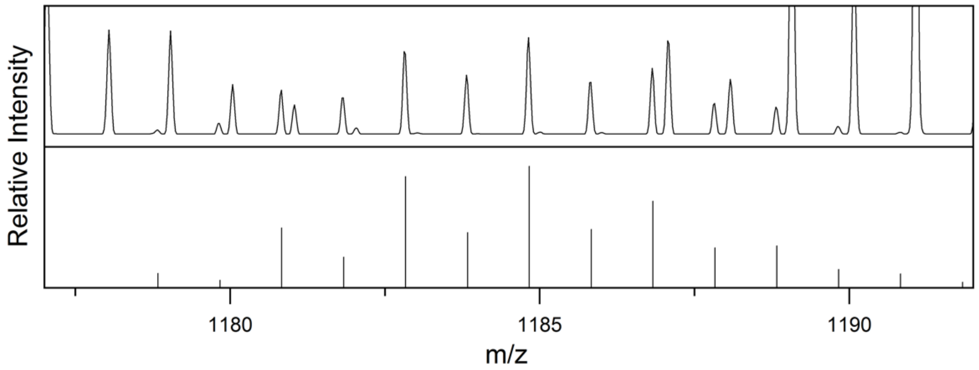

Supplementary Figure 67: Experimental pattern (top) and calculated pattern (bottom) for $[HCu_9](Cp^*)(Mes)_4$. Low signal intensity and overlap with $[Cu_7](Cp^*)_2(Mes)_4$ makes the pattern difficult to distinguish

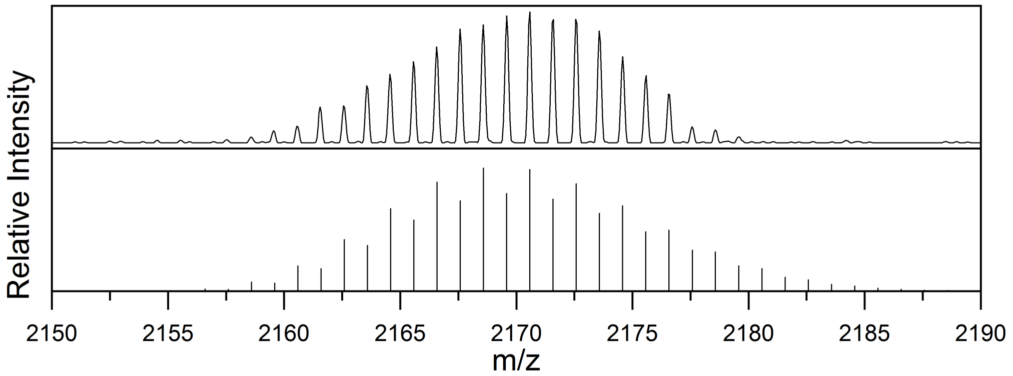

Supplementary Figure 68: Experimental pattern (top) and calculated pattern (bottom) for $[Cu_{11}Zn_6](Cp^*)_7(CO_2)_2(HCO_2)$.

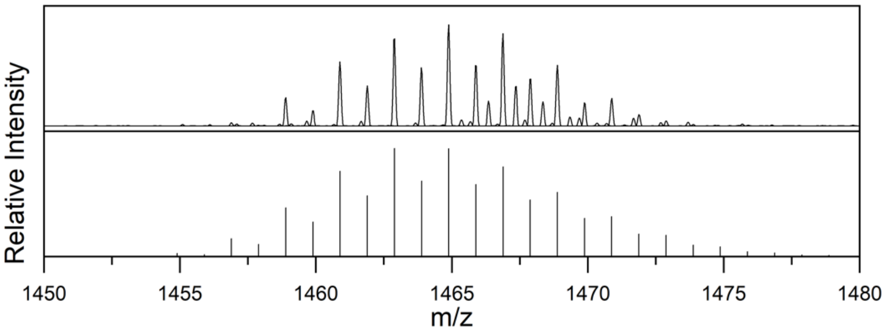

Supplementary Figure 69: Experimental pattern (top) and calculated pattern (bottom) for $[Cu_7Zn_4](Cp^*)_5(Hex)H$.

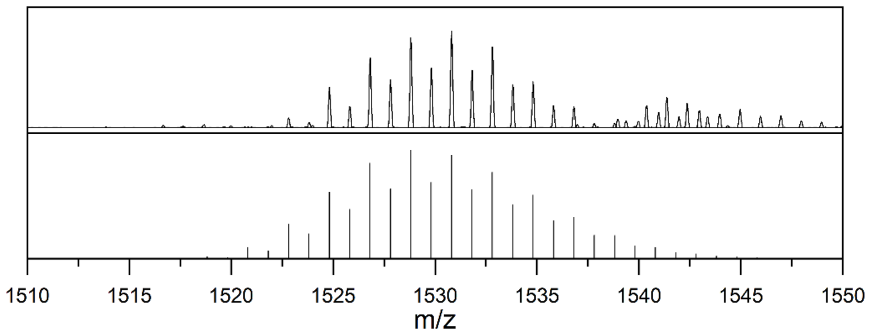

Supplementary Figure 70: Experimental pattern (top) and calculated pattern (bottom) for $[Cu_7Zn_5](Cp^*)_5(Hex)H$.

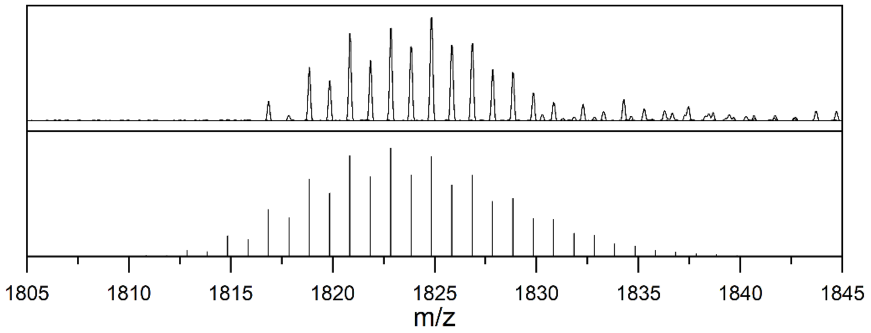

Supplementary Figure 71: Experimental pattern (top) and calculated pattern (bottom) for $[Cu_9Zn_5](Cp^*)_5(Hex)_3H_3$.

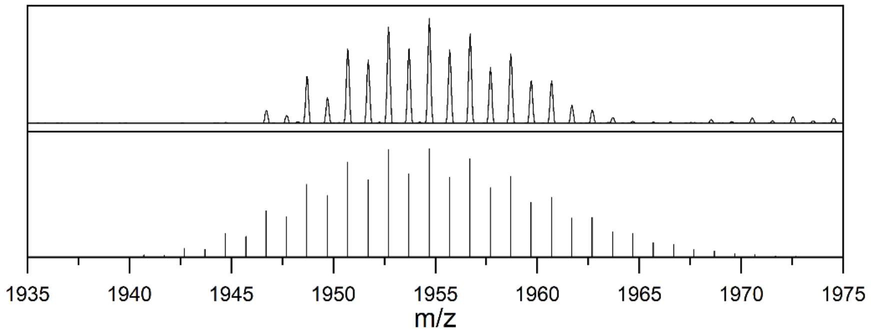

Supplementary Figure 72: Experimental pattern (top) and calculated pattern (bottom) for $[Cu_9Zn_7](Cp^*)_5(Hex)_3H_3$.

1.1.1.4. Fragmentation Analysis

Fragmentation behavior was assessed based on an approach similar to energy dependent ESI-MS[1] and stepped collision energy in orbitrap systems known from peptide fragmentation[2].

Molecular ion peaks are associated with a continuous decrease of peak intensity for increasing collision energies. Species with such a fragmentation behavior may still be fragment formed during the ionization procedure however (e.g. due to thermal or discharge processes on the emitter). For fragment ions, an increase of peak intensity is expected for increasing collision energies due to their enhanced formation at higher collision energies. In some cases, no clear decision can be made either due to fluxional or continuous behavior of peak intensities at rising collision energies.

Spectra were recorded at normalized collision energies between 10 and 23. Note that 10 is the lowest possible collision energy using the given set-up and that use of normalized collision energies in arbitrary units is common for the given set-up.[2] For collision energies higher than 23, an overall decrease of peak intensities for all peaks in the spectrum was observed. Obviously, all the sensitive cluster species in the gas phase, be it fragments or molecular ions, are unstable under these experimental conditions.

Peak intensities of interest (i.e. for the peaks unambiguously identified by labeling experiments) were determined by computerized integration relative to the overall integral of the spectrum. To determine the influence of statistic fluctuations of peak intensities, which are not correlated to the collision energy applied, each experiment was repeated five times. The average value of peak intensity $\bar{I}$, as well as the corresponding coefficient of variation (CV) were calculated for every peak. Statistic fluctuations of peak intensities are supposed to occur during the ionization procedure. The analyte concentration on the LIFDI-emitter, which is hardly controllable during sample supply, chemical/physical modifications on the emitter´s surface (e.g. metal deposition, geometric rearrangements) or thermal decomposition of part of the analyte during ionization might be possible reasons for these fluctuations. 60 mA/s was determined as the optimum heating rate with respect to a minimum variation coefficient of peak intensities under identical measurement conditions.

Data was thereafter analyzed as follows: average peak intensities at elevated collision energies were compared with the average peak intensity at standard conditions. Fragments are associated with an increase in peak intensity with respect to the average peak intensity at standard conditions. Contrary, parent ions are associated with a decrease in peak intensity. In detail, the development of peak intensities with rising collision energy was analyzed by linear regression analysis. A positive slope of the regression line is thereafter indicative of a fragment, a negative one indicative of a parent ion. Only in cases, in which the absolute value of the slope was higher than the standard deviation of the slope, the results can be considered as significant.

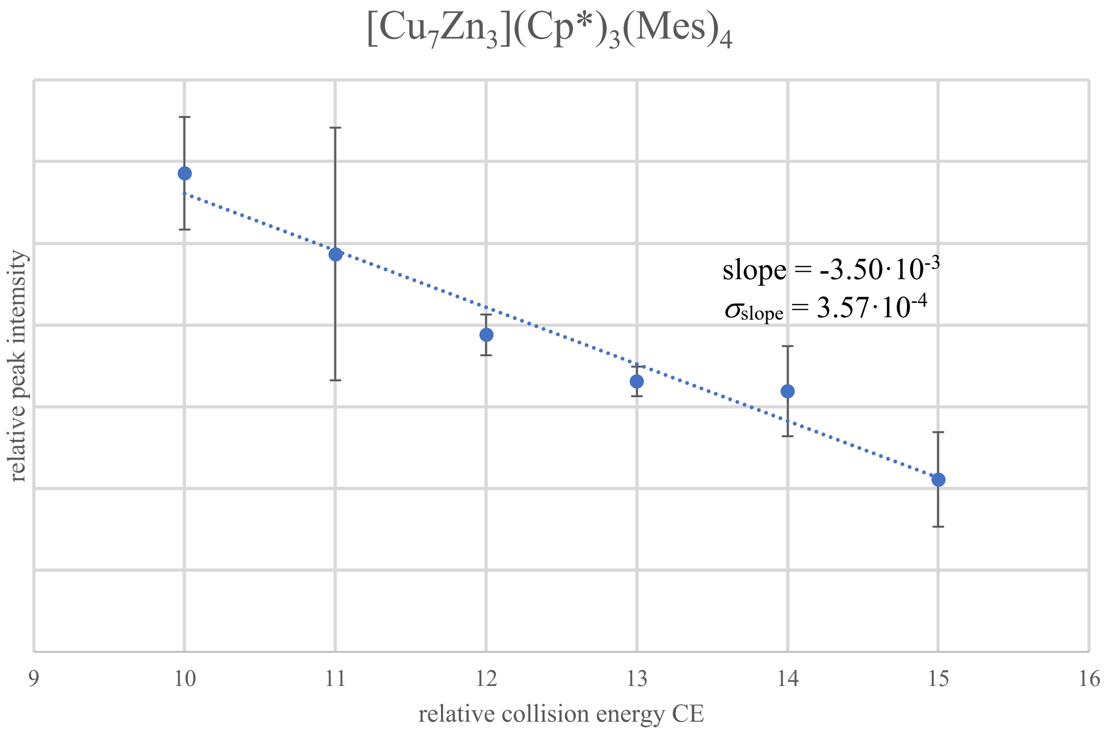

Supplementary Figure 73: CE vs. I plot of the ion $\{[Cu_7Zn_3](Cp^*)_4(Mes)_4\}^+$. The species is clearly assigned as a molecular ion according to the CE vs. I plot. The regression was performed on the statistical means of five independent measurements under identical experimental conditions for each CE value. The error bars represent the standard deviations of these measurements.

The resulting I vs. CE plots for every ion from the Cu/Zn library are shown below. Obviously and as expected, the onset of fragmentation processes, as well as the extent of fragmentation with respect to collision energy, are different for different ions. Whereas for the molecular ion $\{[Cu_7Zn_3](Cp^*)_3(Mes)_4\}^+$ (Supplementary Figure 73) a continuous decrease in peak intensity is detected, the situation is more complicated for $\{[HCu_5](Cp^*)_2(Mes)\ \}^+$: deviation from linearity is observed at higher collision energies (17 and 18). The latter effect is explained by significant production of light fragments with *m/z* < 200, escaping the overall integral and distorting therefore the determined relative peak intensities (which are calculated with respect to the overall integral of all peaks in the spectrum). Similar effects were observed for several peaks. Due to the intrinsically different onset of fragmentation and due to the bias in the data at higher collision energies, the linear region in the CE vs. I plots is slightly different for different ions. Noteworthy, the analysis by linear regression does therefore only allow for qualitative analysis (fragment vs. parent ion) but not for a quantitative analysis of fragmentation processes.

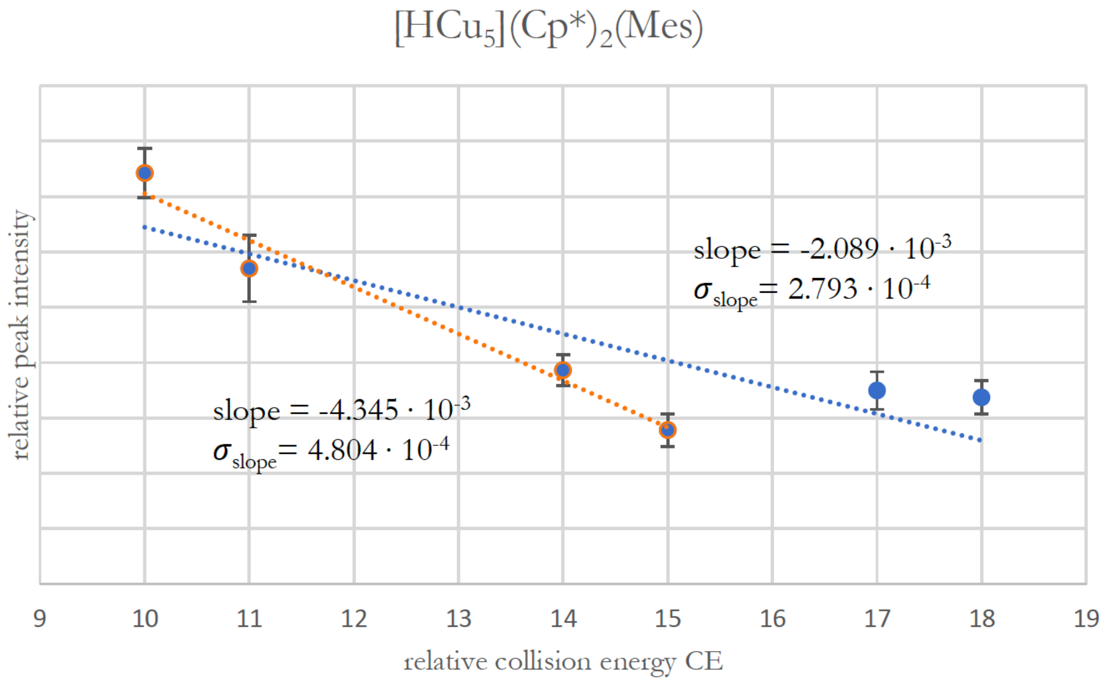

Supplementary Figure 74: CE vs. I plot of the ion $\{[HCu_5](Cp^*)_2(Mes)\}^+$. The species is clearly assigned as a molecular ion according to the CE vs. I plot. The slight increase in peak intensity at CE > 15 is supposed to be due to the bias in integration. The regression was performed on the statistical means of five independent measurements under identical experimental conditions for each CE value. The error bars represent the standard deviations of these measurements.

The resulting list of all molecular ion species and fragments obtained by careful analysis of all I vs. CE plots are given in Supplementary Tables 2 and 3. For some species, no significant variation in peak intensity was detected. Hence, no decision whether they are fragments or not is possible. They might either be rather robust molecular ions that do not undergo fragmentation or fragment ions prone to further fragmentation reactions. The latter situation would lead to a "steady state" situation, in which formation and decay of the species is in equilibrium and therefore independent on the collision energy applied. With the data and instrumentation at hand it is not possible to experimentally assign a fragment to a specific parent ion, which is due to the lack of an ion trap allowing for isolation of specific ions and study of their decay. However, the fragments can be tentatively assigned to a parent ion according to chemical considerations and according to fragmentation behavior observed for isolated species.

Note: Peak intensities were determined as relative integrals with respect to the overall integral of all peaks in the spectrum. This leads to a bias in integration at CE > 15 due to the enhanced formation of lighter fragments with m/z < 200, lying outside the measurement range. Actual peak intensities are therefore expected to be lower than detected at CE > 15. For the ions to which this situation applies, an additional fit in plotted for intensities until CE = 15 (in orange).

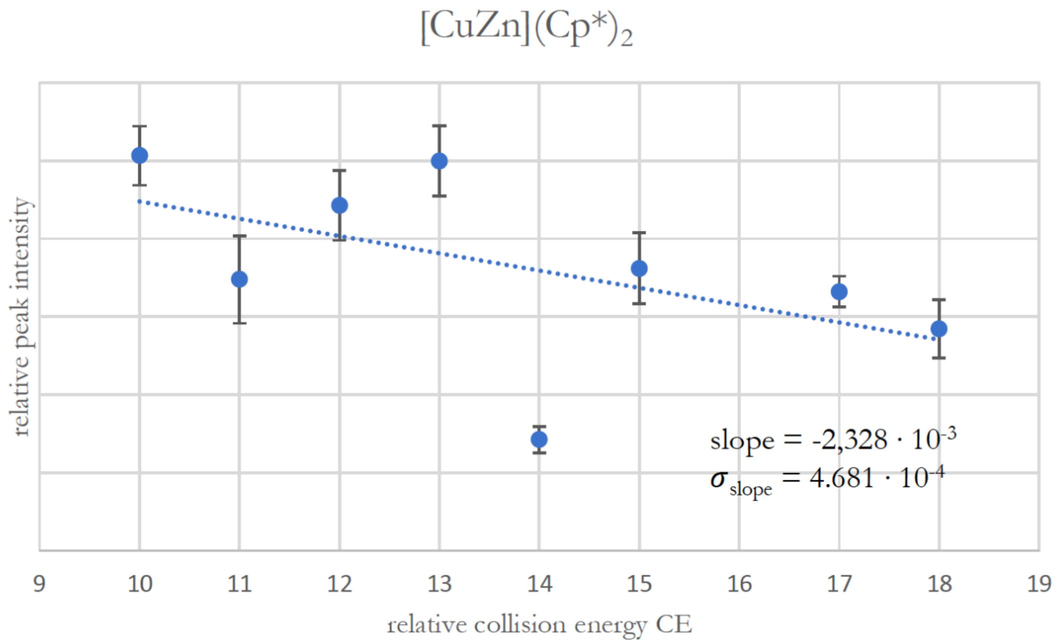

Supplementary Figure 75: CE vs. I plot of the ion {[CuZn]$(Cp^*)_2$}$^+$. The ion was identified in the LIFDI-MS spectrum of isolated **A**. The CE vs. I plot is indicative of a molecular ion. The species is supposed to be a fragment of **A**, which is formed (thermally) during the ionization process. Consequently, it shows a CE vs. I behavior like a molecular ion. The regression was performed on the statistical means of five independent measurements under identical experimental conditions for each CE value. The error bars represent the standard deviations of these measurements.

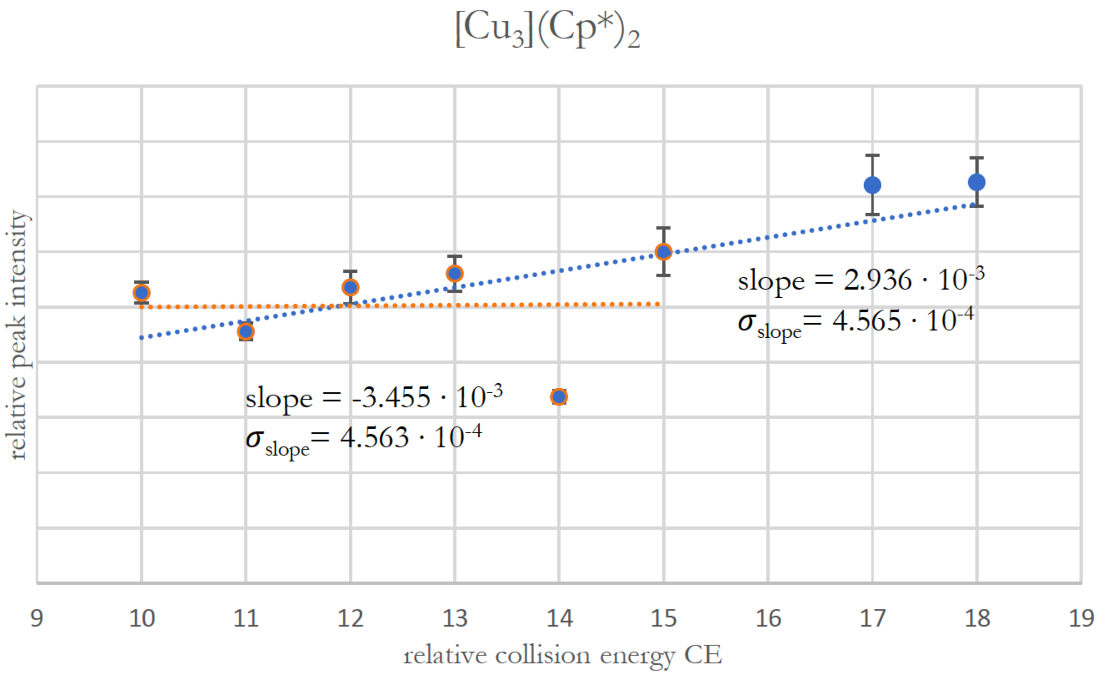

Supplementary Figure 76: CE vs. I plot of the ion {$[Cu_3](Cp^*)_2$}$^+$. The CE vs. I plot is indicative of a fragment ion. The species was observed in the LIFDI-MS spectrum of isolated **A**. The regression was performed on the statistical means of five independent measurements under identical experimental conditions for each CE value. The error bars represent the standard deviations of these measurements.

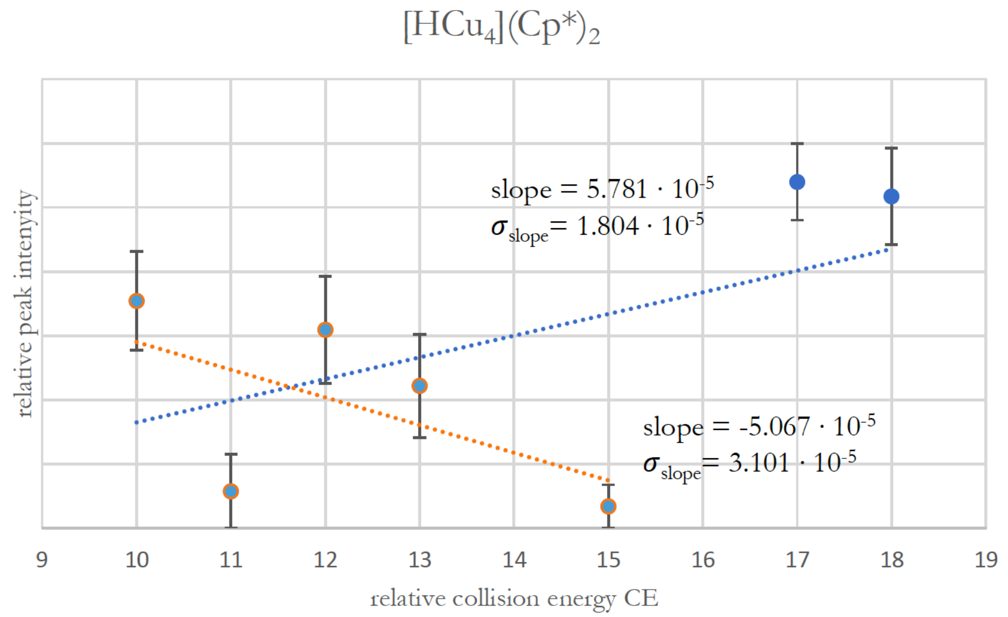

Supplementary Figure 77: CE vs. I plot of the ion $\{[HCu_4](Cp^*)_2\}^+$. The species was identified in the LIFDI-MS spectrum of isolated **A**. The CE vs. I plot is indicative of a molecular ion if considering collision energies from 10-15. At higher collision energies, data interpretation is difficult due to the bias in integration. The species is supposed to be a fragment, which is formed (thermally) during the ionization process. Consequently, it shows a CE vs. I behavior like a molecular ion. The regression was performed on the statistical means of five independent measurements under identical experimental conditions for each CE value. The error bars represent the standard deviations of these measurements.

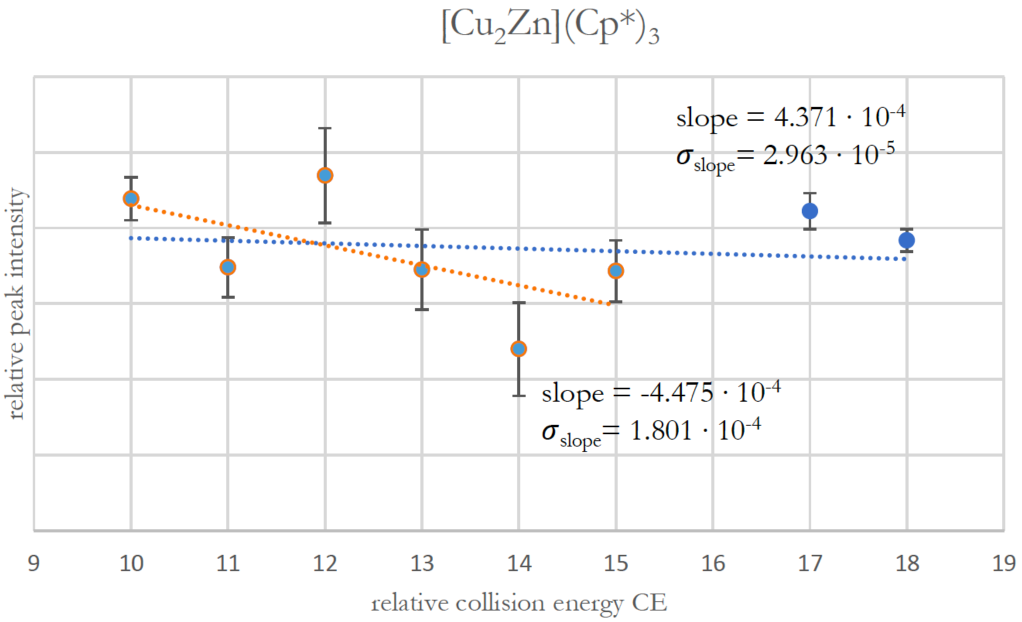

Supplementary Figure 78: CE vs. I plot of the ion $\{[Cu_2Zn](Cp^*)_3\}^+$. A clear assignment of the species is not possible. According to the slope in the CE = 10-15 region, the species might be a molecular ion. The regression was performed on the statistical means of five independent measurements under identical experimental conditions for each CE value. The error bars represent the standard deviations of these measurements.

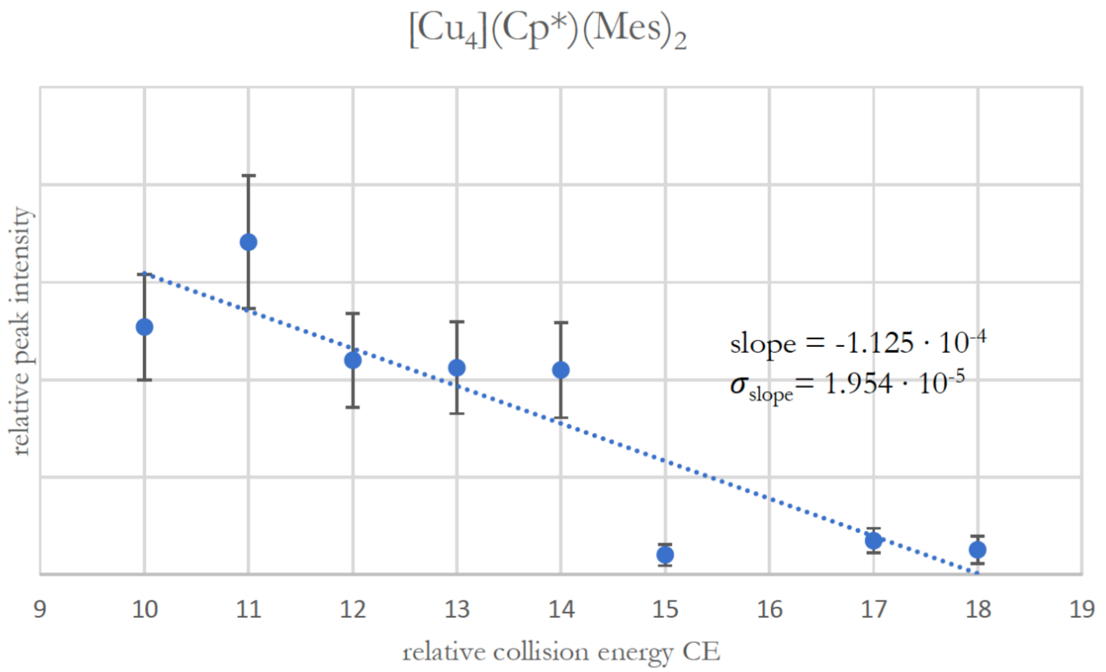

Supplementary Figure 79: CE vs. I plot of the ion $\{[Cu_4](Cp^*)(Mes)_2\}^+$. The species is clearly assigned as a molecular ion according to the CE vs. I plot. However, it may also be a fragment formed during ionization. The regression was performed on the statistical means of five independent measurements under identical experimental conditions for each CE value. The error bars represent the standard deviations of these measurements.

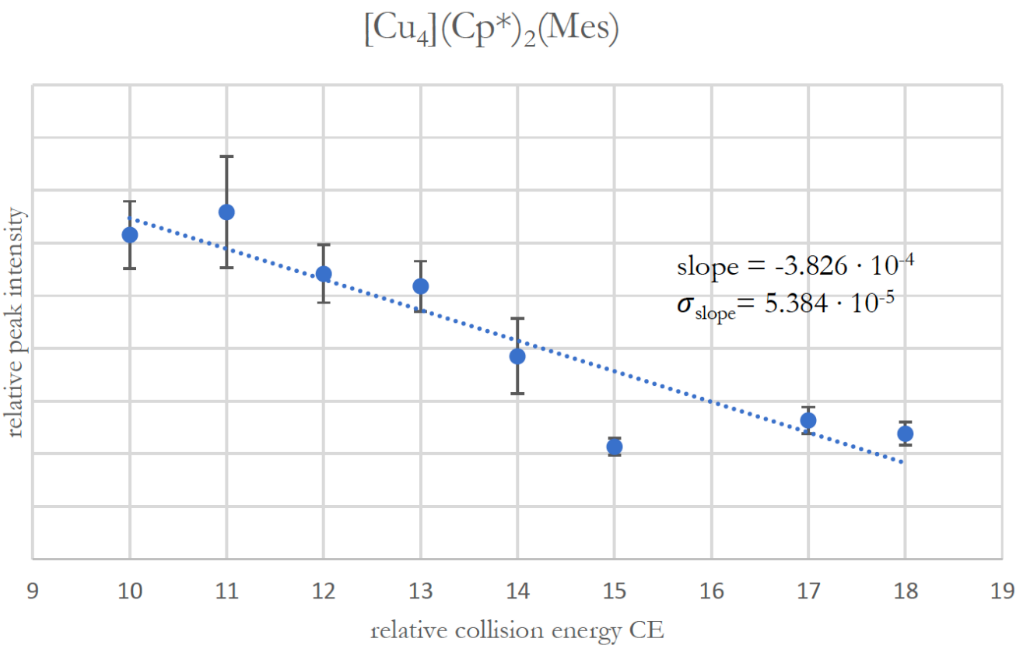

Supplementary Figure 80: CE vs. I plot of the ion $\{[Cu_4](Cp^*)_2(Mes)\}^+$. The species is clearly assigned as a molecular ion according to the CE vs. I plot. However, it may also be a fragment formed during ionization. The regression was performed on the statistical means of five independent measurements under identical experimental conditions for each CE value. The error bars represent the standard deviations of these measurements.

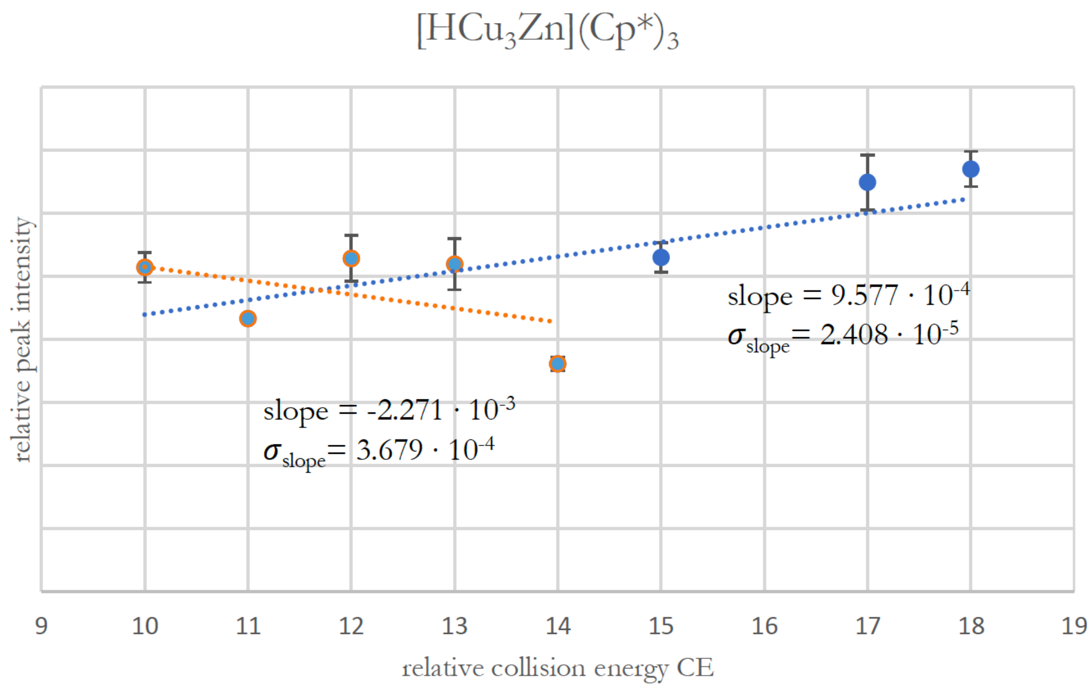

Supplementary Figure 81: CE vs. I plot of the ion {$[HCu_3Zn](Cp^*)_3$}$^+$.A clear assignment of the species is not possible. However according to the CE = 10-14 region, it may be assigned as a molecular ion. The regression was performed on the statistical means of five independent measurements under identical experimental conditions for each CE value. The error bars represent the standard deviations of these measurements.

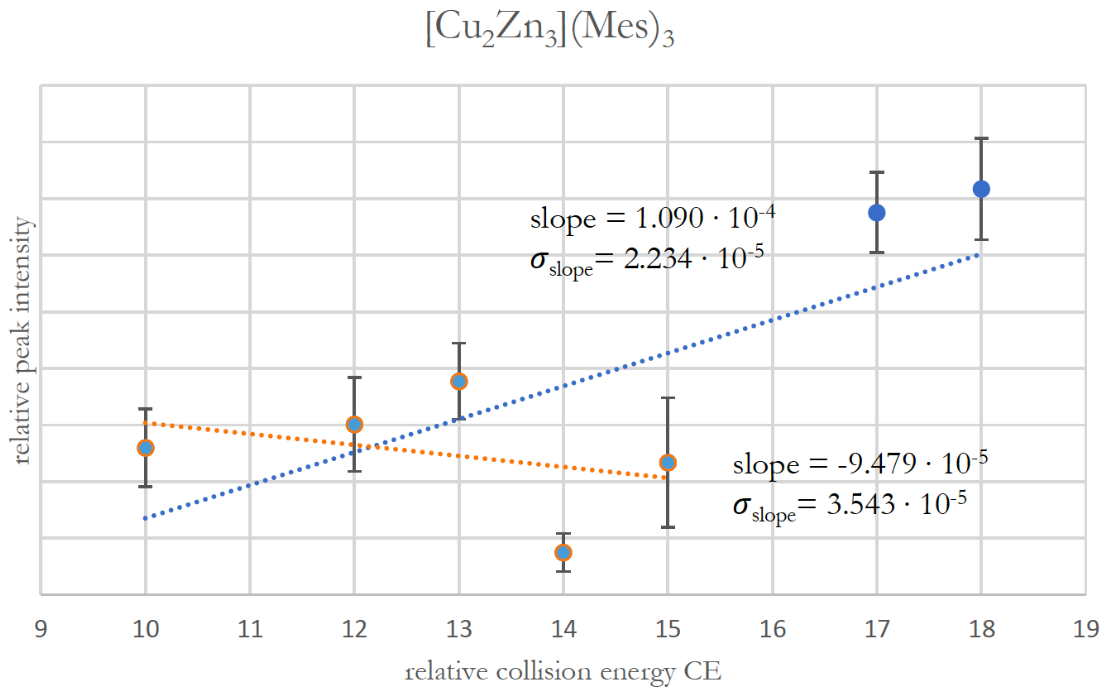

Supplementary Figure 82: CE vs. I plot of the ion {$[Cu_2Zn](Mes)_3$}$^+$. A clear assignment of the species is not possible. However according to the CE = 10-14 region, it may be assigned as a molecular ion. The regression was performed on the statistical means of five independent measurements under identical experimental conditions for each CE value. The error bars represent the standard deviations of these measurements.

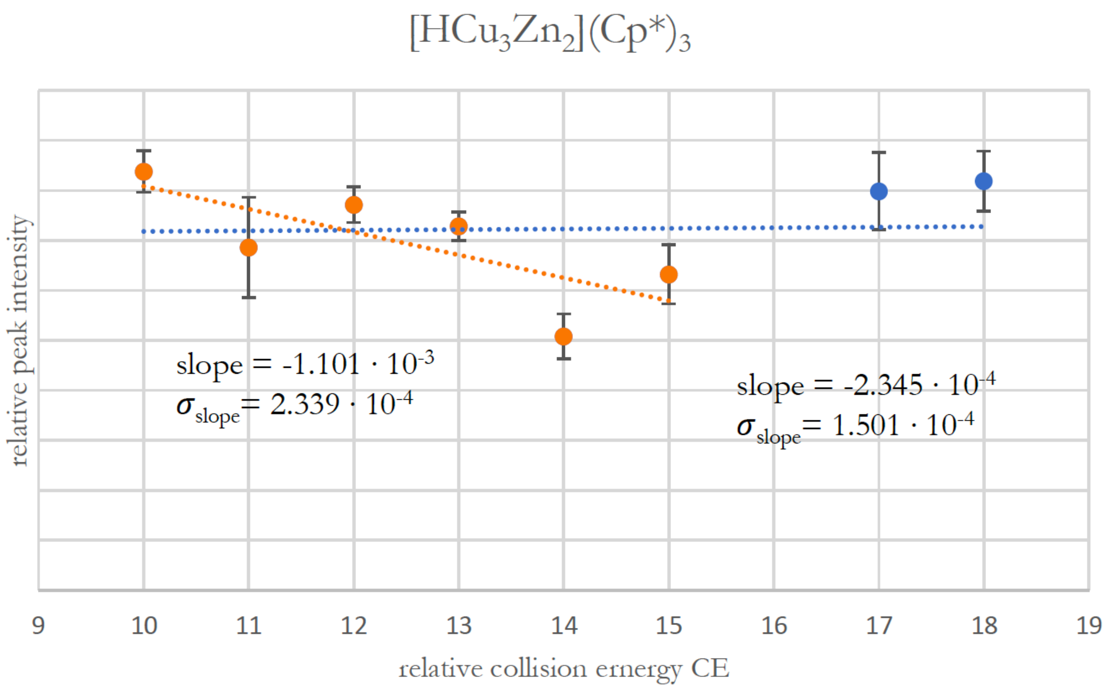

Supplementary Figure 83: CE vs. I plot of the ion $\{[HCu_3Zn_2](Cp^*)_3\}^+$. The CE vs. I plot is indicative of a molecular ion if considering collision energies from 10-15. At higher collision energies, data interpretation is difficult due to the bias in integration. The species is supposed to be a fragment of **A**, which is formed (thermally) during the ionization process. Consequently, it shows a CE vs. I behavior like a molecular ion. The regression was performed on the statistical means of five independent measurements under identical experimental conditions for each CE value. The error bars represent the standard deviations of these measurements.

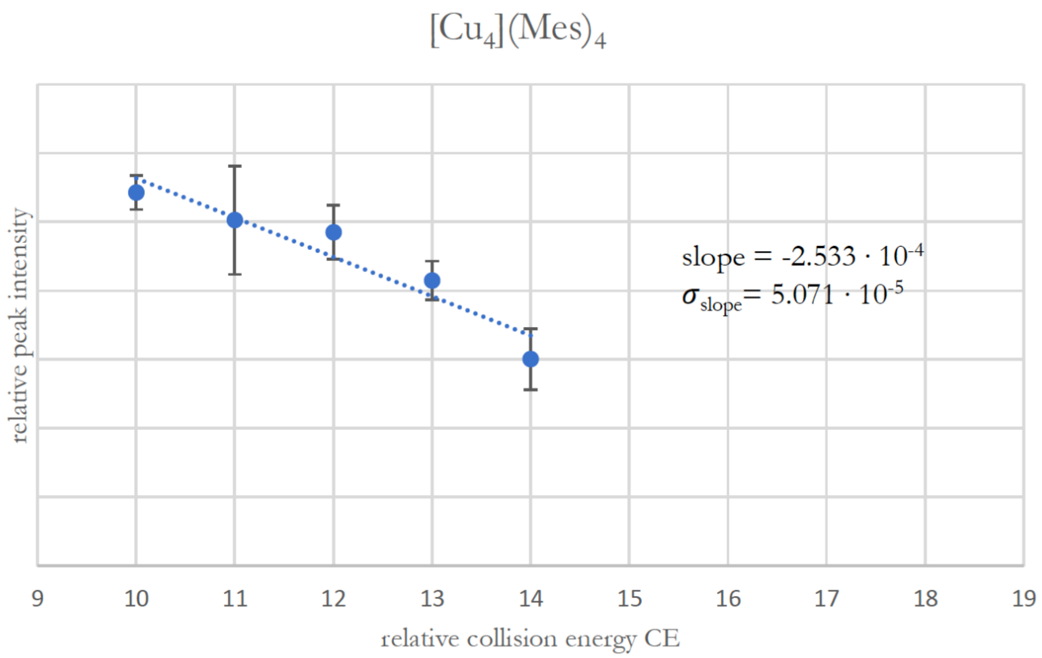

Supplementary Figure 84: CE vs. I plot of the ion $\{[Cu_4](Mes)_4\}^+$. According to the CE vs. I plot, the species clearly is assigned as molecular ion. However, it is supposed that the species is formed out of $[Cu_5](Mes)_5$ by loss of one CuMes unit during ionization. The regression was performed on the statistical means of five independent measurements under identical experimental conditions for each CE value. The error bars represent the standard deviations of these measurements.

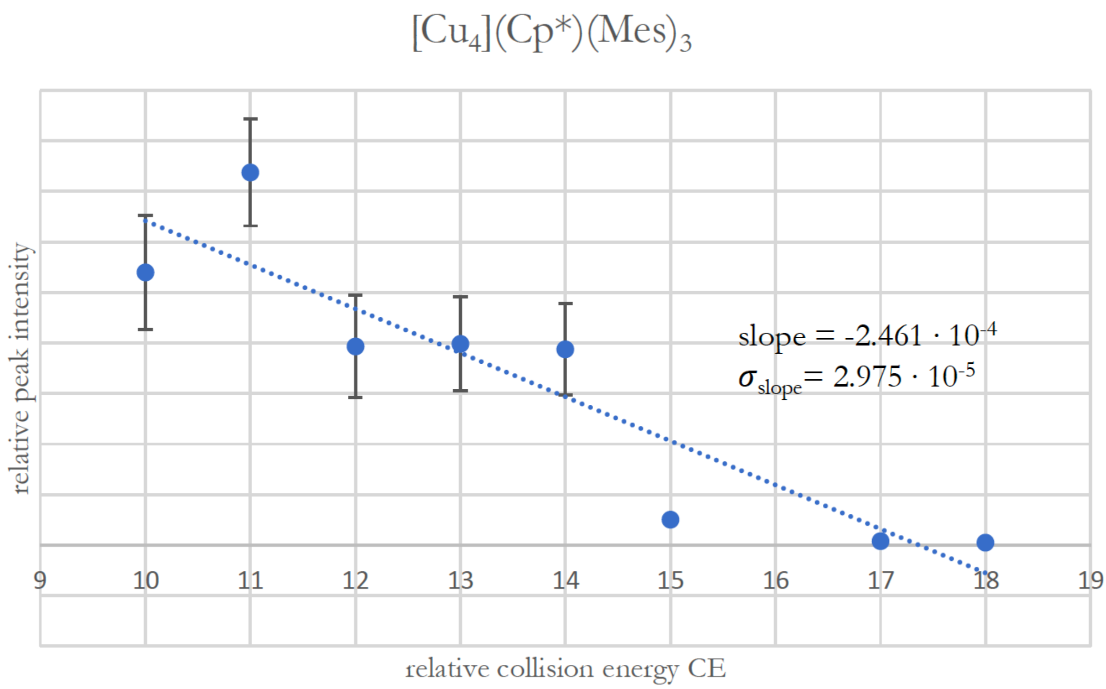

Supplementary Figure 85: CE vs. I plot of the ion $\{[Cu_4](Cp^*)(Mes)_3\}^+$. The species is clearly assigned as a molecular ion according to the CE vs. I plot. However, it may also be a fragment formed during ionization. The regression was performed on the statistical means of five independent measurements under identical experimental conditions for each CE value. The error bars represent the standard deviations of these measurements.

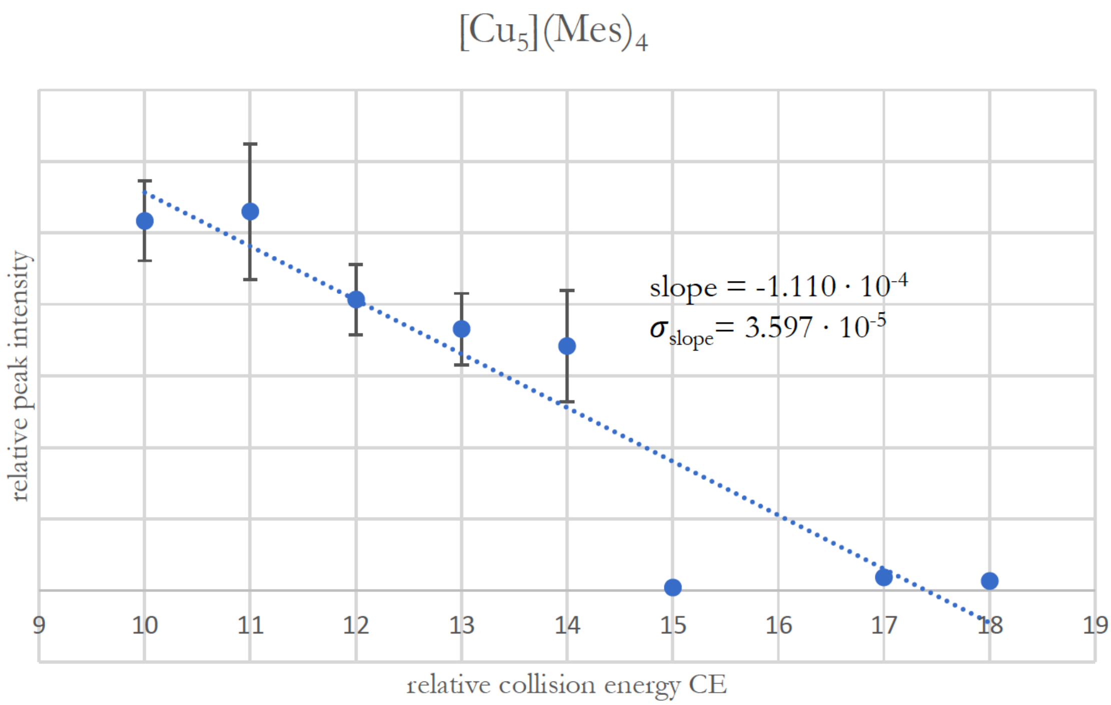

Supplementary Figure 86: CE vs. I plot of the ion $\{[Cu_5](Mes)_4\}^+$. According to the CE vs. I plot, the species clearly is assigned as molecular ion. However, it is supposed that the species is formed out of $[Cu_5](Mes)_5$ by loss of one Mes unit during ionization. The regression was performed on the statistical means of five independent measurements under identical experimental conditions for each CE value. The error bars represent the standard deviations of these measurements.

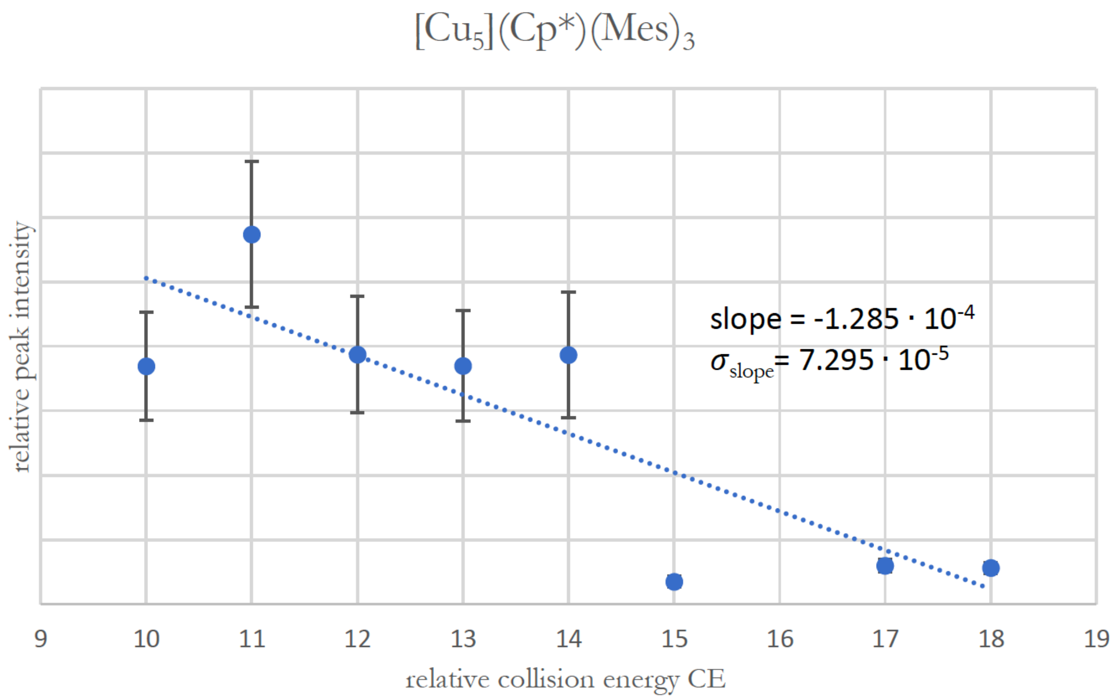

Supplementary Figure 87: CE vs. I plot of the ion $\{[Cu_5](Cp^*)(Mes)_3\}^+$. The species is clearly assigned as a molecular ion according to the CE vs. I plot. However, it may also be a fragment formed during ionization. The regression was performed on the statistical means of five independent measurements under identical experimental conditions for each CE value. The error bars represent the standard deviations of these measurements.

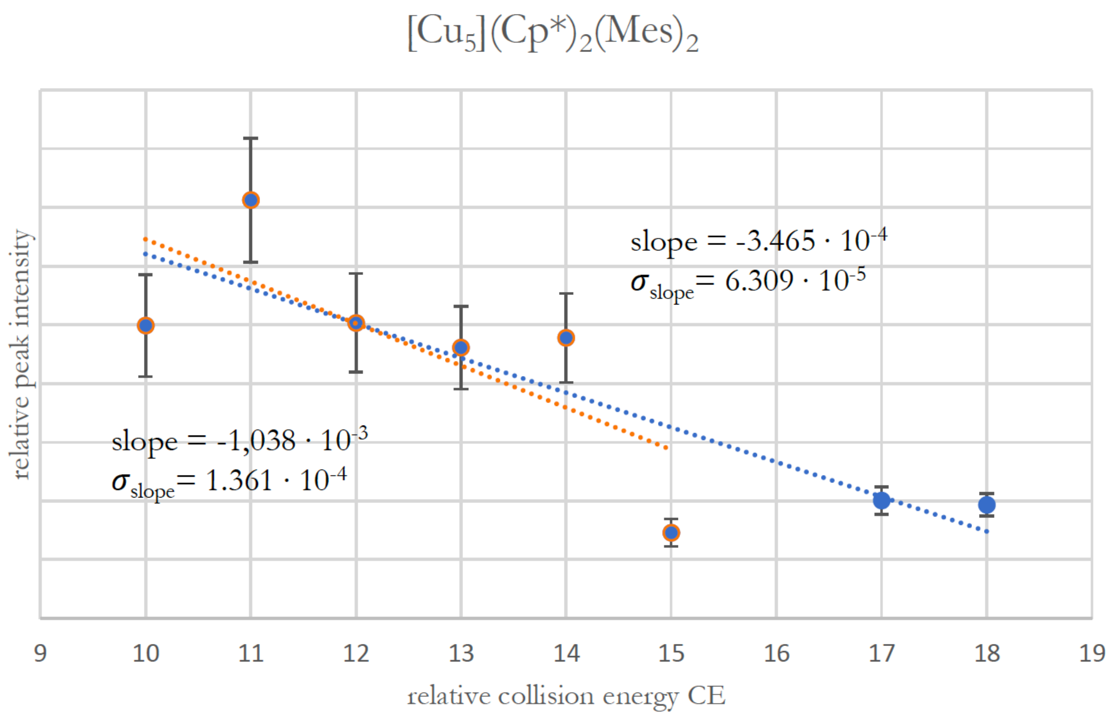

Supplementary Figure 88: CE vs. I plot of the ion $\{[Cu_5](Cp^*)_2(Mes)_2\}^+$. The species is clearly assigned as a molecular ion according to the CE vs. I plot. However, it may also be a fragment formed during ionization. The regression was performed on the statistical means of five independent measurements under identical experimental conditions for each CE value. The error bars represent the standard deviations of these measurements.

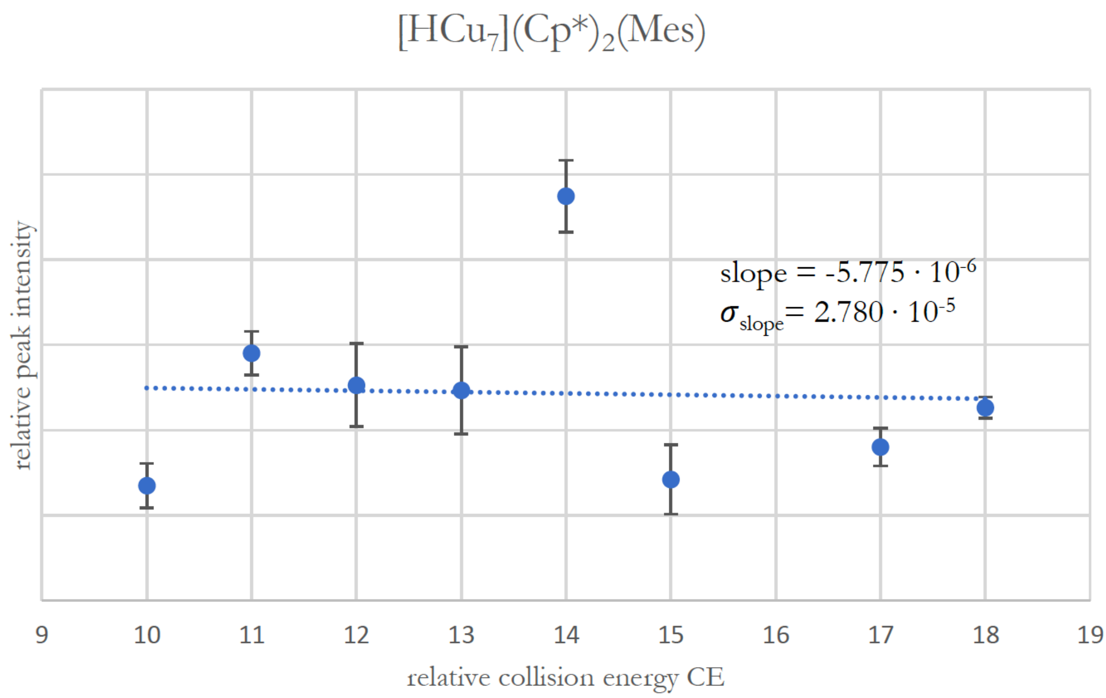

Supplementary Figure 89: CE vs. I plot of the ion $\{[HCu_7](Cp^*)_2(Mes)\}^+$. A clear assignment of the species is not possible. The regression was performed on the statistical means of five independent measurements under identical experimental conditions for each CE value. The error bars represent the standard deviations of these measurements.

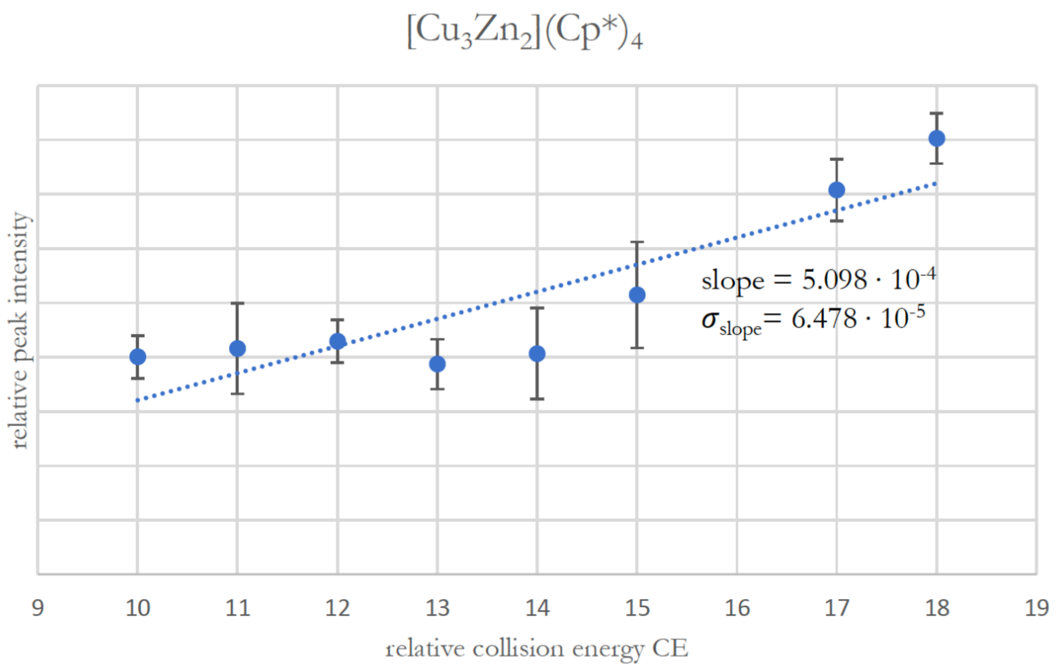

Supplementary Figure 90: CE vs. I plot of the ion $\{[Cu_3Zn_2](Cp^*)_4\}^+$. A clear assignment is difficult to make for this species, as the CE vs. I plot in the CE>15 region has to be regarded with care due to the bias in integration. However, an assignment as a fragment ion seems likely. The regression was performed on the statistical means of five independent measurements under identical experimental conditions for each CE value. The error bars represent the standard deviations of these measurements.

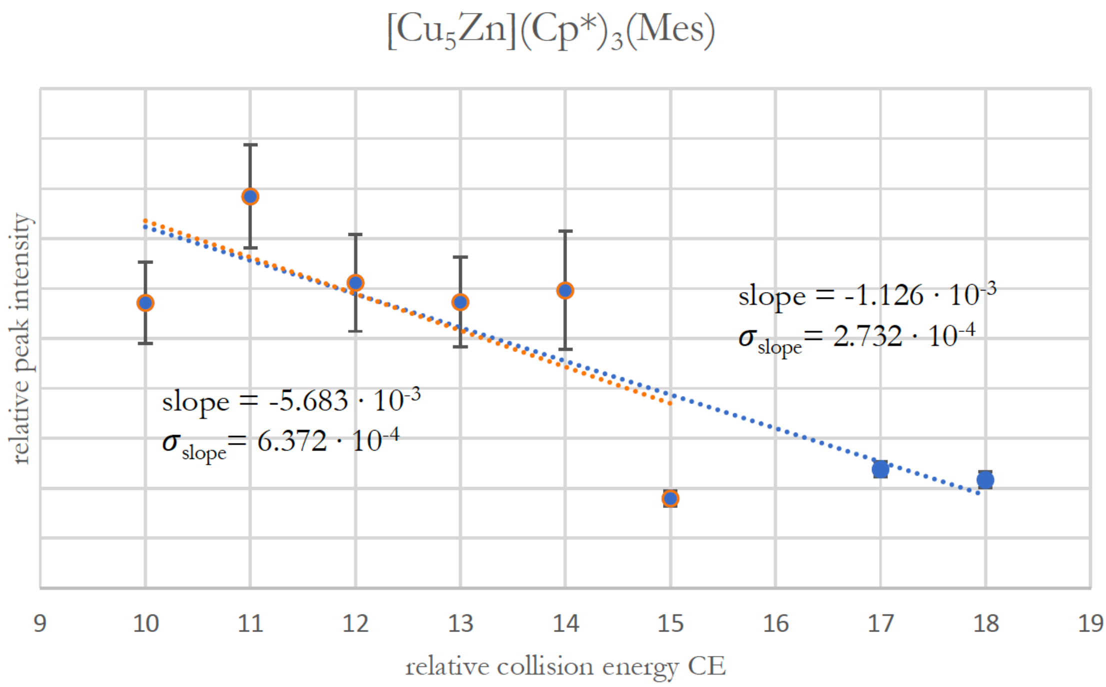

Supplementary Figure 91: CE vs. I plot of the ion $\{[Cu_5Zn](Cp^*)_3(Mes)\}^+$. The species is clearly assigned as a molecular ion according to the CE vs. I plot. However, it may also be a fragment formed during ionization. The regression was performed on the statistical means of five independent measurements under identical experimental conditions for each CE value. The error bars represent the standard deviations of these measurements.

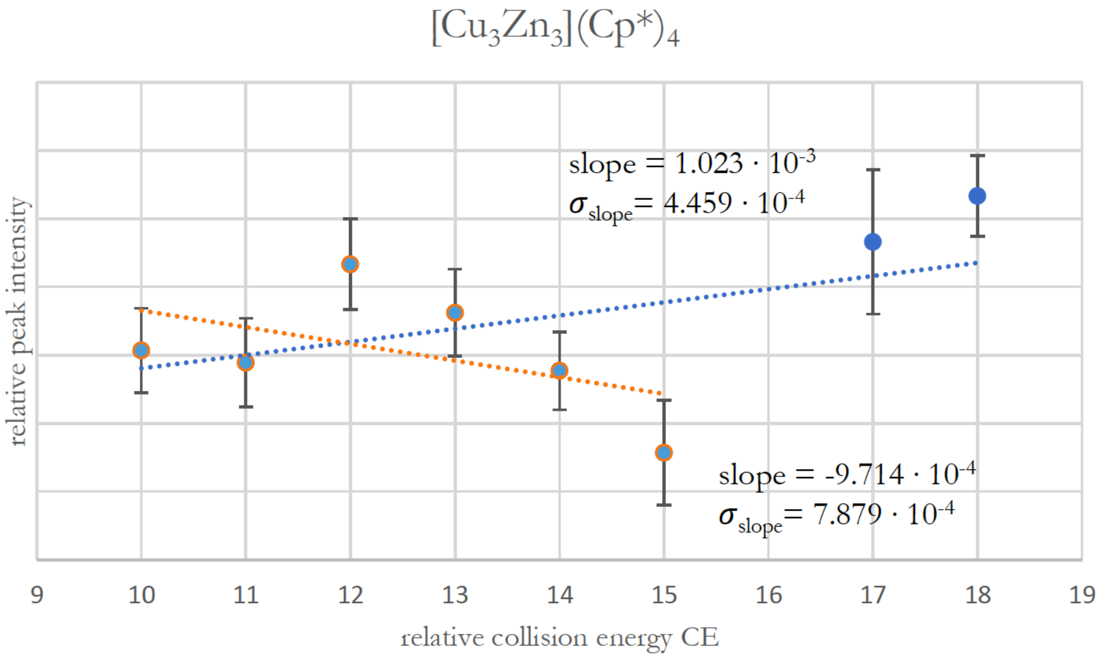

Supplementary Figure 92: CE vs. I plot of the ion $\{[Cu_3Zn_3](Cp^*)_4\}^+$. The data at CE > 15 has to be regarded with care due to bias in data integration. The regression was performed on the statistical means of five independent measurements under identical experimental conditions for each CE value. The error bars represent the standard deviations of these measurements.

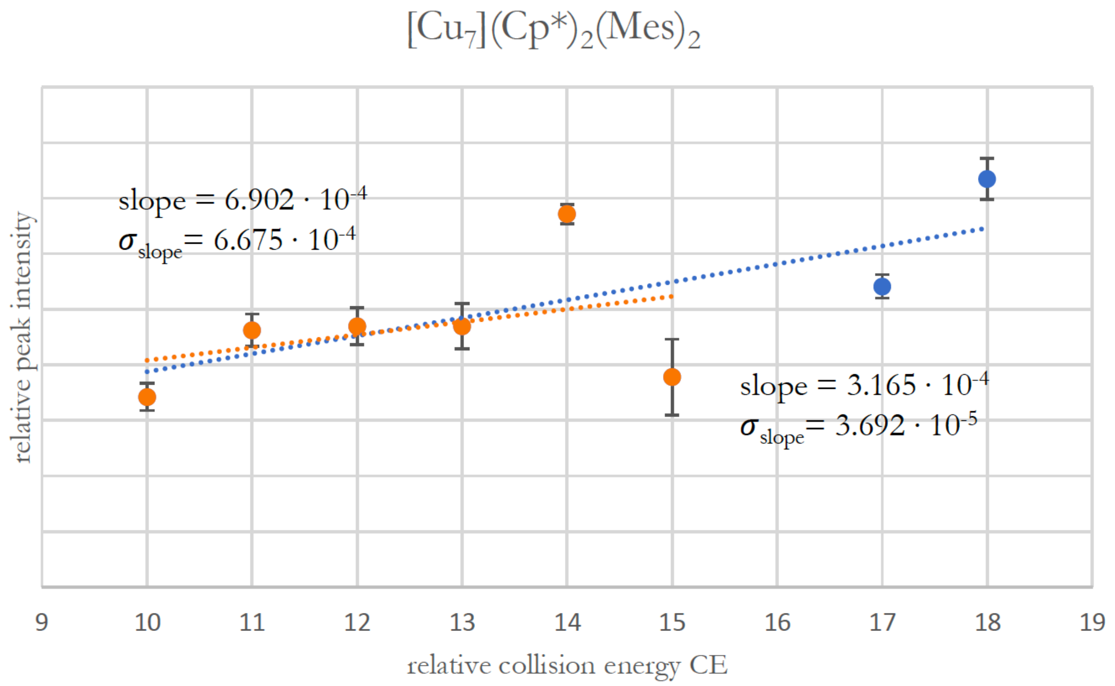

Supplementary Figure 93: CE vs. I plot of the ion $\{[Cu_7](Cp^*)_2(Mes)_2\}^+$. Due to the bias in integration at CE > 15, a clear assignment is difficult for this species. However, it is likely to be a fragment ion (positive slope also in the CE = 10 – 15 region). The regression was performed on the statistical means of five independent measurements under identical experimental conditions for each CE value. The error bars represent the standard deviations of these measurements.

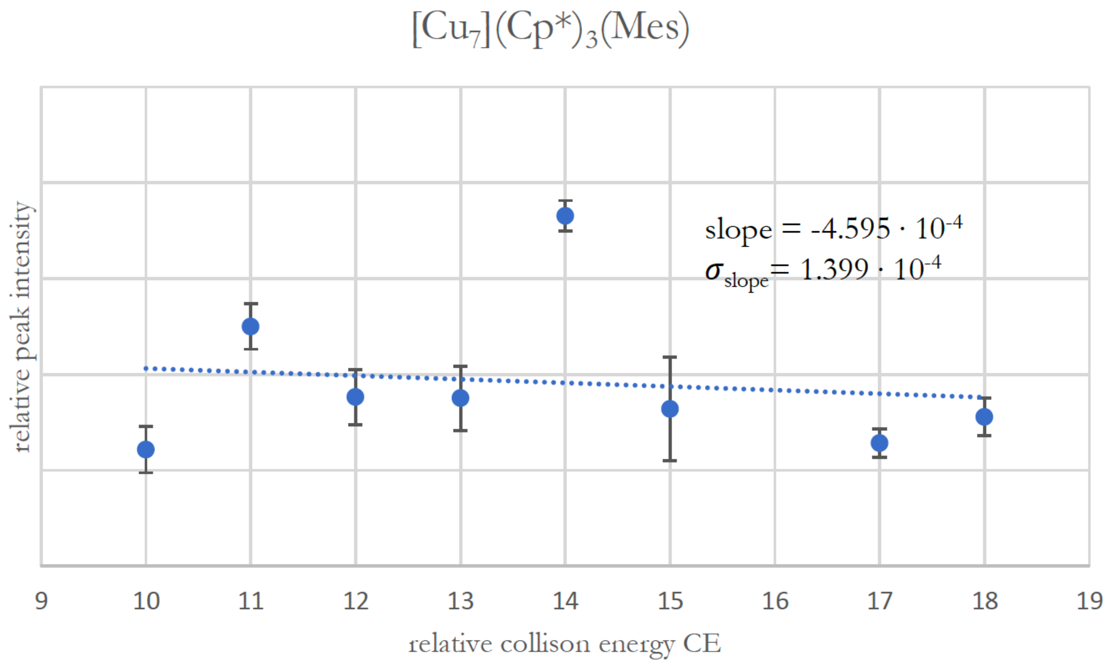

Supplementary Figure 94: CE vs. I plot of the ion $\{[Cu_7](Cp^*)_3(Mes)\}^+$. Due to the steady-state behavior, a clear assignment is not possible for this species. The regression was performed on the statistical means of five independent measurements under identical experimental conditions for each CE value. The error bars represent the standard deviations of these measurements.

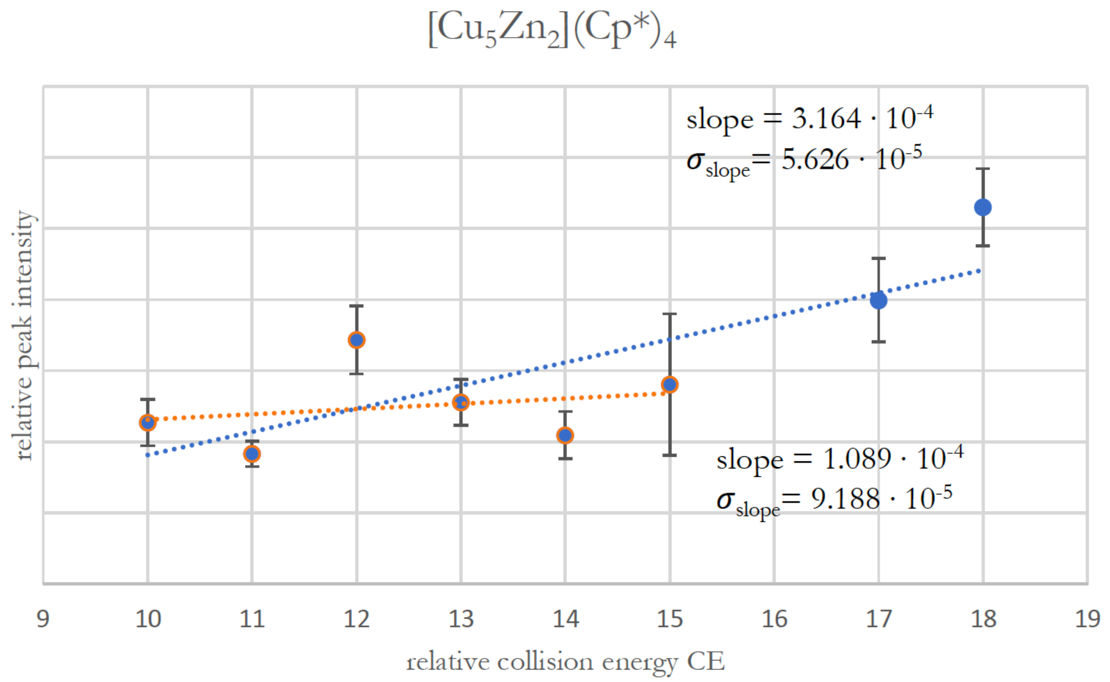

Supplementary Figure 95: CE vs. I plot of the ion $\{[Cu_5Zn_2](Cp^*)_4\}^+$. A clear assignment for this species is difficult due to the bias in integration at CE > 15. However, it is supposed to be a fragment ion. The regression was performed on the statistical means of five independent measurements under identical experimental conditions for each CE value. The error bars represent the standard deviations of these measurements.

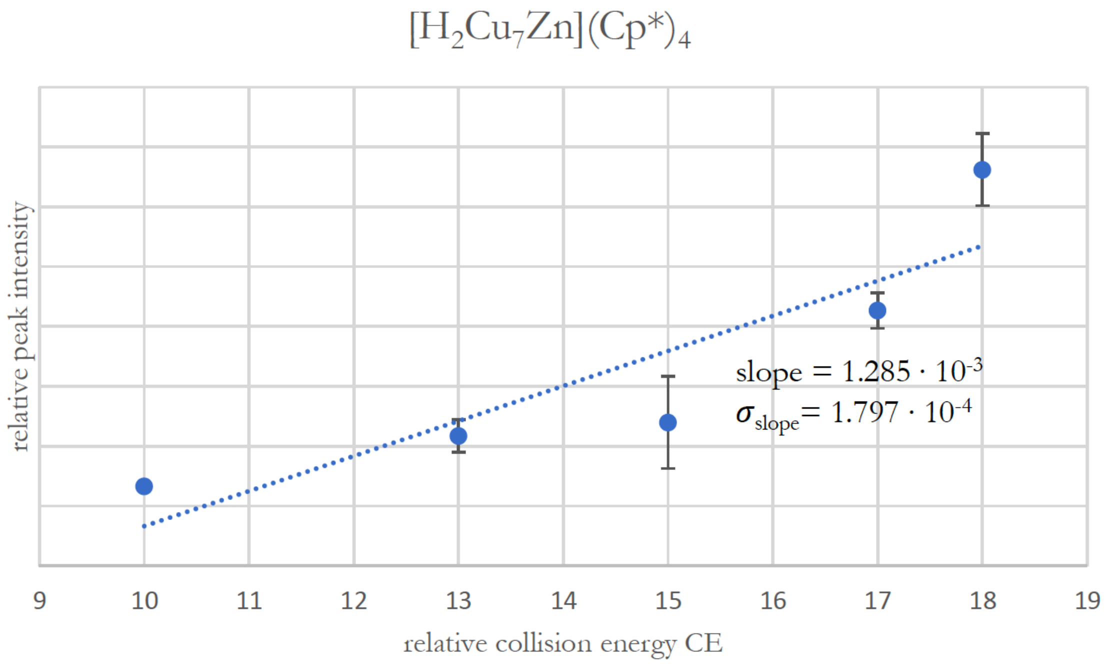

Supplementary Figure 96: CE vs. I plot of the ion $\{[H_2Cu_7Zn](Cp^*)_4\}^+$. The species is identified as a fragment in isolated **A**. It shows also the behavior of a fragment ion in the CE vs. I plot. The regression was performed on the statistical means of five independent measurements under identical experimental conditions for each CE value. The error bars represent the standard deviations of these measurements.

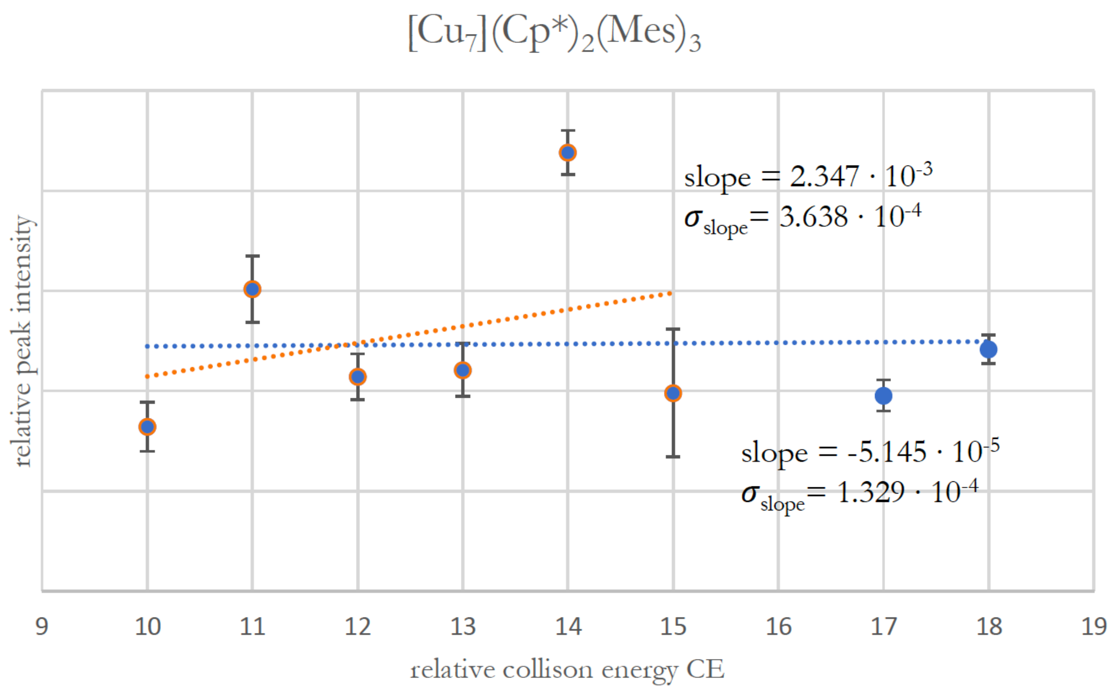

Supplementary Figure 97: CE vs. I plot of the ion $\{[Cu_7](Cp^*)_2(Mes)_3\}^+$. A clear assignment is not possible for this species (no clear trend). The regression was performed on the statistical means of five independent measurements under identical experimental conditions for each CE value. The error bars represent the standard deviations of these measurements.

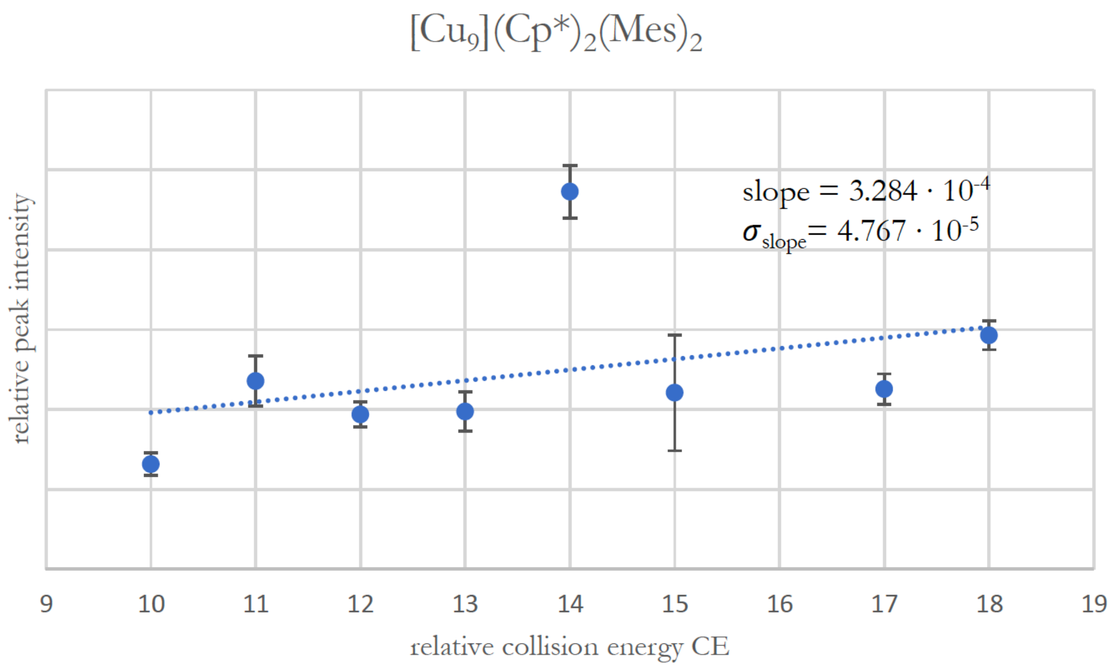

Supplementary Figure 98: CE vs. I plot of the ion $\{[Cu_9](Cp^*)_2(Mes)_2\}^+$. A clear assignment is not possible for this species due to the bias in integration at CE >15. The regression was performed on the statistical means of five independent measurements under identical experimental conditions for each CE value. The error bars represent the standard deviations of these measurements.

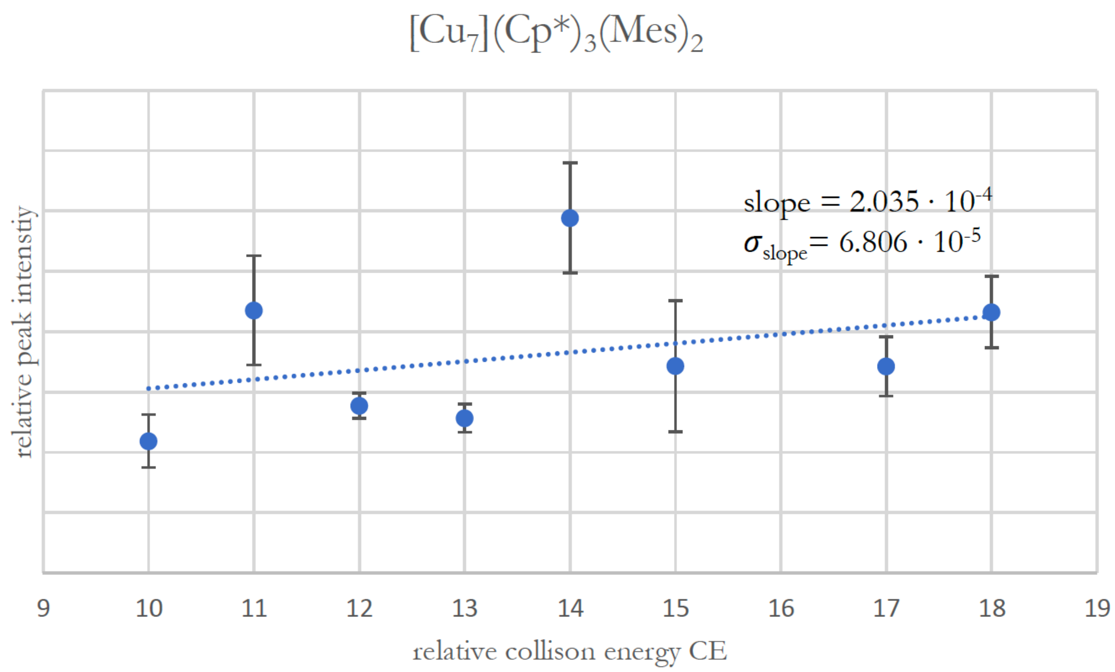

Supplementary Figure 99: CE vs. I plot of the ion $\{[Cu_7](Cp^*)_3(Mes)_2\}^+$. A clear assignment is not possible for this species due to the bias in integration at CE > 15. The regression was performed on the statistical means of five independent measurements under identical experimental conditions for each CE value. The error bars represent the standard deviations of these measurements.

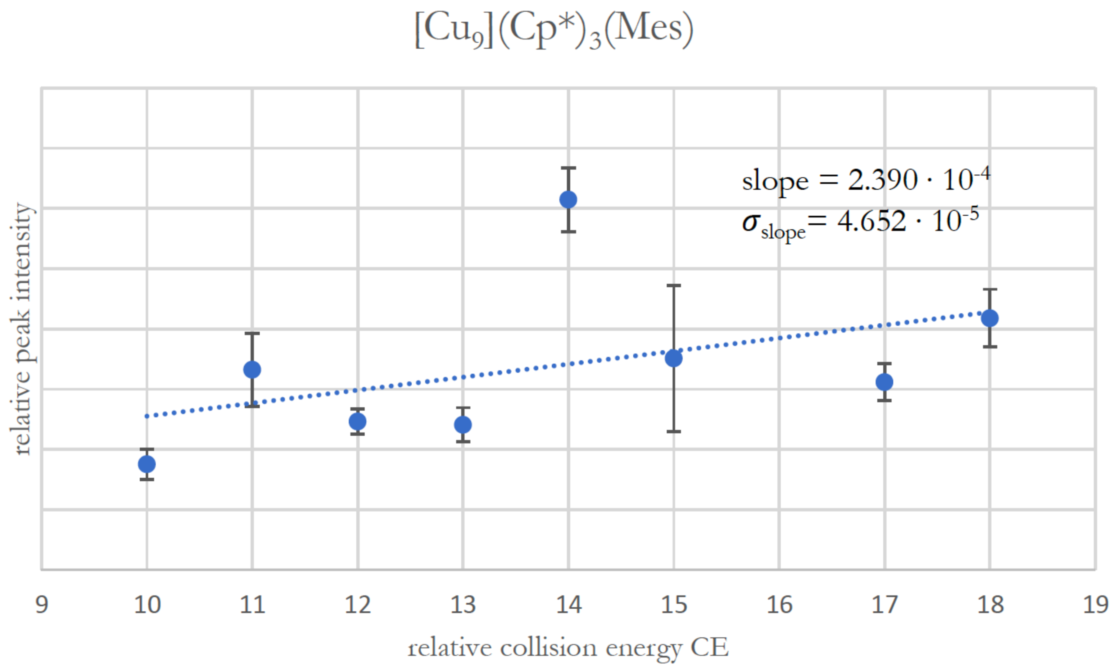

Supplementary Figure 100: CE vs. I plot of the ion $\{[Cu_9](Cp^*)_3(Mes)\}^+$. A clear assignment is not possible for this species due to the bias in integration at CE > 15. The regression was performed on the statistical means of five independent measurements under identical experimental conditions for each CE value. The error bars represent the standard deviations of these measurements.

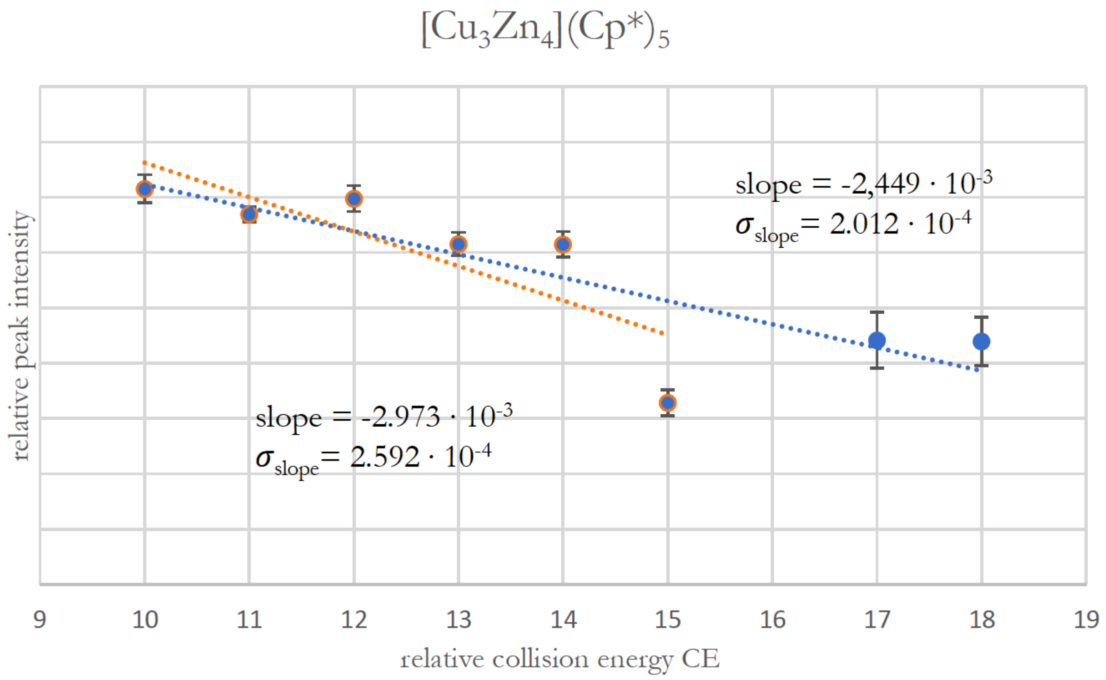

Supplementary Figure 101: CE vs. I plot of the ion $\{[Cu_3Zn_4](Cp^*)_5\}^+$ (**B**$^+$). The species is clearly assigned as a molecular ion and can also be isolated. The regression was performed on the statistical means of five independent measurements under identical experimental conditions for each CE value. The error bars represent the standard deviations of these measurements.

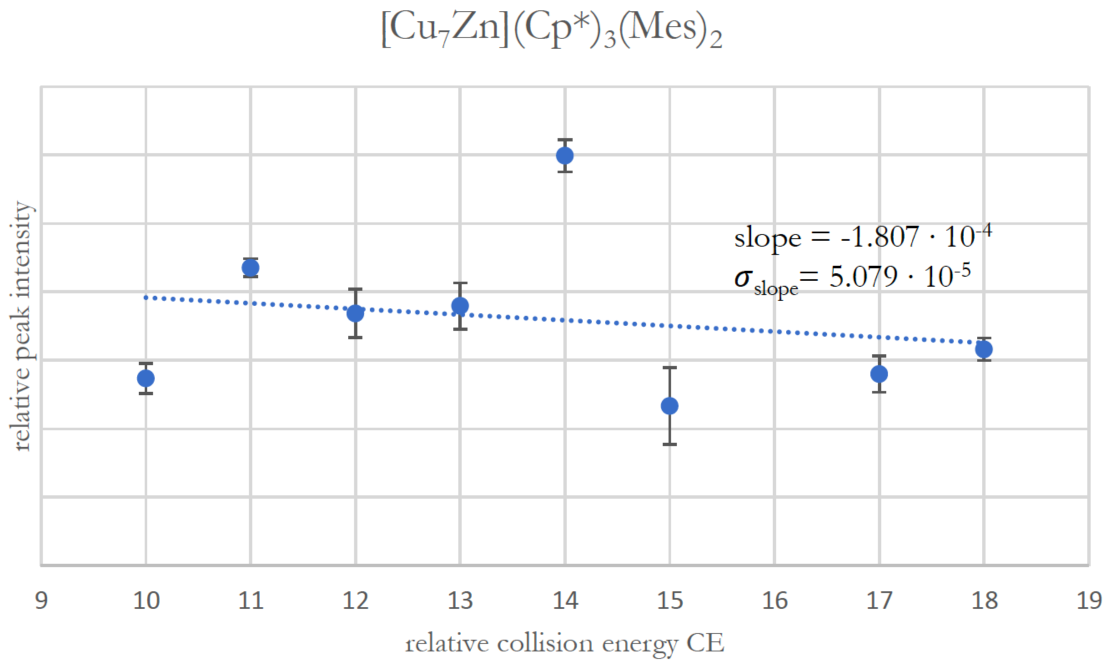

Supplementary Figure 102: CE vs. I plot of the ion $\{[Cu_7Zn](Cp^*)_3(Mes)_2\}^+$. A clear assignment is difficult for this species. However, it is likely to be a molecular ion, as the peak intensities at CE >15 are expected to be even lower than detected. However, it may also be a fragment formed during ionization. The regression was performed on the statistical means of five independent measurements under identical experimental conditions for each CE value. The error bars represent the standard deviations of these measurements.

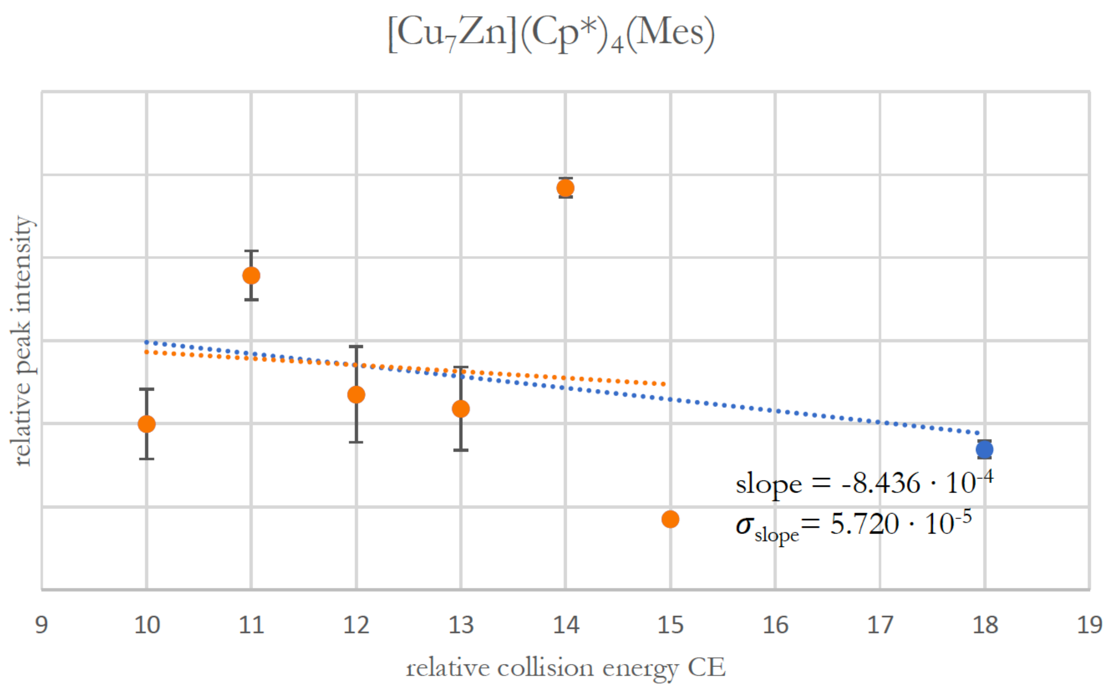

Supplementary Figure 103: CE vs. I plot of the ion {[Cu_7Zn](Cp^*)$_4$(Mes)}$^+$. The species is clearly assigned as a molecular ion according to the CE vs. I plot. However, it may also be a fragment formed during ionization. The regression was performed on the statistical means of five independent measurements under identical experimental conditions for each CE value. The error bars represent the standard deviations of these measurements.

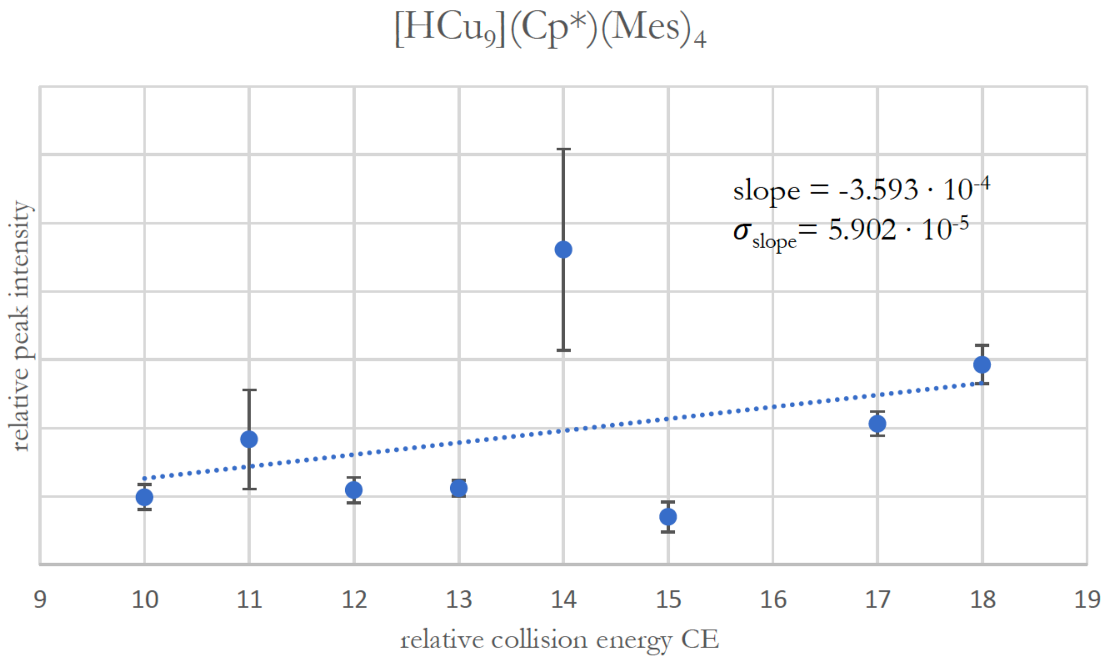

Supplementary Figure 104: CE vs. I plot of the ion {[HCu_9](Cp^*)(Mes)$_4$}$^+$. A clear assignment is not possible for this species due to the bias in integration at CE > 15. The regression was performed on the statistical means of five independent measurements under identical experimental conditions for each CE value. The error bars represent the standard deviations of these measurements.

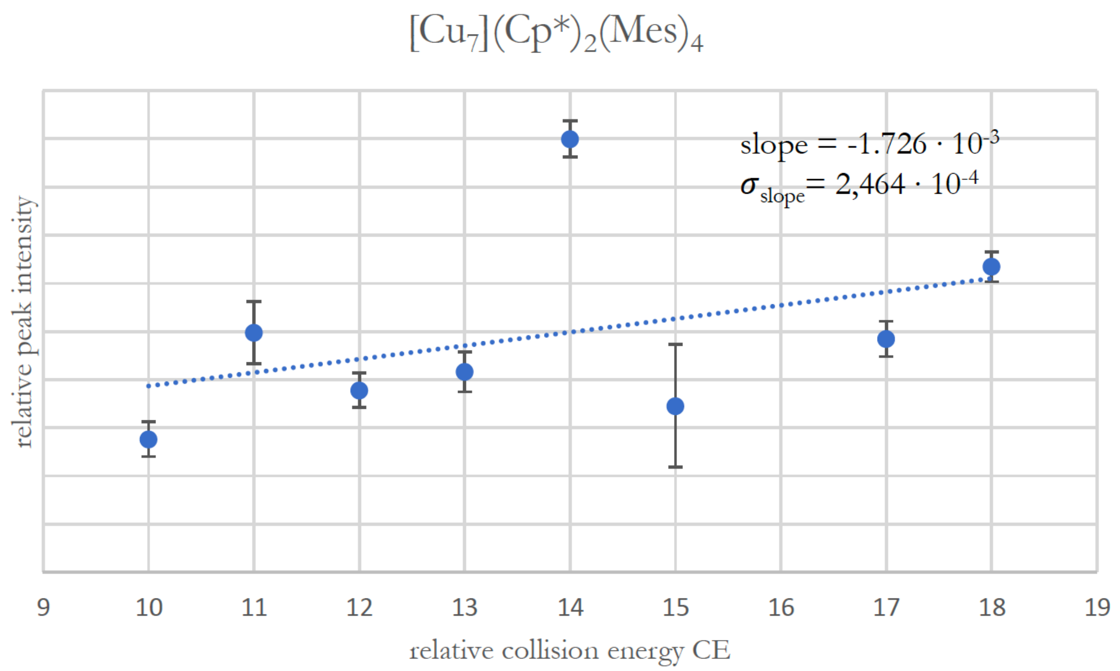

Supplementary Figure 105: CE vs. I plot of the ion {[Cu_7](Cp^*)$_2$(Mes)$_4$}$^+$. A clear assignment is not possible for this species due to the bias in integration at CE > 15. The regression was performed on the statistical means of five independent measurements under identical experimental conditions for each CE value. The error bars represent the standard deviations of these measurements.

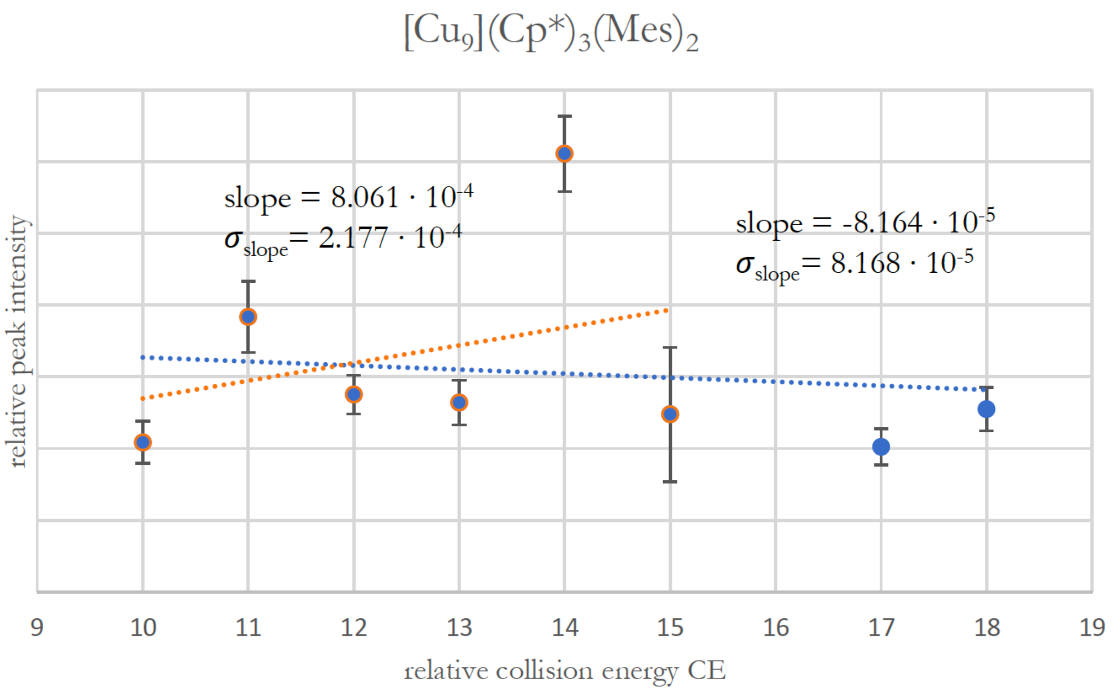

Supplementary Figure 106: CE vs. I plot of the ion {[Cu_9](Cp^*)$_3$(Mes)$_2$}$^+$. A clear assignment is not possible for this species. The regression was performed on the statistical means of five independent measurements under identical experimental conditions for each CE value. The error bars represent the standard deviations of these measurements.

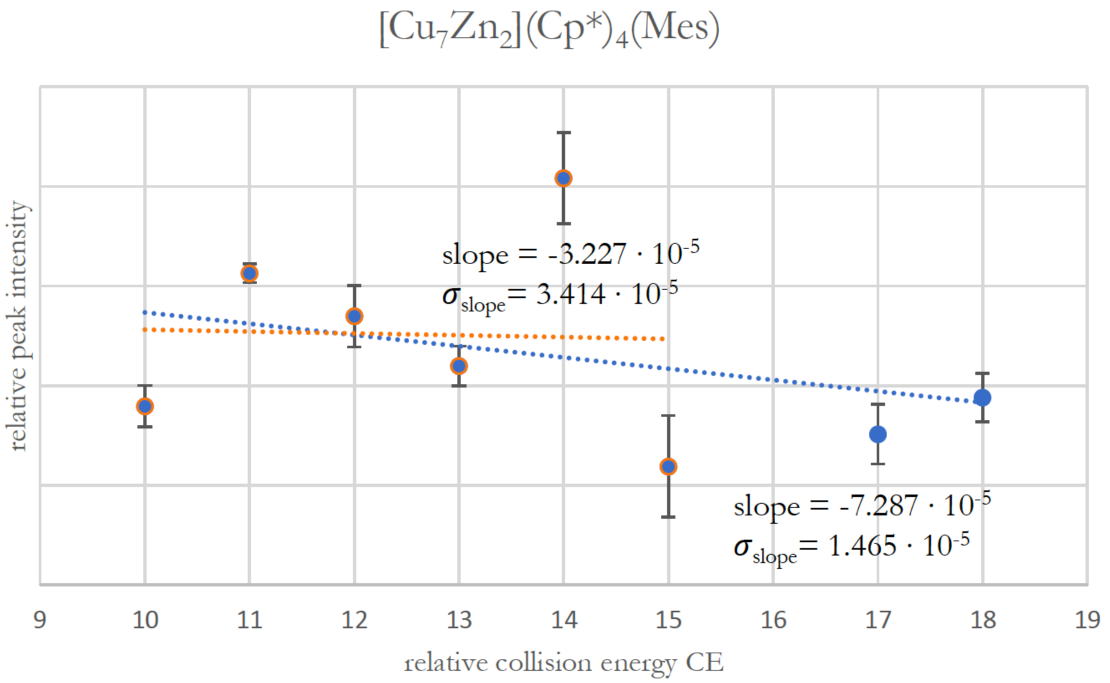

Supplementary Figure 107: CE vs. I plot of the ion $\{[Cu_7Zn_2](Cp^*)_4(Mes)\}^+$. The species can be assigned as molecular ion if considering that a slight increase in peak intensity at CE > 15 is supposed to be caused by the bias in integration. It may however also be a fragment formed during ionization. The regression was performed on the statistical means of five independent measurements under identical experimental conditions for each CE value. The error bars represent the standard deviations of these measurements.

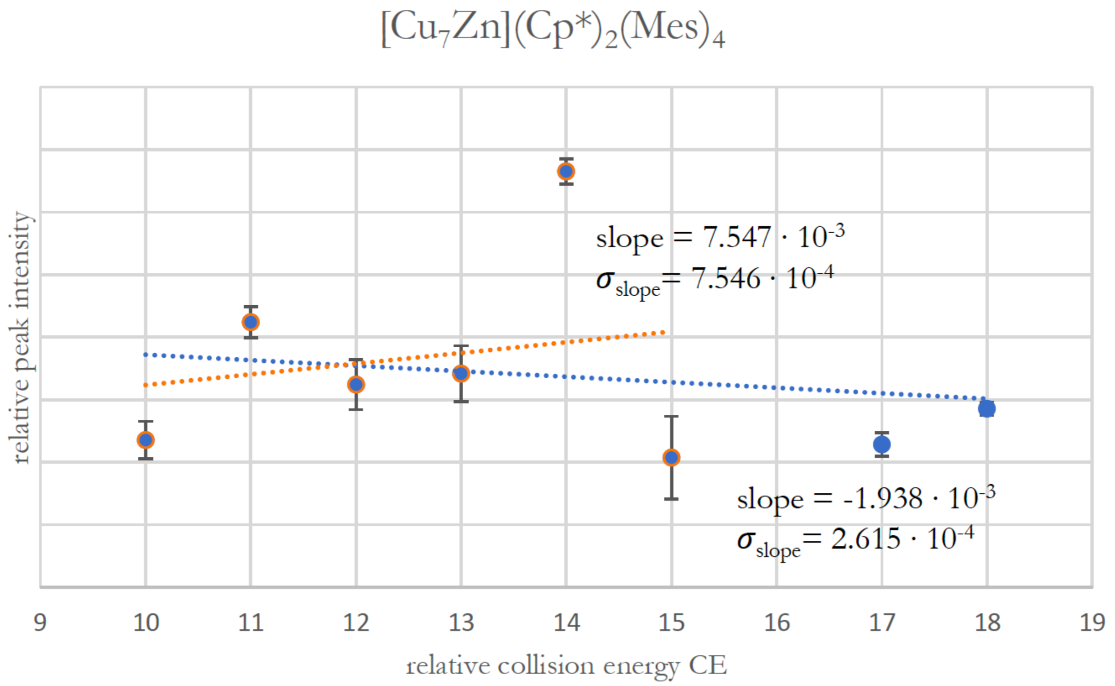

Supplementary Figure 108: CE vs. I plot of the ion $\{[Cu_7Zn](Cp^*)_2(Mes)_4\}^+$. A clear assignment cannot be made for this species, however, it is likely to be molecular ion as the peak intensities at CE >15 are expected to be even lower than detected. However, it may also be a fragment formed during ionization. The regression was performed on the statistical means of five independent measurements under identical experimental conditions for each CE value. The error bars represent the standard deviations of these measurements.

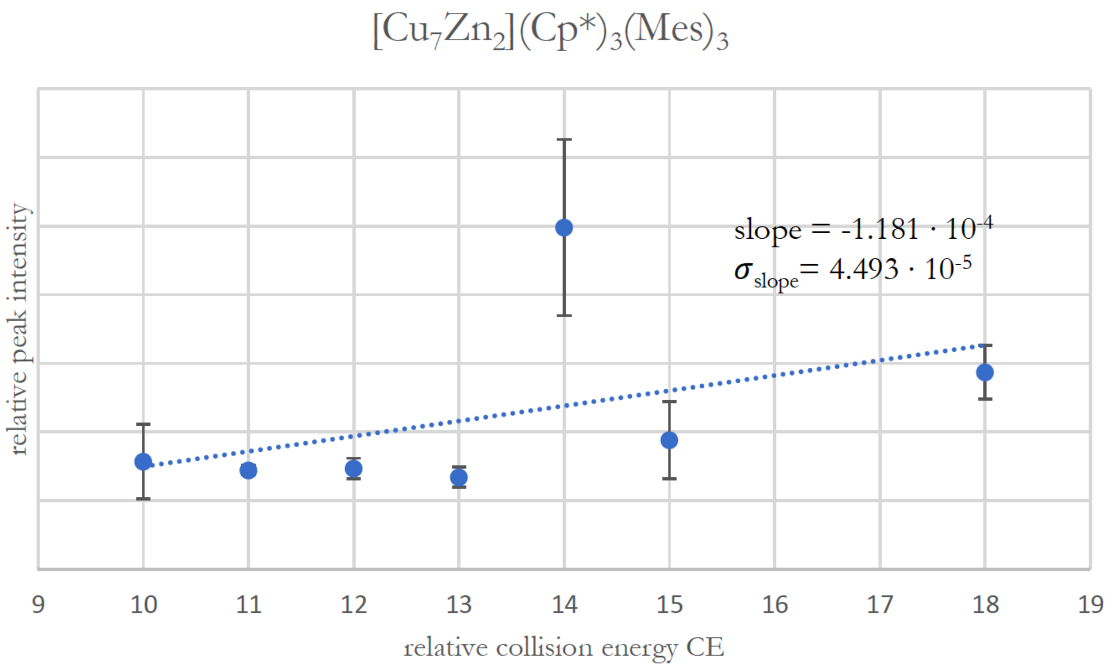

Supplementary Figure 109: CE vs. I plot of the ion $\{[Cu_7Zn_2](Cp^*)_3(Mes)_3\}^+$. A clear assignment cannot be made for this species due to the bias in integration at CE > 15. The regression was performed on the statistical means of five independent measurements under identical experimental conditions for each CE value. The error bars represent the standard deviations of these measurements.

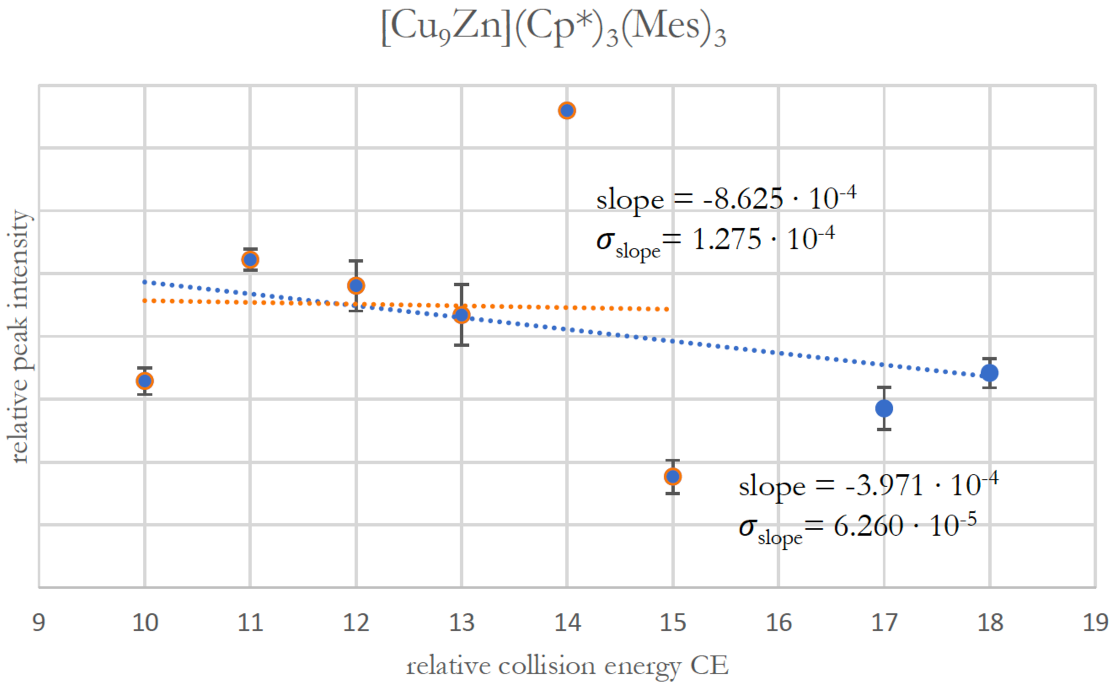

Supplementary Figure 110: CE vs. I plot of the ion $\{[Cu_9Zn](Cp^*)_3(Mes)_3\}^+$. The species can be assigned as molecular ion if considering that a slight increase in peak intensity at CE > 15 is supposed to be caused by the bias in integration. The regression was performed on the statistical means of five independent measurements under identical experimental conditions for each CE value. The error bars represent the standard deviations of these measurements.

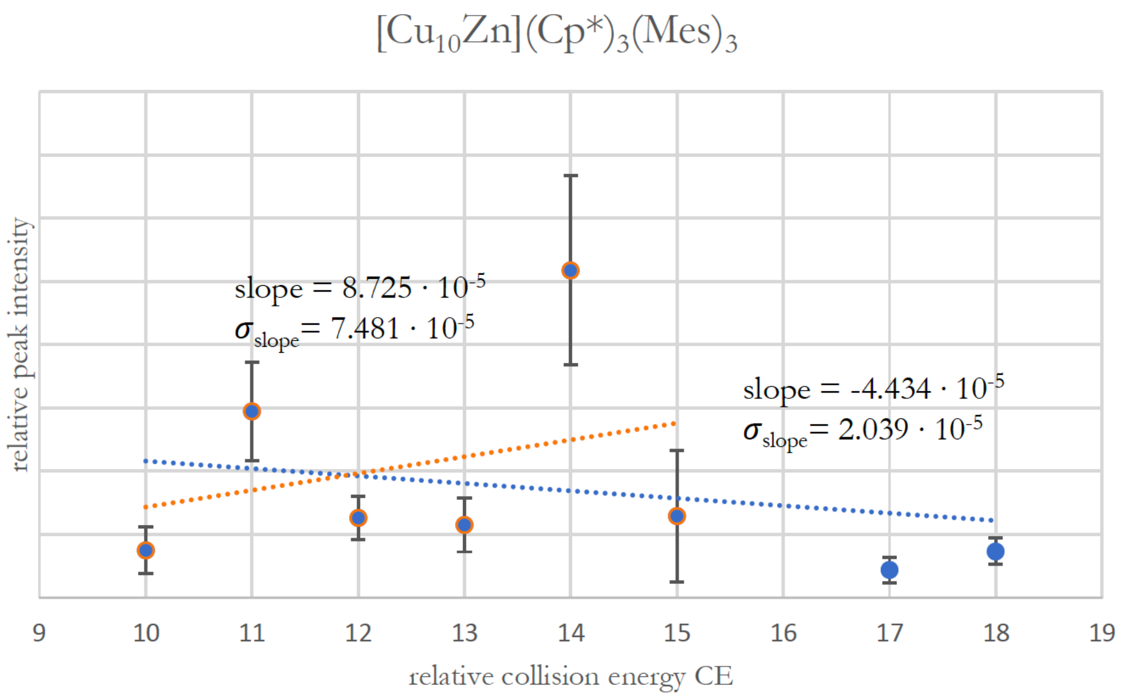

Supplementary Figure 111: CE vs. I plot of the ion {[$Cu_{10}Zn$](Cp^*)$_3$(Mes)$_3$}$^+$. The species is assigned as molecular ion. Due to the bias in integration, the intensity values at CE = 17 and 18 are supposed to be actually even lower than detected. The regression was performed on the statistical means of five independent measurements under identical experimental conditions for each CE value. The error bars represent the standard deviations of these measurements.

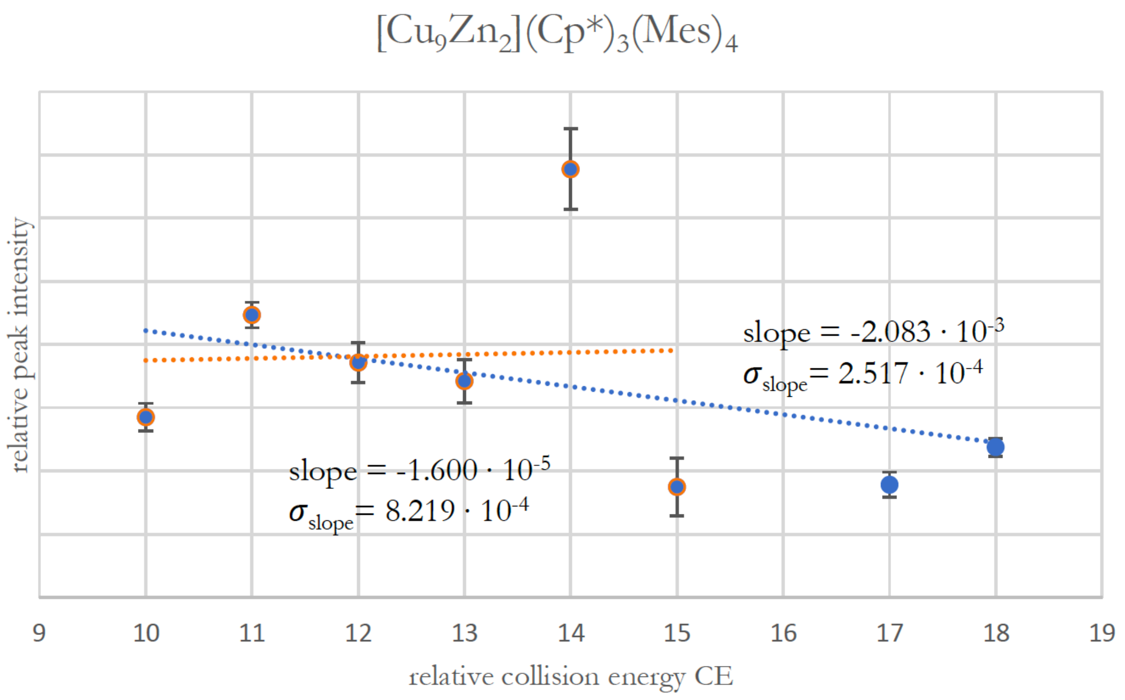

Supplementary Figure 112: CE vs. I plot of the ion {[Cu_9Zn_2](Cp^*)$_3$(Mes)$_4$}$^+$. The species is assigned as molecular ion. Due to the bias in integration, the intensity values at CE = 17 and 18 are supposed to be even lower than detected. The regression was performed on the statistical means of five independent measurements under identical experimental conditions for each CE value. The error bars represent the standard deviations of these measurements.

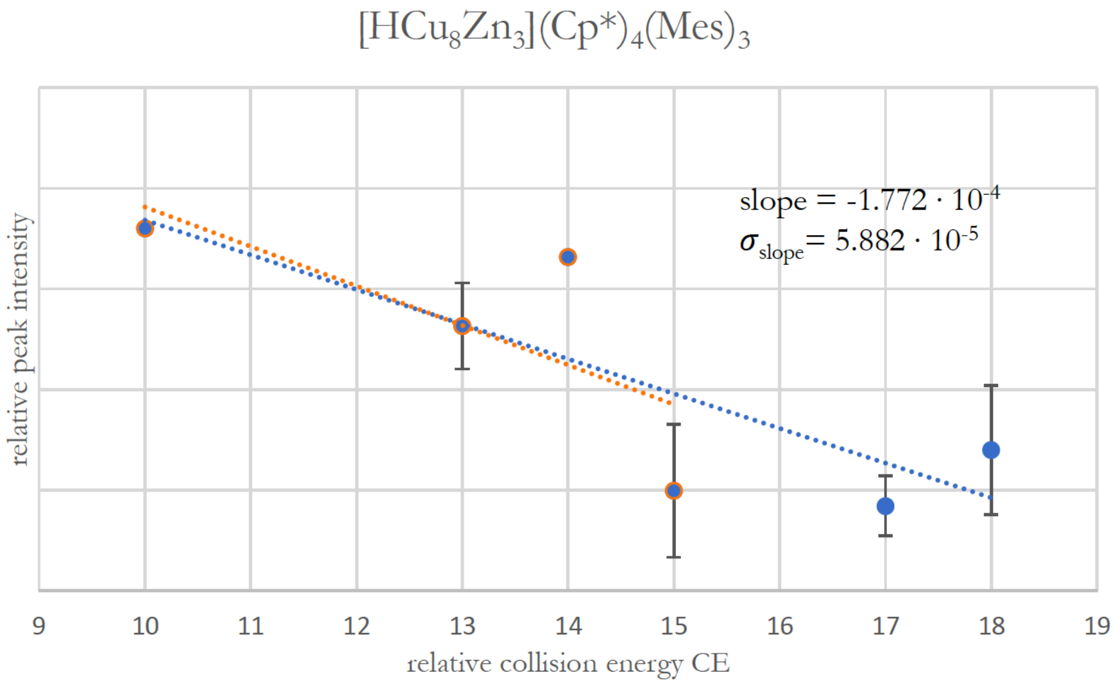

Supplementary Figure 113: CE vs. I plot of the ion $\{[HCu_8Zn_3](Cp^*)_4(Mes)_3\}^+$(**E**$^+$). The species is assigned as molecular ion. Due to the bias in integration, the intensity values at CE = 17 and 18 are supposed to be actually even lower than detected. The regression was performed on the statistical means of five independent measurements under identical experimental conditions for each CE value. The error bars represent the standard deviations of these measurements.

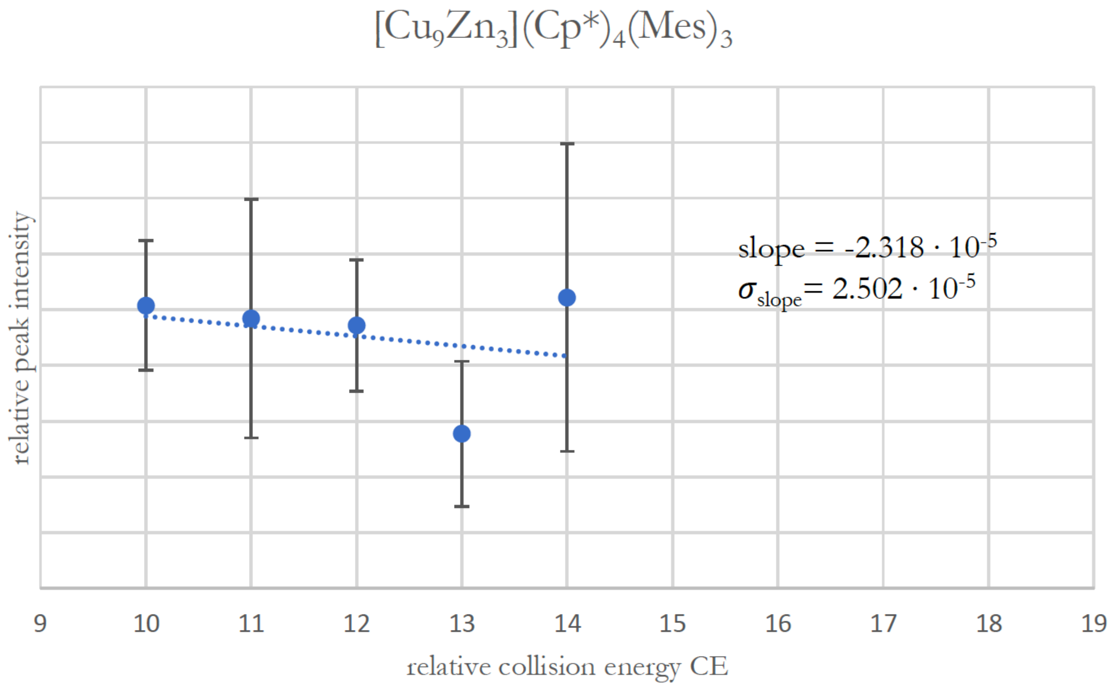

Supplementary Figure 114: CE vs. I plot of the ion $\{[Cu_9Zn_3](Cp^*)_4(Mes)_3\}^+$. The species is assigned as a molecular ion. The regression was performed on the statistical means of five independent measurements under identical experimental conditions for each CE value. The error bars represent the standard deviations of these measurements.

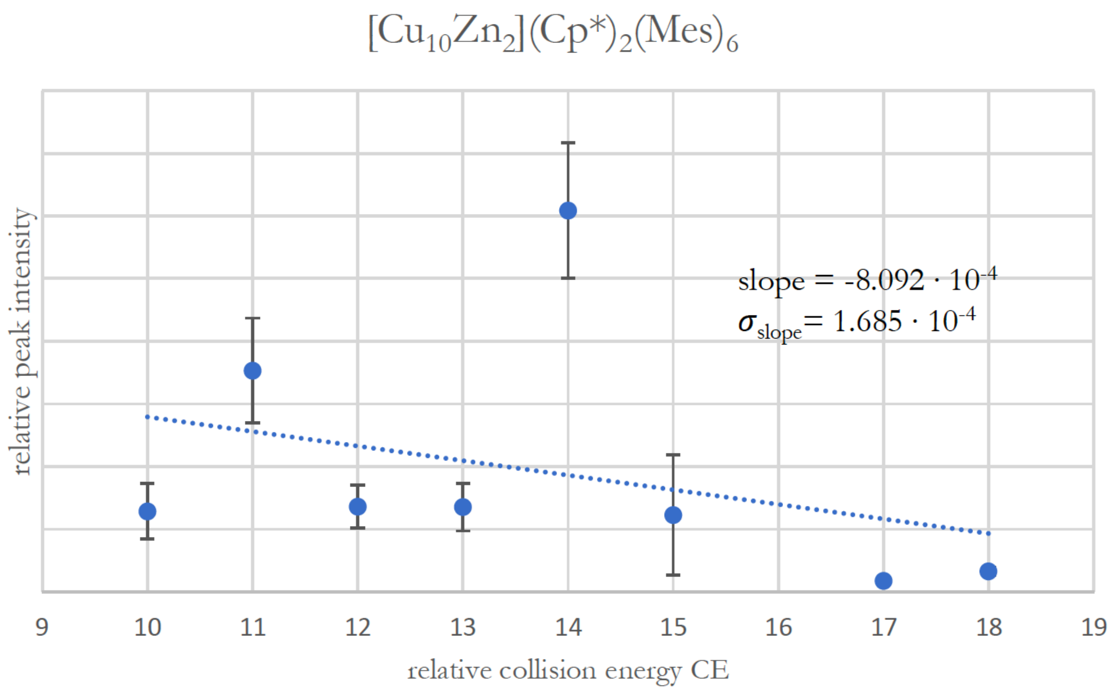

Supplementary Figure 115: CE vs. I plot of the ion $\{[Cu_{10}Zn_2](Cp^*)_2(Mes)_6\}^+$. The species is assigned as molecular ion and can also be isolated. The regression was performed on the statistical means of five independent measurements under identical experimental conditions for each CE value. The error bars represent the standard deviations of these measurements.

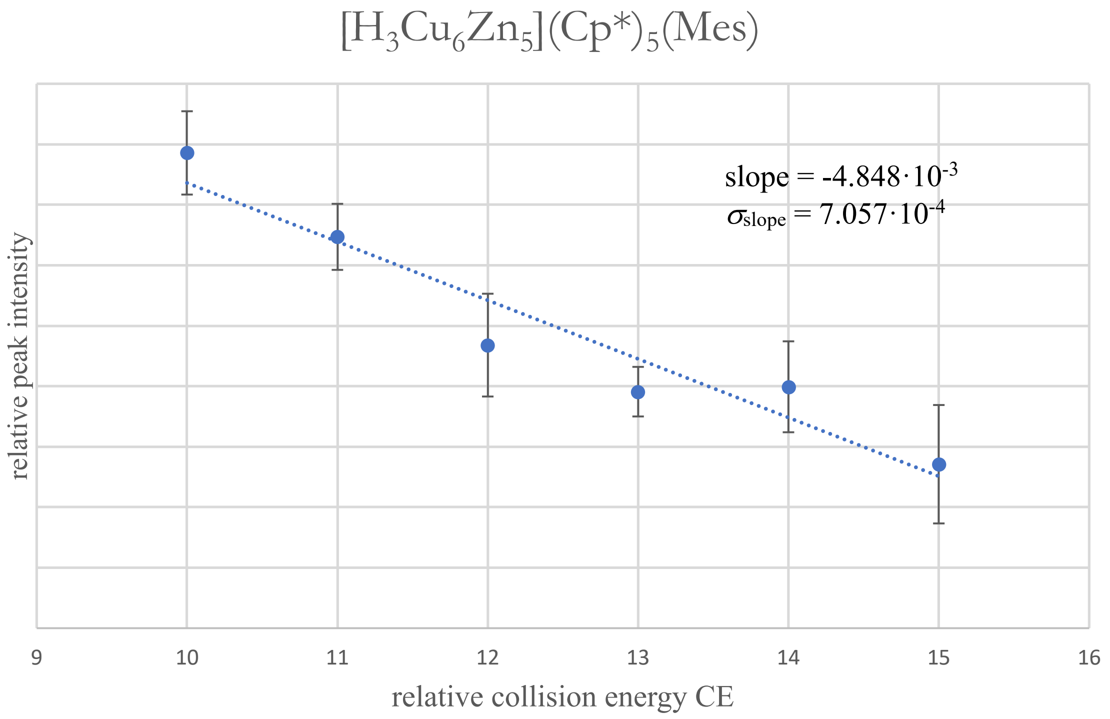

Supplementary Figure 116: CE vs. I plot of the ion $\{[H_3Cu_6Zn_5](Cp^*)_5(Mes)\}^+$(**D**$^+$). The species is assigned as molecular ion. The regression was performed on the statistical means of five independent measurements under identical experimental conditions for each CE value. The error bars represent the standard deviations of these measurements.

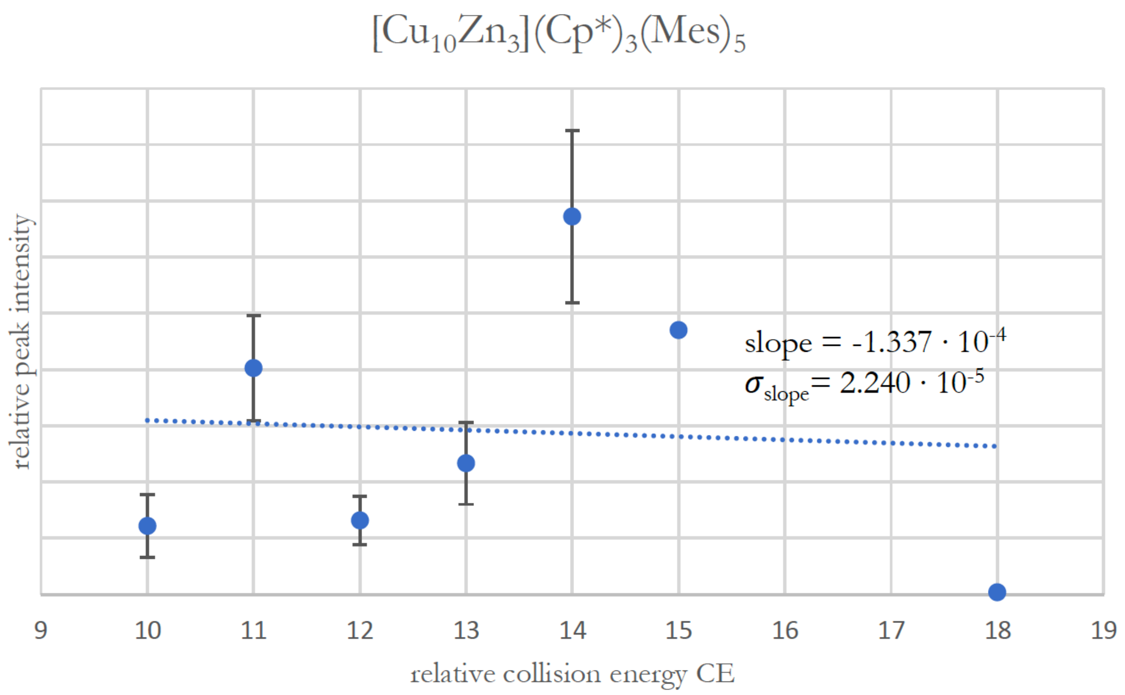

Supplementary Figure 117: CE vs. I plot of the ion $\{[Cu_{10}Zn_3](Cp^*)_3(Mes)_5\}^+$. A clear assignment cannot be made for this species. The regression was performed on the statistical means of five independent measurements under identical experimental conditions for each CE value. The error bars represent the standard deviations of these measurements.

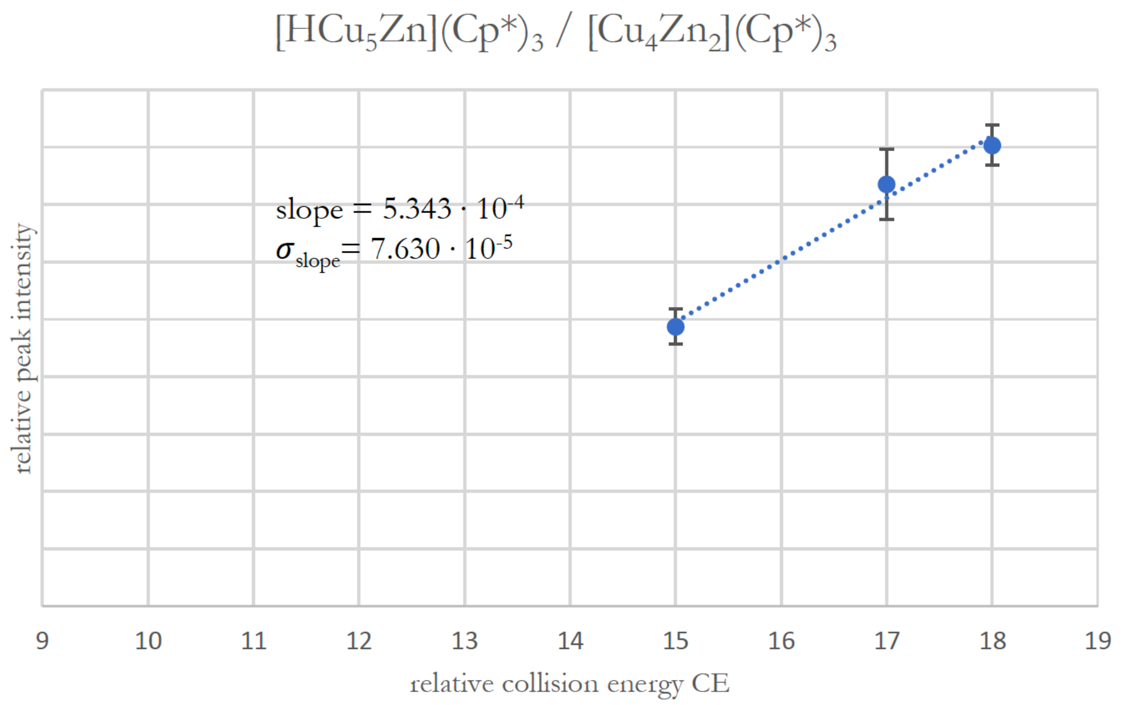

Supplementary Figure 118: CE vs. I plot of the ion $\{[HCu_5Zn](Cp^*)_3\}^+$ and $[Cu_4Zn_2](Cp^*)_3$. Both ions were identified by labeling experiments with ^{68}Zn. In the unlabeled spectra however, their peaks are overlapping. Both ions can be assigned as fragments according to the CE vs. I plots. The regression was performed on the statistical means of five independent measurements under identical experimental conditions for each CE value. The error bars represent the standard deviations of these measurements.

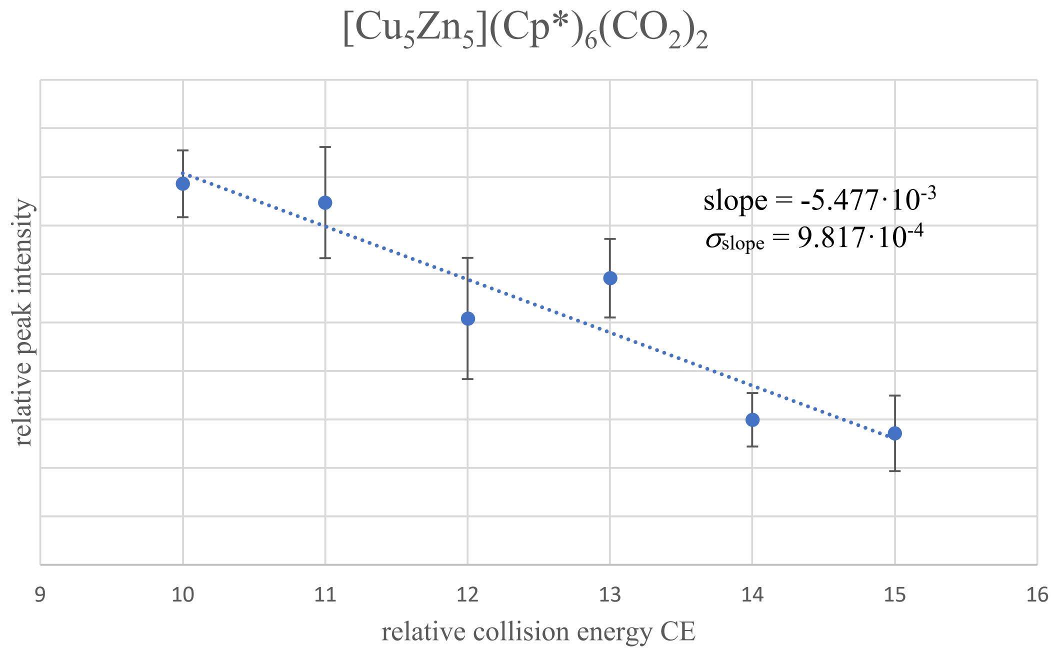

Supplementary Figure 119: CE vs. I plot of the ion $\{[Cu_5Zn_5](Cp^*)_6(CO_2)_2\}^+$ (**X**$^+$). The species is assigned as molecular ion. The regression was performed on the statistical means of five independent measurements under identical experimental conditions for each CE value. The error bars represent the standard deviations of these measurements.

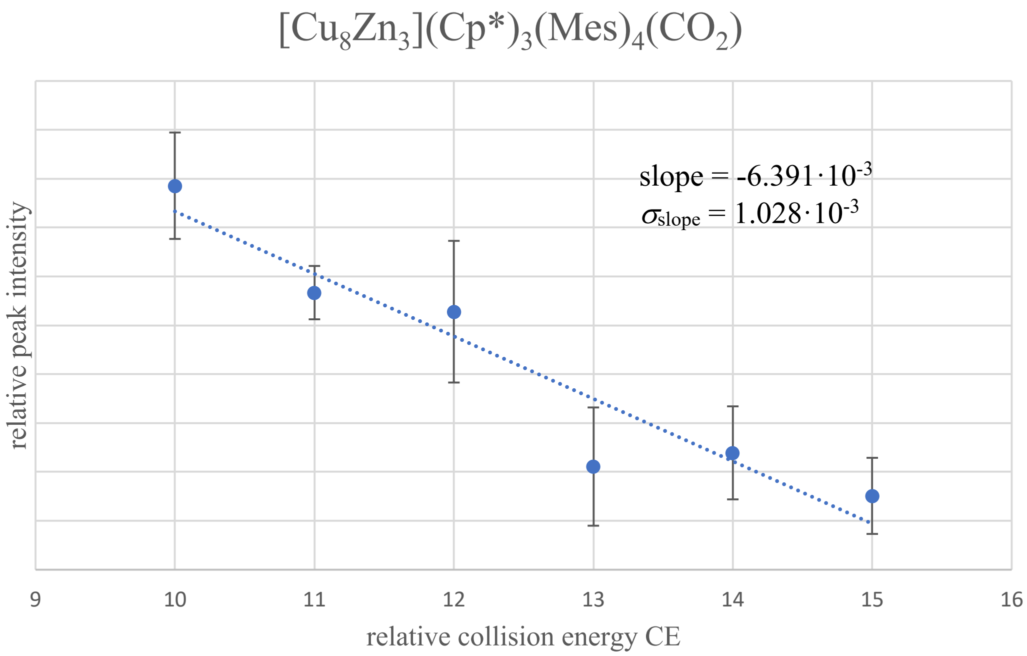

Supplementary Figure 120: CE vs. I plot of the ion $\{[Cu_8Zn_3](Cp^*)_3(Mes)_4(CO_2)\}^+$ (**Y**$^+$). The species is assigned as molecular ion. The regression was performed on the statistical means of five independent measurements under identical experimental conditions for each CE value. The error bars represent the standard deviations of these measurements.

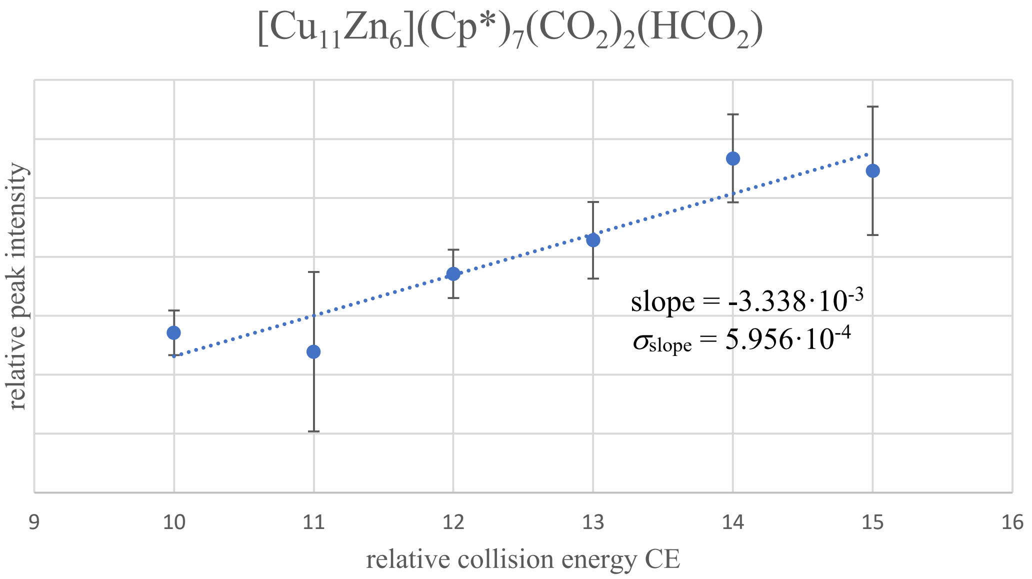

Supplementary Figure 121: CE vs. I plot of the ion $\{[Cu_{11}Zn_6](Cp^*)_7(CO_2)_2(HCO_2)\}^+$. The species is assigned as fragment ion. The regression was performed on the statistical means of five independent measurements under identical experimental conditions for each CE value. The error bars represent the standard deviations of these measurements.

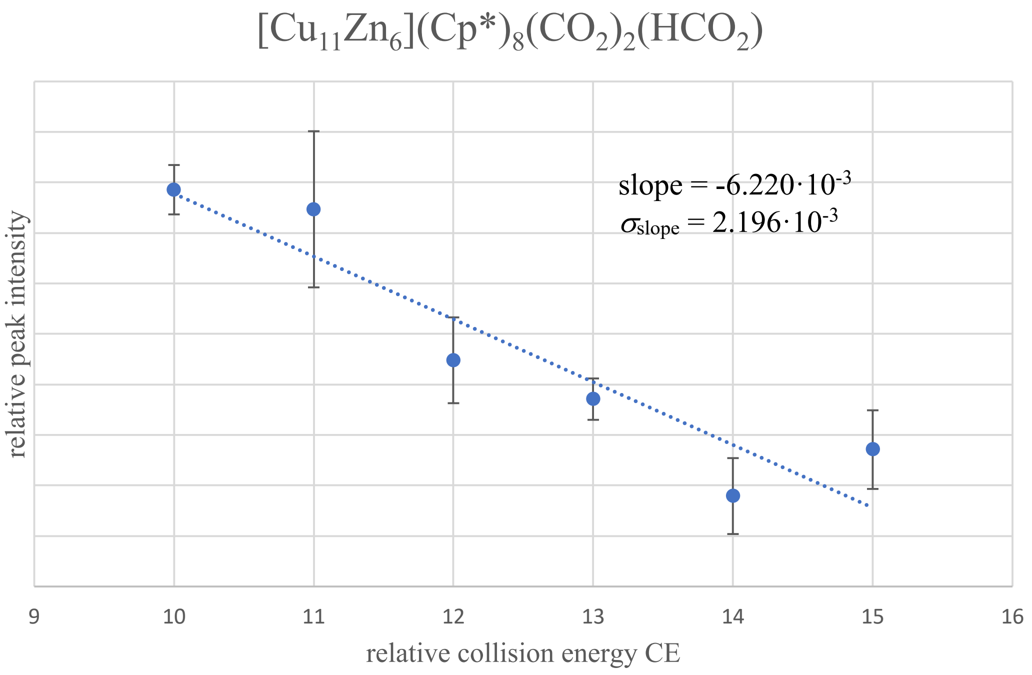

Supplementary Figure 122: CE vs. I plot of the ion $\{[Cu_{11}Zn_6](Cp^*)_8(CO_2)_2(HCO_2)\}^+$ (**Z**$^+$). The species is assigned as molecular ion. The regression was performed on the statistical means of five independent measurements under identical experimental conditions for each CE value. The error bars represent the standard deviations of these measurements.

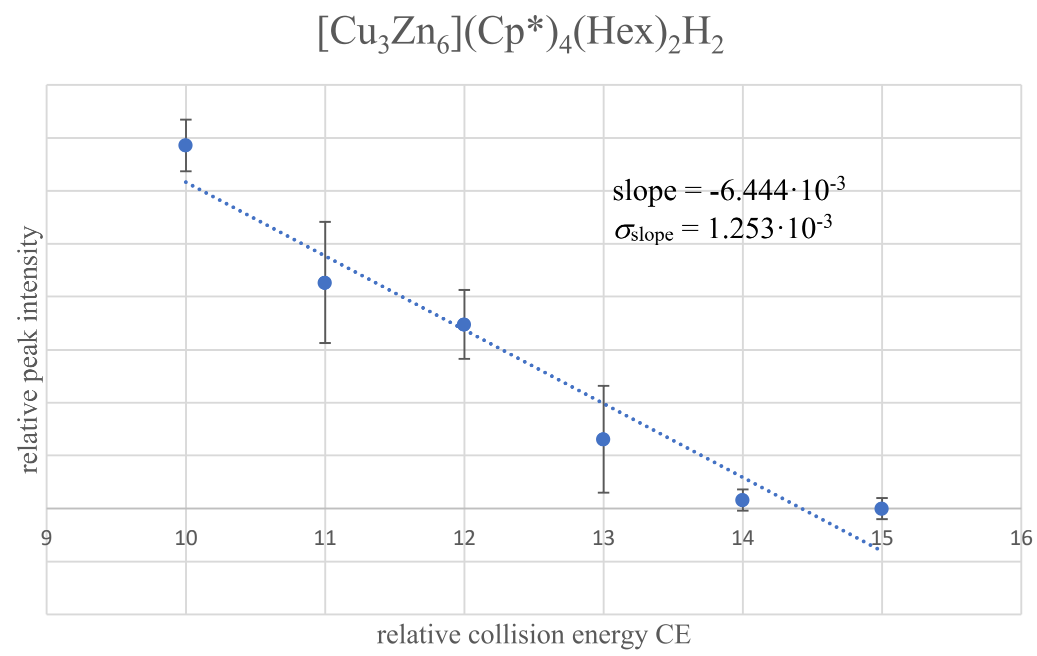

Supplementary Figure 123: CE vs. I plot of the ion $\{[Cu_3Zn_6](Cp^*)_4(Hex)_2H_2\}^+$. The species is assigned as molecular ion. The regression was performed on the statistical means of five independent measurements under identical experimental conditions for each CE value. The error bars represent the standard deviations of these measurements.

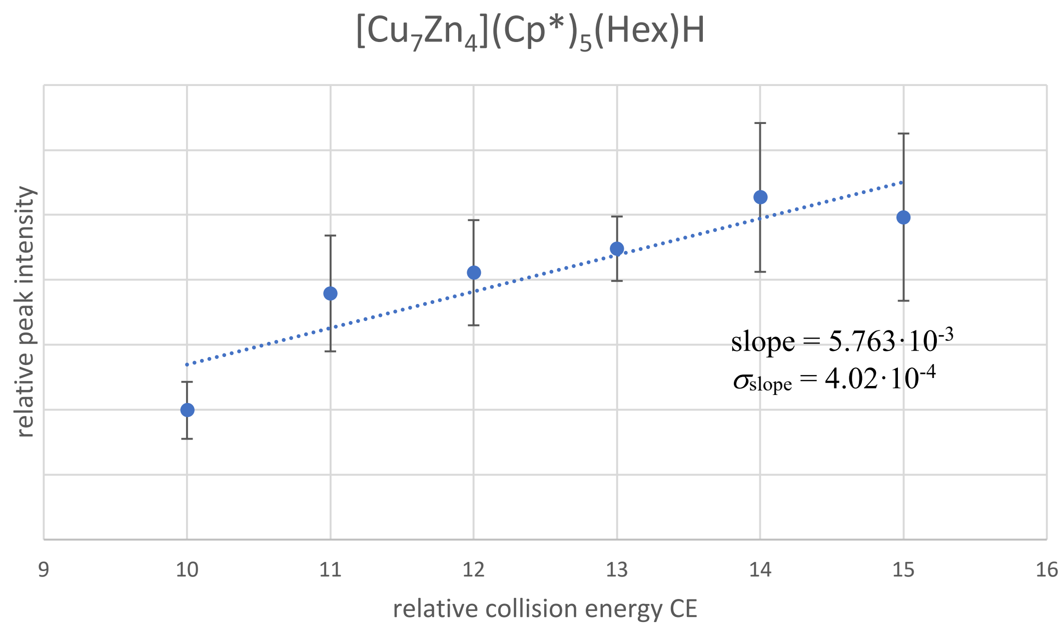

Supplementary Figure 124: CE vs. I plot of the ion $\{[Cu_7Zn_4](Cp^*)_5(Hex)H\}^+$. The species is assigned as fragment ion. The regression was performed on the statistical means of five independent measurements under identical experimental conditions for each CE value. The error bars represent the standard deviations of these measurements.

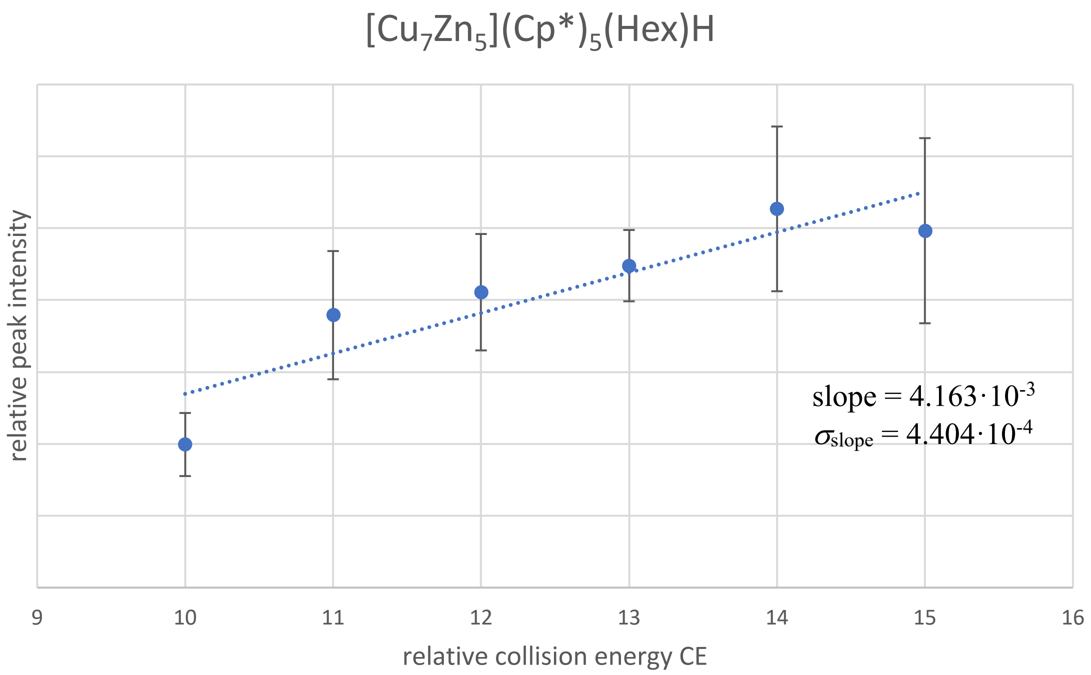

Supplementary Figure 125: CE vs. I plot of the ion $\{[Cu_7Zn_5](Cp^*)_5(Hex)H\}^+$. The species is assigned as fragment ion. The regression was performed on the statistical means of five independent measurements under identical experimental conditions for each CE value. The error bars represent the standard deviations of these measurements.

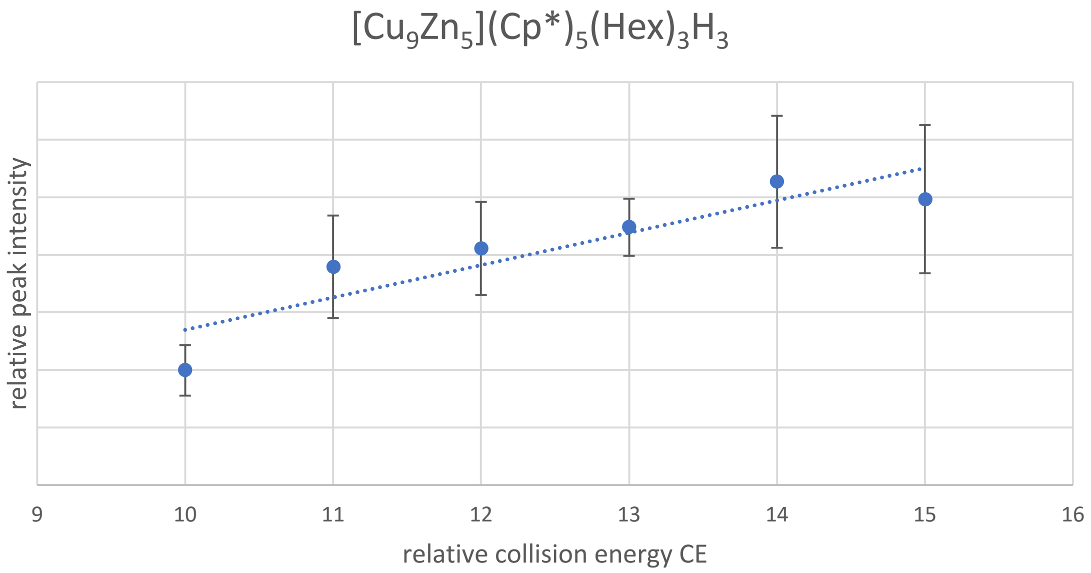

Supplementary Figure 126: CE vs. I plot of the ion $\{[Cu_9Zn_5](Cp^*)_5(Hex)_3H_3\}^+$. The species is assigned as fragment ion, e.g. $[W^+ - ZnZnCp^*]$. The regression was performed on the statistical means of five independent measurements under identical experimental conditions for each CE value. The error bars represent the standard deviations of these measurements.

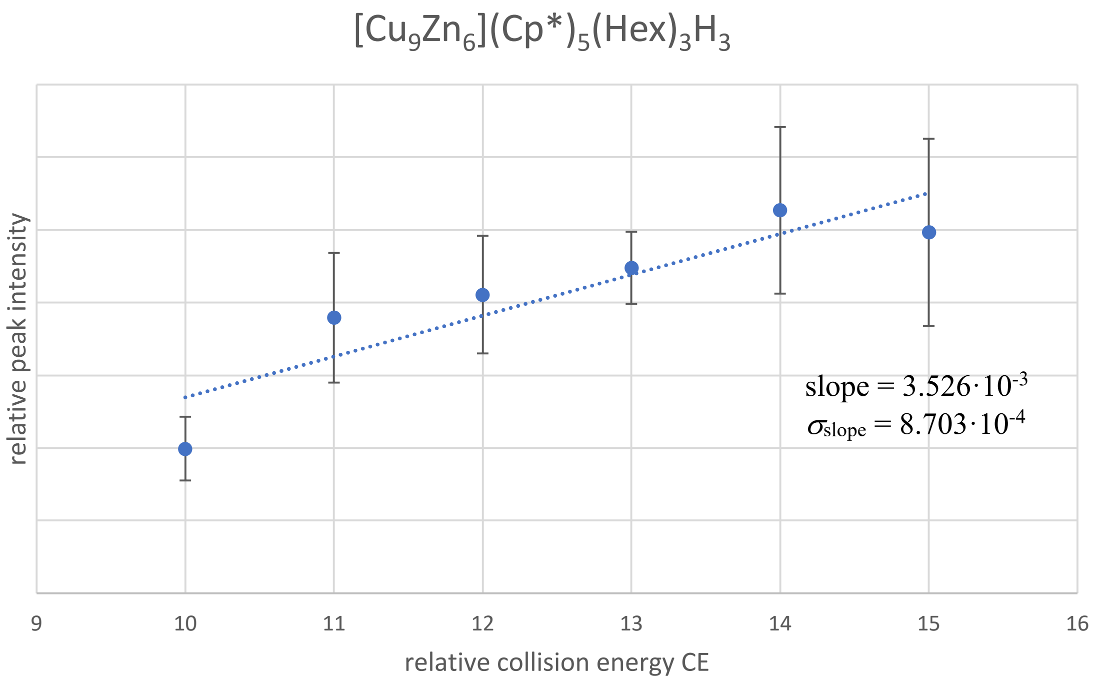

Supplementary Figure 127: CE vs. I plot of the ion $\{[Cu_9Zn_6](Cp^*)_5(Hex)_3H_3\}^+$. The species is assigned as fragment ion, i.e. $[W^+ - ZnCp^*]$. The regression was performed on the statistical means of five independent measurements under identical experimental conditions for each CE value. The error bars represent the standard deviations of these measurements.

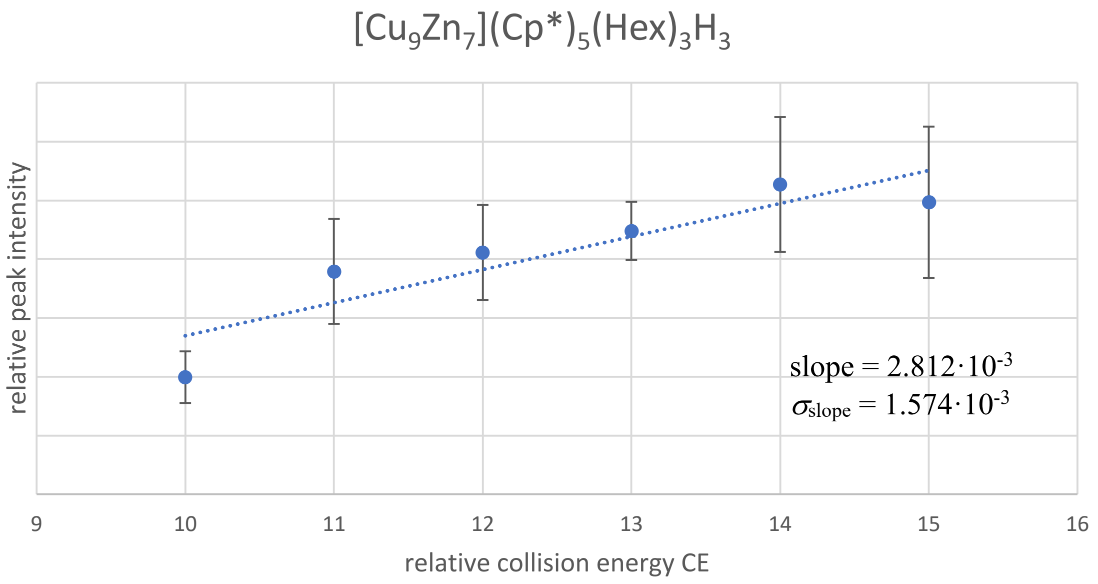

Supplementary Figure 128: CE vs. I plot of the ion $\{[Cu_9Zn_7](Cp^*)_5(Hex)_3H_3\}^+$. The species is assigned as fragment ion, i.e. $[W^+ - Cp^*]$. The regression was performed on the statistical means of five independent measurements under identical experimental conditions for each CE value. The error bars represent the standard deviations of these measurements.

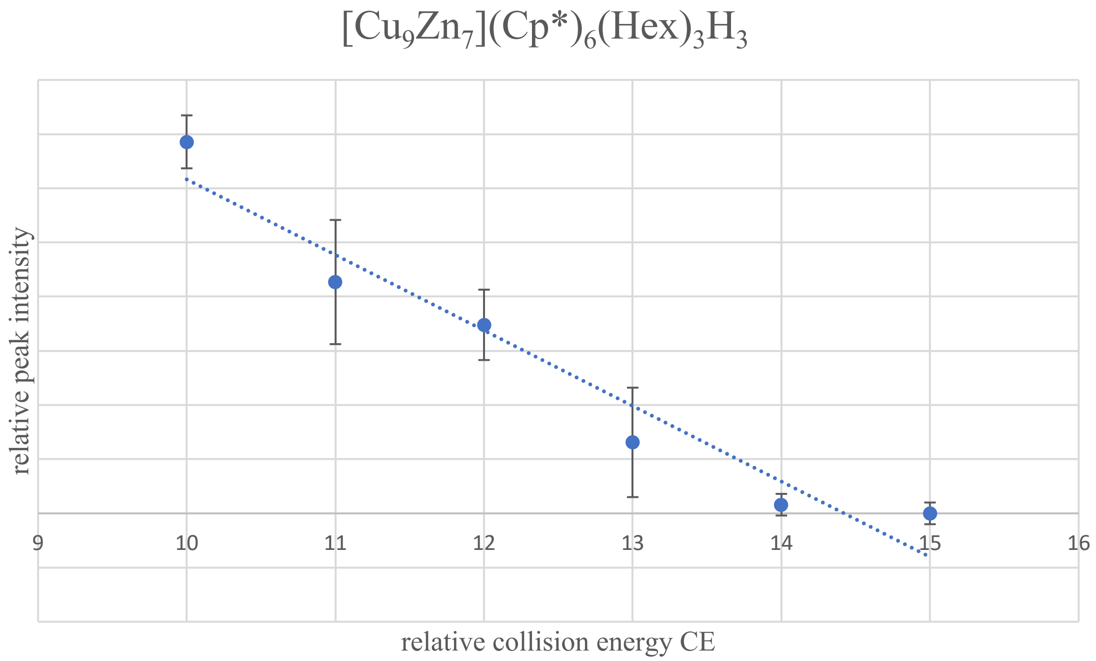

Supplementary Figure 129: CE vs. I plot of the ion $\{[Cu_9Zn_7](Cp^*)_6(Hex)_3H_3\}^+$ (**W**$^+$). The species is assigned as molecular ion. The regression was performed on the statistical means of five independent measurements under identical experimental conditions for each CE value. The error bars represent the standard deviations of these measurements.

1.1.2. NMR Spectra

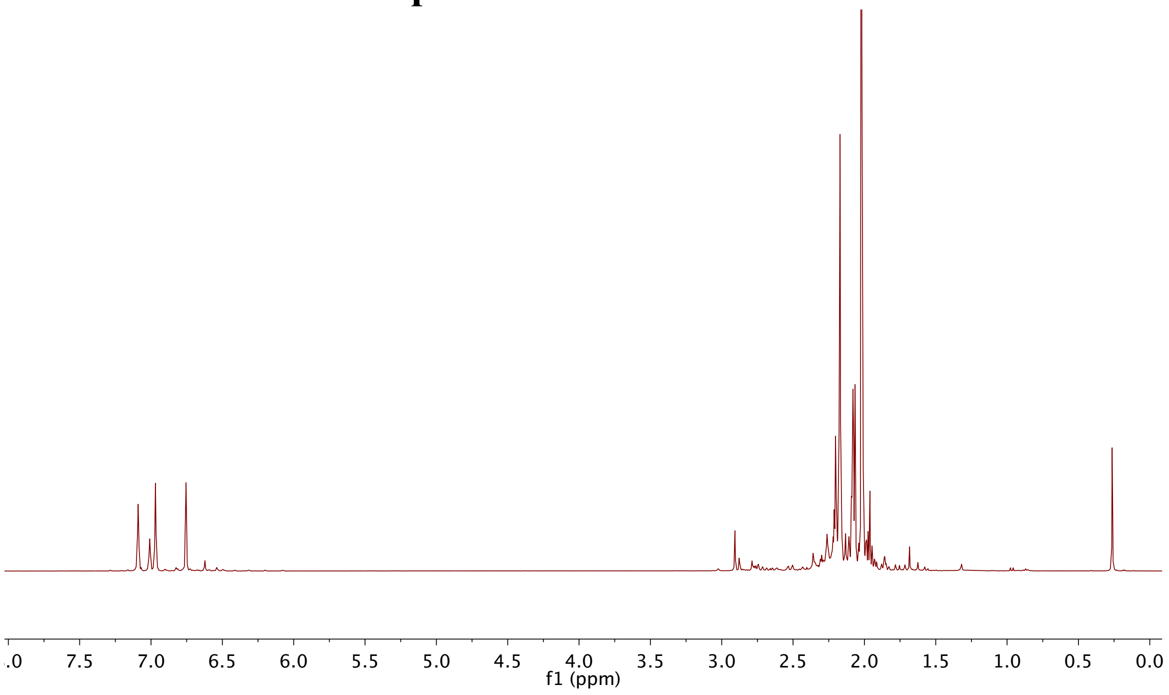

Supplementary Figure 130: ^{1}H NMR spectrum of the Cu/Zn library {**1**} (toluene-d_8).

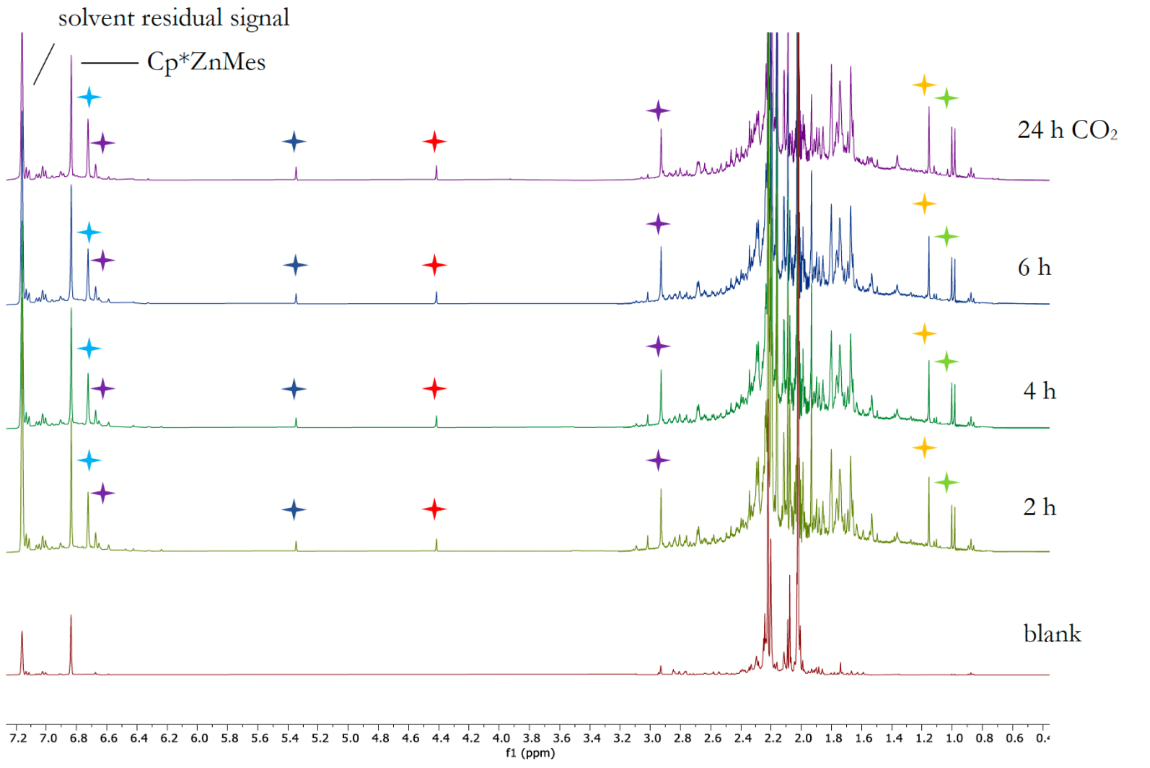

Supplementary Figure 131: Time dependent evolution in situ ^{1}H NMR of the reaction of the Cu/Zn library {**1**} with 1 bar CO_2 at room temperature (benzene-d_6) {**2**}. ✦ Fulvene ✦ Fulvalene ✦ Cp*H ✦ Mesitylene ✦ Dihydrogen ✦ [Cu_5]Mes_5.

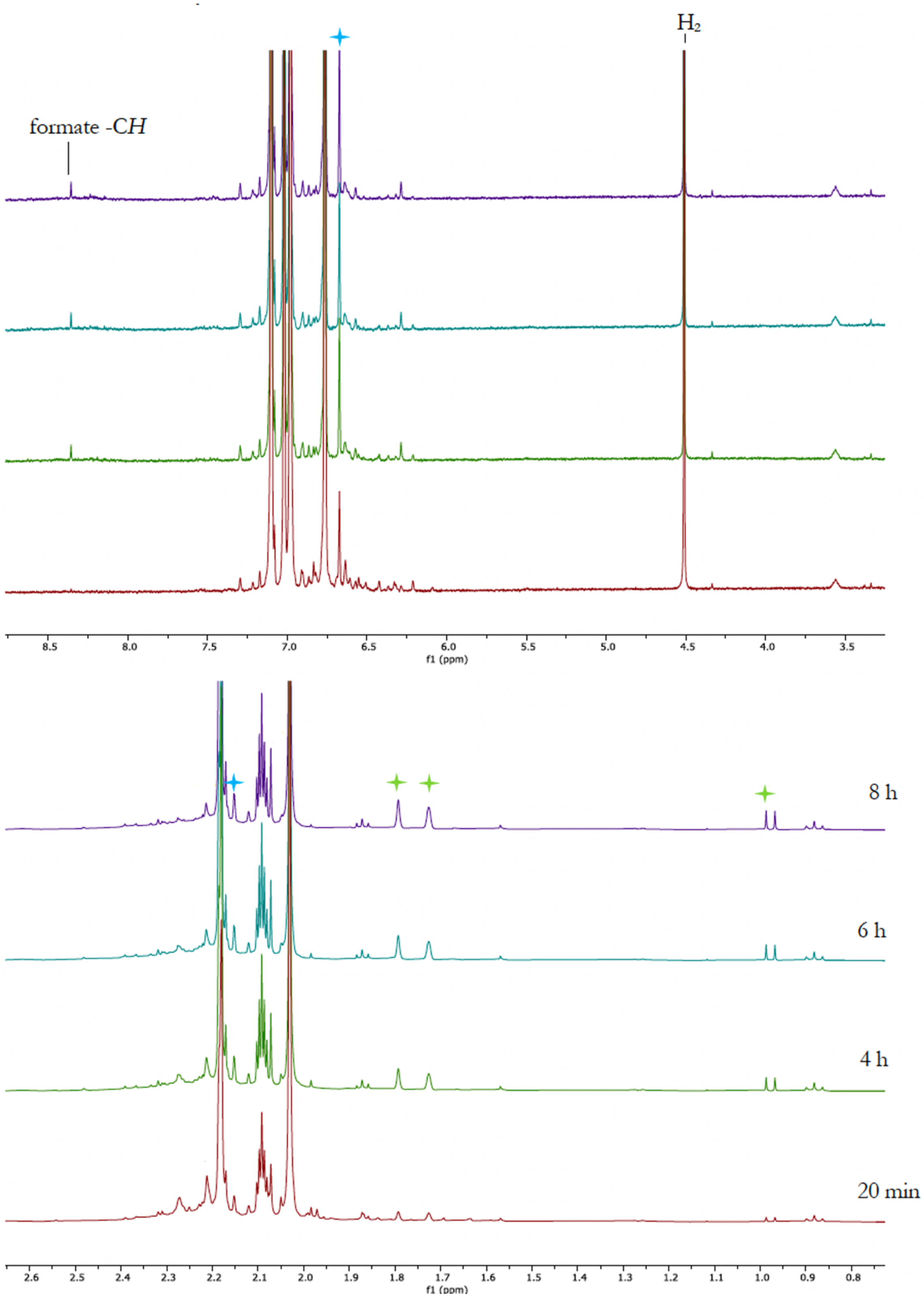

Supplementary Figure 132: Time dependent evolution in situ ^{1}H NMR of the reaction of the Cu/Zn library {**2**} with 2 bar H_2 at room temperature after reaction with CO_2 (toluene-d_8) {**3**}. The formation of formate is observed after four hours already. ✦Mesitylene ✦ Cp*H.

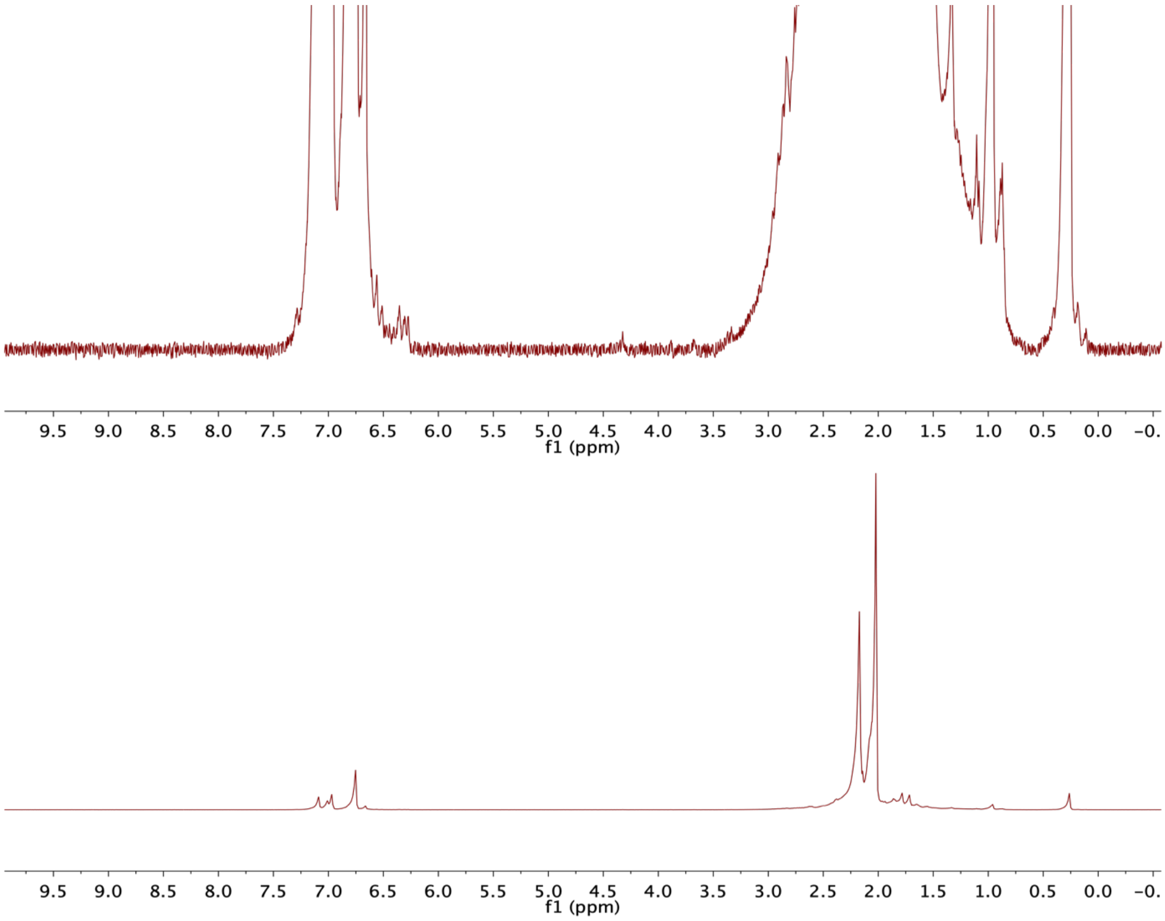

Supplementary Figure 133: Time dependent evolution in situ ^{1}H NMR of the reaction of the Cu/Zn library {**1**} with 2 bar D_2 at room temperature after reaction with CO_2 (toluene-d_8) {**3**}. No deuterated formate is observed, proving its formation from the dihydrogen gas applied to the library.

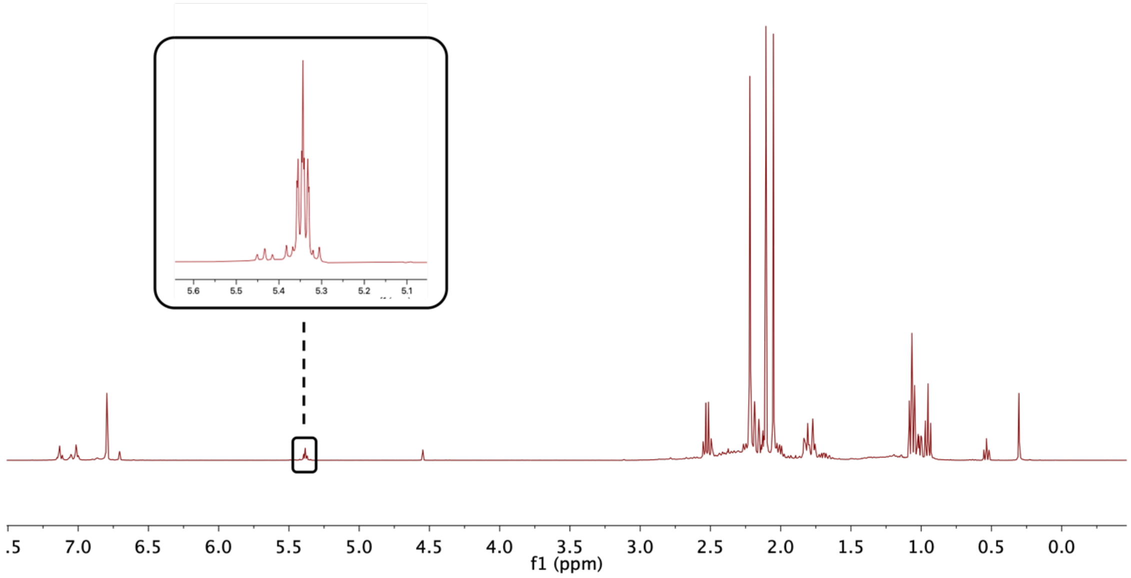

Supplementary Figure 134: ^{1}H NMR spectrum of the Cu/Zn library {**4**} (toluene-d_8) showing the formation of 3-hexene.

1.1.3. FT-IR Spectra

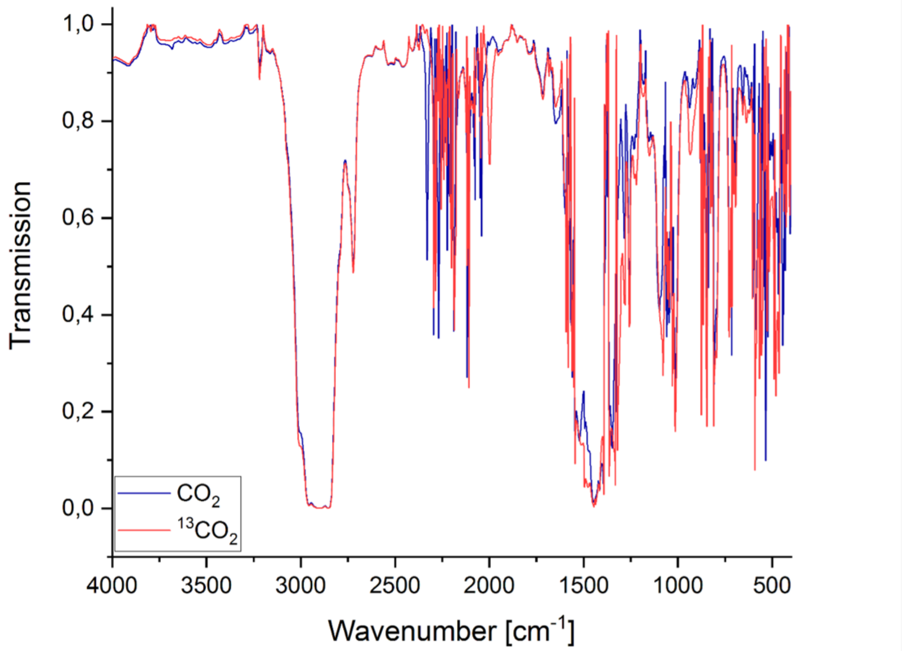

Supplementary Figure 135: Stacked FT-IR spectra of the Cu/Zn library {**2**} after reaction with 1 bar CO_2 at room temperature. No carbonyl vibration is observed, showing that the carbon dioxide is not split at the CO_2 bearing clusters.

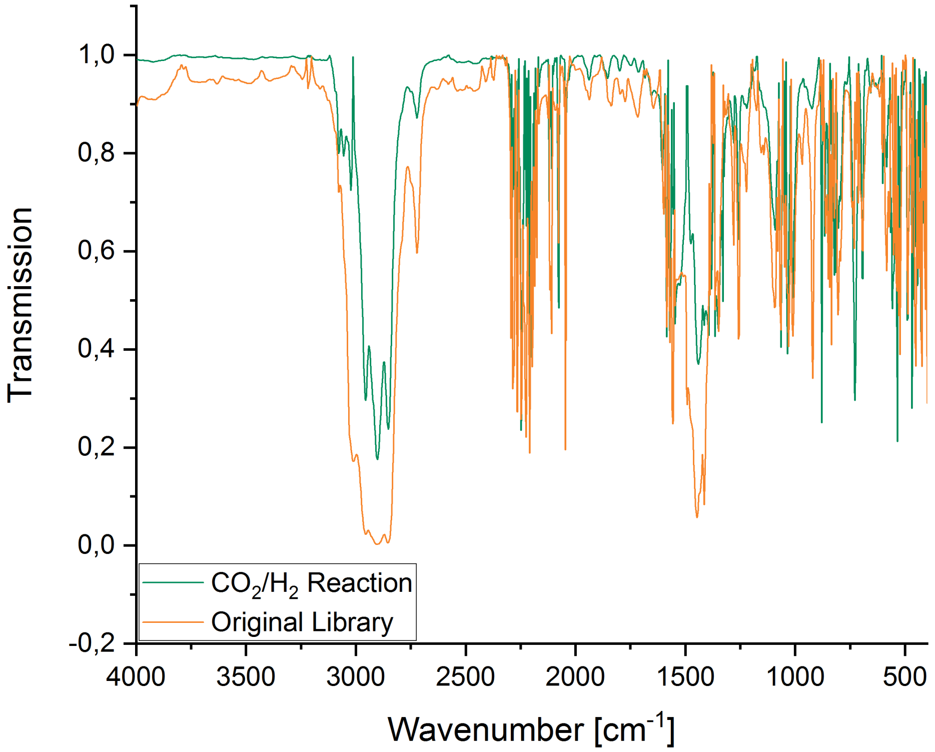

Supplementary Figure 136: Stacked FT-IR spectra of the original Cu/Zn library {**1**} (orange) and of the Cu/Zn library {**3**} after reaction with CO_2 and H_2 (green).

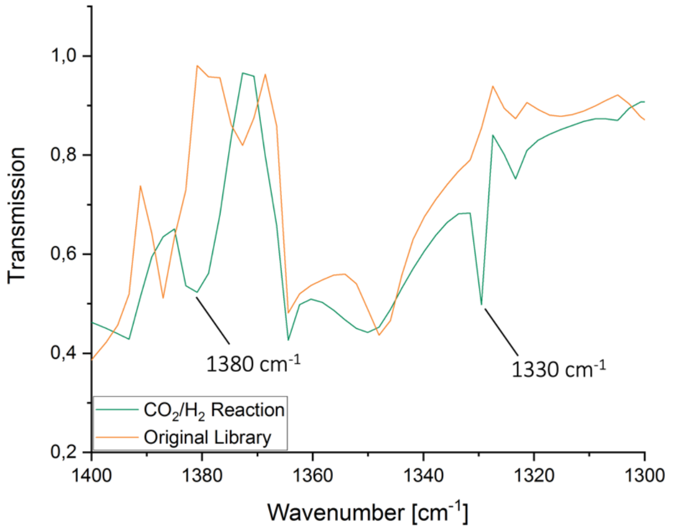

Supplementary Figure 137: Cut-out of the FT-IR spectra of the original Cu/Zn library {**1**} (orange) and of the Cu/Zn library {**3**} after reaction with CO_2 and H_2 (green). The absorption bands at 1330 and 1380 cm^{-1} indicate the formation of copper-formate (C-H in plane bending / COO rocking and C-O symmetric stretching respectively).

1.2. $[Cu_4Zn_{10}]Cp*_8$ (F)

1.2.1. Experimental Characterization

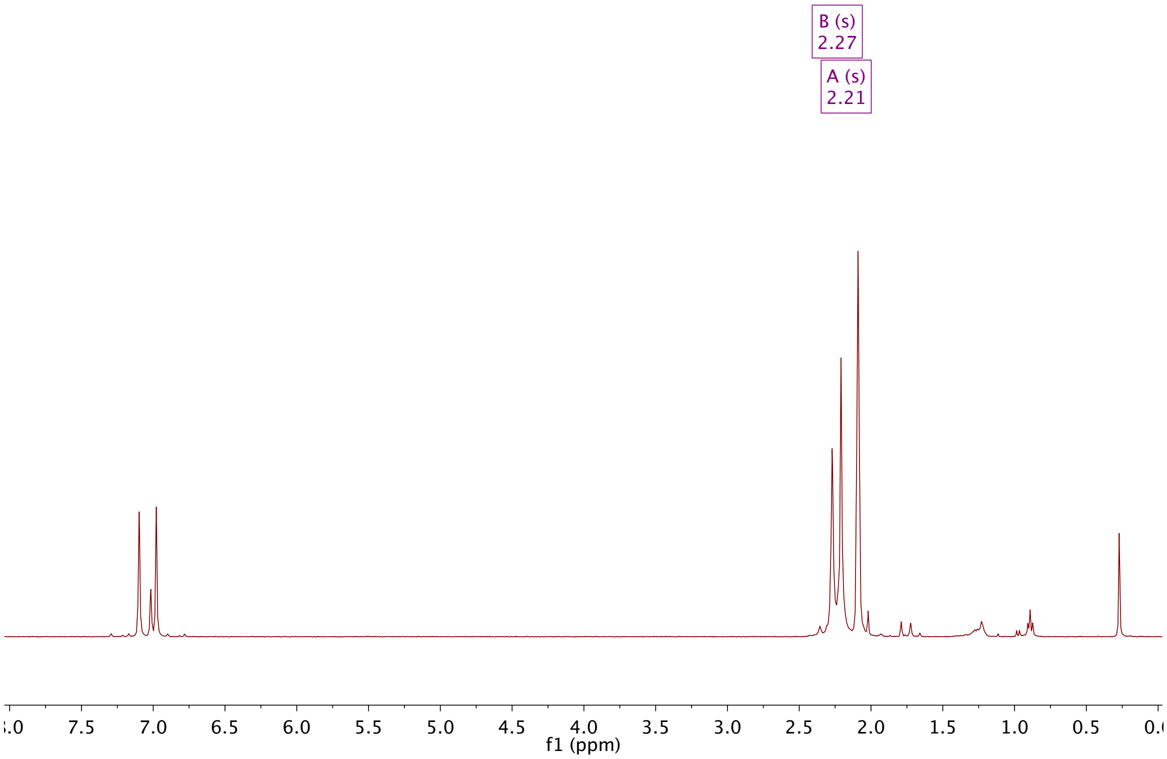

Supplementary Figure 138: ^{1}H NMR spectrum of $[Cu_4Zn_{10}]Cp*_8$ (**F**) (toluene-d_8). δ [ppm] = 2.27 (s, 45H, Cp*), 2.21 (s, 75H, Cp*).

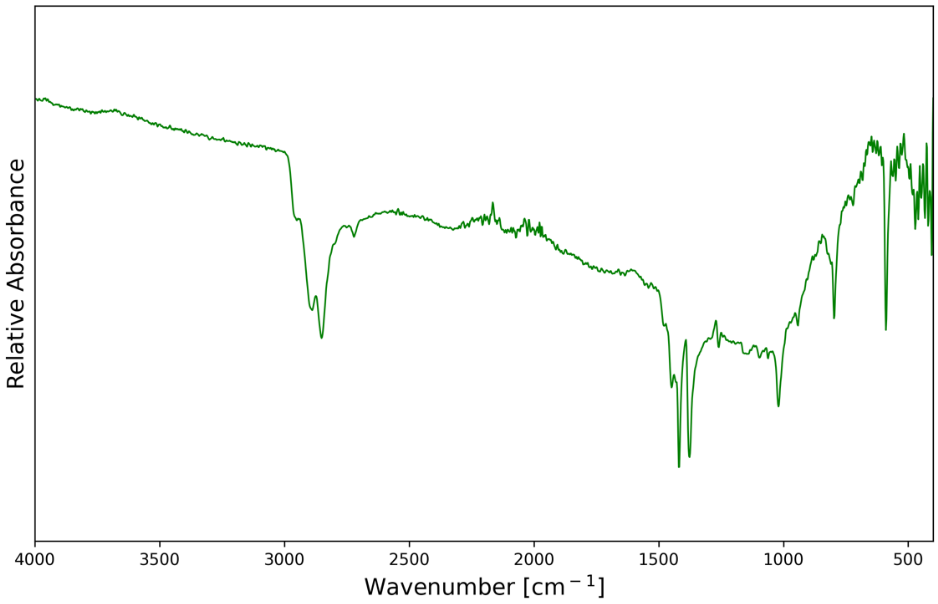

Supplementary Figure 139: ATR-IR spectrum of $[Cu_4Zn_{10}]Cp*_8$ (**F**). No hydride bands are observed. The C-H vibrations are observed between 2700 and 3000 cm^{-1}; the aromatic C-C vibrations between 1300 and 1500 cm^{-1}.

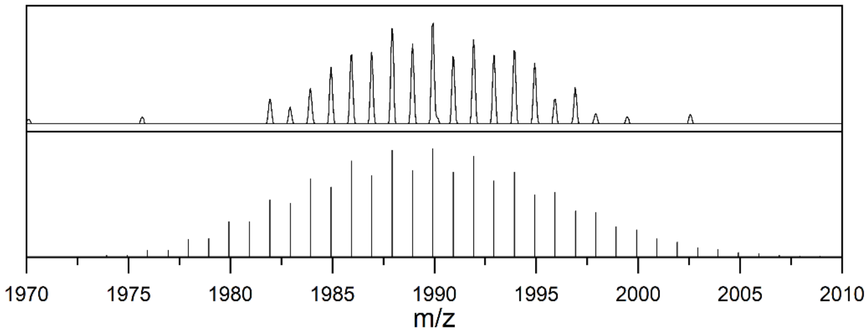

Supplementary Figure 140: Experimental LIFDI-MS (top) and calculated (bottom) peak pattern for the molecular ion of $[Cu_4Zn_{10}]Cp*_8$ (**F**).

1.2.1.1. Single-Crystal Crystallography

A black, plate-shaped crystal of **F**, $C_{80}H_{120}Cu_4Zn_{10}$, coated with perfluorinated ether and fixed on top of a Kapton micro sampler was used for X-ray crystallographic analysis. The X-ray intensity data were collected at 100(2) K on a Bruker D8 VENTURE three-angle diffractometer with a TXS rotating anode with MoK_α radiation (λ=0.71073 Å) using APEX4.[3] The diffractometer was equipped with a Helios optic monochromator, a Bruker PHOTON-100 CMOS detector, and a low temperature device.

A matrix scan was used to determine the initial lattice parameters. All data were integrated with the Bruker SAINT V8.40B software package using a narrow-frame algorithm and the reflections were corrected for Lorentz and polarization effects, scan speed, and background. The integration of the data using a triclinic unit cell yielded a total of 203812 reflections within a 2θ range [°] of 4.01 to 50.05 (0.84 Å), of which 16507 were independent. Data were corrected for absorption effects including odd and even ordered spherical harmonics by the multi-scan method (SADABS 2016/2).[4] Space group assignment was based upon systematic absences, E statistics, and successful refinement of the structure.

The structure was solved by iterative methods using SHELXT and refined by full-matrix least-squares methods against F^2 by minimizing $\Sigma w(F_o^2 - F_c^2)^2$ using SHELXL in conjunction with SHELXLE.[5-7] All non-hydrogen atoms were refined with anisotropic displacement parameters. Hydrogen atoms were refined isotropically on calculated positions using a riding model with their U_{iso} values constrained to 1.5 times the U_{eq} of their pivot atoms for terminal sp^3 carbon atoms and a C–H distance of 0.98 Å. Non-methyl hydrogen atoms were refined using a riding model with methylene, aromatic, and other C–H distances of 0.99 Å, 0.95 Å, and 1.00 Å, respectively, and U_{iso} values constrained to 1.2 times the U_{eq} of their pivot atoms. Neutral atom scattering factors for all atoms and anomalous dispersion corrections for the non-hydrogen atoms were taken from International Tables for Crystallography.[8] The unit cell of $C_{80}H_{120}Cu_4Zn_{10}$ contains 1.8 heavily disordered molecules of toluene which were treated as a diffuse contribution to the overall scattering without specific atom positions using the PLATON/SQUEEZE procedure.[9]

Crystallographic data for the structures reported in this paper have been deposited with the Cambridge Crystallographic Data Centre.[10] Supplementary crystallographic data reported in this paper have been deposited with the Cambridge Crystallographic Data Centre (CCDC 2390361) and can be obtained free of charge from The Cambridge Crystallographic Data Centre via www.ccdc.cam.ac.uk/structures.[10] This report and the CIF file were generated using FinalCif.[11]

Supplementary Table 7: Crystal data and structure refinement for cluster F.

CCDC number	2390361
Empirical formula	$C_{80}H_{120}Cu_4Zn_{10}$
Formula weight	1989.61
Temperature [K]	100(2)
Crystal system	triclinic
Space group (number)	$P\bar{1}$ (2)
a [Å]	12.5626(9)
b [Å]	16.6481(12)
c [Å]	25.1386(19)
α [°]	99.320(3)
β [°]	99.634(3)
γ [°]	111.420(3)
Volume [Å^3]	4680.7(6)
Z	2
ρ_{calc} [gcm^{-3}]	1.412
μ [mm^{-1}]	3.432
F(000)	2032
Crystal size [mm^3]	0.076×0.149×0.280
Crystal colour	black
Crystal shape	plate
Radiation	MoK_α (λ=0.71073 Å)
2θ range [°]	4.01 to 50.05 (0.84 Å)
Index ranges	$-14 \le h \le 14$<br>$-19 \le k \le 19$<br>$-29 \le l \le 29$
Reflections collected	203812
Independent reflections	16507<br>R_{int} = 0.0458<br>R_{sigma} = 0.0186
Completeness to θ = 25.027°	99.9 %
Data / Restraints / Parameters	16507 / 949 / 1093
Goodness-of-fit on F^2	1.039
Final R indexes [$I \ge 2\sigma(I)$]	R_1 = 0.0466<br>wR_2 = 0.1232
Final R indexes [all data]	R_1 = 0.0524<br>wR_2 = 0.1272
Largest peak/hole [eÅ^{-3}]	1.95/−0.96

Refinement details:

During refinement the structure of $[Cu_4Zn_{10}](Cp^*)_8$ has shown to be highly intricate. Overall, it comprises major disorder of metal atoms within the core. The issue is the fact that copper and zinc atoms cannot be distinguished from each other by using only crystallographic methods. Moreover, the first solution using SHELXT only showed 13 metal atoms instead of 14 and high residual electron density (up to 30 e^-) around the inner cluster core (Supplementary Figure 141).

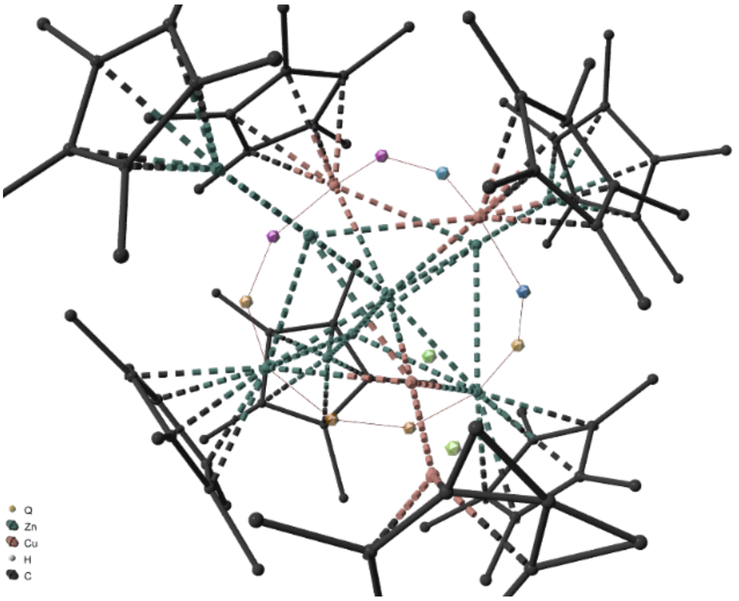

Supplementary Figure 141: First solution for $[Cu_4Zn_{10}](Cp^*)_8$ using SHELXT. The model only comprises 13 metal atoms and shows severe disorder of metal atoms around the inner cluster core.

Therefore, the 14th atom must be highly disordered in a circular fashion around the inner part of the cluster. Mass spectra acquired from isolated single crystals, however, unambiguously showed us that the sum formula $[Cu_4Zn_{10}](Cp^*)_8$ must be correct (Supplementary Figure 140). Note that there is no evidence for twinning for this structure (no split reflections, low symmetry, P-1).

The only structural motif that can be directly assigned from the first solution are Cu1 as the innermost atom of the cluster as well as four Zn–Zn–Cp* units (named Zn1–Zn7, Zn2–Zn8, Zn3–Zn9 and Zn4–Zn10 in the final model). Only zinc is capable of forming such a structural motif to the best of our knowledge. We deduced thereof that disorder of metal atoms is limited to the inner core of $[Cu_4Zn_{10}](Cp^*)_8$. Hence, two zinc and three copper atoms (minus the four Zn–Zn–Cp* units and Cu1) must be "distributed" between four Cp* units. This results in three "capping" and one Cp* bridging two metal atoms. Consequently, only five principle isomers (neglecting enantiomers and diastereomers) may exist for the core of this compound (Supplementary Figure 142). DFT calculations (*vide infra*) showed us that isomer **I** with two bridging copper units is the one lowest in energy.

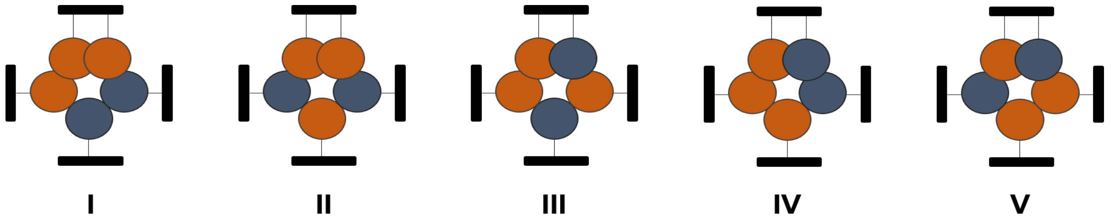

Supplementary Figure 142: Possible isomers for the core structure of $[Cu_4Zn_{10}](Cp^*)_8$.

Based on these data, we found in total four layers of disordered metal atoms during refinement. For that, the position and the atomic displacement parameters for atoms at the same positions were constrained to be equal using the commands EADP and EXYZ. All four layers were added up to 1 using SUMP. The over-all result of the DFG guided X-ray structural analysis of **F** is given in Supplementary Figure 143a.

1.2.2. DFT Bonding Analysis

The single crystal X-ray structure of **F** (Supplementary Figure 143a) suffers accuracy from its disordered nature and, owing to the close proximity of the Cu and Zn atomic numbers, some of the metal positions could not be clearly identified as M = Cu or Zn. This fairly unsymmetrical structure reveals a central Cu atom surrounded by eight moieties: four Zn-ZnCp*; three MCp* and a "bidentate" $M_2\{\mu\text{-}(\eta^2\text{:}\eta^2\text{-}Cp^*)\}$. This last coordination mode is expected to be highly fluctional. This results in a ninefold coordination of the central copper atom. Furthermore, some bonding interactions seems to exist between these nine metal atoms (Supplementary Figure 143a).

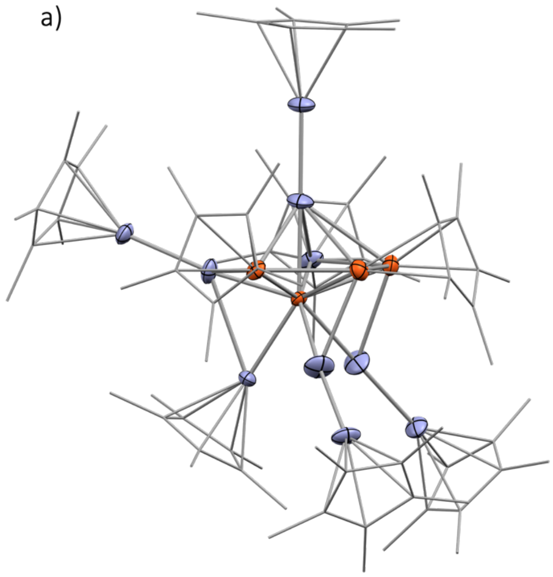

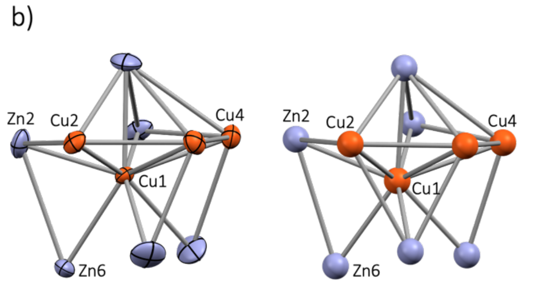

c)

Bond	SC-XRD [Å]	DFT Optimized F_1 [Å]	Wiberg Bond Indices F_1
Cu1 – Cu2 (CuCp*)	2.395	2.405	0.196
Cu1 – Cu4 (Cu_2Cp^*)	2.420	2.389	0.178
Cu1 – Zn2 (ZnZnCp*)	2.347	2.331	0.094
Cu1 – Zn6 (ZnCp*)	2.405	2.309	0.302

Supplementary Figure 143: a) Single-crystal X-Ray diffraction structure of **F** (ellipsoids drawn at 50% probability, Cp* as wireframe for clarity). b) Comparison of the experimental (left) and DFT optimized (right) $[Cu_4Zn_6]^{4+}$ core

of **F**. c) Selected representative bond lengths from the SC-XRD and DFT optimized structure as well as the associated Wiberg bond indices. Color code: copper = orange; zinc = blue, carbon = grey.

DFT calculations at the BP86/TZ2P/ D3(BJ) level (see details in the methods section of the main text) were carried out on **F**, in order to ascertain the Cu *vs.* Zn nature of the M positions and to provide a rationalization of its structure and stability. Calculations were performed from the X-ray structure of **F** as starting geometry and the optimizations of a series of isomers with various Cu *vs.* Zn distributions on the metal sites were performed. The ten most energetically favorable structures (Supplementary Table 8) exhibit significant structural differences between them. Nevertheless, the lowest energy isomer (**F_1**) is also the closest to the X-ray structure (Supplementary Figure 143b and Supplementary Table 9). From the electron counting point of view, the number of cluster metal valence electrons (discarding 3d electrons) of $[Cu_4Zn_{10}](Cp^*)_8$ (**F**) is 4 + (10 x 2) – 8 = 16. Assuming that the Zn-Zn bonds in the four Zn-ZnCp* units are localized 2-electron/2-center bonds, the number of electrons associated with metal-metal bonding within the coordination sphere of the central Cu atom is 16 – (4 x 2) = 8. Together with the 10 non-bonding 3d(Cu) electrons, this results in a central Cu atom following the 18-electron rule, analogous to a regular ML_n organometallic complex (Supplementary Figure 144). However, regular organometallic complexes do not exhibit bonding between ligands, whereas in **F** several bonding contacts exist between the metalloligands surrounding the central Cu (Supplementary Figure 143). This is a result of the delocalization of the 8 cluster bonding electrons. This delocalization is caused by the number of metalloligands exceeding the number of available AOs for bonding to the central d^{10} copper center (four). This is further facilitated by the possibility of non-negligible overlap between the metalloligands, allowing the nine diffuse sp-type (4s/4p) frontier orbitals of the eight metalloligands (seven mono- and one bi-dentate) to interact between one another. This interaction causes a dispersion of the orbitals of the metalloligands sphere fragment, in turn allowing the bonding orbitals to interact preferentially with the 4s and 4p AOs of the central Cu, then conferring to the four MOs containing the 8 metal electrons with a substantial metalloligands···metalloligands bonding character, as exemplified by the corresponding Wiberg bond indices (Supplementary Table 8). This bonding situation, sketched in Supplementary Figure 144 was already put forward in the case of $[CuZn_{10}](Cp^*)_7$.[12]

Supplementary Table 8: Relative total (ΔE) and free (ΔG) energies and HOMO-LUMO gaps (ΔE_{H-L}) of the computed isomers of $[Cu_4Zn_{10}](Cp^*)_8$ (**F**) of lowest energy.

Isomer	*F_1*	*F_2*	*F_3*	*F_4*	*F_5*	*F_6*	*F_7*	*F_8*	*F_9*	*F_{10}*
ΔE (eV)	0.00	0.06	0.15	0.15	0.20	0.36	0.57	0.60	0.63	1.30
ΔG (eV)	0.00	0.12	0.11	0.22	0.20	0.42	0.56	0.54	0.64	1.17
ΔE_{H-L} (eV)	1.38	1.63	1.27	1.39	1.41	1.28	1.21	1.44	1.33	1.48

Supplementary Table 9: Selected computed data for **F₁** (WBI = Wiberg bond index).

Interatomic contact	*Distance (Å)*	*WBI*
Cu1-Cu2	2.405	0.196
Cu1-Cu3	2.493	0.120
Cu1-Cu4	2.389	0.178
Cu1-Zn1	2.407	0.056
Cu1-Zn2	2.331	0.094
Cu1-Zn3	2.328	0.084
Cu1-Zn4	2.335	0.103
Cu1-Zn5	2.321	0.238
Cu1-Zn6	2.309	0.302
Zn1-Zn7	2.318	0.517
Zn2-Zn8	2.318	0.451
Zn3-Zn9	2.309	0.502
Zn4-Zn10	2.319	0.444
Cu2-Cu3	2.415	0.165
Cu3-Cu4	2.423	0.067
Zn1-Cu2	2.538	0.090
Zn1-Cu3	2.541	0.058
Zn1-Cu4	2.723	0.033
Zn1-Zn5	2.676	0.057
Zn2-Cu2	2.692	0.064
Zn3-Cu2	2.600	0.063
Zn3-Cu3	2.502	0.088
Zn4-Cu4	2.540	0.096
Zn5-Cu4	2.513	0.123
Zn4-Zn6	2.784	0.048

Atom	*Natural atomic charge*
Cu1	-0.72
Cu2	0.20
Cu3	0.34
Cu4	0.24
Zn1	0.56
Zn2	0.50
Zn3	0.55
Zn4	0.52
Zn5	0.82
Zn6	0.74
Zn7	0.70
Zn8	0.65
Zn9	0.71
Zn10	0.66

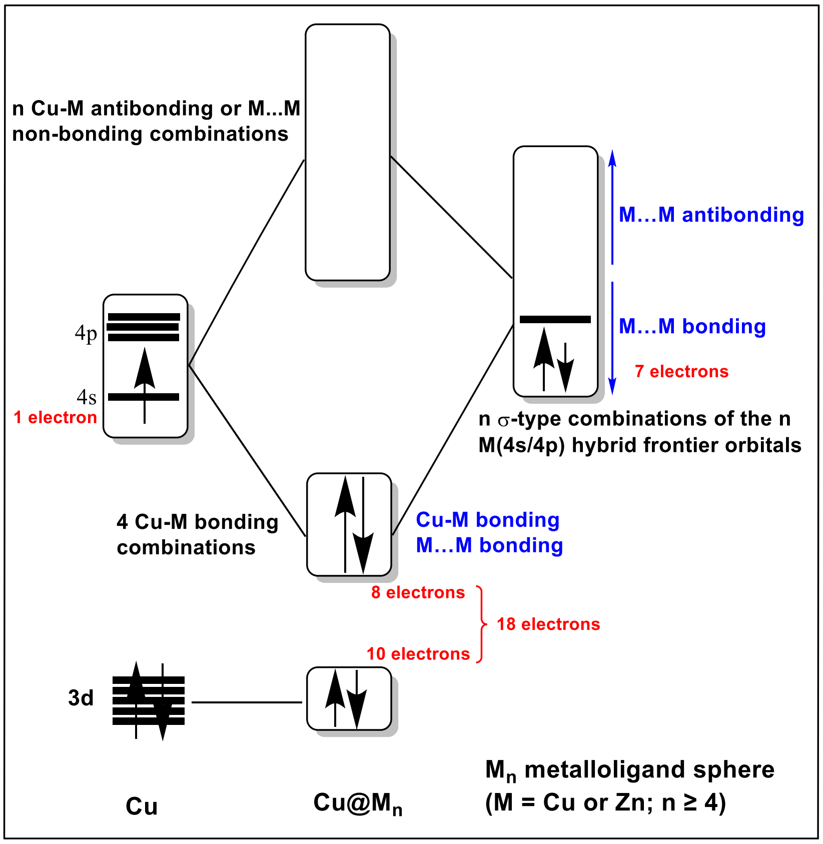

Supplementary Figure 144: Simplified MO interaction diagram for a stable cluster made of a central Cu atom surrounded by at least four metalloligands of the type CuCp*, ZnCp*, ZnZnCp*…, thus having (at least) one σ-type frontier orbital, and capable of allowing bonding interaction between them. In the case of **F**, the metalloligand sphere is composed of the following units: four 1-electron Zn-ZnCp*, two 1-electron ZnCp*, one 0-electron CuCp* and a bidentate 1-electron Cu_2\{μ-(η^2:η^2-Cp*)\}.

1.3. Catalytic Semi-Hydrogenation

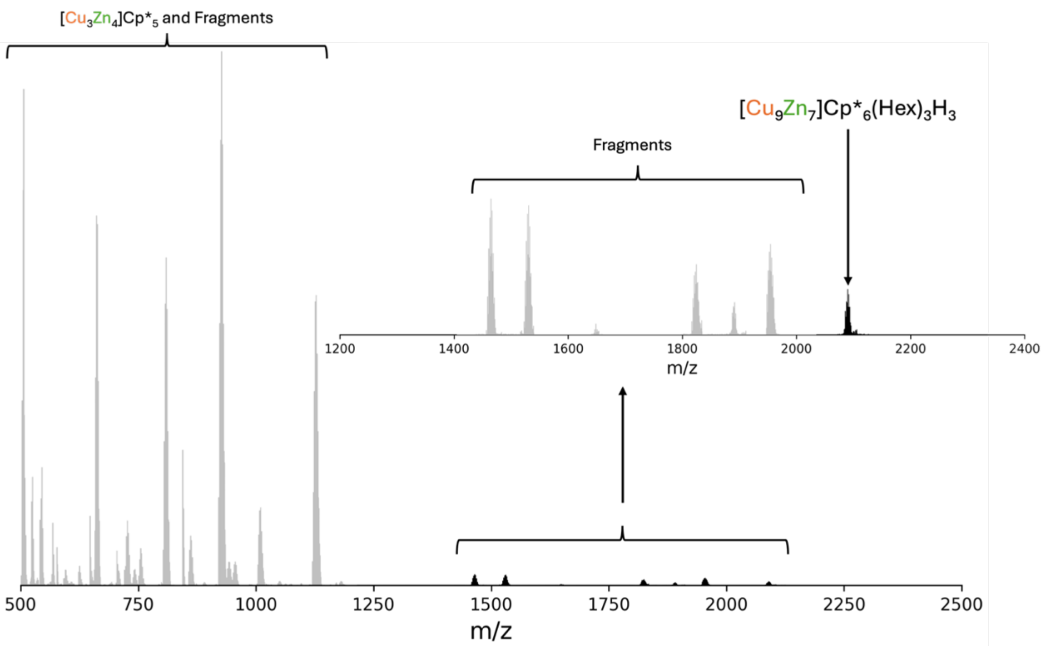

Supplementary Figure 145: In situ LIFDI-MS of the conversion of 3-hexyne with H_2 and 5.0 mol% $[Cu_4Zn_{10}]Cp^*_8$ (**F**) at 100 °C showing the formation of $[Cu_9Zn_7]Cp^*_6(Hex)_3H_3$ (**W**). $[Cu_3Zn_4]Cp^*_5$ (**B**) is also observed as due to its facile ionization. It is detectable in the mass spectrum, as much lower quantities are sufficient as compared for ^{1}H-NMR observation.

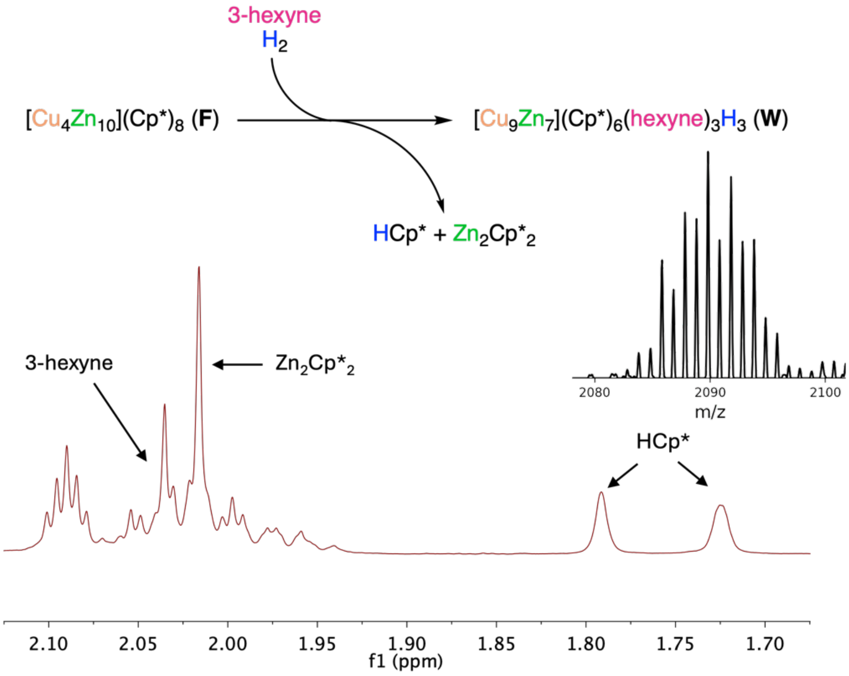

Supplementary Figure 146: Reaction scheme for the formation of species **W** from **F** upon conversion with 3-hexyne/H_2 at 100 °C. The side products are identified by ^{1}H-NMR spectroscopy and the patter of the molecular ion of **W** is shown.

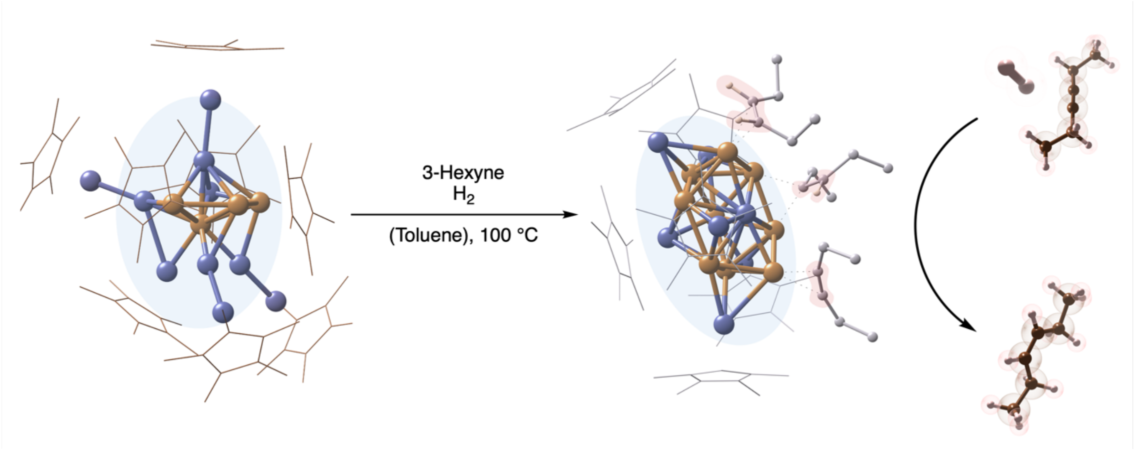

Supplementary Figure 147: Schematic representation for the formation of species **W** from **F** upon conversion with 3-hexyne/H_2 at 100 °C and subsequent semi-hydrogenation of 3-hexyne.

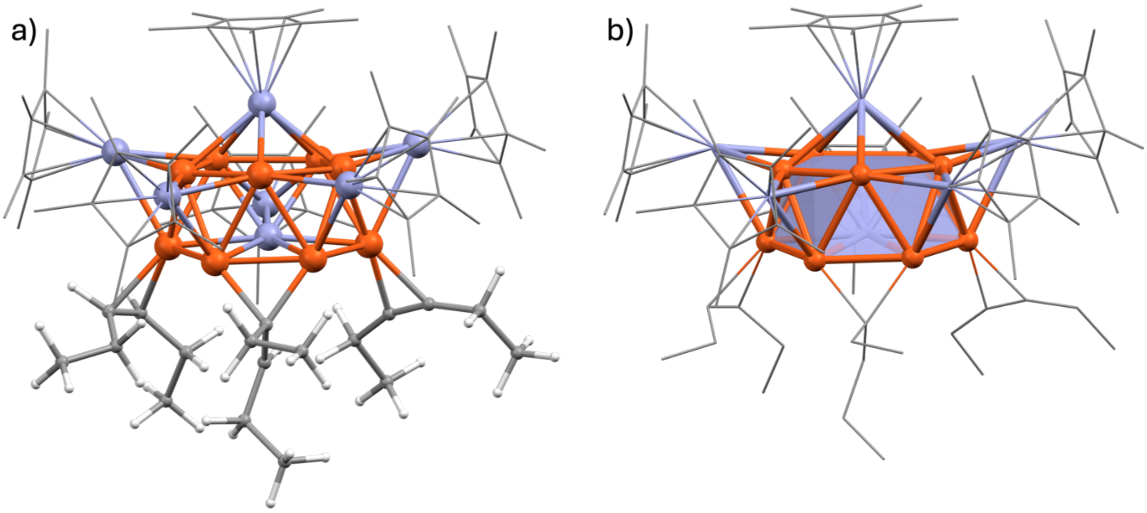

Supplementary Figure 148: Calculated structure of $[Cu_9Zn_7](Cp^*)_6(Hex)_3H_3$ (**W**) showing species in a $\mu_1\eta^2$-3-hexyne, $\mu_1\eta^2$-cis-3-hexene and $\mu_2\eta^1$-cis-3-hexenyl coordinated to cluster core as a result of hexyne insertion into cluster hydride species (alkyne semi-hydrogenation mechanism). a) Calculated structure of **W** (Cp* as wireframe for clarity) and b) Calculated structure of **W** with the surface of the Cu_9Zn pentagonal antiprism marked (ligands as wireframe for clarity). Color code: orange = copper, blue = zinc, grey = carbon and white = hydrogen.

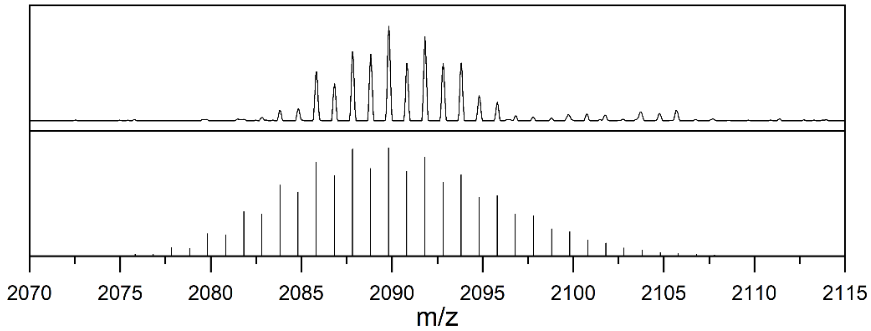

Supplementary Figure 149: Experimental pattern (top) and calculated pattern (bottom) for $[Cu_9Zn_7](Cp^*)_6(Hex)_3H_3$ (**W**).

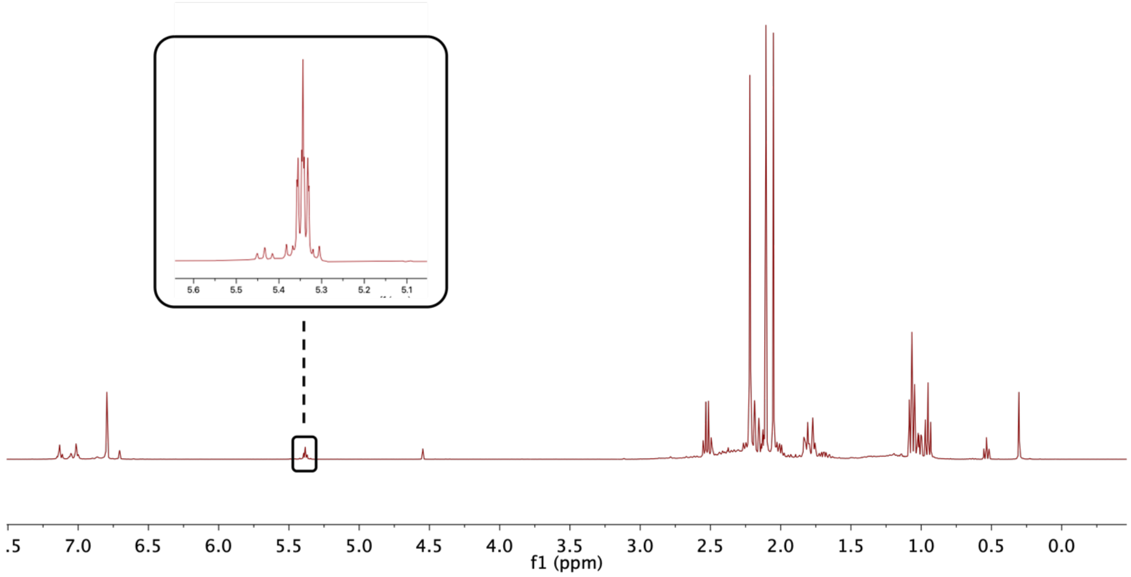

Supplementary Figure 150: ^{1}H NMR spectrum of the Cu/Zn library {**4**} (toluene-d_8) showing the catalytic formation of 3-hexene after 4 hours reaction time.

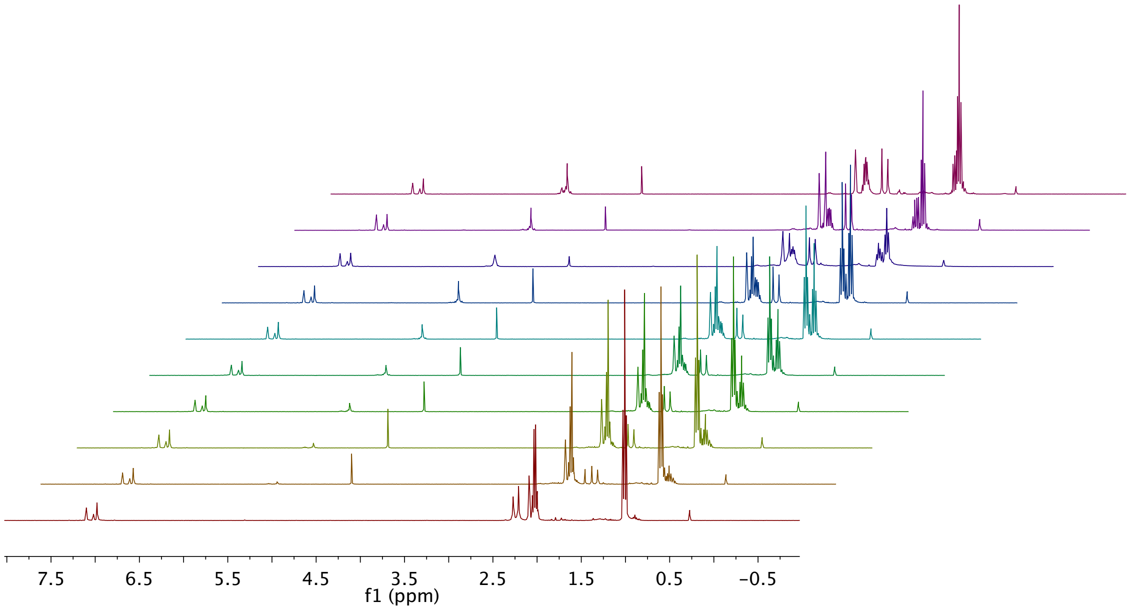

Supplementary Figure 151: ^{1}H NMR spectrum of the catalytic semi-hydrogenation of 3-hexyne with 5 mol% **F** (toluene-d_8). No n-hexane formation is observed.

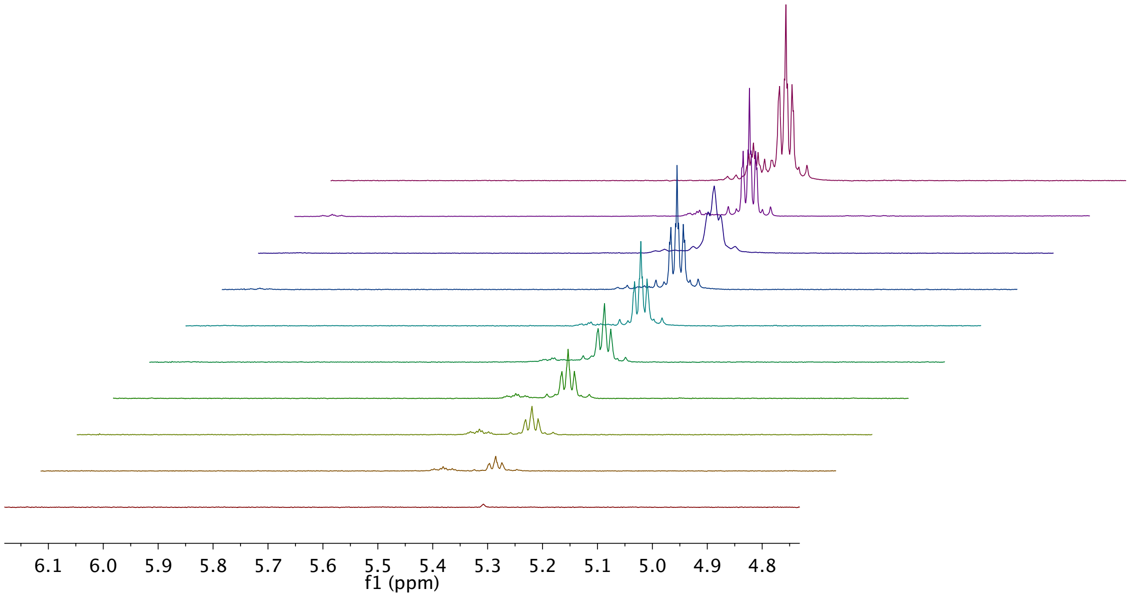

Supplementary Figure 152: ^{1}H NMR spectrum of the catalytic semi-hydrogenation of 3-hexyne with 5 mol% **F** (toluene-d_8) focused on the CH_2 signals of the 3-hexens (cis and trans).

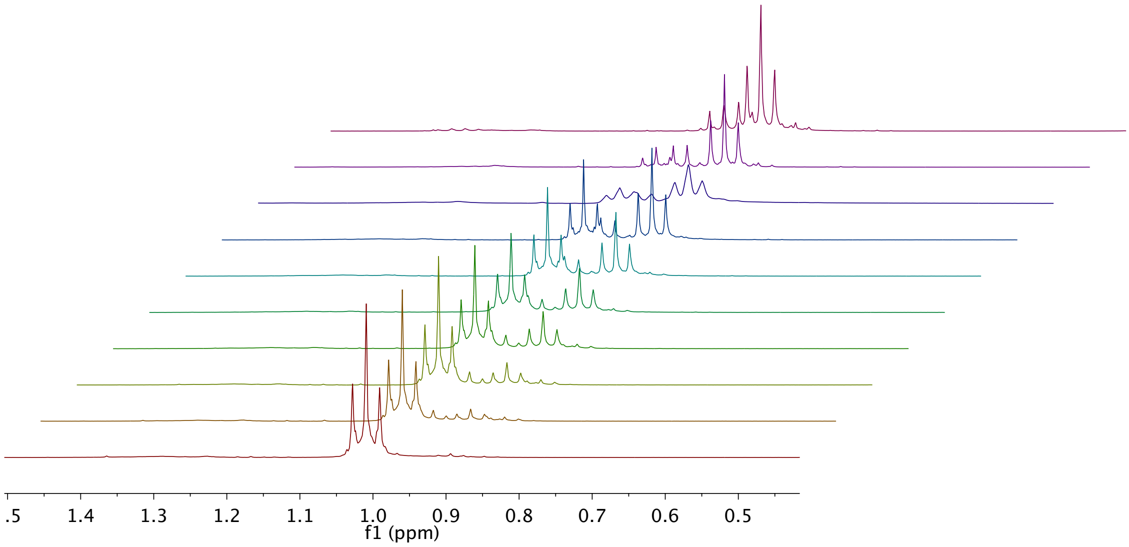

Supplementary Figure 153: ^{1}H NMR spectrum of the catalytic semi-hydrogenation of 3-hexyne with 5 mol% **F** (toluene-d$_8$) focused on the CH_3 signals of the 3-hexyne and 3-hexenes (cis and trans).

Calculation and Comparison of TOF data:

The TOF data were determined based on the conversion of 3-hexyne after four hours with both **F** and {**1**} as pre-catalyst and pre-catalytic system respectively. As the quantification of **F** in {**4**} is not possible due to an overlap of the different species of {**1**} in the ^{1}H NMR spectrum, the TOF is calculated based on the total molar amount of Cu in solution. In this regard, 5 mol% **F** $[Cu_4Zn_{10}](Cp^*)_8$ results in the presence of 20 mol% Cu with respect to the amount of 3-hexyne. For the catalytic semi-hydrogenation with {**1**} as pre-catalytic system (resulting in {**4**} during the catalysis), the conversion of 3-hexyne is performed with a total molar amount 27 mol% Cu (derived from the synthetic conditions of library {**1**}) with respect to the amount of 3-hexyne. Thus, we compare the obtained TOF values of the two experiments to the same level of mol% Cu present in the reaction solution. The conversion of 3-hexyne to *cis*- and *trans*-3-hexene and *n*-hexane was monitored via ^{1}H NMR. After four hours of reaction time, the conversion of 3-hexyne is observed to be 48% with the isolated **F** and 16% with {**4**}. This results in a TOF of 0.60 $h^{-1}Cu^{-1}$ when using isolated **F** as pre-catalyst (TOF = 2.4 h^{-1} based on **F**) and of 0.15 $h^{-1}Cu^{-1}$ with {**1**} as precatalytic system.

$$TOF = \frac{TON}{t} = \frac{\frac{n_{hexene}\%}{n_{cat}\%}}{t}$$

$$TOF_{\boldsymbol{F}} = \frac{\frac{48\ mol\%}{20\ mol\%}}{4\ h} = 0.60\ h^{-1}Cu^{-1}$$

$$TOF_{\{\mathbf{4}\}} = \frac{\frac{16\ mol\%}{27\ mol\%}}{4\ h} = 0.15\ h^{-1}Cu^{-1}$$

1.4. References

1. Dyson, P. J., Johnson, B. F., McIndoe, J. S. & Langridge-Smith, P. R. Energy-dependent electrospray ionisation mass spectrometry: applications in transition metal carbonyl chemistry. *Rapid Commun. Mass Spectrom.* **5**, 311-313 (2000)
2. Diedrich, J. K., Pinto, A. F. & Yates III, J. R. Energy dependence of HCD on peptide fragmentation: stepped collisional energy finds the sweet spot. *J. Am. Chem. Soc. Mass Spectrom.* **11**, 1690-1699 (2013)
3. APEX4 Suite of Crystallographic Software. Version 2021-10.0 ed: Bruker AXS Inc., Madison, Wisconsin, USA; 2021.
4. Krause, L., Herbst-Irmer, R., Sheldrick, G. M. & Stalke, D. 2015. *J. Appl. Cryst.* **48**, 3–10
5. Huebschle, C. B., Sheldrick, G. M. & Dittrich, B. *J. Appl. Cryst.* 1281–1284 (2011)
6. Sheldrick, G. M. *Acta Cryst.* **A71**, 3–8 (2015)
7. Sheldrick, G. M. *Acta Cryst.* 3–8 (2015)
8. *International Tables for Crystallography Volume C, Mathematical, Physical and Chemical Tables*. International Union of Crystallography: Chester, England, 2006.
9. Spek, A. *Acta Cryst.* 9-18 (2015)
10. Groom, C. R., Bruno, I. J., Lightfoot, M. P. & Ward, S. C. *Acta Cryst.* 171–179 (2016)
11. Kratzert, D. FinalCif, V106. [cited]Available from: https://dkratzert.de/finalcif.html.
12. Schütz, M., *et al.* Contrasting Structure and Bonding of a Copper-Rich and a Zinc-Rich Intermetalloid Cu/Zn Cluster. *Inorganic Chemistry* **13**, 9077-9085 (2020)

2 Computational Framework

The computational framework allows us to explore all different structural possibilities and compare the energies of the various possible structures of a given clusters to determine the most likely one. The energies of different structures can only be used relative to one another for a given sum formula.

2.1 Introduction to the Generation of Metal Complexes

The computational design of molecular structures can be achieved using global optimization algorithms such as Basi-Hopping Monte Carlo (BHMC),[1] where the Monte Carlo algorithm is combined with local optimization algorithms such as conjugated gradient, steepest descent, etc.[2] The investigation of phase space necessitates an extensive number of total energy minimization, which can be performed using either *ab initio* methods or force field theoretical frameworks.[1] Normally, those techniques, e.g., BHMC, have been applied for the study of a wide range of molecular systems, including particles (clusters and nanoclusters), in which the starting molecular configurations are atoms randomly distributed within a box space.[1]

In general, metal complexes are composed of a metal core with a few atoms surrounded by ligand species. Thus, metal complexes can be visualized as molecular structures composed of two molecular fragments, namely (i) a core region composed of few atoms and (ii) chemical species connected to the core region (ligands).[3] Therefore, the computational design of metal complexes can take advantage of this observation to accelerate the computational design of molecular trial configurations for evaluations by total energy methods.

Thus, our computational strategies take advantage of our computational global optimization techniques in the study of finite-size metal clusters,[1] density functional theory (DFT) calculations for finite-size systems,[4] and machine learning-based algorithms.[5] As expected in any development study, we considered and implemented several computational strategies (algorithms), such as different core generation procedures, various scripts for the distribution of ligands around the core region, different optimization routines, etc. Thus, in the end, the present document only summarizes the final and successful algorithm, which is summarized in

Figure 154. It contains several modules that work sequentially step by step to deliver a set of family structures for a selected metal complex.

As indicated in the flow chart, the present algorithm can be implemented using different levels of computational methodologies for the total energy evaluations at each step, namely *ab initio* calculations based on DFT or less accurate force field calculations. For example, for the DFT calculations, the following DFT implementations could be used: (i) Vienna *Ab initio* Simulation Package (VASP),[6,7] Amsterdan Density Functional (ADF),[8] GAMESS,[9] GAUSSIAN,[10], Fritz–Harber Institute - *Ab initio* Materials simulation package (FHI-aims),[11,12] etc.

In this work, for the computational design of metal complexes, we selected the FHI-aims implementation, which provides great flexibility to control the computational cost of the DFT calculations by a proper selection of the basis-set; i.e., it contains different levels of the basis-set. In the following, we will discuss step by step all the computational details on the algorithms. Furthermore, we provided the procedure compiled in an automated `Python` language, namely `cluster_assembler`, which is available at `https://github.com/QTNano/Cluster-Assembler/`.

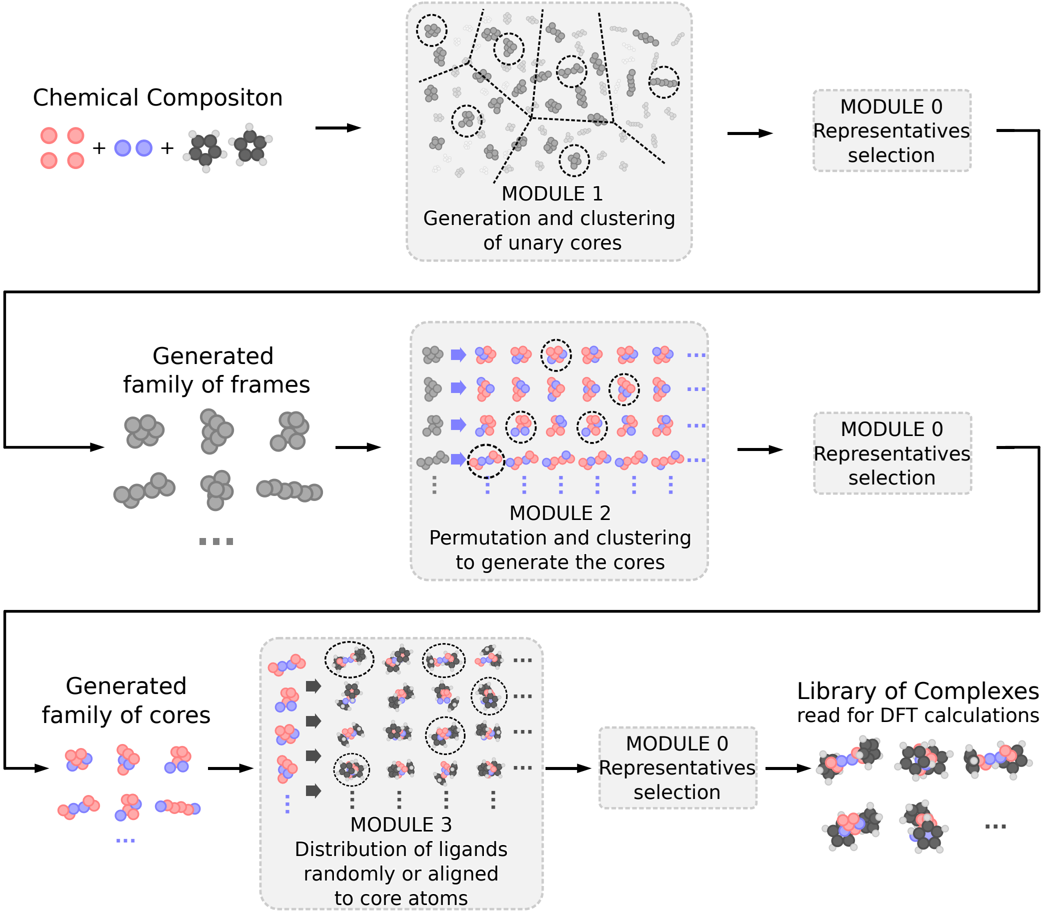

Supplementary Figure 154: Flowchart illustrating a general computational framework to design molecular structures for metal complexes.

2.2 Additional Computational Details on the Framework for the Generation of Metal Complexes

The proposed algorithm is general, however, to make the discussion easier, we selected the [Cu_aZn_b] system for our discussion, which will helps to understand the proposed ideas and selected strategies to build up a family of metal complexes *ab initio* computational design, i.e., it does not include previous experimental knowledge on the metal complex structure.

2.2.1 Generation of Molecular Unary Frames

Given the desired chemical composition of the cores, e.g., [Cu_aZn_b], we start by generating a family of unary frames, e.g., [$Cu_{(a+b)}$] and [$Zn_{(a+b)}$]. The process begins by defining the shape

of the box that will be used to construct the frame. Two types of boxes are considered: Sphere and Cube boxes. For example,

1. For the Sphere, the radius is determined by the following formula[13]:

$$\text{sphere\_radius} = 2 \times d_0 \times \left(\text{factor} + \left(\frac{3 \times n_{\text{atoms}}}{4 \times \pi \times \sqrt{2}} \right)^{\frac{1}{3}} \right)$$

   where d_0 is the covalent radius of the species, factor is a tolerance parameter, and n_{atoms} is the number of atoms in the frame.

2. For the Cube box, the edge length of the cube container is set to:

$$\text{cube\_edge} = \text{sphere\_radius} \times \left(\frac{4 \times \pi}{3} \right)^{\frac{1}{3}}$$

The atoms are iteratively added to the frame, with the constraint that each atom must be placed inside the defined box. Additionally, the distance to the nearest neighbor must respect the covalent radius of the species with a gamma tolerance. Specifically, the distance between the newly added atom and its closest neighbor must be within the range $d_0 - \gamma$ to $d_0 + \gamma$, where γ is a tolerance parameter that accounts for slight variations in the lengths of the bonds.

Finally, to guarantee that all frames are valid, we provide a Python function that checks the frame structure using a graph approach. For that, the frame structure is encoded as a graph, where nodes represent atoms, and edges represent the links between pairs of atoms whose distance is shorter than the covalent radius multiplied by a parameter greater than one, representing the tolerance for establishing the connection. If the resulting graph contains a single component, the frame is approved. Otherwise, it is rejected. At the end of this process, the generation of the frames is provided by Module 1, depicted in Supplementary Figure 154.

2.2.2 Clustering of Molecular Structures via Coulomb Matrix Representation

To manage the large number of trial configurations and identify unique structures, we apply the *k-means* clustering algorithm.[14] First, we encode all structures into vectors using the eigenvalues of their Coulomb matrices,[15] allowing us to group similar configurations effectively. The

Coulomb matrix is a representation of the molecular structure where each element M_{ij} is defined as:

$$M_{ij} = \begin{cases} 0.5 Z_i^{2.4} & \text{if } i = j \\ \frac{Z_i Z_j}{\|\mathbf{R}_i - \mathbf{R}_j\|} & \text{if } i \neq j \end{cases}$$

in which, Z_i and Z_j are the atomic numbers of atoms i and j, respectively, and $\|\mathbf{R}_i - \mathbf{R}_j\|$ is the Euclidean distance between atoms i and j. The eigenvalues of the Coulomb matrix provide a compact, invariant descriptor of the molecular configuration that is used for clustering.

The *k-means* clustering algorithm is a partitioning method that divides n observations into k groups. Each observation belongs to the group with the nearest mean, or centroid. In our case, we select the configuration closest to the centroid as the representative of the group. Formally, given a set of observations $(x_1, x_2, ..., x_n)$, where each observation is a d-dimensional real vector, *k-means* clustering aims to partition the n observations into k ($\leq n$) sets $S = \{S_1, S_2, ..., S_k\}$ to minimize the within-group sum of squares (WCSS):

$$\arg\min_S \sum_{i=1}^{k} \sum_{x \in S_i} \|x - \mu_i\|^2$$

where μ_i is the mean of points in S_i (cluster centroid).

The number of groups, k, can be specified by the user or automatically determined. For automatic determination, we use silhouette analysis from multiple runs of the clustering algorithm.[16] The silhouette score evaluates the similarity of an object to its own group compared to others. By running the clustering algorithm n times and evaluating the silhouette score for each run, we select the most frequent k (number of groups) which results in the highest silhouette score.

This method ensures that we efficiently and accurately identify unique configurations from the large set of trial structures, providing a robust way to analyze and filter the generated frames. It is worth noting that this module, named Module 0, might be invoked in the following modules as well, in order to reduce the number of chemical structures.

2.2.3 Generation of the Binary Core Structures

The next step in our methodology involves generating binary cores based on the frames provided by Module 1. Considering the same composition illustrated in Section 2.2.1, [Cu_aZn_b], we start by taking each frame, with the composition [$Cu_{(a+b)}$] or [$Zn_{(a+b)}$], and run all possible permutations by replacing the original atoms of the frame with a atoms of Zn and b atoms of Cu.

After generating all permutations, we apply the same connectivity filter used in Module 1 (see Section 2.2.1) to validate the cores produced by this step, which we refer to as Module 2. This filter ensures that the generated cores are chemically reasonable by checking that each atom is within a valid distance range from its nearest neighbors, adhering to the specified covalent-radius tolerance.

The permutation process can generate a large number of structures, especially when a and b are larger. Thus, to manage this potentially large number of structures and identify a small set of representative cores, we can also use the clustering method described in Section 2.2.2. This involves applying the *k-means* clustering method on data represented by the eigenvalues of the Coulomb matrix. This approach ensures that we maintain a diverse yet manageable set of binary core structures for further analysis and optimization. By combining the permutation of elemental compositions and clustering, Module 2 generates valid and representative binary cores based on the initial frames from Module 1.

2.2.4 Low-cost Geometric Optimizations for the Core

At the end of this step, we obtained a set of trial molecular configurations for the core; however, these fragments will be combined with the ligands in the following modules for final total energy optimizations. Therefore, in this step, it is not necessary to perform high-accurate geometry optimizations. Thus, it is recommend to use low-cost DFT calculations. In this step, we used lower-cost geometric optimizations based on smaller basis-set. Therefore, for the examples provided in the following sections, the family of binary cores was submitted at the PBE+TS-vdW optimization level with the *light-tier1* basis-set improvement and light convergence parameters of the FHI-aims[11] package. Additional information on the theoretical approach and computational details is provided in Section 2.2.9.

2.2.5 Generation of Sites on the Core Surface

We start by generating a set of n sites (points) uniformly distributed over a sphere that circumscribes the core (one of the fragments of the metal complex). For this purpose, two steps are considered:

a) We use a Fibonacci approach, named the Fibonacci lattice, to distribute the sites.[17] This method uses the Fibonacci sequence to distribute n points so that they are approximately equidistant from each other on the surface of the sphere;

b) The sites defined in Step a) undergo a fine adjustment based on a coarse force field process. Each site is considered a charged particle that is loose over the surface of the sphere. Because the particles have the same charge, they repel each other until an equilibrium is reached. In equilibrium, the n points are almost equally spaced on the surface of the sphere.

c) Furthermore, the defined sites can also be adjusted to the core surface (deformation), which can be activated by the user by selecting the deformation step.

Thus, our implementation provides great flexibility to the generation of the n points on the core region, which is a key part of the algorithm.

2.2.6 Addition of Ligands on the Core Surface

Given the sites, the method will begin to add the ligands selected by the user at a predetermined distance d. An arbitrary number of different ligands can be considered; for example, the user can choose to add m ligands A and n ligands B, e.g., Cp, Cp* for A and B, respectively. The ligands are added to the site using one of two distinct approaches, also selected by the user during the simulation:

a) The ligands are randomly positioned. In this case, for each ligand, the molecule will be randomly rotated before addition;

b) The ligands are positioned in an oriented manner. In this scenario, the user must provide a set of atoms from the ligand to orient the positioning.

2.2.7 Complexes Filtering

For large $[Cu_aZn_b]A_mB_n$ complexes with a large number of ligands, the generated structures may exhibit unrealistic configurations, such as closely spaced overlapping atoms, which can lead to issues in electronic structure calculations, i.e., those calculations cannot reach electronic convergence. To address this, we have implemented a filter to prevent atoms from overlapping. When applying this filter, we check if the following condition holds for all possible distances d between atoms a and b:

$$d \geq (r_a + r_b) \times 0.8 \, , \tag{1}$$

where r_a and r_b represent the covalent radii of each pair of atoms a and b, respectively. If any distance between atoms violates this rule, the structure is discarded. The threshold of 0.8 can be adjusted to be more lenient or stricter, depending on the specific requirements.

2.2.8 Representative Complexes via Clustering Algorithm

Finally, since the number of complexes can be very large and with many redundancies (very similar structures), the clustering method depicted in Section 2.2.2 can also be activated to reduce the complexes to a smaller subset of representative molecular structures. Following this methodology, we ensure that the generated complexes are both chemically reasonable and diverse, providing a robust family of structures. Therefore, the combination of the *k-means* algorithm based on a Coulomb representation and the silhouette analysis from multiple runs[16] plays a critical role in selecting the most successful molecular configurations for the evaluation of total energy by geometric optimizations.

2.2.9 Low-cost Geometric Optimization of Metal Complexes

At this step, the final family of $[Cu_aZn_b]R_n$ complexes is externally submitted to DFT calculations with low computational parameters for the self-consistent convergence of the electron density and optimization parameters. For the examples provided in the following sections, these complexes were optimized at the PBE+TS-vdW level with the *light-tier1* basis set and light convergence parameters using the FHI-aims software.[11] The optimized set provides

a $[Cu_aZn_b]R_n$ family of local minimum configurations for each composition of interest, where we expect to find structures that closely resemble those obtained experimentally, as they exhibit lower relative energy. Although additional configurations, particularly those with a great variety of R_n ligands, can be manually designed, in which this final family of complexes can serve as inspiration. Moreover, additional physical-chemical properties observed experimentally for the complexes can guide this design process more efficiently towards new local minimum configurations.

2.2.10 Optimization with Tight Criteria

At this optional step, the selected representative structures via our clustering implementation can be externally submitted to DFT optimization with tight self-consistence parameters and optimization parameters, particularly to perform additional analysis, e.g., NBOs, AIMS, EDA-NOC, and others. Depending on the computational resources, this step can be done previously for a large number of configurations; however, it will increase the computational. From our computational tests, two-step geometric optimization plays an important role in reducing the computational cost.

2.3 Additional Details on the Theoretical Approach and Computational Details for the Optimization Calculations

All *ab initio* total energy calculations were based on spin-polarized DFT within the semi-local exchange-correlation energy functional proposed by Perdew–Burke–Ernzenhof (PBE).[18] To improve the description of weak long-range interactions for all complexes, we used the Tkatchenko–Scheffler (TS) van der Waals (vdW) correction (vdW-TS).[19] In this formalism, the attractive vdW energy correction is added to the plain DFT-PBE total energy.

The calculations were performed in the Fritz—Haber Institute - *Ab initio* Materials simulations (FHI-aims) package,[11]where the Kohn–Sham (KS) orbitals were expanded in numerical atom-centered orbitals (NAOs).[12] Specifically, we used the light-tier1 basis (adopting FHI-aims terminology), which are the first basis-set improvement from the free-atom orbitals

minimal basis.[20] The electrons were treated within the scalar-relativistic framework using the atomic zeroth-order relativistic approximation (atomic ZORA).[21]

The self-consistent electronic density (SCF) for the complexes was achieved by meeting the following convergence criteria, i.e. a total energy difference of less than 1.00×10^{-4} eV and atomic forces less than 1.00×10^{-2} eV Å^{-1}. Thus, to obtain the equilibrium geometries, we used an atomic force criterion of 1.00×10^{-1} eVÅ^{-1}. Furthermore, to ensure the correct occupation of the electronic states, which is crucial for the complexes, we employed a Gaussian broadening parameter of 10 meV for all total energy calculations.

2.4 `Cluster_Assembler` - Documentation

The `cluster_assembler` package is the workflow implementation used in this work to design the metal complexes within the experimental living library, through DFT *ab initio* calculations combined with machine learning-based algorithms. The source code has been made available to the community under the Apache-2.0 license, accessible at `https://github.com/QTNano/Cluster-Assembler/`. In addition, detailed comments on its functionalities are also provided in the accompanying documentation, which is available at the same link.

1. **Description** – The code provides a general process, divided into four modules (0−3), to generate complexes (core + ligands), i.e., protected clusters. Further details about each step are provided in Section 1 of documentation file.

2. **Recommendations** – Before running the code, the user should check the following observations:

   (a) If the main directory contains all mandatory files and folders: `main.py`, `mol.txt`, `/core`, `/ligands`, `/bin`, and properly prepared the input `parameters.json` file, which is fully-documented at our repository. Do not exclude the **.py* files within these folders if you still pretend to run the code. Additional files as `job_pbs` and `environment.yml` are provided to run `cluster_assembler` on HPC facilities within specified `Python`-modules, respectively;

(b) Check the installation of `Python`;

(c) We strongly recommend installation `Anaconda` possibly via `Anaconda`.

3. **Installation** – Before running the code, the user has to mount all python-modules dependencies required for `cluster_assembler` toolbox run without any problem, by executing the following command:

```
$ conda env create -f environment.yml
```

4. **Running the Code** – The user has to set-up the `parameters.json` input file by specifying all the parameters according to the tasks that `cluster_assembler` will perform. Therefore, we recommend that inexperienced users keep advanced parameters set to their `default` values. However, all parameters can be adjusted as needed. Hence, after setting up the `parameters.json` file, the workflow can be started simply by running the following to commands:

```
$ conda activate py396
```

```
$ python3 main.py parameters.json
```

Note that all folders are created in the same working directory where you called `python3 main.py`. Therefore, when the code or parameter file requires an address, e.g., `INPUT_FOLDER`, the user only needs to type the file's name. However, changing the folder directory requires you to provide the complete path for the address.

5. **Module 0 - Mod0** – *k-means* clustering tool to select the representatives unary cores, binary cores, and complexes. Thus, the original *k*-means clustering algorithm is available at https://github.com/quiles/Adsorption_Clus/blob/main/representative.py. However, a modified and enhanced version of *k-means* is internally implemented within `cluster_assembler` code.

6. **Module 1 - Mod1** – In this module, unary cores are generated, processed, analyzed, and clustered. Therefore, users can choose between combining external sources and internally

generating the unary cores using our implemented subroutine. Subsequently, the cores are checked according to the integrity filter and clustered by *k-means* if the `RUN_MOD_ZERO` flag is activated.

7. **Module 2 - Mod2** – From the unary cores, permutations — i.e., all possible or user-specified —are performed in each of the selected unary cores by an internal subroutine of `cluster_assembler`. Subsequently, the binary cores are checked according to the integrity filter and clustered by *k-means* if the RUN_MOD_ZERO flag is activated.

8. **Module 3 - Mod3** – Module responsible to distribute the ligands around the selected binary cores. Therefore, the ligands file (in $*.xyz$ format) should be prepared beforehand in the `/ligands` folder, particularly when the ligand is oriented distributed. Thus, several ligands might be used in the process. However, their orientation regarding the complex core is controlled by the user, which can select a random or specific orientation (provided by a set of base atoms). An overlap filter iteratively excludes structures with overlapping atoms (integrity). The default threshold is set to t = 1.25, and can be manually tuned. The filtered complexes are again clustered by *k-means* if the RUN_MOD_ZERO flag is activated. Finally, the selected structures are the representatives within the living library family of structures, and can be externally optimized using methods such as DFT, Tight-Binding, etc.

9. **Additional Observations** to use the computational implementation:

    (a) All submission job files for the `cluster_assembler` code at a personal computer (in *job.sh*) and HPC clusters (in *job.pbs*) are provided in the `/jobs` folder;

    (b) Steps 2 and 3 must be done externally and can be automated using appropriate quantum chemistry software, e.g., FHI-aims, CREST, VASP, ADF;

    (c) *k*-means needs the Pandas module needs to work properly. However, we strongly suggest the user install Anaconda since Pandas is already a part of Anaconda

distribution.

2.5 Computational Parameters to Generate the Family of Metal Complexes

Above in the Supplementary Table 10, we provide detailed input parameters for generating the $[A_aB_b](C)_c$ metal complexes' family reported herein. To enhance the unary frames set, we incorporated frames from our previous work,[22] using DFT-optimized cores of $A_{(a+b)}$ and $B_{(b+a)}$ clusters for *INPUT_DATA*. A reduced final family of $[A_aB_b](C)_c$ complexes, containing the representative structures of the living libraries, was achieved by applying the Module 0 (k = 15), depicted on the following section. Based on the physico-chemical properties of this family, additional structures were added to the final set, ensuring a more complete representation of the configuration space by incorporating specific configurations with experimental physic-chemical features.

Supplementary Table 10: Selected input parameters for the `parameters.json` file used as input for the `Cluster_Assembler` code for the generation of the family of $[A_aB_b](C)_c$ metal complexes reported in this work. A reference guide explaining each input parameter can be found at `https://github.com/QTNano/Cluster-Assembler/`. All other parameters were set to their default values.

	Module 1		Module 2				Module 3			
Complexes $[A_aB_b](C)_c$	$A_{(a+b)}$ and $B_{(b+a)}$ frames	Trial $[A_aB_b]$ cores	Representative $[A_aB_b]$ cores	Optimized $[A_aB_b]$ cores	C Orientation	AB – C Distance (Å)	Trial $[A_aB_b](C)_c$ Complexes	Filtered $[A_aB_b](C)_c$ Complexes	Representative $[A_aB_b](C)_c$ Complexes	Final Family of Optimized $[A_aB_b](C)_c$ Complexes
$[CuZn_2](Cp^*)_3$	40	120	40	40	Cp* [0,1,2]	2.25	400	*	20	18
$[Cu_3Zn_4](Cp^*)_5$	21	735	200	198	Cp* [0,1,2]	2.25	1995840	*	300	289
$[Cu_5Zn_5](Cp^*)_6(CO_2)_2$	40	1080	200	179	Cp* [0,1,2]<br>CO_2 [-1]	2.25	2160000	22314	300	208
$[Cu_6Zn_5](Cp^*)_5(Mes)H_3$	54	7798	120	107	Cp* [0,1,2]<br>Mes [11,12,13]<br>H [-1]	2.5	1979500	31873	300	218
$[Cu_8Zn_3](Cp^*)_3(Mes)_4CO_2$	54	8910	300	278	Cp* [0,1,2]<br>Mes [11,12,13]<br>CO_2 [-1]	2.5	2085000	176285	300	209
$[Cu_8Zn_3](Cp^*)_4(Mes)_3H$	54	8910	300	278	Cp* [0,1,2]<br>Mes [11,12,13]<br>H [-1]	2.5	2085000	103278	300	159
$[Cu_9Zn_7](Cp^*)_6(Hex)_3H_3$	200	*	300	291	Cp* [1,3,4]<br>Hex [5,4,3]<br>H [-1]	2.5	2007900	11881	300	43
$[Cu_{11}Zn_6](Cp^*)_8(CO_2)_2(HCO_2)$	60	742560	300	290	Cp* [0,1,2]<br>CO_2 [-1]<br>HCO_2 [-1]	2.5	2001000	3369	300	70
$[Cu_8Al_6](Cp^*)_6$	46	138138	200	197	Cp* [0,1,2]	2.5	2019250	36176	300	213
$[Ni_7Ga_6](Cp^*)_6$	61	104676	200	196	Cp* [0,1,2]	2.5	2009000	20856	300	155

2.6 Additional Results for the A_aB_b Cores

Below, we will report all data obtained for the DFT calculations for the core fragments.

2.6.1 [$CuZn_2$]

Supplementary Table 11: Energetic and electronic properties for [$CuZn_2$] cores obtained with PBE/light-tier1 level and light SCF parameters: configuration number (i), relative total energy (ΔE_{tot}), total magnetic moment (m_{tot}), HOMO energy (ε_H), LUMO energy (ε_L), and LUMO-HOMO energy gap (E_g).

i	ΔE_{tot} (meV)	m_{tot} (μ_B)	ε_H (eV)	ε_L (eV)	E_g (eV)
39	465	1	−4.20	−3.55	0.66
27	461	1	−4.22	−3.55	0.66
8	453	1	−4.27	−3.58	0.69
9	453	1	−4.27	−3.59	0.68
34	435	1	−4.04	−3.41	0.63
5	433	1	−4.41	−3.73	0.68
31	430	1	−4.06	−3.41	0.65
32	418	1	−4.02	−3.40	0.62
16	417	1	−4.34	−3.65	0.69
14	410	1	−3.95	−3.34	0.62
24	382	1	−3.86	−3.27	0.59
19	356	1	−3.83	−3.24	0.59
2	343	1	−3.78	−3.20	0.59
36	176	1	−4.19	−3.59	0.60
10	174	1	−4.20	−3.60	0.60
12	172	1	−4.19	−3.60	0.60
21	158	1	−4.15	−3.55	0.60
20	143	1	−4.11	−3.52	0.59

Continued on next page

Supplementary Table 11: Energetic and electronic properties for [$CuZn_2$] cores obtained with PBE/light-tier1 level and light SCF parameters: configuration number (i), relative total energy (ΔE_{tot}), total magnetic moment (m_{tot}), HOMO energy (ε_H), LUMO energy (ε_L), and LUMO-HOMO energy gap (E_g).

i	ΔE_{tot} (meV)	m_{tot} (μ_B)	ε_H (eV)	ε_L (eV)	E_g (eV)
38	140	1	−4.09	−3.50	0.59
4	139	1	−4.09	−3.50	0.59
13	138	1	−4.08	−3.50	0.59
22	133	1	−4.06	−3.47	0.59
37	131	1	−4.03	−3.44	0.59
7	116	1	−4.00	−3.41	0.59
40	108	1	−3.94	−3.35	0.59
35	106	1	−3.98	−3.39	0.59
26	98	1	−3.93	−3.35	0.59
30	96	1	−3.94	−3.36	0.59
28	90	1	−3.88	−3.29	0.59
33	70	1	−3.86	−3.28	0.59
29	68	1	−3.84	−3.25	0.59
15	41	1	−3.78	−3.19	0.59
1	40	1	−3.81	−3.23	0.58
6	35	1	−3.76	−3.17	0.59
17	34	1	−3.78	−3.21	0.57
23	33	1	−3.73	−3.13	0.60
11	25	1	−3.75	−3.17	0.58
25	10	1	−3.71	−3.12	0.59
18	4	1	−3.71	−3.13	0.58
3	0	1	−3.69	−3.09	0.59

2.6.2 $[Cu_3Zn_4]$

The $[Cu_3Zn_4]$ structures were used to design both $[Cu_3Zn_4](Cp)_5$ and $[Cu_3Zn_4](Cp^*)_5$ complexes.

Supplementary Table 12: Energetic and electronic properties for $[Cu_3Zn_4]$ cores obtained with PBE/light-tier1 level and light SCF parameters: configuration number (i), relative total energy (ΔE_{tot}), total magnetic moment (m_{tot}), HOMO energy (ε_H), LUMO energy (ε_L), and LUMO-HOMO energy gap (E_g).

i	ΔE_{tot} (meV)	m_{tot} (μ_B)	ε_H (eV)	ε_L (eV)	E_g (eV)
34	1485	3	−3.77	−3.62	0.16
32	1483	3	−3.83	−3.62	0.21
153	1418	3	−4.06	−3.75	0.31
117	1414	3	−4.04	−3.71	0.33
158	1409	3	−3.89	−3.75	0.14
43	1326	3	−3.92	−3.81	0.11
188	1277	1	−3.86	−3.73	0.12
189	1254	1	−4.09	−3.78	0.31
108	1222	3	−3.81	−3.66	0.15
148	1210	1	−3.93	−3.53	0.40
111	1205	1	−3.90	−3.51	0.39
186	1111	1	−3.95	−3.59	0.36
120	1061	1	−4.16	−3.77	0.39
151	1060	1	−4.15	−3.76	0.39
49	1059	3	−3.97	−3.77	0.20
55	1035	3	−4.04	−3.74	0.31
8	996	1	−3.74	−3.35	0.39
33	954	3	−3.89	−3.60	0.30
191	940	1	−4.04	−3.75	0.29

Continued on next page

Supplementary Table 12: Energetic and electronic properties for $[Cu_3Zn_4]$ cores obtained with PBE/light-tier1 level and light SCF parameters: configuration number (i), relative total energy (ΔE_{tot}), total magnetic moment (m_{tot}), HOMO energy (ε_H), LUMO energy (ε_L), and LUMO-HOMO energy gap (E_g).

i	ΔE_{tot} (meV)	m_{tot} (μ_B)	ε_H (eV)	ε_L (eV)	E_g (eV)
89	901	1	−4.15	−3.85	0.30
174	888	1	−3.73	−3.37	0.35
199	888	1	−4.38	−4.08	0.29
131	862	1	−3.97	−3.62	0.36
156	860	1	−3.81	−3.51	0.30
1	856	1	−3.92	−3.52	0.40
138	817	1	−4.02	−3.64	0.38
66	809	1	−3.86	−3.48	0.38
5	781	1	−3.94	−3.50	0.45
92	768	1	−3.97	−3.63	0.33
154	768	1	−3.92	−3.51	0.41
197	763	1	−3.66	−3.48	0.18
47	760	1	−3.90	−3.54	0.36
196	712	1	−4.56	−4.29	0.27
143	704	1	−3.84	−3.50	0.34
112	656	1	−4.06	−3.52	0.53
150	653	1	−4.05	−3.52	0.53
100	650	1	−3.88	−3.44	0.44
103	646	1	−3.88	−3.48	0.39
50	646	1	−4.25	−3.86	0.38
162	639	1	−3.54	−3.35	0.19
38	624	1	−3.81	−3.33	0.48

Continued on next page

Supplementary Table 12: Energetic and electronic properties for $[Cu_3Zn_4]$ cores obtained with PBE/light-tier1 level and light SCF parameters: configuration number (i), relative total energy (ΔE_{tot}), total magnetic moment (m_{tot}), HOMO energy (ε_H), LUMO energy (ε_L), and LUMO-HOMO energy gap (E_g).

i	ΔE_{tot} (meV)	m_{tot} (μ_B)	ε_H (eV)	ε_L (eV)	E_g (eV)
152	624	1	−3.96	−3.61	0.35
113	621	1	−3.96	−3.59	0.36
68	617	1	−3.84	−3.44	0.40
90	609	1	−3.80	−3.39	0.41
183	595	1	−3.88	−3.48	0.40
13	592	1	−3.77	−3.37	0.40
37	585	1	−3.84	−3.47	0.37
57	581	1	−3.79	−3.38	0.41
18	571	1	−3.61	−3.31	0.30
161	565	1	−3.92	−3.55	0.37
105	563	1	−4.41	−3.94	0.46
46	559	1	−3.94	−3.55	0.39
130	548	1	−4.09	−3.70	0.39
144	544	1	−3.92	−3.57	0.35
132	526	1	−4.06	−3.69	0.37
133	523	1	−3.87	−3.47	0.41
44	521	1	−3.63	−3.27	0.36
95	521	1	−4.11	−3.74	0.37
72	519	1	−4.16	−3.81	0.36
35	488	1	−4.06	−3.74	0.32
110	487	1	−3.94	−3.56	0.37
75	486	1	−3.97	−3.59	0.37

Continued on next page

Supplementary Table 12: Energetic and electronic properties for [Cu_3Zn_4] cores obtained with PBE/light-tier1 level and light SCF parameters: configuration number (i), relative total energy (ΔE_{tot}), total magnetic moment (m_{tot}), HOMO energy (ε_H), LUMO energy (ε_L), and LUMO-HOMO energy gap (E_g).

i	ΔE_{tot} (meV)	m_{tot} (μ_B)	ε_H (eV)	ε_L (eV)	E_g (eV)
54	482	1	−3.87	−3.50	0.37
184	480	1	−4.10	−3.70	0.40
147	478	1	−3.92	−3.55	0.37
106	475	1	−4.17	−3.78	0.39
78	472	1	−3.90	−3.56	0.34
195	471	1	−3.78	−3.39	0.39
194	469	1	−4.00	−3.66	0.34
185	468	1	−4.03	−3.69	0.35
22	461	1	−4.06	−3.76	0.30
134	460	1	−3.87	−3.49	0.39
177	459	1	−4.14	−3.75	0.39
198	458	1	−4.14	−3.76	0.38
79	457	1	−3.88	−3.51	0.37
142	455	1	−3.96	−3.59	0.38
64	452	1	−4.06	−3.61	0.45
71	452	1	−3.94	−3.57	0.37
187	445	1	−3.88	−3.49	0.39
31	445	1	−3.89	−3.45	0.44
15	440	1	−3.89	−3.45	0.43
67	439	1	−3.89	−3.48	0.41
168	437	1	−3.98	−3.60	0.38
170	432	1	−3.91	−3.50	0.41

Continued on next page

Supplementary Table 12: Energetic and electronic properties for [Cu_3Zn_4] cores obtained with PBE/light-tier1 level and light SCF parameters: configuration number (i), relative total energy (ΔE_{tot}), total magnetic moment (m_{tot}), HOMO energy (ε_H), LUMO energy (ε_L), and LUMO-HOMO energy gap (E_g).

i	ΔE_{tot} (meV)	m_{tot} (μ_B)	ε_H (eV)	ε_L (eV)	E_g (eV)
48	424	1	−3.90	−3.51	0.39
167	420	1	−3.89	−3.51	0.37
73	417	1	−3.73	−3.30	0.44
122	417	1	−3.80	−3.35	0.45
176	407	1	−4.30	−3.93	0.37
107	406	1	−3.71	−3.28	0.43
180	406	1	−3.69	−3.26	0.43
69	399	1	−4.06	−3.54	0.52
7	395	1	−3.91	−3.52	0.38
86	393	1	−4.15	−3.74	0.41
159	387	1	−3.53	−3.12	0.41
42	358	1	−3.93	−3.50	0.44
98	355	1	−4.14	−3.72	0.41
11	354	1	−3.91	−3.62	0.29
115	353	1	−3.96	−3.66	0.30
93	348	1	−3.75	−3.37	0.38
146	342	1	−4.12	−3.80	0.32
172	335	1	−3.81	−3.43	0.38
91	334	1	−4.12	−3.79	0.32
114	334	1	−4.03	−3.72	0.31
3	330	1	−3.90	−3.59	0.31
129	329	1	−3.83	−3.41	0.42

Continued on next page

Supplementary Table 12: Energetic and electronic properties for [Cu_3Zn_4] cores obtained with PBE/light-tier1 level and light SCF parameters: configuration number (i), relative total energy (ΔE_{tot}), total magnetic moment (m_{tot}), HOMO energy (ε_H), LUMO energy (ε_L), and LUMO-HOMO energy gap (E_g).

i	ΔE_{tot} (meV)	m_{tot} (μ_B)	ε_H (eV)	ε_L (eV)	E_g (eV)
61	329	1	−3.82	−3.44	0.38
40	325	1	−3.60	−3.21	0.39
63	317	1	−4.02	−3.63	0.39
165	309	1	−3.76	−3.35	0.41
136	309	1	−3.65	−3.26	0.39
76	305	1	−3.87	−3.45	0.43
96	305	1	−4.01	−3.61	0.39
155	301	1	−3.64	−3.19	0.46
23	300	1	−4.02	−3.61	0.41
88	298	1	−4.08	−3.67	0.42
6	287	1	−4.02	−3.49	0.53
128	285	1	−3.86	−3.48	0.38
59	284	1	−3.76	−3.36	0.40
135	283	1	−3.85	−3.41	0.44
81	283	1	−3.82	−3.39	0.44
99	275	1	−3.73	−3.33	0.40
173	275	1	−3.58	−3.10	0.48
65	268	1	−3.76	−3.37	0.39
124	267	1	−3.87	−3.46	0.42
118	265	1	−3.83	−3.44	0.39
140	263	1	−3.46	−3.04	0.42
45	261	1	−3.67	−3.24	0.43

Continued on next page

Supplementary Table 12: Energetic and electronic properties for [Cu_3Zn_4] cores obtained with PBE/light-tier1 level and light SCF parameters: configuration number (i), relative total energy (ΔE_{tot}), total magnetic moment (m_{tot}), HOMO energy (ε_H), LUMO energy (ε_L), and LUMO-HOMO energy gap (E_g).

i	ΔE_{tot} (meV)	m_{tot} (μ_B)	ε_H (eV)	ε_L (eV)	E_g (eV)
82	261	1	−3.81	−3.37	0.44
52	260	1	−3.73	−3.35	0.38
9	258	1	−3.86	−3.43	0.42
41	256	1	−3.68	−3.26	0.42
83	256	1	−3.71	−3.28	0.42
53	249	1	−3.70	−3.26	0.43
85	248	1	−3.76	−3.36	0.40
179	248	1	−3.78	−3.33	0.45
97	246	1	−3.84	−3.40	0.44
39	243	1	−3.76	−3.41	0.35
166	243	1	−3.59	−3.12	0.46
51	227	1	−3.87	−3.47	0.40
164	226	1	−3.73	−3.36	0.36
181	225	1	−3.71	−3.35	0.36
182	224	1	−3.61	−3.12	0.49
21	224	1	−3.94	−3.52	0.41
169	224	1	−3.73	−3.29	0.44
24	220	1	−3.89	−3.45	0.44
4	218	1	−3.94	−3.52	0.42
163	214	1	−3.91	−3.50	0.41
30	211	1	−3.83	−3.40	0.42
178	203	1	−3.90	−3.46	0.43

Continued on next page

Supplementary Table 12: Energetic and electronic properties for $[Cu_3Zn_4]$ cores obtained with PBE/light-tier1 level and light SCF parameters: configuration number (i), relative total energy (ΔE_{tot}), total magnetic moment (m_{tot}), HOMO energy (ε_H), LUMO energy (ε_L), and LUMO-HOMO energy gap (E_g).

i	ΔE_{tot} (meV)	m_{tot} (μ_B)	ε_H (eV)	ε_L (eV)	E_g (eV)
102	196	1	−3.89	−3.45	0.44
84	191	1	−3.83	−3.38	0.45
109	185	1	−3.83	−3.40	0.43
10	184	1	−3.83	−3.40	0.42
28	181	1	−3.83	−3.40	0.43
20	181	1	−3.86	−3.43	0.43
27	161	1	−3.93	−3.48	0.46
145	154	1	−3.81	−3.43	0.39
17	152	1	−4.05	−3.57	0.48
56	144	1	−3.72	−3.30	0.42
101	142	1	−3.69	−3.27	0.42
29	142	1	−3.85	−3.34	0.51
14	139	1	−4.07	−3.62	0.45
175	138	1	−3.69	−3.28	0.42
123	132	1	−3.84	−3.39	0.45
157	130	1	−3.76	−3.37	0.39
193	128	1	−3.76	−3.36	0.40
116	126	1	−3.78	−3.39	0.39
126	121	1	−3.76	−3.37	0.39
192	117	1	−3.76	−3.37	0.39
119	113	1	−3.74	−3.35	0.39
149	112	1	−3.75	−3.36	0.39

Continued on next page

Supplementary Table 12: Energetic and electronic properties for $[Cu_3Zn_4]$ cores obtained with PBE/light-tier1 level and light SCF parameters: configuration number (i), relative total energy (ΔE_{tot}), total magnetic moment (m_{tot}), HOMO energy (ε_H), LUMO energy (ε_L), and LUMO-HOMO energy gap (E_g).

i	ΔE_{tot} (meV)	m_{tot} (μ_B)	ε_H (eV)	ε_L (eV)	E_g (eV)
2	112	1	−3.93	−3.44	0.49
104	111	1	−3.81	−3.37	0.44
139	111	1	−4.04	−3.63	0.41
74	109	1	−3.81	−3.37	0.44
160	109	1	−3.84	−3.32	0.51
127	107	1	−3.81	−3.37	0.44
94	106	1	−4.02	−3.62	0.40
12	105	1	−3.80	−3.35	0.44
62	104	1	−3.80	−3.36	0.44
36	104	1	−3.80	−3.36	0.44
80	102	1	−3.92	−3.52	0.40
77	92	1	−3.97	−3.59	0.38
16	85	1	−3.73	−3.18	0.55
26	82	1	−3.74	−3.19	0.55
60	80	1	−3.99	−3.61	0.37
137	62	1	−3.96	−3.58	0.38
87	11	1	−3.82	−3.37	0.45
19	8	1	−3.79	−3.33	0.45
190	4	1	−3.80	−3.35	0.45
70	3	1	−3.78	−3.32	0.46
58	2	1	−3.80	−3.34	0.46
25	2	1	−3.78	−3.32	0.46

Continued on next page

Supplementary Table 12: Energetic and electronic properties for [Cu_3Zn_4] cores obtained with PBE/light-tier1 level and light SCF parameters: configuration number (i), relative total energy (ΔE_{tot}), total magnetic moment (m_{tot}), HOMO energy (ε_H), LUMO energy (ε_L), and LUMO-HOMO energy gap (E_g).

i	ΔE_{tot} (meV)	m_{tot} (μ_B)	ε_H (eV)	ε_L (eV)	E_g (eV)
171	1	1	−3.80	−3.35	0.46
141	1	1	−3.79	−3.33	0.46
125	0	1	−3.78	−3.32	0.46

2.6.3 [Cu_5Zn_5]

Supplementary Table 13: Energetic and electronic properties for [Cu_5Zn_5] cores obtained with PBE/light-tier1 level and light SCF parameters: configuration number (i), relative total energy (ΔE_{tot}), total magnetic moment (m_{tot}), HOMO energy (ε_H), LUMO energy (ε_L), and LUMO-HOMO energy gap (E_g).

i	ΔE_{tot} (meV)	m_{tot} (μ_B)	ε_H (eV)	ε_L (eV)	E_g (eV)
96	1803	3	−3.99	−3.88	0.11
97	1708	3	−4.02	−3.92	0.10
94	1588	3	−4.07	−3.87	0.20
178	1368	1	−3.85	−3.56	0.28
140	1319	3	−4.09	−3.78	0.31
12	1277	3	−4.09	−3.93	0.16
30	1203	3	−4.10	−3.99	0.12
177	1192	1	−3.74	−3.48	0.25
139	1075	1	−4.02	−3.74	0.28
176	1046	1	−3.76	−3.46	0.30
32	1016	1	−4.11	−4.04	0.07

Continued on next page

Supplementary Table 13: Energetic and electronic properties for $[Cu_5Zn_5]$ cores obtained with PBE/light-tier1 level and light SCF parameters: configuration number (i), relative total energy (ΔE_{tot}), total magnetic moment (m_{tot}), HOMO energy (ε_H), LUMO energy (ε_L), and LUMO-HOMO energy gap (E_g).

i	ΔE_{tot} (meV)	m_{tot} (μ_B)	ε_H (eV)	ε_L (eV)	E_g (eV)
68	1012	1	−3.95	−3.57	0.38
172	964	1	−3.96	−3.67	0.29
46	963	1	−4.04	−3.76	0.28
11	954	1	−4.15	−3.87	0.27
45	949	1	−3.92	−3.64	0.29
145	938	1	−3.91	−3.61	0.30
36	930	1	−3.75	−3.34	0.41
173	923	1	−4.22	−3.93	0.29
175	922	1	−3.95	−3.69	0.26
91	911	1	−4.21	−3.92	0.29
78	899	1	−3.98	−3.61	0.36
153	892	1	−3.88	−3.62	0.26
60	865	1	−4.12	−3.87	0.25
73	862	1	−3.83	−3.52	0.31
13	858	3	−4.21	−4.07	0.14
155	847	1	−3.77	−3.44	0.33
114	841	1	−3.91	−3.67	0.24
160	830	1	−4.25	−3.93	0.31
82	807	1	−4.30	−4.03	0.27
24	800	1	−4.18	−3.87	0.31
26	799	1	−4.13	−3.78	0.35
93	787	1	−4.04	−3.74	0.31

Continued on next page

Supplementary Table 13: Energetic and electronic properties for [Cu_5Zn_5] cores obtained with PBE/light-tier1 level and light SCF parameters: configuration number (i), relative total energy (ΔE_{tot}), total magnetic moment (m_{tot}), HOMO energy (ε_H), LUMO energy (ε_L), and LUMO-HOMO energy gap (E_g).

i	ΔE_{tot} (meV)	m_{tot} (μ_B)	ε_H (eV)	ε_L (eV)	E_g (eV)
22	783	1	−4.03	−3.74	0.29
144	776	1	−3.90	−3.58	0.32
95	750	1	−3.89	−3.57	0.31
119	749	1	−4.13	−3.83	0.29
72	746	1	−4.04	−3.78	0.27
70	742	1	−3.94	−3.63	0.32
41	739	1	−3.85	−3.58	0.27
66	730	1	−4.07	−3.74	0.33
133	720	1	−4.20	−3.95	0.25
25	682	1	−4.10	−3.79	0.30
74	679	1	−4.11	−3.77	0.34
100	677	1	−4.02	−3.74	0.28
143	677	1	−4.14	−3.85	0.28
105	670	1	−3.66	−3.39	0.28
87	662	1	−4.14	−3.84	0.30
54	652	1	−4.14	−3.84	0.30
169	643	1	−3.74	−3.56	0.18
8	639	1	−4.30	−4.06	0.24
99	636	1	−4.18	−3.89	0.29
174	634	1	−4.05	−3.75	0.30
80	627	1	−4.18	−3.87	0.31
122	624	1	−3.89	−3.55	0.34

Continued on next page

Supplementary Table 13: Energetic and electronic properties for [Cu_5Zn_5] cores obtained with PBE/light-tier1 level and light SCF parameters: configuration number (i), relative total energy (ΔE_{tot}), total magnetic moment (m_{tot}), HOMO energy (ε_H), LUMO energy (ε_L), and LUMO-HOMO energy gap (E_g).

i	ΔE_{tot} (meV)	m_{tot} (μ_B)	ε_H (eV)	ε_L (eV)	E_g (eV)
149	617	1	−3.74	−3.42	0.32
27	611	1	−4.14	−3.87	0.27
152	608	1	−4.02	−3.74	0.28
37	608	1	−3.88	−3.56	0.33
115	608	1	−4.03	−3.73	0.30
121	605	1	−4.13	−3.82	0.32
179	601	1	−3.78	−3.46	0.32
5	600	1	−4.01	−3.66	0.35
180	597	1	−3.89	−3.61	0.29
39	596	1	−3.97	−3.72	0.25
165	596	1	−3.88	−3.60	0.27
7	590	1	−4.22	−3.93	0.29
132	586	1	−4.16	−3.87	0.29
147	583	1	−3.74	−3.44	0.30
120	579	1	−4.08	−3.75	0.33
104	579	1	−4.09	−3.76	0.33
137	578	1	−3.90	−3.64	0.26
161	578	1	−4.28	−4.00	0.28
103	575	1	−3.81	−3.53	0.28
75	572	1	−4.06	−3.68	0.38
16	567	1	−3.89	−3.76	0.13
18	566	1	−4.23	−3.94	0.28

Continued on next page

Supplementary Table 13: Energetic and electronic properties for [Cu_5Zn_5] cores obtained with PBE/light-tier1 level and light SCF parameters: configuration number (i), relative total energy (ΔE_{tot}), total magnetic moment (m_{tot}), HOMO energy (ε_H), LUMO energy (ε_L), and LUMO-HOMO energy gap (E_g).

i	ΔE_{tot} (meV)	m_{tot} (μ_B)	ε_H (eV)	ε_L (eV)	E_g (eV)
90	564	1	−4.09	−3.78	0.32
163	562	1	−3.83	−3.51	0.33
123	558	1	−4.07	−3.79	0.28
156	558	1	−3.83	−3.56	0.27
23	554	1	−4.08	−3.77	0.32
77	553	1	−4.04	−3.66	0.38
1	550	1	−4.06	−3.79	0.26
76	549	1	−4.03	−3.68	0.36
171	545	1	−3.90	−3.61	0.29
62	537	1	−3.99	−3.63	0.37
98	536	1	−3.90	−3.59	0.30
108	529	1	−4.00	−3.70	0.31
124	526	1	−3.80	−3.51	0.29
47	509	1	−4.37	−4.09	0.28
159	509	1	−4.10	−3.81	0.29
112	508	1	−3.78	−3.44	0.34
92	508	1	−4.01	−3.74	0.27
71	505	1	−4.12	−3.76	0.36
128	503	1	−4.39	−4.12	0.27
9	502	1	−3.88	−3.54	0.34
83	499	1	−4.13	−3.84	0.30
157	496	1	−3.83	−3.50	0.33

Continued on next page

Supplementary Table 13: Energetic and electronic properties for $[Cu_5Zn_5]$ cores obtained with PBE/light-tier1 level and light SCF parameters: configuration number (i), relative total energy (ΔE_{tot}), total magnetic moment (m_{tot}), HOMO energy (ε_H), LUMO energy (ε_L), and LUMO-HOMO energy gap (E_g).

i	ΔE_{tot} (meV)	m_{tot} (μ_B)	ε_H (eV)	ε_L (eV)	E_g (eV)
35	495	1	−4.03	−3.70	0.33
135	494	1	−4.14	−3.87	0.26
158	490	1	−4.05	−3.76	0.29
106	489	1	−4.15	−3.83	0.33
43	489	1	−3.81	−3.51	0.30
69	486	1	−4.03	−3.74	0.29
3	485	1	−4.02	−3.68	0.34
34	481	1	−3.73	−3.43	0.30
129	479	1	−4.25	−3.99	0.27
134	477	1	−4.24	−3.98	0.26
50	476	1	−4.23	−3.97	0.27
164	470	1	−3.88	−3.61	0.26
146	466	1	−3.83	−3.48	0.35
17	458	1	−4.04	−3.77	0.27
40	454	1	−3.88	−3.58	0.30
101	453	1	−3.76	−3.43	0.33
136	449	1	−4.01	−3.69	0.32
117	434	1	−3.89	−3.59	0.31
131	433	1	−3.97	−3.66	0.31
52	429	1	−3.97	−3.64	0.33
21	428	1	−4.13	−3.76	0.37
142	428	1	−3.89	−3.57	0.32

Continued on next page

Supplementary Table 13: Energetic and electronic properties for $[Cu_5Zn_5]$ cores obtained with PBE/light-tier1 level and light SCF parameters: configuration number (i), relative total energy (ΔE_{tot}), total magnetic moment (m_{tot}), HOMO energy (ε_H), LUMO energy (ε_L), and LUMO-HOMO energy gap (E_g).

i	ΔE_{tot} (meV)	m_{tot} (μ_B)	ε_H (eV)	ε_L (eV)	E_g (eV)
84	427	1	−4.21	−3.96	0.25
53	423	1	−4.14	−3.77	0.38
20	420	1	−3.79	−3.48	0.32
130	413	1	−4.22	−3.95	0.27
42	411	1	−4.04	−3.63	0.41
56	398	1	−3.93	−3.58	0.36
138	397	1	−3.98	−3.69	0.29
64	396	1	−3.97	−3.64	0.33
15	394	1	−3.90	−3.58	0.32
148	386	1	−3.85	−3.56	0.30
79	385	1	−3.93	−3.61	0.32
150	379	1	−3.98	−3.65	0.33
89	375	1	−3.80	−3.53	0.28
167	372	1	−3.89	−3.61	0.28
65	369	1	−3.76	−3.45	0.31
38	367	1	−4.17	−3.84	0.32
4	364	1	−3.86	−3.48	0.38
67	357	1	−4.16	−3.80	0.36
151	355	1	−4.16	−3.87	0.30
125	355	1	−4.03	−3.70	0.33
2	350	1	−3.94	−3.60	0.34
141	346	1	−3.89	−3.57	0.32

Continued on next page

Supplementary Table 13: Energetic and electronic properties for [Cu_5Zn_5] cores obtained with PBE/light-tier1 level and light SCF parameters: configuration number (i), relative total energy (ΔE_{tot}), total magnetic moment (m_{tot}), HOMO energy (ε_H), LUMO energy (ε_L), and LUMO-HOMO energy gap (E_g).

i	ΔE_{tot} (meV)	m_{tot} (μ_B)	ε_H (eV)	ε_L (eV)	E_g (eV)
88	344	1	−3.95	−3.65	0.30
166	341	1	−3.78	−3.50	0.28
29	337	1	−3.89	−3.58	0.31
86	331	1	−4.05	−3.78	0.27
28	328	1	−3.91	−3.63	0.28
102	327	1	−4.13	−3.82	0.31
63	318	1	−3.95	−3.64	0.31
110	307	1	−4.13	−3.86	0.28
85	300	1	−4.10	−3.79	0.31
58	299	1	−3.88	−3.53	0.36
116	298	1	−4.05	−3.78	0.28
55	294	1	−4.02	−3.68	0.34
154	291	1	−3.98	−3.68	0.30
33	291	1	−4.11	−3.81	0.30
168	284	1	−3.76	−3.45	0.30
170	280	1	−3.79	−3.48	0.31
127	279	1	−4.01	−3.75	0.25
113	279	1	−3.77	−3.47	0.30
31	252	1	−3.90	−3.59	0.31
19	243	1	−4.02	−3.70	0.32
107	241	1	−3.85	−3.55	0.30
126	200	1	−3.89	−3.59	0.31

Continued on next page

Supplementary Table 13: Energetic and electronic properties for [Cu_5Zn_5] cores obtained with PBE/light-tier1 level and light SCF parameters: configuration number (i), relative total energy (ΔE_{tot}), total magnetic moment (m_{tot}), HOMO energy (ε_H), LUMO energy (ε_L), and LUMO-HOMO energy gap (E_g).

i	ΔE_{tot} (meV)	m_{tot} (μ_B)	ε_H (eV)	ε_L (eV)	E_g (eV)
48	195	1	−4.05	−3.75	0.29
118	194	1	−3.82	−3.51	0.32
111	185	1	−3.84	−3.50	0.34
44	183	1	−4.06	−3.75	0.31
57	181	1	−3.81	−3.52	0.29
14	179	1	−3.90	−3.61	0.29
61	174	1	−3.68	−3.35	0.33
51	155	1	−4.18	−3.89	0.30
6	150	1	−3.96	−3.62	0.34
10	148	1	−3.83	−3.47	0.35
109	146	1	−3.97	−3.65	0.32
59	135	1	−3.69	−3.35	0.34
49	134	1	−4.10	−3.81	0.29
81	107	1	−3.86	−3.51	0.35
162	0	1	−3.88	−3.54	0.34

2.6.4 [Cu_6Zn_5]

Supplementary Table 14: Energetic and electronic properties for $[Cu_6Zn_5]$ cores obtained with PBE/light-tier1 level and light SCF parameters: configuration number (i), relative total energy (ΔE_{tot}), total magnetic moment (m_{tot}), HOMO energy (ε_H), LUMO energy (ε_L), and LUMO-HOMO energy gap (E_g).

i	ΔE_{tot} (meV)	m_{tot} (μ_B)	ε_H (eV)	ε_L (eV)	E_g (eV)
99	2580	2	−4.17	−4.00	0.17
52	1584	2	−3.98	−3.88	0.10
30	1549	2	−3.96	−3.67	0.29
47	1530	2	−3.73	−3.62	0.10
115	1503	0	−4.40	−3.13	1.27
100	1464	2	−3.84	−3.56	0.27
97	1455	2	−4.05	−3.83	0.21
28	1420	2	−3.81	−3.60	0.21
116	1397	0	−4.21	−3.10	1.11
33	1360	2	−4.12	−3.89	0.23
34	1349	0	−4.14	−4.03	0.11
96	1339	2	−4.04	−3.85	0.19
87	1338	2	−4.24	−4.06	0.18
11	1325	2	−3.92	−3.82	0.10
37	1290	0	−4.24	−3.89	0.35
36	1262	0	−4.26	−4.03	0.23
118	1246	2	−4.01	−3.76	0.25
117	1221	0	−4.22	−3.13	1.09
5	1200	2	−4.16	−3.80	0.36
75	1161	2	−3.80	−3.71	0.09
39	1158	0	−4.24	−3.62	0.62
114	1151	0	−4.29	−3.03	1.27

Continued on next page

Supplementary Table 14: Energetic and electronic properties for [Cu_6Zn_5] cores obtained with PBE/light-tier1 level and light SCF parameters: configuration number (i), relative total energy (ΔE_{tot}), total magnetic moment (m_{tot}), HOMO energy (ε_H), LUMO energy (ε_L), and LUMO-HOMO energy gap (E_g).

i	ΔE_{tot} (meV)	m_{tot} (μ_B)	ε_H (eV)	ε_L (eV)	E_g (eV)
88	1144	0	−4.36	−3.86	0.50
32	1129	2	−4.07	−3.85	0.23
20	1125	2	−4.26	−4.12	0.14
35	1121	2	−4.26	−4.05	0.21
103	1107	2	−3.90	−3.75	0.15
66	1098	2	−4.06	−3.76	0.30
53	1098	0	−4.48	−3.70	0.78
23	1084	2	−4.00	−3.89	0.12
79	1083	0	−4.43	−3.50	0.93
106	1074	0	−4.45	−3.78	0.67
63	1011	0	−4.31	−3.29	1.02
78	996	2	−4.17	−3.98	0.20
60	968	0	−4.34	−3.86	0.48
86	955	0	−4.29	−3.94	0.35
8	955	0	−4.29	−3.60	0.69
68	951	2	−4.01	−3.89	0.12
51	937	0	−4.40	−3.63	0.77
29	936	0	−4.30	−3.25	1.06
69	933	2	−4.06	−3.82	0.25
9	924	0	−4.18	−3.66	0.53
77	923	0	−4.15	−3.31	0.84
62	910	0	−4.33	−3.35	0.98

Continued on next page

Supplementary Table 14: Energetic and electronic properties for [Cu_6Zn_5] cores obtained with PBE/light-tier1 level and light SCF parameters: configuration number (i), relative total energy (ΔE_{tot}), total magnetic moment (m_{tot}), HOMO energy (ε_H), LUMO energy (ε_L), and LUMO-HOMO energy gap (E_g).

i	ΔE_{tot} (meV)	m_{tot} (μ_B)	ε_H (eV)	ε_L (eV)	E_g (eV)
55	858	2	−4.09	−3.80	0.29
119	853	0	−4.44	−3.59	0.85
45	853	2	−4.03	−3.92	0.12
17	850	0	−4.43	−3.33	1.11
43	838	0	−4.42	−3.88	0.55
73	836	0	−4.31	−3.30	1.01
21	834	0	−4.40	−3.55	0.85
104	831	0	−4.32	−3.31	1.00
26	829	2	−4.15	−4.06	0.09
90	825	0	−4.24	−3.04	1.20
85	810	0	−4.39	−3.81	0.58
95	810	2	−4.06	−3.86	0.20
89	809	2	−4.10	−3.97	0.12
80	804	0	−4.46	−3.65	0.81
15	803	0	−4.11	−3.48	0.62
16	802	0	−4.13	−3.49	0.64
102	778	0	−4.35	−3.14	1.21
4	754	0	−4.10	−3.17	0.93
93	751	0	−4.22	−3.11	1.12
50	742	0	−4.20	−3.71	0.49
101	730	0	−4.34	−3.10	1.24
111	718	0	−4.43	−3.25	1.18

Continued on next page

Supplementary Table 14: Energetic and electronic properties for [Cu_6Zn_5] cores obtained with PBE/light-tier1 level and light SCF parameters: configuration number (i), relative total energy (ΔE_{tot}), total magnetic moment (m_{tot}), HOMO energy (ε_H), LUMO energy (ε_L), and LUMO-HOMO energy gap (E_g).

i	ΔE_{tot} (meV)	m_{tot} (μ_B)	ε_H (eV)	ε_L (eV)	E_g (eV)
72	716	0	−4.32	−3.36	0.96
6	706	0	−4.40	−3.15	1.25
18	693	0	−4.30	−3.47	0.84
3	688	0	−4.29	−3.75	0.55
48	678	0	−4.41	−3.25	1.16
61	675	0	−4.21	−3.20	1.01
64	663	0	−4.32	−3.75	0.57
76	658	0	−4.18	−3.26	0.92
98	652	0	−4.28	−3.71	0.57
105	631	0	−4.22	−3.13	1.10
1	628	0	−4.35	−3.78	0.57
56	617	0	−4.44	−3.36	1.08
31	606	0	−4.46	−3.47	0.99
41	594	2	−4.14	−3.87	0.27
57	590	0	−4.35	−3.41	0.94
12	588	0	−4.17	−3.87	0.31
25	571	0	−4.28	−3.63	0.65
59	548	0	−4.47	−3.64	0.84
91	542	0	−4.17	−3.58	0.59
40	540	2	−4.22	−4.09	0.13
42	533	0	−4.23	−3.72	0.51
82	497	0	−4.28	−3.71	0.57

Continued on next page

Supplementary Table 14: Energetic and electronic properties for $[Cu_6Zn_5]$ cores obtained with PBE/light-tier1 level and light SCF parameters: configuration number (i), relative total energy (ΔE_{tot}), total magnetic moment (m_{tot}), HOMO energy (ε_H), LUMO energy (ε_L), and LUMO-HOMO energy gap (E_g).

i	ΔE_{tot} (meV)	m_{tot} (μ_B)	ε_H (eV)	ε_L (eV)	E_g (eV)
38	494	0	−4.32	−3.39	0.93
19	449	0	−4.34	−3.23	1.11
22	449	0	−4.39	−3.55	0.84
109	445	0	−4.47	−3.04	1.43
10	438	0	−4.38	−3.28	1.11
112	397	0	−4.41	−3.33	1.07
70	388	2	−4.04	−3.77	0.27
107	382	0	−4.34	−3.68	0.66
92	367	0	−4.26	−3.15	1.11
65	332	0	−4.14	−3.84	0.30
67	285	0	−4.27	−3.69	0.59
71	277	0	−4.46	−3.40	1.06
24	259	0	−4.42	−3.78	0.64
44	171	0	−4.35	−3.77	0.58
94	160	0	−4.57	−3.81	0.76
108	105	0	−4.39	−2.92	1.47
2	83	0	−4.56	−3.83	0.73
13	66	0	−4.31	−3.80	0.52
14	26	0	−4.42	−3.84	0.58
7	0	0	−4.43	−3.73	0.70

2.6.5 $[Cu_8Zn_3]$

The $[Cu_8Zn_3]$ structures were used to design both $[Cu_8Zn_3](Cp^*)_3(Mes)_4CO_2$ and $[Cu_8Zn_3](Cp^*)_4(Mes)_3H$ complexes.

Supplementary Table 15: Energetic and electronic properties for $[Cu_8Zn_3]$ cores obtained with PBE/light-tier1 level and light SCF parameters: configuration number (i), relative total energy (ΔE_{tot}), total magnetic moment (m_{tot}), HOMO energy (ε_H), LUMO energy (ε_L), and LUMO-HOMO energy gap (E_g).

i	ΔE_{tot} (meV)	m_{tot} (μ_B)	ε_H (eV)	ε_L (eV)	E_g (eV)
161	2299	4	−4.00	−3.94	0.06
109	2147	2	−4.15	−3.93	0.22
181	2134	4	−3.95	−3.80	0.14
165	1876	2	−3.98	−3.75	0.23
230	1867	2	−3.94	−3.66	0.28
106	1730	2	−4.25	−3.96	0.29
220	1726	4	−3.87	−3.67	0.20
87	1718	4	−4.11	−4.02	0.09
209	1711	4	−4.12	−4.01	0.11
239	1710	2	−4.15	−3.93	0.22
179	1693	2	−3.92	−3.69	0.23
160	1691	2	−3.94	−3.64	0.30
243	1666	2	−4.11	−3.93	0.18
216	1611	4	−3.88	−3.70	0.18
108	1597	0	−4.10	−3.85	0.25
86	1580	2	−4.13	−3.98	0.15
274	1575	2	−3.86	−3.66	0.20
279	1565	2	−4.03	−3.94	0.09
259	1565	2	−3.80	−3.53	0.27

Continued on next page

Supplementary Table 15: Energetic and electronic properties for $[Cu_8Zn_3]$ cores obtained with PBE/light-tier1 level and light SCF parameters: configuration number (i), relative total energy (ΔE_{tot}), total magnetic moment (m_{tot}), HOMO energy (ε_H), LUMO energy (ε_L), and LUMO-HOMO energy gap (E_g).

i	ΔE_{tot} (meV)	m_{tot} (μ_B)	ε_H (eV)	ε_L (eV)	E_g (eV)
215	1556	0	−4.19	−3.50	0.69
89	1555	2	−4.12	−3.99	0.13
159	1550	2	−3.85	−3.59	0.26
144	1544	2	−3.77	−3.68	0.10
81	1535	2	−4.06	−3.81	0.25
158	1498	2	−3.91	−3.67	0.25
253	1495	2	−3.77	−3.69	0.08
162	1491	2	−3.77	−3.70	0.07
227	1471	2	−3.97	−3.65	0.32
265	1446	0	−4.19	−3.56	0.63
57	1437	2	−4.04	−3.97	0.07
260	1433	2	−3.81	−3.74	0.07
282	1428	0	−4.06	−3.19	0.87
222	1417	2	−3.91	−3.75	0.16
110	1413	2	−4.05	−3.93	0.12
241	1399	2	−3.90	−3.77	0.12
180	1377	2	−4.06	−3.76	0.29
262	1373	2	−3.75	−3.61	0.15
84	1369	2	−4.11	−3.79	0.32
22	1369	2	−4.13	−3.82	0.31
248	1367	2	−3.66	−3.42	0.24
199	1366	2	−3.93	−3.67	0.26

Continued on next page

Supplementary Table 15: Energetic and electronic properties for $[Cu_8Zn_3]$ cores obtained with PBE/light-tier1 level and light SCF parameters: configuration number (i), relative total energy (ΔE_{tot}), total magnetic moment (m_{tot}), HOMO energy (ε_H), LUMO energy (ε_L), and LUMO-HOMO energy gap (E_g).

i	ΔE_{tot} (meV)	m_{tot} (μ_B)	ε_H (eV)	ε_L (eV)	E_g (eV)
212	1365	2	−4.12	−3.83	0.29
82	1361	2	−4.08	−3.84	0.24
252	1357	0	−4.38	−3.58	0.80
41	1355	2	−3.89	−3.76	0.13
261	1344	2	−3.84	−3.70	0.13
147	1317	2	−3.80	−3.71	0.10
278	1290	2	−3.90	−3.72	0.18
76	1274	2	−4.03	−3.95	0.08
39	1266	2	−3.96	−3.92	0.03
242	1264	2	−3.90	−3.80	0.10
290	1259	2	−3.83	−3.69	0.14
68	1251	2	−3.83	−3.72	0.11
130	1248	0	−4.12	−3.77	0.35
257	1222	0	−4.10	−3.28	0.83
271	1210	0	−4.18	−3.72	0.46
119	1193	2	−3.84	−3.70	0.14
145	1184	2	−3.83	−3.71	0.12
83	1179	0	−4.07	−3.58	0.49
213	1175	0	−4.08	−3.57	0.50
66	1170	0	−4.11	−3.60	0.52
131	1166	0	−4.18	−3.58	0.60
67	1151	2	−3.93	−3.82	0.11

Continued on next page

Supplementary Table 15: Energetic and electronic properties for $[Cu_8Zn_3]$ cores obtained with PBE/light-tier1 level and light SCF parameters: configuration number (i), relative total energy (ΔE_{tot}), total magnetic moment (m_{tot}), HOMO energy (ε_H), LUMO energy (ε_L), and LUMO-HOMO energy gap (E_g).

i	ΔE_{tot} (meV)	m_{tot} (μ_B)	ε_H (eV)	ε_L (eV)	E_g (eV)
75	1149	2	−4.07	−3.97	0.10
34	1127	2	−4.21	−4.08	0.14
263	1126	0	−4.17	−3.25	0.92
185	1114	0	−4.15	−3.34	0.80
122	1112	2	−3.91	−3.61	0.30
146	1109	2	−3.98	−3.72	0.26
56	1103	0	−4.15	−3.56	0.59
286	1098	0	−4.65	−3.30	1.35
121	1072	2	−3.87	−3.63	0.24
25	1052	2	−4.09	−3.98	0.11
28	1050	2	−4.12	−3.90	0.22
156	1048	2	−3.83	−3.77	0.06
143	1042	2	−3.83	−3.60	0.24
182	1040	0	−4.03	−3.41	0.62
35	1031	2	−4.14	−4.05	0.09
92	1029	2	−3.94	−3.80	0.13
74	1025	0	−4.29	−3.80	0.49
80	1021	0	−4.08	−3.66	0.43
136	1010	2	−3.95	−3.74	0.21
47	1006	2	−4.07	−3.85	0.22
247	999	0	−4.22	−3.28	0.93
50	992	0	−4.28	−3.68	0.60

Continued on next page

Supplementary Table 15: Energetic and electronic properties for [Cu_8Zn_3] cores obtained with PBE/light-tier1 level and light SCF parameters: configuration number (i), relative total energy (ΔE_{tot}), total magnetic moment (m_{tot}), HOMO energy (ε_H), LUMO energy (ε_L), and LUMO-HOMO energy gap (E_g).

i	ΔE_{tot} (meV)	m_{tot} (μ_B)	ε_H (eV)	ε_L (eV)	E_g (eV)
12	988	2	−3.89	−3.72	0.17
43	983	2	−3.85	−3.75	0.10
258	980	0	−4.09	−3.34	0.75
27	979	2	−4.04	−3.93	0.11
284	975	0	−4.46	−3.26	1.19
235	972	0	−4.23	−3.87	0.37
5	970	2	−4.06	−3.84	0.22
8	969	2	−3.94	−3.77	0.16
210	961	0	−4.07	−3.53	0.54
19	960	2	−3.81	−3.66	0.14
69	955	2	−4.00	−3.74	0.26
183	954	0	−4.06	−3.30	0.76
153	952	0	−4.11	−3.64	0.47
206	948	2	−4.01	−3.76	0.25
238	945	0	−4.29	−3.98	0.31
52	943	2	−3.89	−3.80	0.09
276	942	0	−4.31	−3.44	0.87
102	930	0	−4.16	−3.72	0.44
224	919	2	−3.82	−3.65	0.17
117	917	0	−4.01	−3.38	0.63
78	914	0	−4.18	−3.64	0.54
285	911	0	−4.43	−3.25	1.18

Continued on next page

Supplementary Table 15: Energetic and electronic properties for [Cu_8Zn_3] cores obtained with PBE/light-tier1 level and light SCF parameters: configuration number (i), relative total energy (ΔE_{tot}), total magnetic moment (m_{tot}), HOMO energy (ε_H), LUMO energy (ε_L), and LUMO-HOMO energy gap (E_g).

i	ΔE_{tot} (meV)	m_{tot} (μ_B)	ε_H (eV)	ε_L (eV)	E_g (eV)
138	909	2	−3.85	−3.72	0.13
155	906	0	−4.27	−3.67	0.60
283	906	0	−4.54	−3.11	1.43
140	899	2	−3.79	−3.51	0.28
281	895	0	−4.58	−3.36	1.21
93	885	2	−3.90	−3.71	0.20
101	869	2	−4.09	−4.03	0.06
267	868	0	−4.19	−3.47	0.72
275	863	0	−4.31	−3.30	1.01
133	857	2	−3.98	−3.85	0.14
139	855	2	−3.81	−3.65	0.16
115	851	2	−4.01	−3.89	0.12
264	846	0	−4.31	−3.24	1.07
149	842	0	−4.28	−3.55	0.73
137	833	2	−3.79	−3.52	0.28
33	832	0	−4.14	−3.75	0.39
118	830	0	−4.21	−3.39	0.82
225	829	0	−4.36	−3.27	1.09
18	827	2	−3.86	−3.67	0.19
17	824	2	−4.01	−3.81	0.20
120	818	0	−4.24	−3.51	0.73
85	817	0	−4.05	−3.61	0.44

Continued on next page

Supplementary Table 15: Energetic and electronic properties for [Cu_8Zn_3] cores obtained with PBE/light-tier1 level and light SCF parameters: configuration number (i), relative total energy (ΔE_{tot}), total magnetic moment (m_{tot}), HOMO energy (ε_H), LUMO energy (ε_L), and LUMO-HOMO energy gap (E_g).

i	ΔE_{tot} (meV)	m_{tot} (μ_B)	ε_H (eV)	ε_L (eV)	E_g (eV)
13	813	2	−3.95	−3.74	0.22
88	811	2	−3.91	−3.80	0.11
29	810	2	−3.81	−3.64	0.17
96	792	2	−4.02	−3.78	0.24
112	792	2	−3.89	−3.72	0.16
114	788	2	−3.90	−3.88	0.03
30	781	2	−3.96	−3.93	0.03
3	779	2	−4.06	−3.91	0.15
214	764	2	−4.11	−4.03	0.08
277	760	0	−4.39	−3.34	1.06
90	756	0	−4.10	−3.57	0.54
240	751	0	−4.19	−3.41	0.78
232	744	0	−4.18	−3.69	0.49
36	744	0	−4.36	−3.79	0.57
37	741	0	−4.08	−3.39	0.68
198	739	0	−4.41	−3.44	0.97
26	739	0	−4.19	−3.60	0.58
48	737	0	−3.96	−3.56	0.40
172	733	0	−4.24	−3.10	1.13
233	732	0	−4.26	−3.75	0.51
46	727	0	−4.30	−3.56	0.75
125	724	0	−4.26	−3.29	0.96

Continued on next page

Supplementary Table 15: Energetic and electronic properties for [Cu_8Zn_3] cores obtained with PBE/light-tier1 level and light SCF parameters: configuration number (i), relative total energy (ΔE_{tot}), total magnetic moment (m_{tot}), HOMO energy (ε_H), LUMO energy (ε_L), and LUMO-HOMO energy gap (E_g).

i	ΔE_{tot} (meV)	m_{tot} (μ_B)	ε_H (eV)	ε_L (eV)	E_g (eV)
91	715	0	−4.07	−3.46	0.61
134	706	0	−4.13	−3.56	0.57
38	695	0	−4.23	−3.65	0.58
59	692	0	−4.16	−3.46	0.71
229	690	0	−4.36	−3.44	0.92
127	683	0	−4.49	−3.67	0.82
184	682	0	−4.25	−3.40	0.84
16	681	0	−4.27	−3.47	0.81
268	677	0	−4.46	−3.55	0.91
211	674	2	−3.91	−3.75	0.16
49	659	0	−4.27	−3.47	0.80
178	657	0	−4.06	−3.57	0.49
251	655	0	−4.12	−3.43	0.70
65	650	0	−4.32	−3.28	1.04
70	645	0	−4.24	−3.27	0.97
197	644	0	−4.08	−3.42	0.66
128	626	0	−4.15	−3.60	0.55
9	626	0	−3.93	−3.43	0.50
23	622	2	−3.87	−3.76	0.11
132	622	0	−4.40	−3.60	0.80
204	611	0	−4.05	−3.58	0.48
53	610	0	−4.29	−3.33	0.96

Continued on next page

Supplementary Table 15: Energetic and electronic properties for $[Cu_8Zn_3]$ cores obtained with PBE/light-tier1 level and light SCF parameters: configuration number (i), relative total energy (ΔE_{tot}), total magnetic moment (m_{tot}), HOMO energy (ε_H), LUMO energy (ε_L), and LUMO-HOMO energy gap (E_g).

i	ΔE_{tot} (meV)	m_{tot} (μ_B)	ε_H (eV)	ε_L (eV)	E_g (eV)
40	608	0	−4.23	−3.86	0.37
221	606	0	−4.36	−3.16	1.21
154	604	0	−4.03	−3.70	0.34
126	603	0	−4.30	−3.56	0.74
244	599	0	−4.18	−3.51	0.67
270	595	0	−4.30	−3.55	0.75
7	592	2	−3.98	−3.68	0.30
124	592	0	−4.08	−3.44	0.64
62	591	0	−4.25	−3.52	0.73
150	590	0	−4.10	−3.41	0.68
195	589	0	−4.19	−3.40	0.79
280	585	0	−4.41	−3.04	1.37
151	583	0	−4.04	−3.62	0.41
237	576	0	−4.21	−3.75	0.46
187	575	0	−4.12	−3.61	0.51
272	565	0	−4.26	−3.53	0.73
6	551	0	−4.19	−3.57	0.62
77	549	0	−4.32	−3.17	1.15
171	542	0	−4.29	−3.53	0.77
107	533	0	−4.27	−3.62	0.65
202	532	0	−4.22	−3.68	0.53
44	516	0	−4.32	−3.41	0.91

Continued on next page

Supplementary Table 15: Energetic and electronic properties for $[Cu_8Zn_3]$ cores obtained with PBE/light-tier1 level and light SCF parameters: configuration number (i), relative total energy (ΔE_{tot}), total magnetic moment (m_{tot}), HOMO energy (ε_H), LUMO energy (ε_L), and LUMO-HOMO energy gap (E_g).

i	ΔE_{tot} (meV)	m_{tot} (μ_B)	ε_H (eV)	ε_L (eV)	E_g (eV)
200	512	0	−4.22	−3.44	0.78
63	511	0	−4.26	−3.25	1.01
10	500	0	−4.28	−3.48	0.80
218	499	0	−4.14	−3.49	0.66
226	498	0	−4.49	−3.43	1.06
287	497	0	−4.12	−3.13	0.99
188	491	0	−4.18	−3.50	0.67
236	486	0	−4.34	−3.84	0.49
72	485	0	−4.17	−3.36	0.80
97	484	0	−4.21	−3.45	0.76
4	480	0	−4.26	−3.51	0.75
167	479	0	−4.21	−3.64	0.57
100	478	0	−4.22	−3.74	0.48
103	477	0	−4.32	−3.66	0.67
203	476	0	−4.38	−3.36	1.02
148	473	0	−4.33	−3.60	0.72
168	466	0	−4.23	−3.67	0.56
104	466	0	−4.35	−3.64	0.71
234	460	0	−4.32	−3.61	0.71
207	456	0	−4.23	−3.57	0.66
192	456	0	−4.17	−3.56	0.61
152	452	0	−4.22	−3.54	0.68

Continued on next page

Supplementary Table 15: Energetic and electronic properties for $[Cu_8Zn_3]$ cores obtained with PBE/light-tier1 level and light SCF parameters: configuration number (i), relative total energy (ΔE_{tot}), total magnetic moment (m_{tot}), HOMO energy (ε_H), LUMO energy (ε_L), and LUMO-HOMO energy gap (E_g).

i	ΔE_{tot} (meV)	m_{tot} (μ_B)	ε_H (eV)	ε_L (eV)	E_g (eV)
289	451	0	−4.38	−3.39	0.98
61	448	0	−4.24	−3.23	1.01
113	443	0	−4.31	−3.54	0.78
45	441	0	−4.42	−3.32	1.11
256	440	0	−4.32	−3.16	1.16
273	438	0	−4.42	−3.66	0.75
11	436	0	−4.39	−3.69	0.70
94	426	0	−4.17	−3.49	0.68
129	417	0	−4.08	−3.27	0.81
201	404	0	−4.27	−3.38	0.89
245	391	0	−4.29	−3.18	1.10
174	391	0	−4.18	−3.39	0.80
14	382	0	−4.33	−3.51	0.82
111	379	0	−4.27	−3.56	0.71
173	373	0	−4.35	−3.58	0.76
176	362	0	−4.30	−3.62	0.68
166	359	0	−4.25	−3.52	0.72
191	358	0	−4.27	−3.37	0.90
98	355	0	−4.20	−3.46	0.74
141	354	0	−4.21	−3.47	0.74
60	351	0	−4.26	−3.27	0.99
20	345	0	−4.21	−3.42	0.79

Continued on next page

Supplementary Table 15: Energetic and electronic properties for [Cu_8Zn_3] cores obtained with PBE/light-tier1 level and light SCF parameters: configuration number (i), relative total energy (ΔE_{tot}), total magnetic moment (m_{tot}), HOMO energy (ε_H), LUMO energy (ε_L), and LUMO-HOMO energy gap (E_g).

i	ΔE_{tot} (meV)	m_{tot} (μ_B)	ε_H (eV)	ε_L (eV)	E_g (eV)
219	342	0	−4.28	−3.62	0.66
269	338	0	−4.28	−3.40	0.88
73	336	0	−4.30	−3.22	1.08
54	327	0	−4.24	−3.43	0.81
194	320	0	−4.21	−3.46	0.76
31	316	0	−4.31	−3.28	1.02
142	307	0	−4.37	−3.22	1.16
163	301	0	−4.28	−3.40	0.88
177	299	0	−4.39	−3.13	1.26
175	290	0	−4.24	−3.31	0.92
249	287	0	−4.46	−3.29	1.16
217	276	0	−4.16	−3.42	0.74
288	275	0	−4.44	−3.32	1.12
170	273	0	−4.29	−3.38	0.91
58	256	0	−4.27	−3.51	0.76
123	253	0	−4.33	−3.22	1.11
169	250	0	−4.21	−3.57	0.64
51	243	0	−4.20	−3.25	0.95
55	240	0	−4.26	−3.32	0.95
223	226	0	−4.39	−3.60	0.80
1	220	0	−4.31	−3.62	0.70
266	218	0	−4.45	−3.43	1.03

Continued on next page

Supplementary Table 15: Energetic and electronic properties for [Cu_8Zn_3] cores obtained with PBE/light-tier1 level and light SCF parameters: configuration number (i), relative total energy (ΔE_{tot}), total magnetic moment (m_{tot}), HOMO energy (ε_H), LUMO energy (ε_L), and LUMO-HOMO energy gap (E_g).

i	ΔE_{tot} (meV)	m_{tot} (μ_B)	ε_H (eV)	ε_L (eV)	E_g (eV)
164	217	0	−4.17	−3.42	0.75
193	209	0	−4.40	−3.23	1.17
228	194	0	−4.30	−3.31	0.99
190	180	0	−4.42	−3.30	1.12
196	175	0	−4.41	−3.31	1.11
189	168	0	−4.15	−3.31	0.83
71	150	0	−4.44	−3.11	1.33
246	138	0	−4.44	−3.42	1.03
208	135	0	−4.44	−3.14	1.30
231	129	0	−4.31	−3.31	0.99
157	117	0	−4.21	−3.50	0.70
24	112	0	−4.07	−3.36	0.71
64	107	0	−4.28	−3.20	1.07
186	105	0	−4.30	−3.22	1.07
254	98	0	−4.41	−3.32	1.09
32	67	0	−4.38	−3.47	0.91
250	18	0	−4.44	−3.24	1.19
105	0	0	−4.38	−3.21	1.17

2.6.6 [Cu_4Zn_{10}]

Supplementary Table 16: Energetic and electronic properties for $[Cu_4Zn_{10}]$ cores obtained with PBE/light-tier1 level and light SCF parameters: configuration number (i), relative total energy (ΔE_{tot}), total magnetic moment (m_{tot}), HOMO energy (ε_H), LUMO energy (ε_L), and LUMO-HOMO energy gap (E_g).

i	ΔE_{tot} (meV)	m_{tot} (μ_B)	ε_H (eV)	ε_L (eV)	E_g (eV)
227	2334	2	−3.97	−3.80	0.17
168	1946	2	−4.05	−3.87	0.18
228	1896	2	−3.91	−3.75	0.16
26	1895	2	−3.84	−3.73	0.11
100	1794	2	−3.97	−3.89	0.09
152	1785	0	−4.32	−3.33	0.99
29	1675	2	−3.98	−3.75	0.23
154	1651	0	−4.20	−3.39	0.81
135	1599	2	−3.97	−3.83	0.14
61	1573	2	−3.77	−3.49	0.28
156	1550	0	−4.46	−3.14	1.32
163	1543	4	−4.03	−3.77	0.26
2	1531	2	−3.79	−3.60	0.19
4	1512	2	−4.04	−3.82	0.22
40	1486	2	−3.87	−3.79	0.09
120	1470	0	−4.06	−3.57	0.49
165	1451	2	−4.00	−3.72	0.28
198	1411	0	−4.27	−3.17	1.10
57	1401	0	−4.28	−3.39	0.89
208	1382	2	−3.82	−3.74	0.08
79	1376	2	−3.91	−3.80	0.12
112	1347	2	−4.00	−3.81	0.18

Continued on next page

Supplementary Table 16: Energetic and electronic properties for $[Cu_4Zn_{10}]$ cores obtained with PBE/light-tier1 level and light SCF parameters: configuration number (i), relative total energy (ΔE_{tot}), total magnetic moment (m_{tot}), HOMO energy (ε_H), LUMO energy (ε_L), and LUMO-HOMO energy gap (E_g).

i	ΔE_{tot} (meV)	m_{tot} (μ_B)	ε_H (eV)	ε_L (eV)	E_g (eV)
162	1346	2	−3.86	−3.71	0.15
232	1343	2	−3.89	−3.79	0.10
81	1342	0	−4.09	−3.60	0.49
187	1331	0	−4.16	−3.15	1.01
99	1330	2	−3.97	−3.83	0.14
133	1330	2	−3.93	−3.79	0.14
222	1328	2	−3.92	−3.76	0.16
226	1324	0	−4.20	−3.35	0.86
8	1322	2	−3.87	−3.74	0.14
10	1311	2	−3.73	−3.63	0.11
189	1294	2	−3.90	−3.71	0.19
212	1289	2	−3.93	−3.71	0.22
213	1288	2	−4.02	−3.82	0.20
95	1283	0	−4.26	−3.59	0.67
176	1275	2	−3.92	−3.69	0.23
25	1271	0	−4.20	−3.51	0.69
202	1262	0	−4.32	−3.30	1.02
209	1256	2	−4.02	−3.77	0.25
234	1241	2	−3.86	−3.58	0.28
32	1234	2	−3.91	−3.81	0.10
206	1234	0	−4.03	−3.60	0.44
191	1234	2	−4.10	−3.89	0.21

Continued on next page

Supplementary Table 16: Energetic and electronic properties for $[Cu_4Zn_{10}]$ cores obtained with PBE/light-tier1 level and light SCF parameters: configuration number (i), relative total energy (ΔE_{tot}), total magnetic moment (m_{tot}), HOMO energy (ε_H), LUMO energy (ε_L), and LUMO-HOMO energy gap (E_g).

i	ΔE_{tot} (meV)	m_{tot} (μ_B)	ε_H (eV)	ε_L (eV)	E_g (eV)
137	1233	2	−3.96	−3.72	0.25
94	1232	0	−4.28	−3.33	0.95
20	1229	2	−4.08	−3.81	0.27
53	1204	2	−3.97	−3.83	0.15
203	1187	2	−4.02	−3.81	0.21
171	1176	0	−4.04	−3.45	0.59
238	1175	0	−4.09	−3.46	0.63
1	1166	0	−4.42	−3.32	1.10
136	1157	2	−3.97	−3.85	0.12
180	1146	2	−3.90	−3.68	0.22
9	1145	0	−4.21	−3.54	0.66
128	1130	0	−4.26	−3.44	0.82
50	1127	0	−4.25	−3.24	1.01
45	1117	0	−4.32	−3.45	0.87
38	1097	0	−4.14	−3.47	0.67
106	1091	0	−4.21	−3.51	0.71
178	1076	2	−3.90	−3.74	0.17
204	1072	2	−3.90	−3.71	0.19
216	1069	0	−4.26	−3.45	0.80
12	1067	0	−4.19	−3.78	0.41
72	1055	0	−4.28	−3.45	0.83
219	1054	0	−4.42	−3.09	1.33

Continued on next page

Supplementary Table 16: Energetic and electronic properties for $[Cu_4Zn_{10}]$ cores obtained with PBE/light-tier1 level and light SCF parameters: configuration number (i), relative total energy (ΔE_{tot}), total magnetic moment (m_{tot}), HOMO energy (ε_H), LUMO energy (ε_L), and LUMO-HOMO energy gap (E_g).

i	ΔE_{tot} (meV)	m_{tot} (μ_B)	ε_H (eV)	ε_L (eV)	E_g (eV)
5	1040	2	−3.85	−3.61	0.24
215	1040	2	−4.04	−3.81	0.22
190	1035	0	−4.18	−3.58	0.59
205	1034	0	−4.14	−3.55	0.59
153	1032	0	−4.40	−2.99	1.41
197	1029	0	−4.12	−3.21	0.91
13	1023	2	−3.96	−3.81	0.14
115	1013	0	−4.12	−3.52	0.60
27	1008	0	−4.15	−3.53	0.62
105	997	0	−4.21	−3.36	0.85
122	982	0	−4.09	−3.34	0.76
169	977	0	−4.35	−3.47	0.88
183	976	0	−4.33	−3.30	1.02
161	967	0	−4.10	−3.51	0.59
65	962	0	−4.13	−3.35	0.78
233	957	2	−3.78	−3.70	0.08
126	936	0	−4.38	−3.34	1.05
200	935	2	−4.01	−3.78	0.24
103	932	0	−4.09	−3.35	0.74
80	929	0	−4.23	−3.50	0.73
196	927	0	−4.16	−3.07	1.09
89	924	0	−4.19	−3.25	0.94

Continued on next page

Supplementary Table 16: Energetic and electronic properties for [Cu_4Zn_{10}] cores obtained with PBE/light-tier1 level and light SCF parameters: configuration number (i), relative total energy (ΔE_{tot}), total magnetic moment (m_{tot}), HOMO energy (ε_H), LUMO energy (ε_L), and LUMO-HOMO energy gap (E_g).

i	ΔE_{tot} (meV)	m_{tot} (μ_B)	ε_H (eV)	ε_L (eV)	E_g (eV)
177	909	2	−3.74	−3.68	0.06
113	906	0	−4.30	−3.26	1.04
218	904	0	−4.20	−3.31	0.90
157	904	0	−4.34	−2.99	1.34
132	901	0	−4.51	−3.25	1.26
207	900	0	−4.06	−3.26	0.80
195	897	0	−4.28	−3.40	0.88
199	885	0	−4.00	−2.94	1.06
225	884	0	−4.31	−3.28	1.04
194	869	0	−4.12	−3.52	0.60
143	859	0	−4.36	−3.23	1.13
21	850	0	−4.37	−3.08	1.28
164	849	0	−4.30	−3.47	0.83
114	842	0	−4.23	−3.46	0.77
201	833	0	−4.30	−3.22	1.07
109	832	0	−4.14	−3.37	0.77
230	821	0	−4.32	−3.47	0.85
86	821	0	−4.05	−3.44	0.60
217	815	0	−4.28	−3.50	0.78
192	814	0	−4.29	−3.53	0.76
76	813	0	−4.17	−3.52	0.65
71	807	0	−4.15	−3.33	0.82

Continued on next page

Supplementary Table 16: Energetic and electronic properties for [Cu_4Zn_{10}] cores obtained with PBE/light-tier1 level and light SCF parameters: configuration number (i), relative total energy (ΔE_{tot}), total magnetic moment (m_{tot}), HOMO energy (ε_H), LUMO energy (ε_L), and LUMO-HOMO energy gap (E_g).

i	ΔE_{tot} (meV)	m_{tot} (μ_B)	ε_H (eV)	ε_L (eV)	E_g (eV)
231	804	0	−4.10	−3.42	0.67
93	803	0	−4.24	−3.10	1.14
134	802	0	−4.15	−3.48	0.67
139	801	0	−4.18	−3.19	0.99
129	800	0	−4.12	−3.16	0.95
130	790	0	−4.28	−3.24	1.04
19	779	0	−4.30	−3.30	1.00
229	778	0	−4.41	−3.27	1.14
97	764	0	−4.23	−3.46	0.77
91	758	0	−4.15	−3.29	0.86
142	744	0	−4.36	−3.21	1.16
84	737	0	−4.22	−3.26	0.96
11	736	0	−4.31	−3.40	0.91
104	728	0	−4.18	−3.20	0.98
193	727	0	−4.07	−3.33	0.74
188	717	0	−4.28	−3.31	0.97
23	717	0	−4.19	−3.19	1.00
98	715	0	−4.01	−3.18	0.84
14	710	0	−4.29	−3.64	0.65
73	688	0	−4.29	−3.35	0.94
16	687	0	−4.21	−3.42	0.79
31	684	0	−4.40	−3.19	1.21

Continued on next page

Supplementary Table 16: Energetic and electronic properties for $[Cu_4Zn_{10}]$ cores obtained with PBE/light-tier1 level and light SCF parameters: configuration number (i), relative total energy (ΔE_{tot}), total magnetic moment (m_{tot}), HOMO energy (ε_H), LUMO energy (ε_L), and LUMO-HOMO energy gap (E_g).

i	ΔE_{tot} (meV)	m_{tot} (μ_B)	ε_H (eV)	ε_L (eV)	E_g (eV)
159	682	0	−4.21	−3.37	0.84
125	680	0	−4.14	−3.27	0.88
167	676	0	−4.25	−3.58	0.67
185	670	0	−4.40	−3.01	1.39
140	662	0	−4.32	−3.22	1.10
237	662	0	−4.48	−2.93	1.55
108	660	0	−3.96	−3.36	0.60
42	660	0	−4.29	−3.13	1.15
240	657	0	−4.31	−3.03	1.27
15	653	0	−4.25	−3.34	0.91
110	645	0	−4.48	−3.12	1.36
116	645	0	−4.22	−3.12	1.10
181	644	0	−4.21	−3.26	0.95
155	639	0	−4.42	−3.08	1.35
166	628	0	−4.30	−3.25	1.05
214	627	0	−4.27	−3.16	1.11
22	622	0	−4.18	−3.33	0.85
107	602	0	−4.32	−3.32	1.00
90	602	0	−4.34	−3.19	1.15
96	599	0	−4.30	−3.23	1.07
37	597	0	−4.46	−3.20	1.25
28	587	0	−4.41	−3.13	1.28

Continued on next page

Supplementary Table 16: Energetic and electronic properties for $[Cu_4Zn_{10}]$ cores obtained with PBE/light-tier1 level and light SCF parameters: configuration number (i), relative total energy (ΔE_{tot}), total magnetic moment (m_{tot}), HOMO energy (ε_H), LUMO energy (ε_L), and LUMO-HOMO energy gap (E_g).

i	ΔE_{tot} (meV)	m_{tot} (μ_B)	ε_H (eV)	ε_L (eV)	E_g (eV)
63	580	0	−4.28	−3.28	1.00
117	580	0	−4.12	−3.09	1.03
127	568	0	−4.11	−3.42	0.69
88	567	0	−4.32	−3.27	1.04
46	564	0	−4.28	−3.29	1.00
144	564	0	−4.16	−3.27	0.89
70	558	0	−4.02	−3.06	0.96
3	553	0	−4.14	−3.06	1.08
75	551	0	−4.15	−3.30	0.85
175	547	0	−4.33	−3.27	1.06
221	538	0	−4.22	−3.12	1.10
151	537	0	−4.37	−2.95	1.42
59	531	0	−4.31	−3.22	1.09
83	526	0	−4.10	−3.24	0.86
87	525	0	−4.35	−3.04	1.31
49	518	0	−4.32	−3.12	1.21
220	517	0	−4.16	−3.18	0.98
101	509	0	−4.44	−3.06	1.38
239	508	0	−4.33	−3.03	1.30
85	507	0	−4.41	−3.17	1.24
119	499	0	−4.28	−2.96	1.32
124	497	0	−4.18	−3.19	0.99

Continued on next page

Supplementary Table 16: Energetic and electronic properties for $[Cu_4Zn_{10}]$ cores obtained with PBE/light-tier1 level and light SCF parameters: configuration number (i), relative total energy (ΔE_{tot}), total magnetic moment (m_{tot}), HOMO energy (ε_H), LUMO energy (ε_L), and LUMO-HOMO energy gap (E_g).

i	ΔE_{tot} (meV)	m_{tot} (μ_B)	ε_H (eV)	ε_L (eV)	E_g (eV)
147	486	0	−4.27	−3.13	1.14
145	474	0	−4.23	−3.31	0.92
179	461	0	−4.26	−3.33	0.93
141	460	0	−4.23	−2.88	1.35
211	459	0	−4.27	−3.16	1.11
224	456	0	−4.37	−3.03	1.33
123	455	0	−4.02	−3.00	1.02
77	453	0	−4.28	−3.50	0.79
39	448	0	−4.28	−2.96	1.32
92	444	0	−4.23	−3.14	1.09
55	443	0	−4.42	−3.21	1.20
64	434	0	−4.29	−2.97	1.32
67	432	0	−4.25	−3.13	1.12
18	423	0	−4.44	−3.44	1.00
34	418	0	−4.28	−3.02	1.26
235	405	0	−3.99	−3.14	0.85
102	390	0	−4.19	−2.99	1.19
74	385	0	−4.36	−3.39	0.98
69	380	0	−4.11	−3.38	0.73
184	377	0	−4.23	−3.27	0.96
44	368	0	−4.42	−3.21	1.21
186	365	0	−4.48	−3.30	1.19

Continued on next page

Supplementary Table 16: Energetic and electronic properties for $[Cu_4Zn_{10}]$ cores obtained with PBE/light-tier1 level and light SCF parameters: configuration number (i), relative total energy (ΔE_{tot}), total magnetic moment (m_{tot}), HOMO energy (ε_H), LUMO energy (ε_L), and LUMO-HOMO energy gap (E_g).

i	ΔE_{tot} (meV)	m_{tot} (μ_B)	ε_H (eV)	ε_L (eV)	E_g (eV)
47	359	0	−4.27	−3.25	1.02
118	356	0	−4.17	−2.94	1.24
24	355	0	−4.35	−3.14	1.21
30	345	0	−4.30	−3.25	1.05
210	332	0	−4.50	−3.24	1.26
54	321	0	−4.34	−3.34	1.00
66	311	0	−4.37	−3.04	1.33
150	306	0	−4.28	−3.13	1.16
160	299	0	−4.46	−3.30	1.16
78	299	0	−4.49	−3.13	1.35
149	293	0	−4.38	−3.14	1.23
52	288	0	−4.22	−3.13	1.08
58	283	0	−4.32	−3.05	1.27
43	271	0	−4.09	−3.31	0.78
223	248	0	−4.43	−3.28	1.15
60	246	0	−4.37	−3.20	1.17
138	219	0	−4.25	−3.12	1.13
17	213	0	−4.27	−3.05	1.22
35	205	0	−4.24	−3.08	1.16
41	197	0	−4.43	−3.12	1.32
148	193	0	−4.05	−3.08	0.97
172	183	0	−4.36	−3.19	1.16

Continued on next page

Supplementary Table 16: Energetic and electronic properties for [Cu_4Zn_{10}] cores obtained with PBE/light-tier1 level and light SCF parameters: configuration number (i), relative total energy (ΔE_{tot}), total magnetic moment (m_{tot}), HOMO energy (ε_H), LUMO energy (ε_L), and LUMO-HOMO energy gap (E_g).

i	ΔE_{tot} (meV)	m_{tot} (μ_B)	ε_H (eV)	ε_L (eV)	E_g (eV)
236	174	0	−4.29	−3.05	1.24
62	174	0	−4.49	−3.27	1.22
111	163	0	−4.26	−2.94	1.32
33	145	0	−4.42	−3.24	1.18
56	99	0	−4.39	−3.12	1.27
146	97	0	−4.18	−3.09	1.09
182	77	0	−4.23	−3.21	1.03
7	61	0	−4.21	−3.05	1.16
173	42	0	−4.49	−2.94	1.55
68	39	0	−4.49	−3.05	1.43
131	8	0	−4.26	−3.04	1.22
6	0	0	−4.20	−3.20	0.99

2.6.7 [$Cu_{11}Zn$]

Supplementary Table 17: Energetic and electronic properties for [$Cu_{11}Zn_6$] cores obtained with PBE/light-tier1 level and light SCF parameters: configuration number (i), relative total energy (ΔE_{tot}), total magnetic moment (m_{tot}), HOMO energy (ε_H), LUMO energy (ε_L), and LUMO-HOMO energy gap (E_g).

i	ΔE_{tot} (meV)	m_{tot} (μ_B)	ε_H (eV)	ε_L (eV)	E_g (eV)
37	4780	1	−3.75	−3.56	0.20
35	4093	1	−3.87	−3.61	0.26

Continued on next page

Supplementary Table 17: Energetic and electronic properties for [$Cu_{11}Zn_6$] cores obtained with PBE/light-tier1 level and light SCF parameters: configuration number (i), relative total energy (ΔE_{tot}), total magnetic moment (m_{tot}), HOMO energy (ε_H), LUMO energy (ε_L), and LUMO-HOMO energy gap (E_g).

i	ΔE_{tot} (meV)	m_{tot} (μ_B)	ε_H (eV)	ε_L (eV)	E_g (eV)
74	3982	1	−3.80	−3.61	0.19
94	3581	1	−3.98	−3.75	0.23
41	3505	1	−3.81	−3.62	0.18
48	3457	1	−3.91	−3.73	0.17
52	3102	1	−4.04	−3.87	0.17
97	3081	1	−3.90	−3.73	0.16
40	2892	1	−3.88	−3.70	0.18
175	2874	1	−3.79	−3.60	0.19
73	2841	1	−4.03	−3.86	0.17
75	2772	1	−4.01	−3.81	0.20
95	2674	1	−3.71	−3.51	0.19
179	2585	1	−3.89	−3.69	0.19
68	2507	1	−4.19	−4.00	0.19
38	2503	1	−3.92	−3.73	0.18
191	2461	1	−3.74	−3.54	0.21
155	2438	1	−3.94	−3.71	0.23
100	2436	1	−3.87	−3.65	0.21
135	2366	1	−4.23	−4.06	0.18
178	2336	1	−3.84	−3.66	0.18
43	2324	1	−3.85	−3.65	0.20
129	2309	1	−3.93	−3.73	0.20
23	2295	1	−3.84	−3.63	0.21

Continued on next page

Supplementary Table 17: Energetic and electronic properties for $[Cu_{11}Zn_6]$ cores obtained with PBE/light-tier1 level and light SCF parameters: configuration number (i), relative total energy (ΔE_{tot}), total magnetic moment (m_{tot}), HOMO energy (ε_H), LUMO energy (ε_L), and LUMO-HOMO energy gap (E_g).

i	ΔE_{tot} (meV)	m_{tot} (μ_B)	ε_H (eV)	ε_L (eV)	E_g (eV)
72	2228	1	−4.14	−3.97	0.17
120	2154	1	−4.00	−3.82	0.17
50	2150	1	−3.66	−3.41	0.25
205	2134	1	−3.81	−3.62	0.19
103	2106	1	−3.83	−3.63	0.20
219	2097	1	−3.83	−3.60	0.23
149	2095	1	−3.79	−3.61	0.18
12	2088	1	−3.93	−3.77	0.16
105	2062	1	−3.78	−3.56	0.22
67	2032	1	−3.94	−3.74	0.20
220	2031	1	−3.94	−3.73	0.21
46	2027	1	−4.01	−3.72	0.28
188	1991	1	−3.98	−3.80	0.18
186	1989	1	−3.90	−3.71	0.19
134	1964	1	−4.07	−3.82	0.25
127	1962	1	−3.93	−3.73	0.20
221	1957	1	−3.80	−3.55	0.25
133	1916	1	−3.96	−3.72	0.23
206	1914	1	−3.99	−3.81	0.19
64	1907	1	−3.94	−3.75	0.19
165	1834	1	−4.20	−3.98	0.22
130	1826	1	−3.91	−3.73	0.18

Continued on next page

Supplementary Table 17: Energetic and electronic properties for $[Cu_{11}Zn_6]$ cores obtained with PBE/light-tier1 level and light SCF parameters: configuration number (i), relative total energy (ΔE_{tot}), total magnetic moment (m_{tot}), HOMO energy (ε_H), LUMO energy (ε_L), and LUMO-HOMO energy gap (E_g).

i	ΔE_{tot} (meV)	m_{tot} (μ_B)	ε_H (eV)	ε_L (eV)	E_g (eV)
65	1823	1	−4.29	−4.11	0.17
146	1819	1	−4.00	−3.80	0.20
204	1815	1	−3.95	−3.77	0.18
5	1793	1	−3.86	−3.68	0.18
214	1779	1	−3.85	−3.64	0.21
124	1778	1	−3.99	−3.81	0.18
292	1775	1	−3.78	−3.54	0.24
90	1757	1	−3.96	−3.72	0.24
89	1747	1	−3.67	−3.51	0.16
196	1746	1	−4.05	−3.84	0.21
166	1720	1	−4.07	−3.90	0.17
159	1713	1	−3.71	−3.45	0.26
154	1708	1	−3.84	−3.68	0.16
101	1698	1	−4.16	−4.00	0.16
131	1691	1	−4.01	−3.76	0.25
79	1687	1	−4.11	−3.95	0.16
121	1674	1	−3.89	−3.70	0.18
11	1663	1	−4.04	−3.85	0.19
92	1646	1	−4.06	−3.90	0.16
29	1582	1	−3.91	−3.74	0.16
177	1577	1	−4.02	−3.85	0.17
115	1549	1	−4.26	−4.09	0.17

Continued on next page

Supplementary Table 17: Energetic and electronic properties for $[Cu_{11}Zn_6]$ cores obtained with PBE/light-tier1 level and light SCF parameters: configuration number (i), relative total energy (ΔE_{tot}), total magnetic moment (m_{tot}), HOMO energy (ε_H), LUMO energy (ε_L), and LUMO-HOMO energy gap (E_g).

i	ΔE_{tot} (meV)	m_{tot} (μ_B)	ε_H (eV)	ε_L (eV)	E_g (eV)
30	1541	1	−3.87	−3.71	0.16
69	1508	1	−3.64	−3.42	0.22
83	1502	1	−4.03	−3.87	0.17
108	1492	1	−4.02	−3.72	0.29
20	1482	1	−3.92	−3.73	0.19
4	1480	1	−4.08	−3.89	0.19
243	1480	1	−3.95	−3.75	0.20
167	1466	1	−3.78	−3.56	0.22
118	1461	1	−3.86	−3.67	0.19
71	1456	1	−3.69	−3.49	0.20
297	1451	1	−3.81	−3.55	0.26
7	1427	1	−4.00	−3.82	0.18
24	1411	1	−4.00	−3.83	0.16
213	1400	1	−3.76	−3.56	0.20
151	1398	1	−3.89	−3.71	0.18
99	1396	1	−4.26	−4.10	0.16
14	1392	1	−3.59	−3.37	0.22
91	1391	1	−4.18	−3.99	0.19
217	1390	1	−3.91	−3.72	0.19
194	1389	1	−3.85	−3.64	0.21
22	1386	1	−3.96	−3.78	0.19
126	1383	1	−3.97	−3.79	0.18

Continued on next page

Supplementary Table 17: Energetic and electronic properties for $[Cu_{11}Zn_6]$ cores obtained with PBE/light-tier1 level and light SCF parameters: configuration number (i), relative total energy (ΔE_{tot}), total magnetic moment (m_{tot}), HOMO energy (ε_H), LUMO energy (ε_L), and LUMO-HOMO energy gap (E_g).

i	ΔE_{tot} (meV)	m_{tot} (μ_B)	ε_H (eV)	ε_L (eV)	E_g (eV)
150	1380	1	−4.06	−3.85	0.21
9	1366	1	−3.69	−3.49	0.21
270	1364	1	−3.69	−3.47	0.22
202	1363	1	−3.95	−3.77	0.19
54	1360	1	−3.59	−3.37	0.22
10	1360	1	−3.94	−3.76	0.18
157	1358	1	−3.94	−3.75	0.20
210	1334	1	−4.06	−3.88	0.17
85	1334	1	−3.83	−3.66	0.17
51	1330	1	−3.97	−3.75	0.22
265	1314	1	−3.78	−3.55	0.23
266	1289	1	−3.85	−3.56	0.29
109	1272	1	−3.89	−3.71	0.18
25	1269	1	−4.19	−4.01	0.18
156	1265	1	−3.89	−3.70	0.20
152	1254	1	−4.05	−3.84	0.21
296	1244	1	−3.77	−3.57	0.20
244	1237	1	−3.97	−3.81	0.16
56	1219	1	−4.06	−3.87	0.19
269	1216	1	−4.02	−3.76	0.26
96	1209	1	−3.68	−3.47	0.21
111	1207	1	−3.95	−3.75	0.20

Continued on next page

Supplementary Table 17: Energetic and electronic properties for $[Cu_{11}Zn_6]$ cores obtained with PBE/light-tier1 level and light SCF parameters: configuration number (i), relative total energy (ΔE_{tot}), total magnetic moment (m_{tot}), HOMO energy (ε_H), LUMO energy (ε_L), and LUMO-HOMO energy gap (E_g).

i	ΔE_{tot} (meV)	m_{tot} (μ_B)	ε_H (eV)	ε_L (eV)	E_g (eV)
174	1204	1	−3.83	−3.61	0.22
281	1197	1	−3.80	−3.53	0.27
211	1197	1	−3.85	−3.64	0.20
132	1193	1	−3.85	−3.65	0.20
242	1176	1	−4.00	−3.80	0.21
114	1175	1	−3.81	−3.55	0.26
117	1170	1	−3.91	−3.73	0.17
279	1162	1	−3.89	−3.68	0.20
62	1153	1	−3.88	−3.68	0.20
45	1149	1	−3.64	−3.46	0.18
102	1148	1	−3.99	−3.79	0.20
288	1147	1	−3.77	−3.55	0.22
153	1147	1	−3.85	−3.61	0.24
18	1136	1	−4.01	−3.85	0.17
19	1133	1	−3.87	−3.71	0.17
268	1131	1	−3.64	−3.36	0.27
27	1126	1	−4.05	−3.85	0.20
216	1121	1	−3.92	−3.70	0.21
255	1111	1	−3.79	−3.57	0.21
143	1109	1	−4.16	−3.96	0.20
66	1105	1	−3.91	−3.73	0.18
247	1096	1	−4.08	−3.85	0.23

Continued on next page

Supplementary Table 17: Energetic and electronic properties for $[Cu_{11}Zn_6]$ cores obtained with PBE/light-tier1 level and light SCF parameters: configuration number (i), relative total energy (ΔE_{tot}), total magnetic moment (m_{tot}), HOMO energy (ε_H), LUMO energy (ε_L), and LUMO-HOMO energy gap (E_g).

i	ΔE_{tot} (meV)	m_{tot} (μ_B)	ε_H (eV)	ε_L (eV)	E_g (eV)
33	1095	1	−3.77	−3.55	0.21
190	1076	1	−4.12	−3.93	0.19
170	1072	1	−3.85	−3.62	0.23
274	1062	1	−3.81	−3.59	0.23
58	1059	1	−3.73	−3.54	0.19
32	1054	1	−3.78	−3.60	0.18
235	1054	1	−3.76	−3.49	0.27
53	1050	1	−3.96	−3.76	0.21
87	1048	1	−3.82	−3.61	0.21
261	1044	1	−3.75	−3.53	0.22
6	1036	1	−4.03	−3.85	0.18
187	1030	1	−4.01	−3.79	0.22
110	1026	1	−3.79	−3.57	0.22
180	1020	1	−3.89	−3.63	0.26
238	1019	1	−3.77	−3.58	0.19
280	1017	1	−3.90	−3.57	0.33
106	1014	1	−4.06	−3.89	0.17
236	1000	1	−3.85	−3.63	0.22
218	997	1	−3.59	−3.40	0.19
264	970	1	−3.85	−3.68	0.17
107	969	1	−4.14	−3.96	0.18
55	969	1	−3.97	−3.79	0.18

Continued on next page

Supplementary Table 17: Energetic and electronic properties for $[Cu_{11}Zn_6]$ cores obtained with PBE/light-tier1 level and light SCF parameters: configuration number (i), relative total energy (ΔE_{tot}), total magnetic moment (m_{tot}), HOMO energy (ε_H), LUMO energy (ε_L), and LUMO-HOMO energy gap (E_g).

i	ΔE_{tot} (meV)	m_{tot} (μ_B)	ε_H (eV)	ε_L (eV)	E_g (eV)
234	965	1	−3.96	−3.74	0.22
240	960	1	−3.82	−3.62	0.20
142	959	1	−3.90	−3.69	0.21
207	957	1	−3.97	−3.74	0.23
226	937	1	−3.78	−3.53	0.25
227	935	1	−3.91	−3.67	0.24
57	929	1	−3.71	−3.52	0.19
42	924	1	−4.08	−3.91	0.18
200	917	1	−3.89	−3.65	0.23
209	908	1	−3.68	−3.45	0.23
136	904	1	−3.85	−3.65	0.19
300	895	1	−4.03	−3.84	0.19
171	886	1	−3.88	−3.68	0.20
294	885	1	−3.63	−3.42	0.21
176	876	1	−3.76	−3.55	0.21
273	873	1	−4.08	−3.88	0.20
82	872	1	−3.90	−3.72	0.18
17	870	1	−3.72	−3.52	0.20
272	870	1	−3.79	−3.61	0.18
16	866	1	−3.89	−3.70	0.19
163	864	1	−3.91	−3.66	0.25
93	847	1	−3.86	−3.61	0.25

Continued on next page

Supplementary Table 17: Energetic and electronic properties for $[Cu_{11}Zn_6]$ cores obtained with PBE/light-tier1 level and light SCF parameters: configuration number (i), relative total energy (ΔE_{tot}), total magnetic moment (m_{tot}), HOMO energy (ε_H), LUMO energy (ε_L), and LUMO-HOMO energy gap (E_g).

i	ΔE_{tot} (meV)	m_{tot} (μ_B)	ε_H (eV)	ε_L (eV)	E_g (eV)
250	840	1	−4.11	−3.93	0.18
295	812	1	−3.89	−3.67	0.22
299	804	1	−3.66	−3.46	0.20
123	801	1	−3.98	−3.82	0.15
237	798	1	−4.09	−3.86	0.23
49	796	1	−4.07	−3.88	0.19
145	795	1	−3.99	−3.77	0.22
63	792	1	−3.73	−3.51	0.22
241	792	1	−3.78	−3.52	0.26
259	783	1	−3.76	−3.51	0.25
193	776	1	−3.92	−3.75	0.16
86	776	1	−4.19	−3.99	0.21
128	770	1	−3.93	−3.73	0.20
76	765	1	−3.97	−3.72	0.25
34	761	1	−3.95	−3.78	0.17
139	757	1	−4.29	−4.11	0.18
197	753	1	−4.01	−3.80	0.21
228	745	1	−3.72	−3.51	0.22
203	744	1	−3.74	−3.57	0.17
168	734	1	−3.82	−3.65	0.17
60	733	1	−4.10	−3.90	0.20
251	725	1	−3.80	−3.59	0.22

Continued on next page

Supplementary Table 17: Energetic and electronic properties for [$Cu_{11}Zn_6$] cores obtained with PBE/light-tier1 level and light SCF parameters: configuration number (i), relative total energy (ΔE_{tot}), total magnetic moment (m_{tot}), HOMO energy (ε_H), LUMO energy (ε_L), and LUMO-HOMO energy gap (E_g).

i	ΔE_{tot} (meV)	m_{tot} (μ_B)	ε_H (eV)	ε_L (eV)	E_g (eV)
88	725	1	−4.18	−3.98	0.20
230	722	1	−3.82	−3.61	0.21
260	720	1	−3.96	−3.69	0.27
253	706	1	−4.02	−3.81	0.22
212	701	1	−3.94	−3.75	0.19
283	700	1	−4.17	−3.96	0.21
224	699	1	−4.00	−3.76	0.24
21	695	1	−3.91	−3.73	0.18
26	687	1	−3.77	−3.54	0.23
137	683	1	−3.95	−3.78	0.17
289	682	1	−3.82	−3.61	0.21
59	681	1	−3.98	−3.75	0.23
287	676	1	−3.85	−3.63	0.22
257	675	1	−3.70	−3.51	0.19
195	674	1	−4.09	−3.86	0.23
8	671	1	−3.98	−3.79	0.19
229	667	1	−3.77	−3.56	0.21
262	664	1	−4.02	−3.83	0.19
36	655	1	−3.90	−3.73	0.17
252	653	1	−3.83	−3.61	0.22
184	653	1	−4.01	−3.82	0.20
173	648	1	−3.78	−3.57	0.21

Continued on next page

Supplementary Table 17: Energetic and electronic properties for $[Cu_{11}Zn_6]$ cores obtained with PBE/light-tier1 level and light SCF parameters: configuration number (i), relative total energy (ΔE_{tot}), total magnetic moment (m_{tot}), HOMO energy (ε_H), LUMO energy (ε_L), and LUMO-HOMO energy gap (E_g).

i	ΔE_{tot} (meV)	m_{tot} (μ_B)	ε_H (eV)	ε_L (eV)	E_g (eV)
286	646	1	−3.88	−3.65	0.24
208	645	1	−4.09	−3.92	0.17
291	641	1	−3.72	−3.50	0.22
285	638	1	−3.90	−3.67	0.23
162	635	1	−3.77	−3.54	0.22
144	634	1	−4.14	−3.95	0.19
113	632	1	−3.95	−3.75	0.20
138	625	1	−4.19	−4.00	0.19
276	623	1	−4.20	−4.02	0.18
254	623	1	−3.91	−3.68	0.23
15	620	1	−3.99	−3.81	0.18
122	613	1	−3.96	−3.81	0.16
140	600	1	−3.88	−3.65	0.23
199	597	1	−3.94	−3.68	0.25
98	597	1	−4.02	−3.84	0.18
293	595	1	−4.03	−3.84	0.19
239	593	1	−3.71	−3.50	0.21
141	590	1	−3.90	−3.72	0.18
246	588	1	−3.93	−3.70	0.23
215	587	1	−3.90	−3.70	0.21
263	586	1	−3.83	−3.67	0.16
172	581	1	−3.95	−3.76	0.19

Continued on next page

Supplementary Table 17: Energetic and electronic properties for $[Cu_{11}Zn_6]$ cores obtained with PBE/light-tier1 level and light SCF parameters: configuration number (i), relative total energy (ΔE_{tot}), total magnetic moment (m_{tot}), HOMO energy (ε_H), LUMO energy (ε_L), and LUMO-HOMO energy gap (E_g).

i	ΔE_{tot} (meV)	m_{tot} (μ_B)	ε_H (eV)	ε_L (eV)	E_g (eV)
112	572	1	−4.11	−3.91	0.20
223	570	1	−3.99	−3.76	0.23
284	562	1	−3.98	−3.78	0.21
31	551	1	−3.82	−3.57	0.24
232	544	1	−3.99	−3.76	0.23
183	542	1	−4.21	−4.04	0.17
233	538	1	−3.72	−3.48	0.24
278	527	1	−3.84	−3.67	0.18
160	512	1	−3.83	−3.62	0.21
158	499	1	−3.87	−3.69	0.18
298	495	1	−3.94	−3.68	0.26
282	494	1	−3.83	−3.59	0.24
192	493	1	−3.86	−3.68	0.19
164	486	1	−4.00	−3.83	0.17
2	464	1	−4.03	−3.82	0.21
81	453	1	−3.81	−3.62	0.19
201	436	1	−3.98	−3.79	0.19
77	404	1	−4.11	−3.93	0.18
231	399	1	−3.98	−3.72	0.25
189	384	1	−3.96	−3.77	0.19
125	384	1	−3.86	−3.66	0.20
198	376	1	−3.81	−3.64	0.17

Continued on next page

Supplementary Table 17: Energetic and electronic properties for [$Cu_{11}Zn_6$] cores obtained with PBE/light-tier1 level and light SCF parameters: configuration number (i), relative total energy (ΔE_{tot}), total magnetic moment (m_{tot}), HOMO energy (ε_H), LUMO energy (ε_L), and LUMO-HOMO energy gap (E_g).

i	ΔE_{tot} (meV)	m_{tot} (μ_B)	ε_H (eV)	ε_L (eV)	E_g (eV)
78	355	1	−4.06	−3.89	0.17
84	352	1	−4.10	−3.93	0.18
116	351	1	−3.97	−3.79	0.18
258	350	1	−4.12	−3.91	0.21
290	350	1	−4.03	−3.79	0.24
185	335	1	−3.77	−3.58	0.19
61	334	1	−3.96	−3.78	0.18
256	320	1	−3.85	−3.63	0.22
222	285	1	−4.00	−3.83	0.17
169	274	1	−4.19	−3.97	0.22
119	269	1	−3.85	−3.66	0.19
277	262	1	−4.04	−3.79	0.25
28	262	1	−4.01	−3.82	0.19
70	261	1	−3.85	−3.68	0.17
225	240	1	−3.92	−3.71	0.21
267	237	1	−4.08	−3.92	0.16
275	211	1	−4.13	−3.94	0.18
249	179	1	−3.95	−3.74	0.22
104	151	1	−3.86	−3.64	0.22
3	81	1	−3.94	−3.69	0.25
245	77	1	−3.84	−3.63	0.21
161	52	1	−3.85	−3.67	0.18

Continued on next page

Supplementary Table 17: Energetic and electronic properties for [$Cu_{11}Zn_6$] cores obtained with PBE/light-tier1 level and light SCF parameters: configuration number (i), relative total energy (ΔE_{tot}), total magnetic moment (m_{tot}), HOMO energy (ε_H), LUMO energy (ε_L), and LUMO-HOMO energy gap (E_g).

i	ΔE_{tot} (meV)	m_{tot} (μ_B)	ε_H (eV)	ε_L (eV)	E_g (eV)
248	6	1	−4.05	−3.85	0.20
181	0	1	−3.94	−3.73	0.20

2.6.8 [Cu_8Al_6]

Supplementary Table 18: Energetic and electronic properties for [Cu_8Al_6] cores obtained with PBE/light-tier1 level and light SCF parameters: configuration number (i), relative total energy (ΔE_{tot}), total magnetic moment (m_{tot}), HOMO energy (ε_H), LUMO energy (ε_L), and LUMO-HOMO energy gap (E_g).

i	ΔE_{tot} (meV)	m_{tot} (μ_B)	ε_H (eV)	ε_L (eV)	E_g (eV)
140	1860	2	−4.02	−3.78	0.24
55	1848	2	−4.08	−3.80	0.28
6	1784	4	−3.88	−3.64	0.24
185	1738	2	−4.30	−4.08	0.22
154	1680	0	−4.29	−3.72	0.57
162	1678	2	−4.07	−3.76	0.32
181	1663	4	−3.96	−3.85	0.11
178	1635	2	−3.93	−3.70	0.23
157	1609	2	−3.99	−3.83	0.17
194	1508	2	−4.20	−4.02	0.18
88	1483	2	−4.01	−3.89	0.12
10	1452	2	−4.13	−3.86	0.27

Continued on next page

Supplementary Table 18: Energetic and electronic properties for [Cu_8Al_6] cores obtained with PBE/light-tier1 level and light SCF parameters: configuration number (i), relative total energy (ΔE_{tot}), total magnetic moment (m_{tot}), HOMO energy (ε_H), LUMO energy (ε_L), and LUMO-HOMO energy gap (E_g).

i	ΔE_{tot} (meV)	m_{tot} (μ_B)	ε_H (eV)	ε_L (eV)	E_g (eV)
43	1431	2	−3.87	−3.66	0.22
150	1417	2	−4.11	−3.96	0.15
149	1413	0	−4.23	−3.79	0.44
13	1403	2	−3.95	−3.73	0.22
176	1391	2	−4.02	−3.85	0.17
163	1388	2	−4.30	−4.06	0.24
184	1367	0	−4.27	−3.75	0.52
137	1362	0	−4.05	−3.76	0.29
145	1361	0	−4.08	−3.84	0.24
63	1344	2	−4.00	−3.96	0.03
95	1340	0	−4.04	−3.63	0.41
46	1318	2	−4.16	−3.93	0.24
75	1318	0	−4.18	−3.59	0.59
39	1311	2	−4.25	−3.96	0.29
34	1305	2	−3.99	−3.70	0.28
105	1297	2	−3.92	−3.79	0.12
32	1292	2	−4.00	−3.91	0.08
108	1284	2	−4.00	−3.74	0.26
122	1256	2	−4.01	−3.83	0.18
49	1254	0	−4.22	−3.80	0.42
47	1248	2	−4.09	−3.91	0.18
4	1247	0	−4.11	−3.72	0.39

Continued on next page

Supplementary Table 18: Energetic and electronic properties for $[Cu_8Al_6]$ cores obtained with PBE/light-tier1 level and light SCF parameters: configuration number (i), relative total energy (ΔE_{tot}), total magnetic moment (m_{tot}), HOMO energy (ε_H), LUMO energy (ε_L), and LUMO-HOMO energy gap (E_g).

i	ΔE_{tot} (meV)	m_{tot} (μ_B)	ε_H (eV)	ε_L (eV)	E_g (eV)
158	1240	2	−4.18	−3.98	0.21
83	1237	2	−4.22	−3.93	0.29
50	1232	2	−4.05	−3.87	0.18
116	1232	0	−4.26	−3.77	0.50
133	1231	0	−4.09	−3.64	0.45
124	1227	2	−3.93	−3.63	0.30
146	1226	2	−3.96	−3.90	0.06
155	1222	0	−4.38	−3.65	0.74
115	1221	2	−3.92	−3.63	0.29
74	1210	2	−4.11	−3.87	0.23
22	1203	2	−3.89	−3.72	0.17
132	1201	0	−4.21	−3.60	0.61
3	1191	2	−4.00	−3.63	0.37
175	1184	2	−3.97	−3.66	0.31
85	1178	2	−4.07	−3.91	0.17
78	1178	0	−4.20	−3.76	0.44
53	1178	2	−3.99	−3.83	0.15
18	1167	2	−4.15	−4.02	0.13
193	1166	2	−3.98	−3.90	0.07
106	1162	2	−3.75	−3.63	0.12
200	1155	2	−4.08	−3.84	0.24
71	1151	2	−4.20	−3.93	0.27

Continued on next page

Supplementary Table 18: Energetic and electronic properties for [Cu_8Al_6] cores obtained with PBE/light-tier1 level and light SCF parameters: configuration number (i), relative total energy (ΔE_{tot}), total magnetic moment (m_{tot}), HOMO energy (ε_H), LUMO energy (ε_L), and LUMO-HOMO energy gap (E_g).

i	ΔE_{tot} (meV)	m_{tot} (μ_B)	ε_H (eV)	ε_L (eV)	E_g (eV)
89	1137	2	−4.21	−4.01	0.19
16	1117	2	−4.11	−3.90	0.21
135	1112	2	−4.10	−3.88	0.22
134	1109	2	−4.08	−4.03	0.05
69	1106	2	−4.17	−4.02	0.14
126	1106	2	−4.07	−3.89	0.18
66	1094	2	−3.99	−3.88	0.10
72	1093	2	−4.06	−3.90	0.17
97	1080	0	−4.33	−3.75	0.57
142	1078	0	−4.27	−3.88	0.39
187	1078	2	−3.99	−3.67	0.32
130	1066	2	−4.07	−3.80	0.26
52	1063	2	−3.96	−3.91	0.05
28	1057	2	−4.09	−3.92	0.17
197	1054	2	−4.05	−3.87	0.18
144	1047	0	−4.24	−3.87	0.37
114	1031	0	−4.24	−3.78	0.46
198	1028	2	−4.24	−4.00	0.24
110	1023	2	−3.99	−3.91	0.08
120	1022	0	−4.06	−3.70	0.36
125	1021	2	−3.96	−3.70	0.25
102	1016	2	−4.02	−3.86	0.16

Continued on next page

Supplementary Table 18: Energetic and electronic properties for $[Cu_8Al_6]$ cores obtained with PBE/light-tier1 level and light SCF parameters: configuration number (i), relative total energy (ΔE_{tot}), total magnetic moment (m_{tot}), HOMO energy (ε_H), LUMO energy (ε_L), and LUMO-HOMO energy gap (E_g).

i	ΔE_{tot} (meV)	m_{tot} (μ_B)	ε_H (eV)	ε_L (eV)	E_g (eV)
173	1001	2	−4.20	−4.02	0.18
54	997	2	−3.93	−3.70	0.23
42	993	0	−4.24	−3.66	0.58
107	993	2	−3.86	−3.78	0.08
136	990	0	−4.16	−3.55	0.60
186	987	0	−4.13	−3.60	0.53
31	987	2	−4.25	−4.05	0.20
113	980	0	−3.98	−3.53	0.45
183	978	2	−4.08	−3.72	0.36
33	975	2	−3.84	−3.79	0.05
19	966	0	−4.25	−3.81	0.44
30	956	2	−4.10	−3.97	0.13
188	955	2	−4.09	−3.83	0.26
160	952	2	−3.78	−3.74	0.05
192	946	0	−3.94	−3.74	0.20
77	943	2	−4.11	−4.05	0.06
1	938	2	−4.09	−3.76	0.33
15	937	2	−4.09	−3.85	0.24
36	936	2	−3.85	−3.66	0.19
80	930	2	−4.14	−3.94	0.21
7	928	2	−3.85	−3.66	0.19
70	927	2	−4.02	−3.97	0.06

Continued on next page

Supplementary Table 18: Energetic and electronic properties for [Cu_8Al_6] cores obtained with PBE/light-tier1 level and light SCF parameters: configuration number (i), relative total energy (ΔE_{tot}), total magnetic moment (m_{tot}), HOMO energy (ε_H), LUMO energy (ε_L), and LUMO-HOMO energy gap (E_g).

i	ΔE_{tot} (meV)	m_{tot} (μ_B)	ε_H (eV)	ε_L (eV)	E_g (eV)
45	908	2	−4.08	−3.92	0.15
104	894	2	−3.79	−3.76	0.03
117	888	2	−4.14	−3.94	0.21
111	877	2	−3.86	−3.67	0.19
159	871	2	−4.24	−3.96	0.28
179	868	0	−3.97	−3.49	0.48
35	867	0	−4.23	−3.59	0.64
23	866	2	−3.89	−3.63	0.25
27	863	2	−4.25	−4.16	0.09
169	857	2	−4.03	−3.77	0.26
67	841	2	−3.89	−3.81	0.09
112	841	2	−3.83	−3.61	0.22
152	840	2	−3.92	−3.84	0.09
65	837	2	−4.13	−3.99	0.14
123	835	2	−3.95	−3.82	0.12
172	828	2	−3.94	−3.83	0.11
51	813	2	−3.96	−3.84	0.12
5	803	0	−4.03	−3.48	0.55
164	803	0	−4.14	−3.59	0.55
167	802	2	−4.11	−3.84	0.27
82	802	0	−4.20	−3.63	0.57
79	800	0	−4.15	−3.56	0.59

Continued on next page

Supplementary Table 18: Energetic and electronic properties for $[Cu_8Al_6]$ cores obtained with PBE/light-tier1 level and light SCF parameters: configuration number (i), relative total energy (ΔE_{tot}), total magnetic moment (m_{tot}), HOMO energy (ε_H), LUMO energy (ε_L), and LUMO-HOMO energy gap (E_g).

i	ΔE_{tot} (meV)	m_{tot} (μ_B)	ε_H (eV)	ε_L (eV)	E_g (eV)
147	798	0	−4.16	−3.70	0.46
41	767	0	−4.36	−3.71	0.66
40	761	0	−4.32	−3.68	0.64
156	759	0	−4.17	−3.59	0.58
81	758	2	−3.95	−3.81	0.14
98	742	0	−3.91	−3.60	0.31
161	725	0	−4.25	−3.61	0.64
84	712	0	−4.21	−3.63	0.58
86	710	0	−4.26	−3.61	0.64
24	700	0	−4.14	−3.59	0.55
99	699	2	−4.06	−3.89	0.17
8	699	2	−3.97	−3.80	0.17
93	689	2	−4.01	−3.84	0.17
182	689	2	−4.00	−3.86	0.14
129	683	2	−3.99	−3.77	0.22
12	676	2	−4.03	−3.74	0.28
139	676	0	−4.13	−3.69	0.44
119	675	0	−4.17	−3.86	0.32
189	662	0	−4.31	−3.77	0.55
59	656	2	−4.08	−3.99	0.09
48	650	0	−4.16	−3.64	0.52
166	645	2	−4.09	−3.84	0.25

Continued on next page

Supplementary Table 18: Energetic and electronic properties for [Cu_8Al_6] cores obtained with PBE/light-tier1 level and light SCF parameters: configuration number (i), relative total energy (ΔE_{tot}), total magnetic moment (m_{tot}), HOMO energy (ε_H), LUMO energy (ε_L), and LUMO-HOMO energy gap (E_g).

i	ΔE_{tot} (meV)	m_{tot} (μ_B)	ε_H (eV)	ε_L (eV)	E_g (eV)
29	643	2	−4.14	−3.96	0.18
131	631	0	−4.32	−3.68	0.63
94	626	0	−4.33	−3.78	0.55
90	624	0	−4.07	−3.77	0.30
96	624	0	−4.30	−3.85	0.45
38	588	0	−4.29	−3.55	0.74
148	583	0	−4.28	−3.66	0.62
199	580	0	−4.45	−3.46	0.99
143	571	0	−4.26	−3.71	0.56
195	544	0	−4.10	−3.55	0.55
171	544	0	−4.21	−3.68	0.53
103	543	2	−4.07	−3.84	0.23
190	539	0	−4.30	−3.59	0.71
128	496	0	−4.22	−3.69	0.53
101	470	0	−4.06	−3.55	0.51
57	460	0	−4.18	−3.58	0.60
177	458	2	−4.02	−3.78	0.24
127	447	0	−4.27	−3.65	0.62
62	435	0	−4.08	−3.65	0.42
196	428	0	−4.25	−3.56	0.68
58	399	0	−4.33	−3.63	0.70
60	397	0	−4.23	−3.73	0.50

Continued on next page

Supplementary Table 18: Energetic and electronic properties for [Cu_8Al_6] cores obtained with PBE/light-tier1 level and light SCF parameters: configuration number (i), relative total energy (ΔE_{tot}), total magnetic moment (m_{tot}), HOMO energy (ε_H), LUMO energy (ε_L), and LUMO-HOMO energy gap (E_g).

i	ΔE_{tot} (meV)	m_{tot} (μ_B)	ε_H (eV)	ε_L (eV)	E_g (eV)
121	397	0	−4.07	−3.79	0.27
109	381	0	−4.10	−3.54	0.56
44	376	0	−4.13	−3.70	0.43
153	371	0	−4.10	−3.63	0.47
91	353	0	−3.97	−3.63	0.34
92	351	0	−4.08	−3.79	0.30
56	343	0	−4.11	−3.37	0.74
180	335	0	−4.18	−3.54	0.64
2	328	0	−4.12	−3.79	0.33
61	325	0	−4.26	−3.89	0.38
11	323	0	−4.31	−3.46	0.85
17	320	0	−4.31	−3.52	0.79
9	307	0	−4.09	−3.48	0.60
174	298	0	−4.31	−3.42	0.88
100	296	0	−4.14	−3.57	0.57
64	281	0	−4.36	−3.64	0.72
138	248	2	−3.85	−3.72	0.13
26	239	0	−4.26	−3.57	0.69
37	237	0	−4.31	−3.78	0.53
165	222	0	−4.23	−3.51	0.72
73	181	0	−4.14	−3.46	0.69
14	159	0	−4.30	−3.52	0.78

Continued on next page

Supplementary Table 18: Energetic and electronic properties for [Cu_8Al_6] cores obtained with PBE/light-tier1 level and light SCF parameters: configuration number (i), relative total energy (ΔE_{tot}), total magnetic moment (m_{tot}), HOMO energy (ε_H), LUMO energy (ε_L), and LUMO-HOMO energy gap (E_g).

i	ΔE_{tot} (meV)	m_{tot} (μ_B)	ε_H (eV)	ε_L (eV)	E_g (eV)
20	76	0	−4.13	−3.51	0.62
168	51	0	−4.24	−3.60	0.64
170	43	0	−4.15	−3.72	0.42
87	37	0	−4.38	−3.66	0.73
191	0	0	−4.32	−3.54	0.78

2.6.9 [Ni_7Ga_6]

Supplementary Table 19: Energetic and electronic properties for [Ni_7Ga_6] cores obtained with PBE/light-tier1 level and light SCF parameters: configuration number (i), relative total energy (ΔE_{tot}), total magnetic moment (m_{tot}), HOMO energy (ε_H), LUMO energy (ε_L), and LUMO-HOMO energy gap (E_g).

i	ΔE_{tot} (meV)	m_{tot} (μ_B)	ε_H (eV)	ε_L (eV)	E_g (eV)
195	2478	4	−4.02	−3.89	0.13
75	2441	4	−3.88	−3.76	0.12
71	2106	4	−3.90	−3.77	0.13
153	2088	2	−3.90	−3.69	0.21
119	2080	4	−3.86	−3.81	0.05
16	2029	4	−3.99	−3.88	0.11
55	2009	4	−4.06	−3.84	0.22
38	2006	0	−3.92	−3.74	0.19
29	1987	2	−3.91	−3.66	0.25

Continued on next page

Supplementary Table 19: Energetic and electronic properties for [Ni_7Ga_6] cores obtained with PBE/light-tier1 level and light SCF parameters: configuration number (i), relative total energy (ΔE_{tot}), total magnetic moment (m_{tot}), HOMO energy (ε_H), LUMO energy (ε_L), and LUMO-HOMO energy gap (E_g).

i	ΔE_{tot} (meV)	m_{tot} (μ_B)	ε_H (eV)	ε_L (eV)	E_g (eV)
133	1949	4	−3.89	−3.73	0.16
39	1884	2	−3.89	−3.67	0.22
33	1880	2	−3.92	−3.72	0.20
94	1834	4	−3.90	−3.80	0.10
26	1820	4	−4.00	−3.86	0.14
27	1814	2	−3.90	−3.73	0.18
126	1792	4	−3.94	−3.83	0.11
179	1782	2	−3.93	−3.75	0.18
15	1780	2	−3.89	−3.57	0.32
24	1744	4	−4.10	−3.87	0.23
134	1735	4	−3.94	−3.75	0.19
146	1722	2	−3.95	−3.76	0.19
43	1700	4	−3.89	−3.79	0.10
62	1681	2	−3.86	−3.68	0.18
199	1677	2	−3.94	−3.74	0.20
103	1663	2	−3.83	−3.62	0.21
92	1638	2	−3.99	−3.76	0.23
152	1635	4	−3.95	−3.77	0.18
22	1635	4	−4.06	−3.90	0.16
175	1634	2	−3.90	−3.68	0.22
69	1629	2	−3.88	−3.79	0.10
129	1618	2	−3.92	−3.73	0.19

Continued on next page

Supplementary Table 19: Energetic and electronic properties for [Ni_7Ga_6] cores obtained with PBE/light-tier1 level and light SCF parameters: configuration number (i), relative total energy (ΔE_{tot}), total magnetic moment (m_{tot}), HOMO energy (ε_H), LUMO energy (ε_L), and LUMO-HOMO energy gap (E_g).

i	ΔE_{tot} (meV)	m_{tot} (μ_B)	ε_H (eV)	ε_L (eV)	E_g (eV)
161	1591	2	−3.97	−3.81	0.16
148	1591	2	−3.93	−3.73	0.20
23	1589	4	−3.99	−3.83	0.15
123	1551	0	−3.96	−3.73	0.24
137	1545	2	−3.92	−3.68	0.24
127	1527	2	−3.96	−3.66	0.30
34	1522	2	−3.86	−3.71	0.15
57	1522	2	−3.91	−3.74	0.18
115	1516	2	−3.89	−3.82	0.07
66	1513	4	−3.90	−3.81	0.09
113	1500	2	−3.94	−3.74	0.20
67	1494	2	−4.08	−3.95	0.13
190	1487	4	−4.09	−3.91	0.17
32	1463	0	−3.88	−3.48	0.40
101	1451	2	−3.97	−3.75	0.22
74	1448	2	−3.90	−3.83	0.06
110	1429	2	−3.84	−3.71	0.14
130	1414	4	−3.98	−3.84	0.14
58	1385	2	−3.96	−3.78	0.18
131	1384	2	−3.97	−3.78	0.19
176	1373	2	−3.82	−3.56	0.27
187	1365	2	−3.91	−3.72	0.19

Continued on next page

Supplementary Table 19: Energetic and electronic properties for $[Ni_7Ga_6]$ cores obtained with PBE/light-tier1 level and light SCF parameters: configuration number (i), relative total energy (ΔE_{tot}), total magnetic moment (m_{tot}), HOMO energy (ε_H), LUMO energy (ε_L), and LUMO-HOMO energy gap (E_g).

i	ΔE_{tot} (meV)	m_{tot} (μ_B)	ε_H (eV)	ε_L (eV)	E_g (eV)
141	1364	0	−3.94	−3.65	0.28
128	1364	2	−3.95	−3.75	0.20
149	1358	2	−3.86	−3.69	0.17
186	1347	2	−3.86	−3.67	0.19
96	1327	4	−4.04	−3.90	0.14
147	1304	2	−3.90	−3.62	0.28
154	1300	2	−3.92	−3.72	0.21
160	1297	2	−3.90	−3.77	0.13
135	1236	2	−4.10	−3.93	0.18
136	1234	2	−3.81	−3.78	0.03
200	1225	2	−3.92	−3.78	0.14
65	1222	4	−4.00	−3.95	0.05
117	1210	2	−3.87	−3.67	0.20
139	1179	4	−4.02	−3.82	0.20
70	1177	2	−3.97	−3.68	0.30
95	1175	4	−4.06	−3.94	0.12
80	1175	2	−4.02	−3.83	0.19
158	1163	2	−3.88	−3.71	0.17
49	1160	2	−3.74	−3.60	0.14
105	1135	2	−3.86	−3.67	0.20
109	1121	2	−4.10	−3.94	0.16
40	1114	2	−3.90	−3.64	0.25

Continued on next page

Supplementary Table 19: Energetic and electronic properties for [Ni_7Ga_6] cores obtained with PBE/light-tier1 level and light SCF parameters: configuration number (i), relative total energy (ΔE_{tot}), total magnetic moment (m_{tot}), HOMO energy (ε_H), LUMO energy (ε_L), and LUMO-HOMO energy gap (E_g).

i	ΔE_{tot} (meV)	m_{tot} (μ_B)	ε_H (eV)	ε_L (eV)	E_g (eV)
174	1113	2	−4.03	−3.77	0.26
50	1099	2	−3.99	−3.69	0.30
142	1098	2	−3.83	−3.63	0.21
102	1086	2	−3.95	−3.64	0.31
118	1070	4	−3.84	−3.76	0.08
37	1065	0	−3.86	−3.62	0.24
53	1062	2	−3.91	−3.79	0.12
185	1060	2	−3.89	−3.79	0.10
169	1055	2	−3.90	−3.80	0.10
145	1054	2	−3.94	−3.77	0.17
125	1050	2	−3.82	−3.76	0.06
25	1046	2	−3.99	−3.78	0.21
132	1033	2	−4.03	−3.82	0.21
138	1024	2	−4.01	−3.73	0.29
143	1013	2	−3.93	−3.75	0.19
60	1010	2	−3.99	−3.74	0.25
82	1005	0	−4.05	−3.82	0.23
20	998	2	−3.93	−3.64	0.29
193	993	2	−3.91	−3.82	0.10
116	991	2	−3.94	−3.79	0.14
151	991	2	−4.09	−3.80	0.30
97	991	2	−4.03	−3.73	0.29

Continued on next page

Supplementary Table 19: Energetic and electronic properties for [Ni_7Ga_6] cores obtained with PBE/light-tier1 level and light SCF parameters: configuration number (i), relative total energy (ΔE_{tot}), total magnetic moment (m_{tot}), HOMO energy (ε_H), LUMO energy (ε_L), and LUMO-HOMO energy gap (E_g).

i	ΔE_{tot} (meV)	m_{tot} (μ_B)	ε_H (eV)	ε_L (eV)	E_g (eV)
81	989	4	−4.06	−3.94	0.12
48	986	2	−4.01	−3.80	0.21
83	983	2	−4.14	−4.02	0.12
156	978	2	−3.98	−3.82	0.16
72	966	2	−3.98	−3.74	0.24
61	948	4	−4.05	−3.89	0.17
1	938	2	−3.99	−3.82	0.17
178	935	2	−3.79	−3.64	0.16
167	927	2	−3.89	−3.69	0.19
191	924	2	−3.91	−3.72	0.19
171	920	2	−3.98	−3.80	0.18
90	918	2	−4.04	−3.88	0.16
111	914	0	−3.99	−3.73	0.26
17	908	2	−3.94	−3.78	0.17
64	907	2	−3.99	−3.88	0.11
56	891	2	−3.93	−3.72	0.20
31	891	2	−3.92	−3.72	0.20
150	887	2	−3.93	−3.82	0.11
35	880	2	−3.92	−3.71	0.21
121	873	2	−4.01	−3.83	0.17
164	860	2	−3.92	−3.70	0.21
183	855	2	−3.98	−3.80	0.18

Continued on next page

Supplementary Table 19: Energetic and electronic properties for [Ni_7Ga_6] cores obtained with PBE/light-tier1 level and light SCF parameters: configuration number (i), relative total energy (ΔE_{tot}), total magnetic moment (m_{tot}), HOMO energy (ε_H), LUMO energy (ε_L), and LUMO-HOMO energy gap (E_g).

i	ΔE_{tot} (meV)	m_{tot} (μ_B)	ε_H (eV)	ε_L (eV)	E_g (eV)
107	846	2	−4.05	−3.88	0.17
47	844	2	−3.94	−3.77	0.17
112	842	2	−3.99	−3.86	0.13
45	831	2	−3.91	−3.77	0.14
194	810	2	−4.06	−3.82	0.24
19	806	2	−3.91	−3.65	0.25
79	805	2	−4.01	−3.86	0.16
120	803	2	−3.93	−3.68	0.26
104	792	4	−4.20	−3.95	0.24
59	784	2	−4.06	−3.83	0.22
21	783	0	−4.04	−3.67	0.36
162	774	2	−4.00	−3.82	0.18
30	768	2	−4.19	−3.88	0.31
28	763	2	−3.90	−3.67	0.22
18	748	2	−3.86	−3.60	0.26
98	732	2	−4.03	−3.89	0.14
192	728	2	−4.15	−3.89	0.26
196	715	4	−4.11	−4.01	0.10
51	705	2	−3.92	−3.81	0.11
68	701	2	−4.10	−3.88	0.22
198	689	2	−4.06	−3.82	0.25
188	685	2	−4.05	−3.90	0.14

Continued on next page

Supplementary Table 19: Energetic and electronic properties for [Ni_7Ga_6] cores obtained with PBE/light-tier1 level and light SCF parameters: configuration number (i), relative total energy (ΔE_{tot}), total magnetic moment (m_{tot}), HOMO energy (ε_H), LUMO energy (ε_L), and LUMO-HOMO energy gap (E_g).

i	ΔE_{tot} (meV)	m_{tot} (μ_B)	ε_H (eV)	ε_L (eV)	E_g (eV)
197	679	0	−3.77	−3.53	0.24
166	673	0	−3.81	−3.44	0.37
189	666	2	−4.10	−3.82	0.28
44	665	2	−3.97	−3.72	0.25
89	663	0	−4.15	−3.95	0.19
106	662	2	−3.92	−3.77	0.15
63	657	2	−4.14	−3.92	0.22
52	649	2	−3.91	−3.72	0.19
155	643	2	−3.94	−3.79	0.14
124	637	2	−3.98	−3.76	0.21
122	622	2	−4.06	−3.73	0.32
114	618	2	−3.94	−3.84	0.10
11	612	4	−4.05	−3.85	0.19
144	601	2	−4.01	−3.79	0.23
41	584	2	−3.93	−3.61	0.31
2	573	2	−4.03	−3.86	0.18
93	562	2	−4.07	−3.81	0.27
77	537	2	−3.91	−3.65	0.26
184	521	2	−3.96	−3.65	0.31
88	513	2	−3.98	−3.84	0.15
4	510	2	−4.00	−3.82	0.18
76	498	2	−3.94	−3.69	0.25

Continued on next page

Supplementary Table 19: Energetic and electronic properties for $[Ni_7Ga_6]$ cores obtained with PBE/light-tier1 level and light SCF parameters: configuration number (i), relative total energy (ΔE_{tot}), total magnetic moment (m_{tot}), HOMO energy (ε_H), LUMO energy (ε_L), and LUMO-HOMO energy gap (E_g).

i	ΔE_{tot} (meV)	m_{tot} (μ_B)	ε_H (eV)	ε_L (eV)	E_g (eV)
170	471	2	−4.02	−3.86	0.17
3	470	2	−4.26	−4.08	0.18
14	463	2	−4.00	−3.86	0.14
36	461	2	−4.08	−3.89	0.19
13	458	0	−3.87	−3.62	0.25
91	457	2	−4.06	−3.94	0.12
78	405	0	−3.96	−3.76	0.20
140	404	2	−4.02	−3.77	0.25
157	402	2	−3.97	−3.82	0.15
73	393	2	−4.11	−3.78	0.33
86	386	2	−4.00	−3.87	0.13
163	358	4	−4.06	−4.00	0.06
173	337	0	−4.14	−3.90	0.24
108	326	2	−3.95	−3.82	0.13
172	319	2	−3.74	−3.60	0.14
42	284	2	−4.08	−3.84	0.24
46	283	2	−4.05	−3.84	0.22
180	281	0	−4.01	−3.83	0.18
9	269	2	−4.07	−3.69	0.38
85	262	2	−3.98	−3.76	0.22
181	260	0	−3.88	−3.60	0.28
159	251	2	−4.07	−3.88	0.20

Continued on next page

Supplementary Table 19: Energetic and electronic properties for [Ni_7Ga_6] cores obtained with PBE/light-tier1 level and light SCF parameters: configuration number (i), relative total energy (ΔE_{tot}), total magnetic moment (m_{tot}), HOMO energy (ε_H), LUMO energy (ε_L), and LUMO-HOMO energy gap (E_g).

i	ΔE_{tot} (meV)	m_{tot} (μ_B)	ε_H (eV)	ε_L (eV)	E_g (eV)
99	158	2	−3.88	−3.72	0.16
8	96	2	−4.02	−3.83	0.19
10	83	2	−4.02	−3.83	0.19
84	71	2	−3.98	−3.77	0.21
182	58	2	−4.05	−3.88	0.17
177	35	2	−4.01	−3.83	0.18
5	5	2	−4.01	−3.69	0.33
6	0	2	−4.12	−3.90	0.22

2.7 Additional Results for Metal Complexes

In the following subsections, we summarize a large number of results obtained for the computational design of molecular metal complexes. In particular, large part of the data is reported for the [Cu_aZn_b]R_n complexes and also results for miscellaneous complexes using `cluster_assembler` protocol summarized above.

2.7.1 $[CuZn_2](Cp^*)_3$ Complexes

Supplementary Table 20: Energetic and electronic properties for $[CuZn_2](Cp^*)_3$ complexes obtained with PBE/light-tier1 level and light SCF parameters: configuration number (i), relative total energy (ΔE_{tot}), total magnetic moment (m_{tot}), HOMO energy (ε_H), LUMO energy (ε_L), and LUMO-HOMO energy gap (E_g).

i	ΔE_{tot} (meV)	m_{tot} (μ_B)	ε_H (eV)	ε_L (eV)	E_g (eV)
15	6626	0	−3.64	−3.53	0.11
20	4578	0	−4.30	−3.52	0.77
14	3572	0	−4.16	−3.70	0.46
19	3551	0	−4.15	−3.70	0.45
13	3549	0	−4.21	−3.76	0.44
10	3534	0	−4.20	−3.75	0.45
5	3489	0	−4.24	−3.80	0.44
8	3486	0	−4.25	−3.81	0.44
18	3449	0	−4.17	−3.72	0.44
6	2438	0	−4.32	−2.49	1.84
17	431	0	−3.87	−1.52	2.35
1	91	0	−3.97	−1.48	2.49
9	3	0	−3.97	−1.38	2.60
3	1	0	−3.97	−1.38	2.60
11	1	0	−3.97	−1.37	2.59
2	1	0	−3.97	−1.37	2.60
16	0	0	−3.96	−1.36	2.61
7	0	0	−3.97	−1.37	2.60

Supplementary Table 21: Optimized and filtered representative structures of $[CuZn_2](Cp^*)_3$ complexes obtained with PBE/light-tier1 level and light SCF parameters. The relative total energy (ΔE_{tot}) is depicted above each structure.

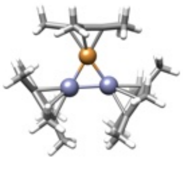

0 meV

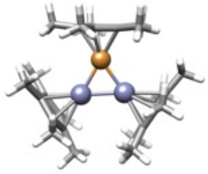

0.28 meV

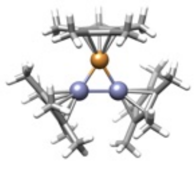

0.56 meV

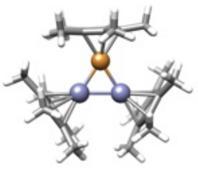

0.82 meV

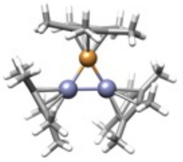

0.96 meV

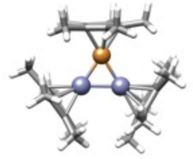

2.78 meV

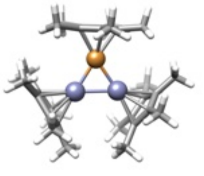

91 meV

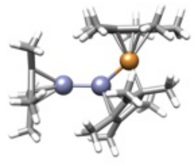

431 meV

2.7.2 $[Cu_3Zn_4](Cp^*)_5$ Complexes

Supplementary Table 22: Energetic and electronic properties for $[Cu_3Zn_4](Cp^*)_5]$ complexes obtained with PBE/light-tier1 level and light SCF parameters: configuration number (i), relative total energy (ΔE_{tot}), total magnetic moment (m_{tot}), HOMO energy (ε_H), LUMO energy (ε_L), and LUMO-HOMO energy gap (E_g).

i	ΔE_{tot} (meV)	m_{tot} (μ_B)	ε_H (eV)	ε_L (eV)	E_g (eV)
12	6403	0	−3.93	−3.51	0.42
124	6233	0	−3.68	−2.71	0.97
239	4815	0	−3.65	−2.87	0.78
233	4803	0	−3.83	−2.46	1.37
81	4676	0	−4.10	−2.82	1.28
2	4565	0	−3.68	−2.74	0.94
26	4469	0	−3.69	−3.47	0.22
8	4435	0	−4.03	−3.20	0.83
266	4305	0	−3.12	−2.95	0.17

Continued on next page

Supplementary Table 22: Energetic and electronic properties for $[Cu_3Zn_4](Cp^*)_5]$ complexes obtained with PBE/light-tier1 level and light SCF parameters: configuration number (i), relative total energy (ΔE_{tot}), total magnetic moment (m_{tot}), HOMO energy (ε_H), LUMO energy (ε_L), and LUMO-HOMO energy gap (E_g).

i	ΔE_{tot} (meV)	m_{tot} (μ_B)	ε_H (eV)	ε_L (eV)	E_g (eV)
4	4284	0	−3.73	−2.91	0.82
19	4228	0	−3.78	−3.52	0.26
216	4056	0	−3.82	−2.44	1.38
205	3800	0	−3.77	−2.97	0.79
154	3671	0	−3.92	−2.99	0.92
97	3452	0	−3.62	−2.92	0.70
44	3448	0	−3.81	−2.68	1.14
160	3419	0	−4.03	−2.86	1.17
146	3389	0	−3.90	−2.66	1.24
168	3329	0	−3.68	−2.64	1.04
223	3293	0	−4.00	−3.13	0.87
167	3241	0	−3.74	−3.16	0.57
227	3214	0	−3.89	−2.97	0.92
131	3147	0	−3.58	−2.58	1.00
183	3138	0	−3.63	−2.56	1.06
299	3129	0	−3.79	−2.88	0.91
291	3128	0	−3.88	−3.10	0.77
70	3100	0	−4.24	−2.56	1.67
204	2941	0	−3.95	−2.42	1.53
138	2924	0	−3.76	−2.48	1.28
256	2892	0	−3.85	−2.58	1.27
133	2869	0	−3.70	−3.60	0.10

Continued on next page

Supplementary Table 22: Energetic and electronic properties for $[Cu_3Zn_4](Cp^*)_5]$ complexes obtained with PBE/light-tier1 level and light SCF parameters: configuration number (i), relative total energy (ΔE_{tot}), total magnetic moment (m_{tot}), HOMO energy (ε_H), LUMO energy (ε_L), and LUMO-HOMO energy gap (E_g).

i	ΔE_{tot} (meV)	m_{tot} (μ_B)	ε_H (eV)	ε_L (eV)	E_g (eV)
33	2837	0	−4.13	−2.72	1.41
116	2758	0	−3.71	−2.61	1.10
232	2755	0	−3.82	−2.94	0.88
215	2704	0	−3.32	−2.99	0.33
217	2685	0	−3.44	−2.62	0.81
286	2671	0	−3.90	−2.54	1.36
293	2666	0	−3.62	−2.53	1.09
241	2663	0	−4.05	−2.90	1.15
78	2621	0	−3.59	−3.15	0.44
222	2512	0	−3.65	−2.70	0.94
31	2502	0	−3.95	−2.71	1.24
326	2445	0	−3.77	−2.59	1.18
150	2350	0	−3.74	−2.59	1.15
282	2338	0	−4.17	−2.99	1.18
255	2313	0	−4.00	−2.76	1.24
86	2294	0	−4.13	−3.03	1.10
55	2252	0	−4.31	−3.21	1.11
37	2209	0	−3.96	−2.93	1.02
111	2208	0	−3.51	−2.64	0.87
247	2204	0	−4.15	−3.35	0.80
56	2201	0	−4.21	−3.26	0.95
308	2176	0	−4.18	−2.58	1.60

Continued on next page

Supplementary Table 22: Energetic and electronic properties for $[Cu_3Zn_4](Cp^*)_5]$ complexes obtained with PBE/light-tier1 level and light SCF parameters: configuration number (i), relative total energy (ΔE_{tot}), total magnetic moment (m_{tot}), HOMO energy (ε_H), LUMO energy (ε_L), and LUMO-HOMO energy gap (E_g).

i	ΔE_{tot} (meV)	m_{tot} (μ_B)	ε_H (eV)	ε_L (eV)	E_g (eV)
182	2133	0	−3.99	−3.29	0.69
115	2130	0	−3.82	−2.76	1.05
325	2123	0	−3.69	−2.74	0.95
54	2123	0	−4.24	−3.06	1.18
9	2114	0	−3.84	−2.77	1.07
93	2104	0	−4.19	−2.76	1.42
60	2085	0	−3.79	−2.73	1.05
149	2084	0	−3.92	−2.77	1.15
68	2069	0	−3.84	−2.25	1.60
109	2062	0	−3.52	−2.66	0.86
100	2062	0	−4.10	−3.02	1.08
288	2057	0	−4.14	−2.65	1.49
3	2055	0	−3.88	−2.77	1.11
158	2026	0	−4.26	−2.66	1.60
161	2025	0	−3.88	−2.87	1.01
218	2020	0	−3.71	−2.60	1.11
63	2009	0	−3.83	−2.59	1.23
113	2005	0	−3.96	−2.68	1.28
324	1988	0	−3.75	−2.59	1.16
297	1966	0	−4.00	−2.12	1.88
172	1962	0	−4.05	−3.54	0.52
274	1949	0	−3.80	−2.94	0.86

Continued on next page

Supplementary Table 22: Energetic and electronic properties for $[Cu_3Zn_4](Cp^*)_5]$ complexes obtained with PBE/light-tier1 level and light SCF parameters: configuration number (i), relative total energy (ΔE_{tot}), total magnetic moment (m_{tot}), HOMO energy (ε_H), LUMO energy (ε_L), and LUMO-HOMO energy gap (E_g).

i	ΔE_{tot} (meV)	m_{tot} (μ_B)	ε_H (eV)	ε_L (eV)	E_g (eV)
84	1947	0	−3.99	−2.68	1.30
21	1946	0	−4.29	−2.93	1.35
173	1944	0	−3.84	−2.69	1.15
252	1940	0	−4.13	−2.46	1.67
300	1940	0	−3.98	−2.92	1.06
53	1937	0	−4.24	−3.17	1.08
126	1929	0	−4.25	−3.37	0.88
16	1924	0	−3.72	−2.72	1.00
235	1908	0	−4.00	−2.43	1.56
39	1894	0	−4.00	−2.46	1.54
226	1887	0	−3.74	−2.28	1.46
6	1882	0	−3.92	−2.45	1.47
157	1882	0	−3.87	−2.84	1.03
237	1881	0	−3.89	−2.41	1.49
238	1869	0	−4.05	−2.69	1.36
316	1868	0	−3.74	−2.57	1.17
87	1858	0	−3.67	−2.67	1.00
276	1853	0	−3.98	−2.37	1.61
127	1848	0	−4.41	−3.25	1.16
139	1839	0	−3.72	−2.58	1.15
275	1835	0	−3.80	−2.62	1.18
28	1827	0	−3.72	−2.73	1.00

Continued on next page

Supplementary Table 22: Energetic and electronic properties for $[Cu_3Zn_4](Cp^*)_5]$ complexes obtained with PBE/light-tier1 level and light SCF parameters: configuration number (i), relative total energy (ΔE_{tot}), total magnetic moment (m_{tot}), HOMO energy (ε_H), LUMO energy (ε_L), and LUMO-HOMO energy gap (E_g).

i	ΔE_{tot} (meV)	m_{tot} (μ_B)	ε_H (eV)	ε_L (eV)	E_g (eV)
176	1802	0	−3.83	−2.66	1.17
195	1796	0	−4.08	−2.37	1.72
27	1795	0	−4.27	−2.65	1.62
49	1790	0	−4.10	−2.69	1.41
61	1788	0	−4.33	−2.96	1.38
210	1785	0	−4.12	−2.52	1.60
229	1767	0	−3.87	−2.65	1.23
136	1767	0	−3.94	−2.34	1.60
190	1766	0	−3.84	−2.58	1.26
98	1761	0	−4.12	−2.73	1.40
231	1753	0	−4.03	−2.40	1.63
67	1747	0	−4.05	−2.45	1.60
321	1739	0	−3.78	−2.54	1.24
162	1736	0	−3.68	−2.46	1.22
320	1736	0	−3.63	−2.50	1.13
181	1729	0	−4.04	−2.24	1.80
311	1726	0	−3.89	−2.40	1.49
92	1724	0	−4.09	−2.36	1.74
128	1724	0	−3.52	−2.63	0.89
121	1723	0	−4.05	−2.86	1.19
265	1720	0	−3.78	−2.67	1.11
262	1719	0	−3.92	−2.51	1.41

Continued on next page

Supplementary Table 22: Energetic and electronic properties for $[Cu_3Zn_4](Cp^*)_5]$ complexes obtained with PBE/light-tier1 level and light SCF parameters: configuration number (i), relative total energy (ΔE_{tot}), total magnetic moment (m_{tot}), HOMO energy (ε_H), LUMO energy (ε_L), and LUMO-HOMO energy gap (E_g).

i	ΔE_{tot} (meV)	m_{tot} (μ_B)	ε_H (eV)	ε_L (eV)	E_g (eV)
187	1712	0	−3.93	−2.67	1.25
319	1710	0	−3.75	−2.06	1.69
110	1709	0	−3.98	−2.27	1.71
202	1702	0	−3.78	−2.28	1.50
108	1702	0	−3.89	−2.44	1.45
166	1696	0	−3.77	−2.56	1.21
250	1696	0	−3.86	−2.45	1.41
59	1694	0	−3.80	−2.28	1.51
203	1692	0	−4.08	−2.53	1.55
292	1679	0	−3.91	−2.48	1.43
212	1671	0	−4.20	−2.21	1.99
85	1666	0	−3.93	−2.62	1.31
290	1665	0	−4.01	−2.44	1.56
270	1664	0	−4.05	−2.59	1.45
244	1657	0	−3.90	−2.46	1.44
177	1655	0	−4.09	−2.59	1.50
82	1651	0	−3.64	−2.64	1.00
258	1648	0	−4.16	−2.73	1.43
101	1640	0	−3.72	−2.41	1.32
45	1638	0	−3.84	−2.62	1.21
137	1637	0	−3.59	−2.66	0.94
117	1636	0	−4.09	−2.54	1.56

Continued on next page

Supplementary Table 22: Energetic and electronic properties for $[Cu_3Zn_4](Cp^*)_5]$ complexes obtained with PBE/light-tier1 level and light SCF parameters: configuration number (i), relative total energy (ΔE_{tot}), total magnetic moment (m_{tot}), HOMO energy (ε_H), LUMO energy (ε_L), and LUMO-HOMO energy gap (E_g).

i	ΔE_{tot} (meV)	m_{tot} (μ_B)	ε_H (eV)	ε_L (eV)	E_g (eV)
22	1633	0	−3.82	−2.68	1.15
119	1632	0	−4.25	−2.79	1.46
130	1628	0	−3.90	−2.49	1.41
47	1619	0	−3.71	−2.16	1.55
180	1603	0	−3.97	−2.74	1.23
197	1601	0	−4.03	−2.59	1.45
189	1599	0	−3.98	−2.58	1.40
112	1593	0	−3.62	−2.27	1.35
277	1591	0	−4.14	−2.63	1.51
155	1588	0	−3.56	−2.65	0.91
169	1587	0	−3.55	−2.69	0.86
186	1582	0	−3.49	−2.52	0.97
164	1575	0	−3.68	−2.68	1.00
322	1574	0	−3.90	−2.69	1.21
13	1573	0	−3.94	−2.53	1.40
99	1566	0	−4.07	−2.26	1.81
199	1561	0	−4.13	−2.54	1.59
107	1560	0	−4.00	−2.45	1.56
14	1557	0	−4.02	−2.58	1.44
32	1554	0	−3.49	−2.52	0.97
224	1551	0	−3.74	−2.02	1.72
51	1548	0	−4.07	−2.69	1.38

Continued on next page

Supplementary Table 22: Energetic and electronic properties for $[Cu_3Zn_4](Cp^*)_5]$ complexes obtained with PBE/light-tier1 level and light SCF parameters: configuration number (i), relative total energy (ΔE_{tot}), total magnetic moment (m_{tot}), HOMO energy (ε_H), LUMO energy (ε_L), and LUMO-HOMO energy gap (E_g).

i	ΔE_{tot} (meV)	m_{tot} (μ_B)	ε_H (eV)	ε_L (eV)	E_g (eV)
135	1544	0	−3.91	−2.34	1.57
246	1542	0	−4.19	−2.87	1.32
185	1535	0	−3.86	−2.71	1.15
198	1531	0	−3.96	−2.50	1.46
36	1526	0	−3.83	−2.17	1.67
57	1524	0	−3.47	−2.52	0.95
174	1524	0	−4.01	−2.63	1.38
272	1518	0	−4.02	−2.52	1.50
71	1516	0	−4.04	−2.63	1.41
171	1514	0	−3.90	−2.43	1.47
15	1513	0	−4.05	−2.49	1.56
191	1508	0	−4.04	−2.39	1.65
65	1507	0	−4.04	−2.40	1.65
257	1507	0	−4.23	−2.55	1.68
122	1506	0	−3.89	−2.18	1.71
228	1504	0	−4.37	−2.58	1.80
46	1489	0	−3.60	−2.58	1.02
134	1477	0	−4.06	−2.62	1.43
208	1477	0	−3.80	−2.36	1.44
129	1472	0	−3.69	−2.64	1.05
118	1471	0	−4.12	−2.54	1.58
153	1461	0	−3.85	−2.48	1.37

Continued on next page

Supplementary Table 22: Energetic and electronic properties for $[Cu_3Zn_4](Cp^*)_5]$ complexes obtained with PBE/light-tier1 level and light SCF parameters: configuration number (i), relative total energy (ΔE_{tot}), total magnetic moment (m_{tot}), HOMO energy (ε_H), LUMO energy (ε_L), and LUMO-HOMO energy gap (E_g).

i	ΔE_{tot} (meV)	m_{tot} (μ_B)	ε_H (eV)	ε_L (eV)	E_g (eV)
38	1458	0	−3.98	−2.06	1.91
25	1457	0	−3.97	−2.27	1.70
50	1454	0	−3.62	−2.57	1.05
243	1451	0	−3.71	−2.17	1.55
201	1451	0	−4.14	−2.35	1.79
152	1449	0	−4.15	−2.58	1.57
5	1445	0	−4.09	−2.33	1.77
89	1440	0	−4.15	−2.43	1.71
211	1433	0	−3.86	−2.44	1.42
142	1431	0	−4.18	−2.47	1.71
236	1430	0	−4.04	−2.68	1.36
140	1425	0	−4.02	−2.65	1.37
96	1423	0	−3.76	−2.40	1.36
289	1421	0	−3.97	−2.32	1.64
75	1416	0	−3.87	−2.02	1.85
80	1413	0	−4.02	−2.18	1.84
280	1405	0	−4.05	−2.62	1.43
294	1404	0	−4.03	−2.27	1.76
145	1398	0	−3.70	−2.41	1.29
295	1389	0	−3.92	−2.32	1.60
42	1389	0	−4.24	−2.56	1.68
52	1385	0	−4.11	−2.21	1.89

Continued on next page

Supplementary Table 22: Energetic and electronic properties for $[Cu_3Zn_4](Cp^*)_5]$ complexes obtained with PBE/light-tier1 level and light SCF parameters: configuration number (i), relative total energy (ΔE_{tot}), total magnetic moment (m_{tot}), HOMO energy (ε_H), LUMO energy (ε_L), and LUMO-HOMO energy gap (E_g).

i	ΔE_{tot} (meV)	m_{tot} (μ_B)	ε_H (eV)	ε_L (eV)	E_g (eV)
317	1381	0	−4.43	−2.57	1.85
147	1380	0	−4.09	−2.76	1.32
90	1375	0	−4.09	−2.27	1.82
64	1373	0	−3.94	−2.45	1.49
285	1371	0	−3.80	−2.27	1.53
214	1368	0	−3.81	−2.37	1.44
151	1367	0	−3.74	−2.17	1.57
281	1367	0	−3.65	−2.24	1.41
58	1364	0	−3.94	−2.33	1.61
304	1360	0	−4.43	−2.70	1.73
296	1360	0	−3.97	−2.14	1.83
105	1353	0	−4.06	−2.22	1.85
248	1353	0	−4.30	−2.82	1.49
88	1347	0	−4.24	−1.86	2.37
125	1333	0	−4.12	−2.45	1.67
175	1331	0	−4.06	−2.43	1.63
264	1329	0	−3.95	−2.27	1.68
310	1329	0	−4.06	−2.31	1.75
23	1329	0	−4.06	−2.31	1.75
170	1328	0	−4.14	−2.50	1.64
77	1311	0	−3.94	−2.29	1.65
298	1309	0	−3.90	−2.32	1.58

Continued on next page

Supplementary Table 22: Energetic and electronic properties for $[Cu_3Zn_4](Cp^*)_5]$ complexes obtained with PBE/light-tier1 level and light SCF parameters: configuration number (i), relative total energy (ΔE_{tot}), total magnetic moment (m_{tot}), HOMO energy (ε_H), LUMO energy (ε_L), and LUMO-HOMO energy gap (E_g).

i	ΔE_{tot} (meV)	m_{tot} (μ_B)	ε_H (eV)	ε_L (eV)	E_g (eV)
69	1308	0	−4.12	−2.41	1.71
103	1298	0	−4.02	−2.68	1.35
144	1295	0	−4.07	−2.24	1.83
79	1292	0	−4.03	−2.14	1.88
327	1285	0	−4.16	−2.58	1.58
305	1285	0	−4.16	−2.58	1.58
143	1283	0	−4.16	−2.72	1.43
200	1282	0	−4.00	−2.10	1.90
73	1282	0	−4.09	−2.61	1.47
72	1281	0	−4.06	−2.59	1.47
273	1279	0	−4.19	−2.46	1.73
17	1279	0	−3.87	−2.45	1.42
213	1276	0	−3.94	−2.30	1.64
40	1276	0	−4.21	−2.49	1.72
263	1260	0	−3.90	−2.48	1.42
132	1259	0	−4.02	−2.56	1.47
179	1257	0	−4.09	−2.60	1.50
104	1254	0	−3.89	−1.99	1.90
62	1248	0	−3.90	−2.45	1.45
278	1248	0	−4.08	−2.49	1.59
312	1247	0	−4.32	−2.67	1.65
287	1242	0	−3.92	−2.05	1.87

Continued on next page

Supplementary Table 22: Energetic and electronic properties for $[Cu_3Zn_4](Cp^*)_5]$ complexes obtained with PBE/light-tier1 level and light SCF parameters: configuration number (i), relative total energy (ΔE_{tot}), total magnetic moment (m_{tot}), HOMO energy (ε_H), LUMO energy (ε_L), and LUMO-HOMO energy gap (E_g).

i	ΔE_{tot} (meV)	m_{tot} (μ_B)	ε_H (eV)	ε_L (eV)	E_g (eV)
219	1241	0	−3.78	−2.19	1.59
165	1238	0	−3.86	−2.59	1.27
268	1225	0	−4.25	−2.17	2.09
220	1225	0	−3.86	−2.40	1.45
83	1223	0	−4.01	−2.02	1.99
184	1216	0	−3.83	−2.41	1.42
43	1208	0	−4.17	−2.39	1.78
20	1207	0	−4.20	−2.29	1.91
35	1205	0	−3.96	−2.15	1.80
249	1194	0	−3.74	−2.61	1.13
234	1182	0	−4.08	−2.34	1.73
102	1182	0	−3.98	−2.36	1.63
209	1176	0	−4.00	−2.34	1.67
196	1166	0	−4.19	−2.89	1.30
30	1163	0	−4.03	−2.21	1.82
193	1157	0	−4.26	−2.90	1.37
7	1151	0	−4.09	−2.41	1.69
269	1151	0	−4.26	−2.13	2.12
34	1148	0	−4.07	−2.36	1.71
314	1144	0	−4.22	−1.95	2.27
221	1141	0	−4.27	−2.06	2.21
207	1133	0	−3.92	−2.28	1.64

Continued on next page

Supplementary Table 22: Energetic and electronic properties for $[Cu_3Zn_4](Cp^*)_5]$ complexes obtained with PBE/light-tier1 level and light SCF parameters: configuration number (i), relative total energy (ΔE_{tot}), total magnetic moment (m_{tot}), HOMO energy (ε_H), LUMO energy (ε_L), and LUMO-HOMO energy gap (E_g).

i	ΔE_{tot} (meV)	m_{tot} (μ_B)	ε_H (eV)	ε_L (eV)	E_g (eV)
1	1131	0	−4.16	−2.37	1.79
245	1123	0	−3.95	−2.10	1.85
267	1121	0	−4.22	−2.15	2.07
120	1118	0	−4.07	−1.94	2.13
283	1115	0	−4.08	−2.21	1.87
66	1114	0	−3.76	−2.67	1.08
76	1111	0	−4.06	−2.33	1.73
240	1110	0	−4.16	−2.40	1.76
260	1085	0	−3.97	−2.09	1.88
315	1078	0	−4.08	−1.97	2.11
284	1036	0	−4.16	−2.09	2.07
194	1019	0	−4.28	−2.05	2.23
192	1016	0	−3.95	−2.14	1.81
178	1013	0	−4.14	−2.19	1.95
148	1011	0	−3.62	−2.35	1.27
91	1007	0	−4.07	−2.37	1.69
279	999	0	−4.08	−2.23	1.86
41	997	0	−4.10	−2.29	1.81
309	991	0	−4.06	−2.27	1.79
94	985	0	−4.00	−2.17	1.83
259	976	0	−3.93	−1.91	2.02
141	971	0	−4.04	−1.94	2.10

Continued on next page

Supplementary Table 22: Energetic and electronic properties for $[Cu_3Zn_4](Cp^*)_5]$ complexes obtained with PBE/light-tier1 level and light SCF parameters: configuration number (i), relative total energy (ΔE_{tot}), total magnetic moment (m_{tot}), HOMO energy (ε_H), LUMO energy (ε_L), and LUMO-HOMO energy gap (E_g).

i	ΔE_{tot} (meV)	m_{tot} (μ_B)	ε_H (eV)	ε_L (eV)	E_g (eV)
188	970	0	−4.18	−2.36	1.82
261	959	0	−3.96	−1.93	2.03
251	949	0	−4.07	−2.02	2.05
163	935	0	−4.07	−2.13	1.93
230	927	0	−4.03	−2.02	2.01
225	920	0	−4.08	−2.19	1.89
18	888	0	−4.06	−2.19	1.87
48	871	0	−4.06	−2.23	1.83
74	811	0	−4.07	−2.12	1.95
114	772	0	−3.91	−2.27	1.64
254	739	0	−4.02	−2.62	1.40
11	678	0	−3.93	−2.36	1.57
271	635	0	−3.89	−2.33	1.57
253	599	0	−3.91	−2.26	1.64
307	0	0	−3.65	−1.79	1.86

Supplementary Table 23: Optimized and filtered representative structures of $[Cu_3Zn_4](Cp^*)_5$ complexes obtained with PBE+TS/light-tier1 level and light SCF parameters. The label and relative total energy (ΔE_{tot}) is depicted above each structure.

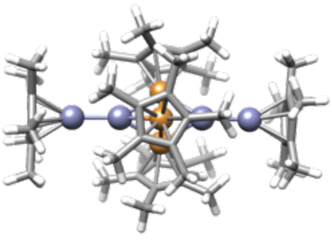	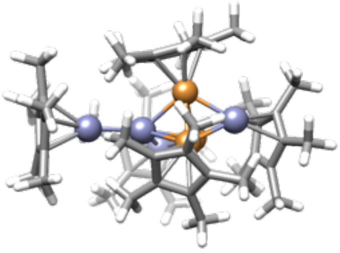	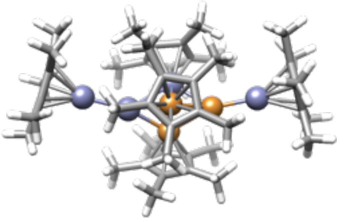
0 meV	599 meV	871 meV
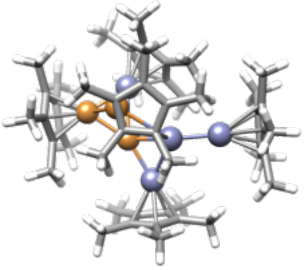	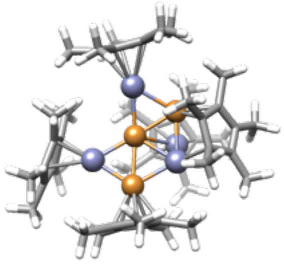	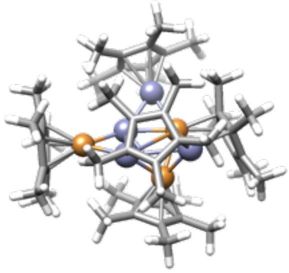
1.15 eV	1.18 eV	1.20 eV
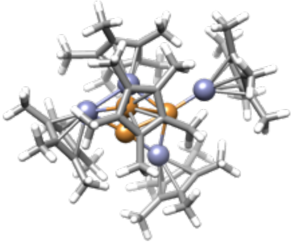	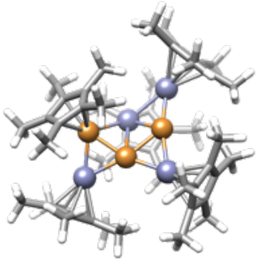	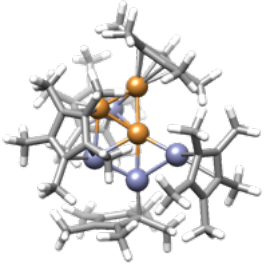
1.28 eV	1.33 eV	1.33 meV
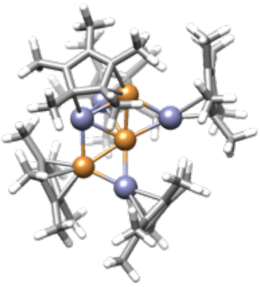	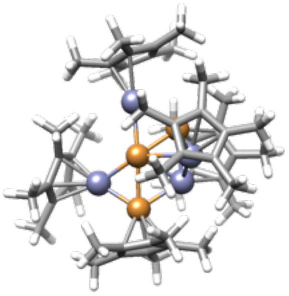	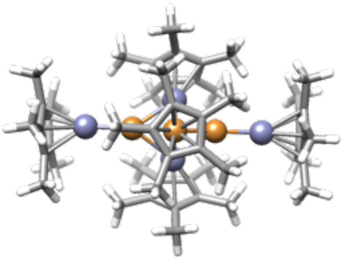
1.33 eV	1.36 eV	1.36 eV

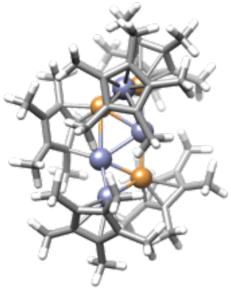

1.49 eV

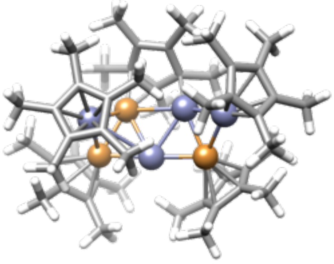

1.58 eV

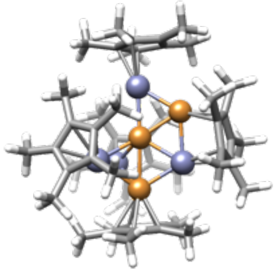

1.59 eV

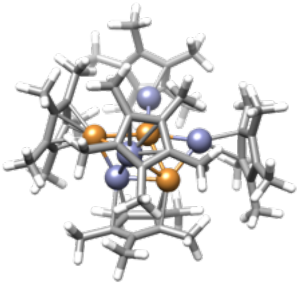

1.71 eV

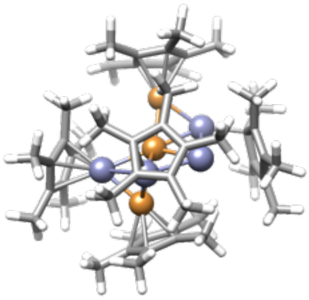

1.72 eV

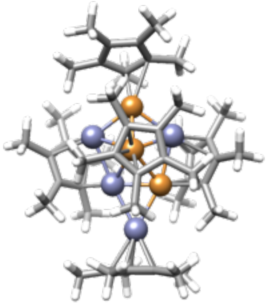

1.74 eV

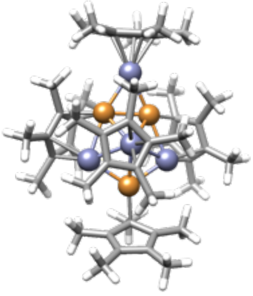

1.84 eV

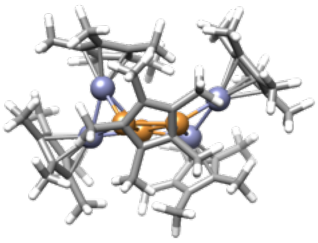

1.87 eV

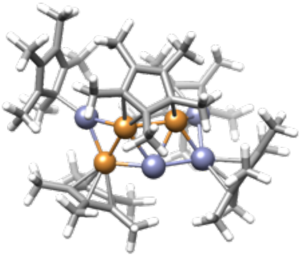

1.88 eV

2.7.3 $[Cu_5Zn_5](Cp^*)_6(CO_2)_2$ Complexes

Supplementary Table 24: Energetic and electronic properties for $[Cu_5Zn_5](Cp^*)_6(CO_2)_2$ complexes obtained with PBE/light-tier1 level and light SCF parameters: configuration number (i), relative total energy (ΔE_{tot}), total magnetic moment (m_{tot}), HOMO energy (ε_H), LUMO energy (ε_L), and LUMO-HOMO energy gap (E_g).

i	ΔE_{tot} (meV)	m_{tot} (μ_B)	ε_H (eV)	ε_L (eV)	E_g (eV)
239	5875	1	−3.73	−3.56	0.17

Continued on next page

Supplementary Table 24: Energetic and electronic properties for $[Cu_5Zn_5](Cp^*)_6(CO_2)_2$ complexes obtained with PBE/light-tier1 level and light SCF parameters: configuration number (i), relative total energy (ΔE_{tot}), total magnetic moment (m_{tot}), HOMO energy (ε_H), LUMO energy (ε_L), and LUMO-HOMO energy gap (E_g).

i	ΔE_{tot} (meV)	m_{tot} (μ_B)	ε_H (eV)	ε_L (eV)	E_g (eV)
169	5758	1	−3.54	−3.37	0.17
197	5613	1	−3.62	−3.19	0.43
95	5559	1	−3.96	−3.71	0.26
62	5538	1	−3.81	−3.49	0.32
204	5022	1	−3.78	−2.83	0.94
245	4896	1	−3.30	−2.94	0.36
210	4893	1	−3.42	−3.19	0.23
49	4766	1	−3.64	−3.41	0.24
58	4713	1	−3.43	−3.26	0.18
83	4527	1	−3.24	−3.06	0.17
238	4411	1	−3.54	−3.38	0.15
167	4354	1	−3.18	−2.97	0.21
99	4333	1	−3.33	−2.95	0.38
143	4313	1	−3.84	−3.38	0.46
18	4247	1	−3.68	−3.53	0.15
94	4247	1	−3.46	−2.95	0.50
254	4194	1	−2.92	−2.62	0.30
205	4181	1	−3.22	−2.98	0.24
319	4045	1	−3.54	−3.36	0.18
76	3999	1	−3.79	−3.62	0.17
36	3960	1	−3.24	−2.94	0.30
221	3933	1	−3.22	−3.03	0.18

Continued on next page

Supplementary Table 24: Energetic and electronic properties for $[Cu_5Zn_5](Cp^*)_6(CO_2)_2$ complexes obtained with PBE/light-tier1 level and light SCF parameters: configuration number (i), relative total energy (ΔE_{tot}), total magnetic moment (m_{tot}), HOMO energy (ε_H), LUMO energy (ε_L), and LUMO-HOMO energy gap (E_g).

i	ΔE_{tot} (meV)	m_{tot} (μ_B)	ε_H (eV)	ε_L (eV)	E_g (eV)
158	3855	1	−3.98	−3.48	0.50
10	3816	1	−3.57	−2.76	0.81
257	3762	1	−3.55	−3.37	0.18
175	3733	1	−3.69	−3.51	0.19
288	3722	1	−3.90	−3.70	0.20
79	3671	1	−3.55	−3.31	0.24
53	3541	1	−3.68	−3.57	0.11
55	3540	1	−3.82	−3.59	0.23
136	3531	1	−3.53	−2.66	0.86
73	3528	1	−3.76	−2.69	1.07
181	3502	1	−3.44	−3.21	0.23
163	3492	1	−4.04	−3.58	0.46
17	3441	1	−3.90	−3.67	0.23
41	3428	1	−3.32	−3.08	0.24
35	3412	1	−3.68	−3.51	0.17
164	3406	1	−3.52	−3.34	0.19
77	3402	1	−3.78	−3.57	0.22
51	3371	1	−3.21	−2.86	0.35
86	3370	1	−3.61	−3.44	0.17
9	3289	1	−3.69	−3.52	0.18
328	3265	1	−3.77	−3.61	0.15
231	3249	1	−3.99	−3.77	0.22

Continued on next page

Supplementary Table 24: Energetic and electronic properties for $[Cu_5Zn_5](Cp^*)_6(CO_2)_2$ complexes obtained with PBE/light-tier1 level and light SCF parameters: configuration number (i), relative total energy (ΔE_{tot}), total magnetic moment (m_{tot}), HOMO energy (ε_H), LUMO energy (ε_L), and LUMO-HOMO energy gap (E_g).

i	ΔE_{tot} (meV)	m_{tot} (μ_B)	ε_H (eV)	ε_L (eV)	E_g (eV)
148	3233	1	−2.97	−2.64	0.33
139	3214	1	−3.87	−3.78	0.09
177	3208	1	−3.30	−3.09	0.21
179	3172	1	−3.60	−3.46	0.13
187	3165	1	−3.15	−2.89	0.26
19	3158	1	−3.92	−3.61	0.31
124	3156	1	−3.75	−3.57	0.19
183	3156	1	−3.32	−3.09	0.23
322	3149	1	−3.40	−3.00	0.41
91	3147	1	−3.19	−2.91	0.29
131	3123	1	−3.14	−2.95	0.20
105	3121	1	−3.43	−3.25	0.17
279	3100	1	−3.71	−3.47	0.24
5	3097	1	−3.16	−2.94	0.22
63	3078	1	−4.10	−3.44	0.66
286	3068	1	−3.88	−3.60	0.28
11	3048	1	−4.10	−3.32	0.78
60	3016	1	−3.85	−3.58	0.27
217	3012	1	−3.61	−3.44	0.18
13	3002	1	−4.11	−3.60	0.50
33	2975	1	−4.03	−3.70	0.33
118	2954	1	−3.30	−3.14	0.16

Continued on next page

Supplementary Table 24: Energetic and electronic properties for $[Cu_5Zn_5](Cp^*)_6(CO_2)_2$ complexes obtained with PBE/light-tier1 level and light SCF parameters: configuration number (i), relative total energy (ΔE_{tot}), total magnetic moment (m_{tot}), HOMO energy (ε_H), LUMO energy (ε_L), and LUMO-HOMO energy gap (E_g).

i	ΔE_{tot} (meV)	m_{tot} (μ_B)	ε_H (eV)	ε_L (eV)	E_g (eV)
107	2921	1	−3.84	−3.53	0.31
132	2906	1	−3.85	−3.42	0.43
285	2888	1	−3.91	−3.52	0.39
273	2886	1	−3.74	−3.63	0.11
23	2862	1	−4.03	−3.50	0.54
291	2860	1	−3.58	−3.41	0.17
228	2849	1	−3.67	−3.44	0.23
250	2843	1	−3.89	−3.52	0.37
39	2836	1	−3.88	−3.60	0.28
123	2830	1	−3.78	−3.49	0.29
216	2823	1	−3.46	−3.28	0.19
266	2786	1	−4.05	−3.53	0.52
274	2784	1	−3.77	−3.60	0.18
137	2773	1	−4.02	−3.65	0.37
247	2747	1	−3.99	−3.62	0.37
295	2746	1	−3.29	−3.12	0.17
185	2737	1	−3.74	−3.59	0.15
2	2736	1	−3.55	−3.30	0.25
71	2725	1	−4.06	−3.53	0.53
256	2724	1	−4.00	−3.57	0.43
81	2718	1	−3.88	−3.52	0.36
100	2705	1	−3.76	−3.62	0.14

Continued on next page

Supplementary Table 24: Energetic and electronic properties for $[Cu_5Zn_5](Cp^*)_6(CO_2)_2$ complexes obtained with PBE/light-tier1 level and light SCF parameters: configuration number (i), relative total energy (ΔE_{tot}), total magnetic moment (m_{tot}), HOMO energy (ε_H), LUMO energy (ε_L), and LUMO-HOMO energy gap (E_g).

i	ΔE_{tot} (meV)	m_{tot} (μ_B)	ε_H (eV)	ε_L (eV)	E_g (eV)
261	2687	1	−3.83	−3.50	0.33
130	2678	1	−3.95	−3.58	0.38
154	2672	1	−3.89	−3.66	0.23
26	2648	1	−4.09	−3.63	0.46
89	2640	1	−4.00	−3.80	0.20
37	2635	1	−3.58	−3.40	0.18
57	2621	1	−3.48	−3.22	0.26
156	2598	1	−3.96	−3.58	0.38
311	2594	1	−4.26	−3.93	0.32
193	2585	1	−3.74	−3.58	0.16
199	2572	1	−3.60	−3.44	0.16
68	2572	1	−3.80	−3.53	0.28
292	2563	1	−3.73	−3.49	0.24
173	2554	1	−3.35	−3.14	0.21
202	2526	1	−3.82	−3.63	0.19
32	2515	1	−3.82	−3.64	0.18
88	2512	1	−3.73	−3.61	0.12
186	2483	1	−3.90	−3.54	0.36
249	2426	1	−3.98	−3.67	0.31
108	2411	1	−3.89	−3.51	0.38
16	2395	1	−3.84	−3.60	0.24
182	2369	1	−3.89	−3.62	0.28

Continued on next page

Supplementary Table 24: Energetic and electronic properties for $[Cu_5Zn_5](Cp^*)_6(CO_2)_2$ complexes obtained with PBE/light-tier1 level and light SCF parameters: configuration number (i), relative total energy (ΔE_{tot}), total magnetic moment (m_{tot}), HOMO energy (ε_H), LUMO energy (ε_L), and LUMO-HOMO energy gap (E_g).

i	ΔE_{tot} (meV)	m_{tot} (μ_B)	ε_H (eV)	ε_L (eV)	E_g (eV)
7	2368	1	−3.80	−3.62	0.18
277	2363	1	−3.67	−3.53	0.14
224	2361	1	−3.91	−3.76	0.15
145	2343	1	−3.85	−3.52	0.34
3	2305	1	−3.72	−3.48	0.25
90	2302	1	−3.86	−3.52	0.34
308	2279	1	−3.99	−3.82	0.16
1	2275	1	−3.64	−3.42	0.22
114	2273	1	−3.90	−3.73	0.17
72	2273	1	−3.64	−3.48	0.16
78	2266	1	−3.46	−3.25	0.21
241	2198	1	−3.86	−3.67	0.19
125	2188	1	−3.42	−3.17	0.26
152	2183	1	−3.77	−3.61	0.17
248	2183	1	−3.92	−3.66	0.25
117	2175	1	−3.50	−3.34	0.16
44	2168	1	−3.78	−3.49	0.29
116	2152	1	−3.79	−3.71	0.08
43	2148	1	−3.71	−3.55	0.16
326	2142	1	−3.38	−3.16	0.22
268	2125	1	−3.88	−3.64	0.23
281	2124	1	−3.78	−3.34	0.44

Continued on next page

Supplementary Table 24: Energetic and electronic properties for $[Cu_5Zn_5](Cp^*)_6(CO_2)_2$ complexes obtained with PBE/light-tier1 level and light SCF parameters: configuration number (i), relative total energy (ΔE_{tot}), total magnetic moment (m_{tot}), HOMO energy (ε_H), LUMO energy (ε_L), and LUMO-HOMO energy gap (E_g).

i	ΔE_{tot} (meV)	m_{tot} (μ_B)	ε_H (eV)	ε_L (eV)	E_g (eV)
20	2105	1	−3.55	−3.36	0.19
174	2066	1	−3.57	−3.37	0.20
201	2063	1	−3.57	−3.43	0.14
42	2050	1	−3.53	−3.32	0.21
129	2035	1	−3.43	−3.25	0.18
155	2033	1	−3.99	−3.79	0.20
121	2025	1	−3.56	−3.39	0.17
142	2010	1	−4.07	−3.62	0.44
87	2010	1	−3.62	−3.49	0.13
127	2006	1	−3.56	−3.38	0.18
309	1999	1	−4.24	−4.08	0.16
258	1988	1	−3.68	−3.53	0.15
69	1981	1	−3.31	−3.12	0.19
162	1976	1	−3.49	−3.33	0.17
294	1973	1	−3.48	−3.33	0.16
212	1966	1	−3.19	−2.93	0.25
318	1965	1	−3.68	−3.47	0.21
312	1956	1	−4.29	−4.11	0.18
101	1937	1	−3.48	−3.29	0.18
209	1929	1	−3.85	−3.69	0.16
150	1907	1	−3.92	−3.70	0.22
240	1889	1	−3.37	−3.17	0.21

Continued on next page

Supplementary Table 24: Energetic and electronic properties for $[Cu_5Zn_5](Cp^*)_6(CO_2)_2$ complexes obtained with PBE/light-tier1 level and light SCF parameters: configuration number (i), relative total energy (ΔE_{tot}), total magnetic moment (m_{tot}), HOMO energy (ε_H), LUMO energy (ε_L), and LUMO-HOMO energy gap (E_g).

i	ΔE_{tot} (meV)	m_{tot} (μ_B)	ε_H (eV)	ε_L (eV)	E_g (eV)
15	1885	1	−3.26	−3.01	0.26
141	1855	1	−3.22	−3.03	0.19
298	1832	1	−3.21	−3.00	0.22
166	1828	1	−3.86	−3.69	0.17
161	1821	1	−3.48	−3.31	0.17
93	1804	1	−3.59	−3.42	0.16
138	1775	1	−3.86	−3.70	0.16
31	1763	1	−4.09	−3.84	0.25
134	1748	1	−3.62	−3.44	0.18
14	1737	1	−3.67	−3.54	0.13
56	1734	1	−3.49	−3.28	0.20
122	1727	1	−3.48	−3.31	0.17
96	1727	1	−3.20	−3.04	0.16
21	1707	1	−3.82	−3.67	0.15
195	1694	1	−3.49	−3.30	0.20
307	1691	1	−4.00	−3.74	0.26
284	1686	1	−3.60	−3.40	0.20
232	1685	1	−3.80	−3.61	0.19
74	1682	1	−3.31	−3.16	0.15
304	1674	1	−3.94	−3.58	0.36
84	1673	1	−3.56	−3.37	0.19
251	1668	1	−3.57	−3.42	0.16

Continued on next page

Supplementary Table 24: Energetic and electronic properties for $[Cu_5Zn_5](Cp^*)_6(CO_2)_2$ complexes obtained with PBE/light-tier1 level and light SCF parameters: configuration number (i), relative total energy (ΔE_{tot}), total magnetic moment (m_{tot}), HOMO energy (ε_H), LUMO energy (ε_L), and LUMO-HOMO energy gap (E_g).

i	ΔE_{tot} (meV)	m_{tot} (μ_B)	ε_H (eV)	ε_L (eV)	E_g (eV)
22	1667	1	−3.50	−3.25	0.25
176	1664	1	−3.73	−3.56	0.17
140	1662	1	−3.67	−3.52	0.15
223	1652	1	−3.56	−3.39	0.16
276	1644	1	−3.62	−3.44	0.19
252	1640	1	−3.70	−3.53	0.16
170	1637	1	−3.72	−3.58	0.14
52	1621	1	−3.35	−3.18	0.17
113	1620	1	−3.34	−3.19	0.16
267	1616	1	−3.68	−3.40	0.28
4	1616	1	−3.41	−3.25	0.17
115	1607	1	−3.54	−3.38	0.16
135	1596	1	−3.11	−2.92	0.19
208	1591	1	−3.58	−3.42	0.16
229	1588	1	−3.87	−3.65	0.23
236	1581	1	−3.32	−3.16	0.16
215	1577	1	−3.22	−3.05	0.18
259	1558	1	−3.61	−3.41	0.19
45	1515	1	−3.54	−3.38	0.16
70	1505	1	−3.48	−3.31	0.17
30	1494	1	−3.83	−3.48	0.35
317	1447	1	−4.07	−3.88	0.19

Continued on next page

Supplementary Table 24: Energetic and electronic properties for $[Cu_5Zn_5](Cp^*)_6(CO_2)_2$ complexes obtained with PBE/light-tier1 level and light SCF parameters: configuration number (i), relative total energy (ΔE_{tot}), total magnetic moment (m_{tot}), HOMO energy (ε_H), LUMO energy (ε_L), and LUMO-HOMO energy gap (E_g).

i	ΔE_{tot} (meV)	m_{tot} (μ_B)	ε_H (eV)	ε_L (eV)	E_g (eV)
6	1428	1	−3.43	−3.26	0.16
296	1423	1	−3.43	−3.27	0.16
144	1414	1	−3.37	−3.20	0.17
59	1411	1	−3.14	−2.95	0.19
153	1378	1	−3.39	−3.17	0.22
92	1370	1	−3.66	−3.48	0.19
85	1361	1	−3.33	−3.10	0.23
305	1316	1	−3.73	−3.48	0.25
168	1314	1	−3.51	−3.35	0.17
119	1296	1	−3.79	−3.63	0.17
66	1267	1	−3.41	−3.24	0.17
133	1254	1	−3.66	−3.42	0.24
29	1249	1	−3.31	−3.09	0.22
321	1225	1	−3.13	−2.76	0.37
235	1215	1	−3.39	−3.22	0.17
147	1187	1	−3.87	−3.69	0.18
171	1164	1	−3.10	−2.91	0.19
61	1147	1	−3.37	−3.19	0.18
47	1124	1	−3.15	−2.96	0.19
103	1084	1	−3.27	−3.11	0.16
316	1061	1	−3.98	−3.81	0.17
324	1025	1	−3.83	−3.46	0.37

Continued on next page

Supplementary Table 24: Energetic and electronic properties for $[Cu_5Zn_5](Cp^*)_6(CO_2)_2$ complexes obtained with PBE/light-tier1 level and light SCF parameters: configuration number (i), relative total energy (ΔE_{tot}), total magnetic moment (m_{tot}), HOMO energy (ε_H), LUMO energy (ε_L), and LUMO-HOMO energy gap (E_g).

i	ΔE_{tot} (meV)	m_{tot} (μ_B)	ε_H (eV)	ε_L (eV)	E_g (eV)
218	1018	1	−3.63	−3.44	0.19
314	984	1	−3.85	−3.69	0.16
271	944	1	−3.69	−3.43	0.26
38	914	1	−3.25	−3.03	0.22
315	914	1	−3.50	−3.33	0.17
310	778	1	−3.84	−3.67	0.17
102	720	1	−3.42	−3.25	0.17
323	607	1	−3.19	−3.03	0.16
320	575	1	−3.17	−3.01	0.16
325	0	1	−3.76	−3.58	0.18

Supplementary Table 25: Optimized and filtered representative structures of $[Cu_5Zn_5](Cp^*)_6(CO_2)_2$ complexes obtained with PBE+TS/light-tier1 level and light SCF parameters. The relative total energy (ΔE_{tot}) is depicted above each structure.

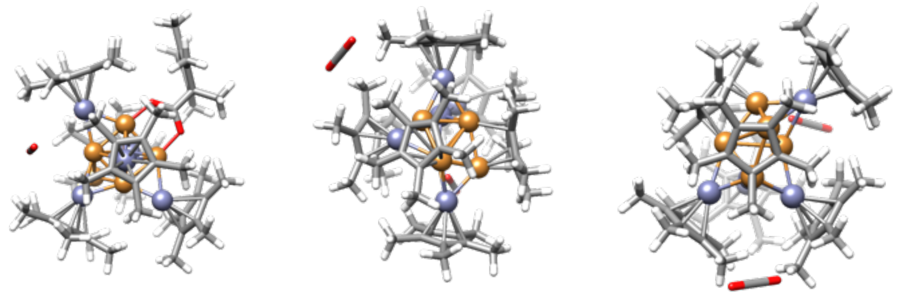

0 meV | 607 meV | 720 meV

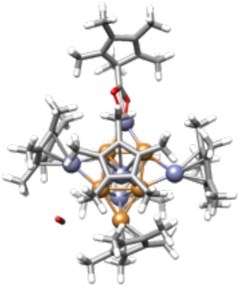

1.06 eV

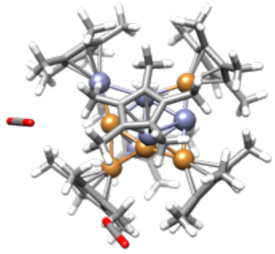

1.12 eV

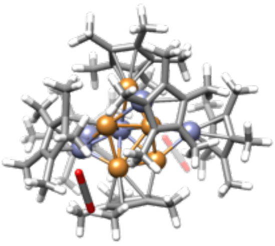

1.15 eV

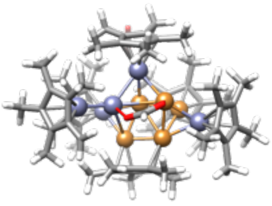

1.22 eV

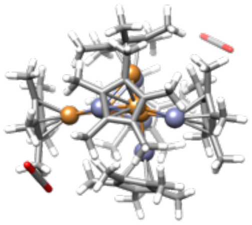

1.41 eV

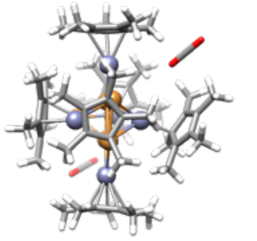

1.64 eV

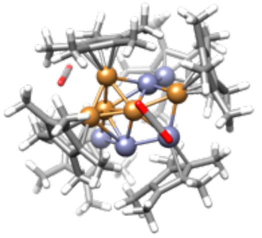

1.65 eV

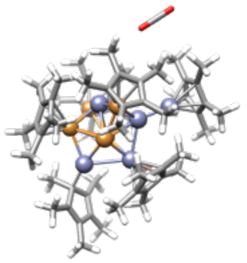

1.67 eV

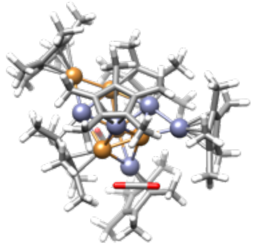

1.71 eV

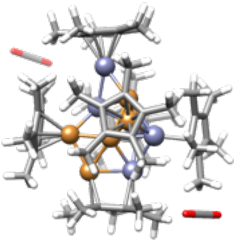

1.74 eV

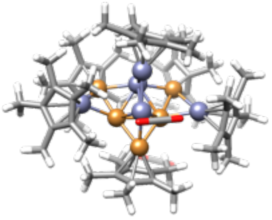

1.93 eV

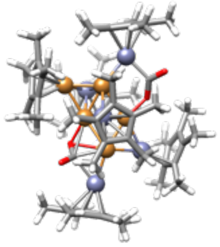

1.96 eV

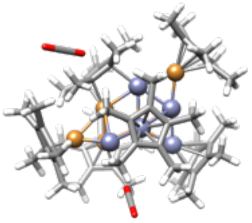

1.99 eV

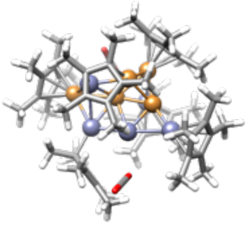

2.02 eV

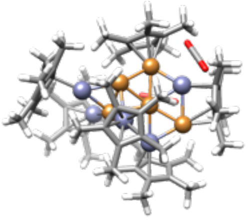

2.28 eV

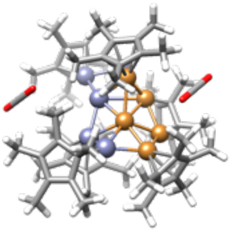
2.56 eV

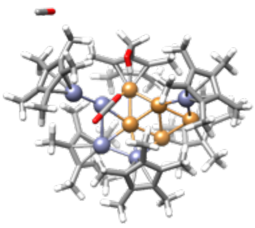
3.26 eV

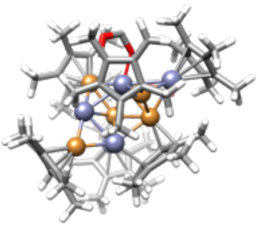
4.00 eV

2.7.4 $[Cu_6Zn_5](Cp^*)_5(Mes)H_3$ Complexes

Supplementary Table 26: Energetic and electronic properties for $[Cu_6Zn_5](Cp^*)_5(Mes)H_3$ complexes obtained with PBE+TS/light-tier1 level and light SCF parameters: configuration number (i), relative total energy (ΔE_{tot}), total magnetic moment (m_{tot}), HOMO energy (ε_H), LUMO energy (ε_L), and LUMO-HOMO energy gap (E_g).

i	ΔE_{tot} (meV)	m_{tot} (μ_B)	ε_H (eV)	ε_L (eV)	E_g (eV)
173	6593	1	−4.03	−3.79	0.24
64	6272	1	−3.70	−2.67	1.03
197	5838	1	−3.84	−3.03	0.81
123	5128	1	−3.67	−3.48	0.19
28	5101	1	−3.76	−3.60	0.16
133	5032	1	−3.78	−3.43	0.35
273	4909	1	−3.76	−3.59	0.17
266	4796	1	−3.83	−2.84	0.99
70	4697	1	−3.82	−3.41	0.41
106	4642	1	−4.04	−3.61	0.43
94	4545	1	−4.04	−3.71	0.33
275	4406	1	−3.47	−3.25	0.22
57	4342	1	−3.75	−3.59	0.16
170	4316	1	−4.07	−3.30	0.78
323	4130	1	−3.79	−3.03	0.77

Continued on next page

Supplementary Table 26: Energetic and electronic properties for $[Cu_6Zn_5](Cp^*)_5(Mes)H_3$ complexes obtained with PBE+TS/light-tier1 level and light SCF parameters: configuration number (i), relative total energy (ΔE_{tot}), total magnetic moment (m_{tot}), HOMO energy (ε_H), LUMO energy (ε_L), and LUMO-HOMO energy gap (E_g).

i	ΔE_{tot} (meV)	m_{tot} (μ_B)	ε_H (eV)	ε_L (eV)	E_g (eV)
185	4055	1	−3.78	−3.38	0.40
8	4006	1	−4.12	−3.73	0.39
37	3997	1	−3.80	−3.36	0.44
227	3959	1	−3.74	−3.55	0.19
224	3884	1	−4.06	−3.18	0.88
81	3798	1	−4.06	−3.20	0.86
233	3738	1	−3.18	−2.99	0.19
60	3733	1	−4.17	−3.51	0.66
240	3728	1	−3.70	−3.60	0.10
12	3688	1	−3.71	−2.74	0.97
255	3579	1	−4.02	−3.17	0.85
286	3542	1	−3.53	−3.35	0.18
281	3493	1	−3.19	−2.72	0.47
189	3447	1	−3.48	−3.26	0.22
53	3442	1	−3.63	−3.41	0.21
33	3420	1	−3.78	−3.30	0.48
54	3407	1	−3.50	−3.34	0.15
61	3357	1	−4.26	−3.52	0.74
102	3327	1	−3.66	−3.43	0.22
159	3304	1	−3.83	−3.67	0.16
195	3297	1	−4.11	−3.48	0.63
282	3288	1	−3.71	−2.93	0.78

Continued on next page

Supplementary Table 26: Energetic and electronic properties for $[Cu_6Zn_5](Cp^*)_5(Mes)H_3$ complexes obtained with PBE+TS/light-tier1 level and light SCF parameters: configuration number (i), relative total energy (ΔE_{tot}), total magnetic moment (m_{tot}), HOMO energy (ε_H), LUMO energy (ε_L), and LUMO-HOMO energy gap (E_g).

i	ΔE_{tot} (meV)	m_{tot} (μ_B)	ε_H (eV)	ε_L (eV)	E_g (eV)
95	3256	1	−3.38	−3.22	0.16
174	3253	1	−3.60	−3.43	0.16
261	3247	1	−3.49	−3.25	0.24
10	3202	1	−4.12	−4.00	0.13
211	3202	1	−4.01	−2.95	1.06
288	3184	1	−3.74	−3.31	0.43
84	3159	1	−4.23	−3.68	0.55
268	3122	1	−3.47	−3.30	0.17
69	3115	1	−3.61	−3.41	0.20
30	3107	1	−3.65	−3.44	0.21
252	3096	1	−3.68	−3.46	0.22
46	3068	1	−3.55	−3.40	0.15
72	3063	1	−3.55	−3.33	0.22
11	3049	1	−3.79	−3.54	0.25
75	3038	1	−3.38	−3.14	0.23
14	3004	1	−4.32	−3.57	0.76
210	2997	1	−4.32	−4.23	0.09
278	2994	1	−3.21	−3.07	0.14
239	2978	1	−3.19	−2.57	0.62
76	2973	1	−3.71	−3.55	0.16
65	2949	1	−3.92	−3.47	0.45
142	2928	1	−4.08	−3.41	0.67

Continued on next page

Supplementary Table 26: Energetic and electronic properties for $[Cu_6Zn_5](Cp^*)_5(Mes)H_3$ complexes obtained with PBE+TS/light-tier1 level and light SCF parameters: configuration number (i), relative total energy (ΔE_{tot}), total magnetic moment (m_{tot}), HOMO energy (ε_H), LUMO energy (ε_L), and LUMO-HOMO energy gap (E_g).

i	ΔE_{tot} (meV)	m_{tot} (μ_B)	ε_H (eV)	ε_L (eV)	E_g (eV)
35	2927	1	−4.24	−3.59	0.65
73	2921	1	−4.31	−3.59	0.72
263	2902	1	−3.38	−3.20	0.17
176	2901	1	−4.10	−3.69	0.40
232	2850	1	−3.57	−3.39	0.17
154	2847	1	−3.62	−3.43	0.19
265	2825	1	−3.54	−3.40	0.14
1	2790	1	−3.50	−3.25	0.26
34	2784	1	−4.01	−3.36	0.65
203	2782	1	−3.99	−3.85	0.14
101	2707	1	−3.46	−3.27	0.19
4	2678	1	−3.91	−3.70	0.21
161	2662	1	−4.21	−3.54	0.67
112	2655	1	−3.40	−3.18	0.22
115	2649	1	−4.09	−3.95	0.15
157	2620	1	−3.70	−3.53	0.18
49	2618	1	−4.20	−4.08	0.11
289	2612	1	−4.07	−3.30	0.77
55	2601	1	−3.93	−3.79	0.14
71	2585	1	−4.08	−3.62	0.47
196	2521	1	−4.02	−3.64	0.38
145	2517	1	−3.57	−3.41	0.16

Continued on next page

Supplementary Table 26: Energetic and electronic properties for $[Cu_6Zn_5](Cp^*)_5(Mes)H_3$ complexes obtained with PBE+TS/light-tier1 level and light SCF parameters: configuration number (i), relative total energy (ΔE_{tot}), total magnetic moment (m_{tot}), HOMO energy (ε_H), LUMO energy (ε_L), and LUMO-HOMO energy gap (E_g).

i	ΔE_{tot} (meV)	m_{tot} (μ_B)	ε_H (eV)	ε_L (eV)	E_g (eV)
56	2508	1	−4.13	−3.72	0.42
132	2468	1	−3.51	−3.31	0.20
229	2444	1	−3.44	−3.28	0.16
241	2439	1	−3.99	−3.58	0.41
146	2437	1	−3.45	−3.24	0.22
87	2420	1	−3.99	−3.58	0.41
284	2419	1	−4.09	−3.38	0.71
13	2411	1	−3.94	−3.43	0.51
158	2375	1	−4.22	−3.56	0.66
235	2369	1	−3.58	−3.44	0.13
175	2350	1	−3.35	−3.18	0.17
96	2337	1	−3.42	−3.24	0.18
162	2331	1	−4.06	−3.69	0.37
315	2323	1	−4.12	−3.96	0.15
26	2320	1	−4.14	−3.36	0.77
82	2310	1	−3.08	−2.87	0.21
228	2305	1	−3.05	−2.83	0.21
164	2292	1	−3.94	−3.59	0.36
137	2288	1	−3.27	−3.07	0.21
198	2287	1	−3.80	−3.61	0.19
39	2272	1	−3.98	−3.52	0.45
110	2271	1	−4.18	−4.07	0.11

Continued on next page

Supplementary Table 26: Energetic and electronic properties for $[Cu_6Zn_5](Cp^*)_5(Mes)H_3$ complexes obtained with PBE+TS/light-tier1 level and light SCF parameters: configuration number (i), relative total energy (ΔE_{tot}), total magnetic moment (m_{tot}), HOMO energy (ε_H), LUMO energy (ε_L), and LUMO-HOMO energy gap (E_g).

i	ΔE_{tot} (meV)	m_{tot} (μ_B)	ε_H (eV)	ε_L (eV)	E_g (eV)
251	2271	1	−3.84	−3.58	0.26
297	2271	1	−4.19	−3.49	0.70
194	2253	1	−4.09	−3.56	0.52
168	2246	1	−3.17	−2.93	0.24
287	2243	1	−4.10	−3.97	0.13
222	2230	1	−3.61	−3.47	0.14
7	2226	1	−3.79	−3.66	0.13
238	2211	1	−3.65	−3.36	0.29
23	2204	1	−3.78	−3.60	0.17
264	2198	1	−3.49	−3.33	0.17
83	2185	1	−3.52	−3.33	0.19
294	2180	1	−3.63	−3.22	0.41
165	2172	1	−3.59	−3.41	0.17
44	2129	1	−3.55	−3.36	0.19
277	2125	1	−3.33	−3.13	0.19
242	2107	1	−3.65	−3.46	0.19
256	2104	1	−3.84	−3.57	0.28
271	2096	1	−3.84	−3.69	0.16
249	2095	1	−3.67	−3.47	0.20
184	2094	1	−3.84	−3.64	0.20
121	2078	1	−4.10	−3.94	0.16
91	2069	1	−3.32	−2.89	0.43

Continued on next page

Supplementary Table 26: Energetic and electronic properties for $[Cu_6Zn_5](Cp^*)_5(Mes)H_3$ complexes obtained with PBE+TS/light-tier1 level and light SCF parameters: configuration number (i), relative total energy (ΔE_{tot}), total magnetic moment (m_{tot}), HOMO energy (ε_H), LUMO energy (ε_L), and LUMO-HOMO energy gap (E_g).

i	ΔE_{tot} (meV)	m_{tot} (μ_B)	ε_H (eV)	ε_L (eV)	E_g (eV)
181	2064	1	−4.15	−3.73	0.42
150	2045	1	−3.60	−3.43	0.17
136	2032	1	−3.83	−3.69	0.14
190	2024	1	−4.09	−3.92	0.17
59	2006	1	−3.93	−3.51	0.42
45	2002	1	−4.09	−3.92	0.16
107	1981	1	−3.59	−3.37	0.21
308	1960	1	−3.70	−3.50	0.20
250	1955	1	−3.60	−3.47	0.13
85	1939	1	−4.02	−3.84	0.18
15	1927	1	−4.20	−4.02	0.18
2	1911	1	−3.41	−3.26	0.15
216	1910	1	−3.27	−3.04	0.24
24	1906	1	−3.93	−3.79	0.14
316	1905	1	−4.04	−3.80	0.24
40	1893	1	−3.58	−3.41	0.16
199	1886	1	−3.49	−3.32	0.17
32	1882	1	−3.83	−3.68	0.14
319	1873	1	−3.58	−3.40	0.18
306	1828	1	−3.95	−3.76	0.19
225	1824	1	−3.90	−3.74	0.16
126	1821	1	−3.69	−3.39	0.30

Continued on next page

Supplementary Table 26: Energetic and electronic properties for $[Cu_6Zn_5](Cp^*)_5(Mes)H_3$ complexes obtained with PBE+TS/light-tier1 level and light SCF parameters: configuration number (i), relative total energy (ΔE_{tot}), total magnetic moment (m_{tot}), HOMO energy (ε_H), LUMO energy (ε_L), and LUMO-HOMO energy gap (E_g).

i	ΔE_{tot} (meV)	m_{tot} (μ_B)	ε_H (eV)	ε_L (eV)	E_g (eV)
52	1816	1	−3.72	−3.56	0.16
50	1793	1	−4.09	−3.71	0.37
214	1787	1	−4.04	−3.82	0.22
113	1785	1	−3.79	−3.62	0.17
248	1780	1	−3.62	−3.43	0.19
131	1776	1	−3.77	−3.59	0.17
98	1766	1	−3.96	−3.80	0.16
9	1763	1	−3.89	−3.49	0.40
322	1749	1	−3.60	−3.44	0.16
179	1738	1	−3.87	−3.70	0.17
231	1729	1	−3.87	−3.70	0.17
215	1721	1	−3.80	−3.62	0.18
269	1711	1	−3.92	−3.77	0.15
169	1705	1	−3.57	−3.39	0.18
144	1702	1	−3.91	−3.79	0.12
17	1682	1	−3.87	−3.68	0.19
314	1672	1	−4.09	−3.89	0.20
213	1650	1	−3.64	−3.41	0.22
149	1641	1	−3.72	−3.57	0.15
80	1630	1	−4.01	−3.86	0.15
20	1610	1	−3.50	−3.34	0.17
67	1603	1	−3.79	−3.62	0.17

Continued on next page

Supplementary Table 26: Energetic and electronic properties for $[Cu_6Zn_5](Cp^*)_5(Mes)H_3$ complexes obtained with PBE+TS/light-tier1 level and light SCF parameters: configuration number (i), relative total energy (ΔE_{tot}), total magnetic moment (m_{tot}), HOMO energy (ε_H), LUMO energy (ε_L), and LUMO-HOMO energy gap (E_g).

i	ΔE_{tot} (meV)	m_{tot} (μ_B)	ε_H (eV)	ε_L (eV)	E_g (eV)
267	1600	1	−3.22	−2.84	0.38
312	1594	1	−3.81	−3.67	0.14
62	1578	1	−3.66	−3.48	0.18
118	1536	1	−4.02	−3.64	0.38
167	1535	1	−3.61	−3.45	0.16
212	1527	1	−3.75	−3.58	0.17
193	1524	1	−3.71	−3.57	0.14
29	1523	1	−3.50	−3.01	0.49
204	1520	1	−3.97	−3.82	0.15
105	1515	1	−3.97	−3.80	0.17
38	1506	1	−3.54	−3.33	0.21
172	1498	1	−3.54	−3.33	0.21
201	1492	1	−3.97	−3.67	0.30
279	1490	1	−3.74	−3.55	0.18
262	1481	1	−3.93	−3.71	0.22
237	1469	1	−3.55	−3.40	0.16
317	1459	1	−4.17	−4.01	0.16
254	1450	1	−3.74	−3.59	0.15
313	1449	1	−3.52	−3.34	0.18
78	1413	1	−3.56	−3.37	0.19
42	1409	1	−3.70	−3.53	0.17
48	1399	1	−3.63	−3.45	0.18

Continued on next page

Supplementary Table 26: Energetic and electronic properties for $[Cu_6Zn_5](Cp^*)_5(Mes)H_3$ complexes obtained with PBE+TS/light-tier1 level and light SCF parameters: configuration number (i), relative total energy (ΔE_{tot}), total magnetic moment (m_{tot}), HOMO energy (ε_H), LUMO energy (ε_L), and LUMO-HOMO energy gap (E_g).

i	ΔE_{tot} (meV)	m_{tot} (μ_B)	ε_H (eV)	ε_L (eV)	E_g (eV)
299	1366	1	−3.69	−3.55	0.14
311	1315	1	−3.80	−3.62	0.17
200	1307	1	−3.85	−3.64	0.21
22	1272	1	−3.80	−3.65	0.16
134	1256	1	−3.55	−3.38	0.17
292	1249	1	−3.76	−3.63	0.13
274	1246	1	−3.59	−3.44	0.15
187	1237	1	−3.55	−3.39	0.16
74	1224	1	−3.69	−3.54	0.16
58	1223	1	−3.51	−3.30	0.20
3	1220	1	−3.93	−3.79	0.14
183	1204	1	−4.23	−4.13	0.10
298	1197	1	−3.13	−2.99	0.14
309	1160	1	−3.70	−3.53	0.17
206	1149	1	−3.38	−3.14	0.25
258	1122	1	−3.67	−3.52	0.15
109	1113	1	−3.66	−3.49	0.16
236	1096	1	−3.44	−3.23	0.21
93	1092	1	−3.76	−3.60	0.16
321	1089	1	−3.60	−3.44	0.17
6	1080	1	−3.71	−3.46	0.24
303	1059	1	−3.82	−3.68	0.14

Continued on next page

Supplementary Table 26: Energetic and electronic properties for $[Cu_6Zn_5](Cp^*)_5(Mes)H_3$ complexes obtained with PBE+TS/light-tier1 level and light SCF parameters: configuration number (i), relative total energy (ΔE_{tot}), total magnetic moment (m_{tot}), HOMO energy (ε_H), LUMO energy (ε_L), and LUMO-HOMO energy gap (E_g).

i	ΔE_{tot} (meV)	m_{tot} (μ_B)	ε_H (eV)	ε_L (eV)	E_g (eV)
122	1049	1	−3.67	−3.39	0.28
205	1026	1	−3.58	−3.40	0.18
117	1018	1	−3.96	−3.78	0.18
166	1013	1	−3.65	−3.45	0.20
304	1000	1	−3.59	−3.43	0.15
310	997	1	−4.05	−3.88	0.16
31	993	1	−3.37	−3.14	0.23
182	986	1	−3.91	−3.75	0.16
86	981	1	−3.71	−3.48	0.23
259	979	1	−3.82	−3.69	0.13
305	972	1	−3.58	−3.45	0.13
51	940	1	−3.62	−3.47	0.15
178	928	1	−3.51	−3.34	0.17
141	919	1	−3.61	−3.44	0.18
253	900	1	−3.82	−3.60	0.22
18	889	1	−3.79	−3.62	0.16
147	855	1	−3.69	−3.48	0.21
226	845	1	−3.34	−3.18	0.16
36	824	1	−3.88	−3.72	0.16
302	812	1	−3.69	−3.55	0.14
324	803	1	−3.61	−3.44	0.18
207	793	1	−3.78	−3.56	0.21

Continued on next page

Supplementary Table 26: Energetic and electronic properties for $[Cu_6Zn_5](Cp^*)_5(Mes)H_3$ complexes obtained with PBE+TS/light-tier1 level and light SCF parameters: configuration number (i), relative total energy (ΔE_{tot}), total magnetic moment (m_{tot}), HOMO energy (ε_H), LUMO energy (ε_L), and LUMO-HOMO energy gap (E_g).

i	ΔE_{tot} (meV)	m_{tot} (μ_B)	ε_H (eV)	ε_L (eV)	E_g (eV)
188	703	1	−3.64	−3.46	0.18
116	635	1	−3.70	−3.55	0.15
120	549	1	−3.36	−3.20	0.16
16	446	1	−3.64	−3.47	0.17
307	0	1	−3.50	−3.34	0.16

Continued on next page

Supplementary Table 27: Optimized and filtered representative structures of $[Cu_6Zn_5](Cp^*)_5(Mes)(H)_3$ complexes obtained with PBE+TS/light-tier1 level and light SCF parameters. The relative total energy (ΔE_{tot}) is depicted above each structure.

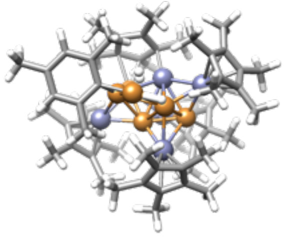

0 meV

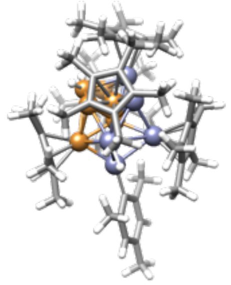

446 meV

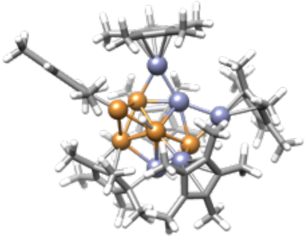

703 meV

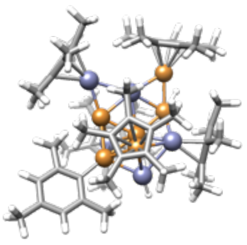

855 meV

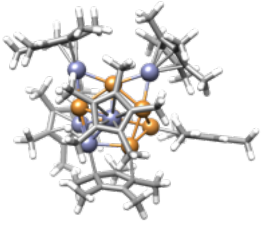

1.00 eV

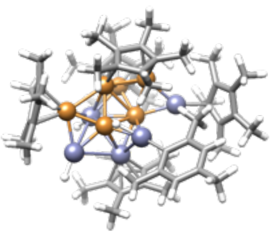

1.09 eV

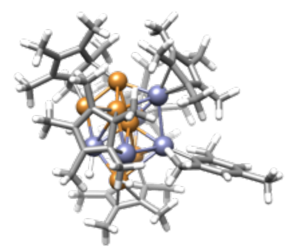

1.12 eV

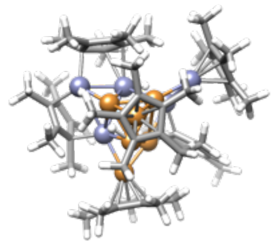

1.20 eV

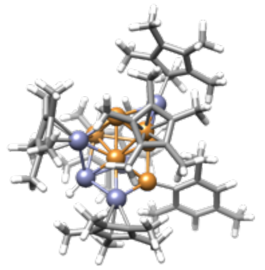

1.20 eV

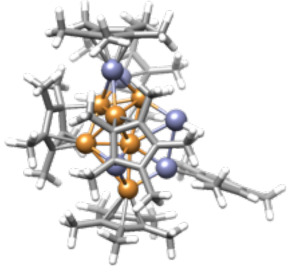

1.27 eV

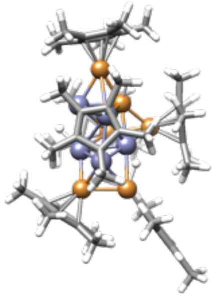

1.50 eV

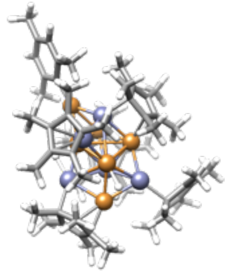

1.59 eV

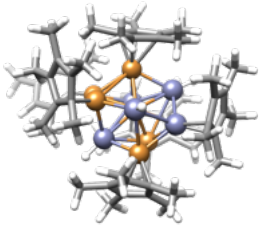

1.71 eV

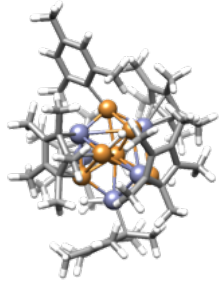

1.75 eV

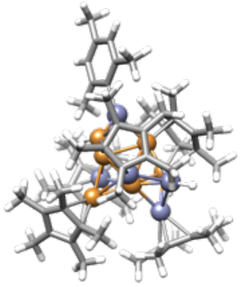

1.82 eV

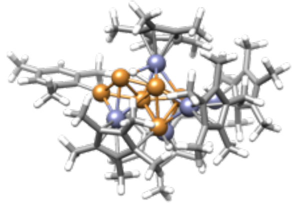

1.87 eV

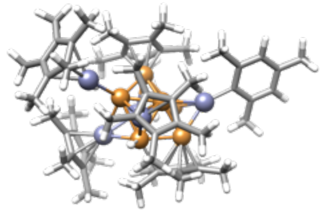

1.88 eV

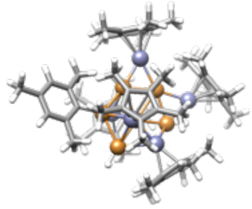

1.90 eV

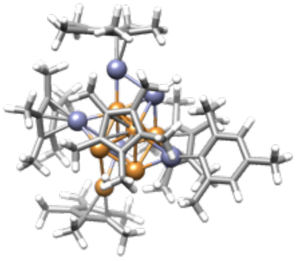

1.94 eV

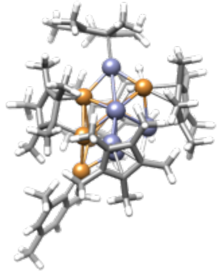

2.20 eV

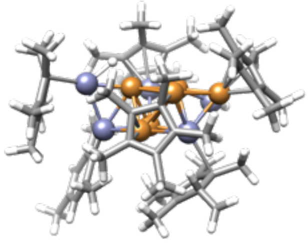

3.29 eV

2.7.5 $[Cu_8Zn_3](Cp^*)_3(Mes)_4CO_2$ Complexes

Supplementary Table 28: Energetic and electronic properties for $[Cu_8Zn_3](Cp^*)_3(Mes)_4CO_2$ complexes obtained with PBE+TS/light-tier1 level and light SCF parameters: configuration number (i), relative total energy (ΔE_{tot}), total magnetic moment (m_{tot}), HOMO energy (ε_H), LUMO energy (ε_L), and LUMO-HOMO energy gap (E_g).

i	ΔE_{tot} (meV)	m_{tot} (μ_B)	ε_H (eV)	ε_L (eV)	E_g (eV)
209	6783	1	−3.40	−3.21	0.19
78	6173	1	−4.43	−3.70	0.72
187	5880	1	−4.24	−3.71	0.53
102	5559	1	−3.80	−3.16	0.64
83	5516	1	−3.57	−3.39	0.18
282	5274	1	−4.21	−3.16	1.06
72	5188	1	−4.25	−3.47	0.78
212	4925	1	−4.01	−2.97	1.05
114	4911	1	−3.88	−3.08	0.80
111	4826	1	−3.70	−3.41	0.30
203	4824	1	−4.09	−3.37	0.73
37	4815	1	−4.30	−3.34	0.96
94	4743	1	−4.18	−3.07	1.11
267	4704	1	−3.52	−3.33	0.19
116	4680	1	−4.34	−3.44	0.90
298	4674	1	−3.78	−3.59	0.19
108	4529	1	−4.10	−3.89	0.20
86	4450	1	−4.22	−3.11	1.12
158	4432	1	−4.03	−3.21	0.81
123	4412	1	−4.21	−3.30	0.91
62	4399	1	−4.35	−3.62	0.73
23	4342	1	−4.06	−3.25	0.81

Continued on next page

Supplementary Table 28: Energetic and electronic properties for $[Cu_8Zn_3](Cp^*)_3(Mes)_4CO_2$ complexes obtained with PBE+TS/light-tier1 level and light SCF parameters: configuration number (i), relative total energy (ΔE_{tot}), total magnetic moment (m_{tot}), HOMO energy (ε_H), LUMO energy (ε_L), and LUMO-HOMO energy gap (E_g).

i	ΔE_{tot} (meV)	m_{tot} (μ_B)	ε_H (eV)	ε_L (eV)	E_g (eV)
103	4284	1	−4.04	−3.26	0.78
112	4279	1	−3.68	−3.49	0.19
41	4251	1	−4.14	−3.81	0.33
265	4212	1	−4.04	−3.79	0.25
238	4166	1	−3.93	−2.84	1.09
285	4164	1	−4.12	−3.31	0.81
317	4069	1	−3.41	−2.75	0.66
223	4043	1	−3.88	−3.71	0.17
19	4038	1	−3.96	−2.97	0.99
142	4028	1	−3.48	−3.20	0.28
313	3997	1	−4.19	−3.99	0.20
293	3993	1	−4.31	−3.69	0.62
257	3976	1	−4.34	−3.65	0.70
180	3968	1	−3.84	−3.14	0.69
100	3964	1	−4.03	−3.25	0.78
30	3956	1	−3.85	−3.04	0.81
154	3907	1	−4.02	−3.88	0.14
300	3875	1	−3.26	−3.09	0.16
125	3846	1	−4.14	−3.62	0.52
10	3811	1	−4.08	−3.23	0.85
247	3790	1	−3.76	−3.53	0.23
181	3752	1	−3.63	−3.01	0.62

Continued on next page

Supplementary Table 28: Energetic and electronic properties for $[Cu_8Zn_3](Cp^*)_3(Mes)_4CO_2$ complexes obtained with PBE+TS/light-tier1 level and light SCF parameters: configuration number (i), relative total energy (ΔE_{tot}), total magnetic moment (m_{tot}), HOMO energy (ε_H), LUMO energy (ε_L), and LUMO-HOMO energy gap (E_g).

i	ΔE_{tot} (meV)	m_{tot} (μ_B)	ε_H (eV)	ε_L (eV)	E_g (eV)
105	3738	1	−4.03	−3.22	0.81
35	3717	1	−4.07	−3.64	0.44
291	3717	1	−4.38	−3.60	0.78
61	3684	1	−4.15	−3.93	0.21
156	3681	1	−3.99	−3.12	0.87
202	3630	1	−3.31	−3.12	0.19
56	3620	1	−3.68	−3.51	0.18
214	3604	1	−4.32	−3.72	0.61
185	3589	1	−3.81	−2.99	0.82
107	3571	1	−4.05	−3.87	0.17
136	3554	1	−3.88	−3.71	0.18
13	3551	1	−3.97	−3.26	0.71
296	3519	1	−3.52	−2.96	0.56
345	3329	1	−4.14	−3.99	0.16
76	3257	1	−4.19	−4.05	0.14
64	3247	1	−3.57	−3.42	0.15
284	3237	1	−3.23	−3.06	0.17
126	3155	1	−3.61	−3.37	0.23
183	3127	1	−3.26	−2.98	0.29
8	3122	1	−3.86	−3.49	0.38
233	3086	1	−3.27	−3.08	0.19
256	3084	1	−4.21	−3.74	0.47

Continued on next page

Supplementary Table 28: Energetic and electronic properties for $[Cu_8Zn_3](Cp^*)_3(Mes)_4CO_2$ complexes obtained with PBE+TS/light-tier1 level and light SCF parameters: configuration number (i), relative total energy (ΔE_{tot}), total magnetic moment (m_{tot}), HOMO energy (ε_H), LUMO energy (ε_L), and LUMO-HOMO energy gap (E_g).

i	ΔE_{tot} (meV)	m_{tot} (μ_B)	ε_H (eV)	ε_L (eV)	E_g (eV)
272	3070	1	−4.00	−3.47	0.53
297	3052	1	−3.24	−3.09	0.14
279	2991	1	−4.12	−3.98	0.14
47	2989	1	−3.73	−3.58	0.16
26	2985	1	−3.80	−3.57	0.23
171	2982	1	−3.37	−3.23	0.14
276	2979	1	−3.52	−3.30	0.21
163	2968	1	−4.12	−3.45	0.67
22	2957	1	−3.75	−3.57	0.18
221	2915	1	−4.00	−3.66	0.33
343	2896	1	−3.71	−3.49	0.22
9	2875	1	−3.53	−3.39	0.14
338	2862	1	−3.82	−3.58	0.24
110	2857	1	−3.78	−3.62	0.16
271	2838	1	−3.98	−3.82	0.16
179	2838	1	−4.08	−3.90	0.18
3	2811	1	−3.88	−3.74	0.15
14	2784	1	−3.60	−3.42	0.18
73	2783	1	−4.09	−3.93	0.16
194	2761	1	−3.62	−3.44	0.18
63	2760	1	−3.55	−3.22	0.33
269	2732	1	−4.16	−3.98	0.18

Continued on next page

Supplementary Table 28: Energetic and electronic properties for $[Cu_8Zn_3](Cp^*)_3(Mes)_4CO_2$ complexes obtained with PBE+TS/light-tier1 level and light SCF parameters: configuration number (i), relative total energy (ΔE_{tot}), total magnetic moment (m_{tot}), HOMO energy (ε_H), LUMO energy (ε_L), and LUMO-HOMO energy gap (E_g).

i	ΔE_{tot} (meV)	m_{tot} (μ_B)	ε_H (eV)	ε_L (eV)	E_g (eV)
204	2716	1	−3.91	−3.73	0.17
259	2716	1	−3.75	−3.59	0.15
119	2683	1	−3.68	−3.51	0.17
208	2682	1	−3.62	−3.47	0.15
45	2648	1	−4.13	−3.98	0.16
231	2636	1	−4.07	−3.89	0.18
137	2607	1	−4.12	−3.91	0.21
250	2600	1	−3.41	−3.17	0.24
320	2583	1	−3.56	−3.37	0.19
168	2574	1	−3.23	−2.81	0.42
97	2565	1	−3.81	−3.64	0.17
346	2539	1	−3.46	−3.31	0.15
229	2523	1	−3.54	−3.38	0.16
248	2517	1	−3.58	−3.40	0.18
287	2495	1	−4.06	−3.86	0.20
20	2484	1	−3.82	−3.65	0.18
340	2477	1	−4.05	−3.87	0.19
243	2473	1	−3.95	−3.76	0.19
15	2472	1	−3.74	−3.55	0.19
16	2468	1	−3.89	−3.65	0.24
191	2449	1	−3.42	−3.21	0.22
252	2446	1	−3.78	−3.62	0.16

Continued on next page

Supplementary Table 28: Energetic and electronic properties for $[Cu_8Zn_3](Cp^*)_3(Mes)_4CO_2$ complexes obtained with PBE+TS/light-tier1 level and light SCF parameters: configuration number (i), relative total energy (ΔE_{tot}), total magnetic moment (m_{tot}), HOMO energy (ε_H), LUMO energy (ε_L), and LUMO-HOMO energy gap (E_g).

i	ΔE_{tot} (meV)	m_{tot} (μ_B)	ε_H (eV)	ε_L (eV)	E_g (eV)
200	2445	1	−3.97	−3.78	0.19
341	2444	1	−4.09	−3.91	0.18
319	2425	1	−4.20	−4.04	0.15
166	2422	1	−3.70	−3.54	0.16
222	2416	1	−3.35	−3.13	0.23
93	2416	1	−3.66	−3.51	0.14
133	2410	1	−3.92	−3.72	0.20
292	2402	1	−3.83	−3.66	0.17
124	2402	1	−4.14	−3.97	0.17
68	2396	1	−3.66	−3.48	0.18
24	2396	1	−4.18	−4.01	0.17
232	2389	1	−3.73	−3.56	0.16
152	2382	1	−3.50	−3.32	0.18
175	2375	1	−3.86	−3.70	0.16
323	2366	1	−3.48	−3.30	0.18
335	2354	1	−3.53	−3.38	0.15
322	2345	1	−3.61	−3.40	0.21
98	2331	1	−3.61	−3.45	0.17
264	2329	1	−3.96	−3.81	0.15
92	2321	1	−3.64	−3.43	0.21
79	2316	1	−3.89	−3.76	0.13
153	2315	1	−3.92	−3.77	0.15

Continued on next page

Supplementary Table 28: Energetic and electronic properties for $[Cu_8Zn_3](Cp^*)_3(Mes)_4CO_2$ complexes obtained with PBE+TS/light-tier1 level and light SCF parameters: configuration number (i), relative total energy (ΔE_{tot}), total magnetic moment (m_{tot}), HOMO energy (ε_H), LUMO energy (ε_L), and LUMO-HOMO energy gap (E_g).

i	ΔE_{tot} (meV)	m_{tot} (μ_B)	ε_H (eV)	ε_L (eV)	E_g (eV)
91	2309	1	−3.74	−3.57	0.16
186	2309	1	−3.51	−3.33	0.19
67	2305	1	−3.75	−3.59	0.16
281	2284	1	−3.88	−3.70	0.18
104	2256	1	−3.52	−3.37	0.15
99	2252	1	−3.96	−3.75	0.21
280	2250	1	−3.73	−3.59	0.15
197	2223	1	−3.66	−3.52	0.14
342	2221	1	−4.04	−3.85	0.19
178	2204	1	−3.74	−3.58	0.16
205	2199	1	−3.31	−3.09	0.22
289	2198	1	−3.99	−3.80	0.18
128	2191	1	−3.46	−3.30	0.16
173	2181	1	−3.82	−3.66	0.16
199	2180	1	−3.96	−3.80	0.16
329	2163	1	−3.95	−3.77	0.17
227	2146	1	−3.65	−3.38	0.26
330	2146	1	−3.96	−3.79	0.17
216	2145	1	−3.75	−3.59	0.16
315	2140	1	−3.75	−3.57	0.18
261	2139	1	−3.67	−3.53	0.14
33	2134	1	−3.82	−3.61	0.21

Continued on next page

Supplementary Table 28: Energetic and electronic properties for $[Cu_8Zn_3](Cp^*)_3(Mes)_4CO_2$ complexes obtained with PBE+TS/light-tier1 level and light SCF parameters: configuration number (i), relative total energy (ΔE_{tot}), total magnetic moment (m_{tot}), HOMO energy (ε_H), LUMO energy (ε_L), and LUMO-HOMO energy gap (E_g).

i	ΔE_{tot} (meV)	m_{tot} (μ_B)	ε_H (eV)	ε_L (eV)	E_g (eV)
82	2132	1	−3.72	−3.54	0.18
167	2127	1	−3.63	−3.45	0.18
164	2119	1	−3.95	−3.80	0.15
288	2098	1	−3.66	−3.47	0.19
120	2096	1	−3.97	−3.79	0.18
134	2095	1	−3.41	−3.22	0.20
211	2090	1	−3.64	−3.45	0.19
235	2080	1	−3.90	−3.75	0.15
277	2075	1	−3.95	−3.73	0.21
213	2070	1	−3.74	−3.60	0.13
347	2069	1	−3.72	−3.55	0.17
2	2068	1	−3.79	−3.66	0.13
106	2064	1	−3.52	−3.37	0.15
7	2051	1	−3.89	−3.57	0.32
294	2050	1	−3.79	−3.62	0.17
305	2048	1	−3.91	−3.74	0.17
6	2041	1	−3.56	−3.38	0.18
198	2035	1	−3.89	−3.73	0.16
266	2034	1	−3.58	−3.37	0.22
336	2031	1	−3.64	−3.47	0.17
201	2027	1	−3.90	−3.74	0.16
328	2010	1	−3.75	−3.57	0.19

Continued on next page

Supplementary Table 28: Energetic and electronic properties for $[Cu_8Zn_3](Cp^*)_3(Mes)_4CO_2$ complexes obtained with PBE+TS/light-tier1 level and light SCF parameters: configuration number (i), relative total energy (ΔE_{tot}), total magnetic moment (m_{tot}), HOMO energy (ε_H), LUMO energy (ε_L), and LUMO-HOMO energy gap (E_g).

i	ΔE_{tot} (meV)	m_{tot} (μ_B)	ε_H (eV)	ε_L (eV)	E_g (eV)
274	2009	1	−3.68	−3.52	0.16
210	1997	1	−3.97	−3.81	0.15
18	1978	1	−3.81	−3.64	0.17
65	1977	1	−3.57	−3.42	0.15
140	1968	1	−3.92	−3.75	0.17
334	1962	1	−3.76	−3.59	0.18
117	1956	1	−3.79	−3.59	0.20
31	1947	1	−3.74	−3.59	0.16
240	1938	1	−4.01	−3.86	0.14
192	1929	1	−3.50	−3.32	0.18
172	1923	1	−3.72	−3.51	0.21
32	1911	1	−3.81	−3.64	0.17
246	1908	1	−3.77	−3.61	0.16
333	1904	1	−3.65	−3.48	0.17
324	1902	1	−3.47	−3.29	0.18
25	1902	1	−3.94	−3.80	0.14
326	1900	1	−3.46	−3.30	0.16
66	1886	1	−3.79	−3.58	0.21
85	1885	1	−3.69	−3.52	0.17
50	1878	1	−3.94	−3.79	0.15
1	1875	1	−3.73	−3.56	0.17
331	1870	1	−3.69	−3.50	0.19

Continued on next page

Supplementary Table 28: Energetic and electronic properties for $[Cu_8Zn_3](Cp^*)_3(Mes)_4CO_2$ complexes obtained with PBE+TS/light-tier1 level and light SCF parameters: configuration number (i), relative total energy (ΔE_{tot}), total magnetic moment (m_{tot}), HOMO energy (ε_H), LUMO energy (ε_L), and LUMO-HOMO energy gap (E_g).

i	ΔE_{tot} (meV)	m_{tot} (μ_B)	ε_H (eV)	ε_L (eV)	E_g (eV)
42	1865	1	−3.46	−3.29	0.17
176	1864	1	−3.59	−3.42	0.17
278	1860	1	−3.99	−3.81	0.18
225	1859	1	−3.75	−3.55	0.20
95	1854	1	−3.82	−3.68	0.13
132	1838	1	−3.72	−3.55	0.16
253	1830	1	−3.77	−3.61	0.16
327	1818	1	−3.99	−3.81	0.18
241	1815	1	−3.95	−3.54	0.40
4	1804	1	−3.81	−3.63	0.19
344	1801	1	−3.58	−3.38	0.20
224	1793	1	−3.64	−3.48	0.16
299	1772	1	−3.56	−3.39	0.18
283	1764	1	−3.40	−3.21	0.19
155	1737	1	−3.30	−3.15	0.15
273	1731	1	−3.45	−3.21	0.24
215	1726	1	−3.80	−3.64	0.17
193	1719	1	−3.48	−3.19	0.29
303	1711	1	−3.77	−3.61	0.16
255	1706	1	−3.42	−3.24	0.18
48	1702	1	−3.66	−3.51	0.15
177	1682	1	−3.66	−3.48	0.18

Continued on next page

Supplementary Table 28: Energetic and electronic properties for $[Cu_8Zn_3](Cp^*)_3(Mes)_4CO_2$ complexes obtained with PBE+TS/light-tier1 level and light SCF parameters: configuration number (i), relative total energy (ΔE_{tot}), total magnetic moment (m_{tot}), HOMO energy (ε_H), LUMO energy (ε_L), and LUMO-HOMO energy gap (E_g).

i	ΔE_{tot} (meV)	m_{tot} (μ_B)	ε_H (eV)	ε_L (eV)	E_g (eV)
44	1649	1	−3.59	−3.44	0.15
286	1643	1	−3.60	−3.45	0.15
318	1623	1	−3.67	−3.46	0.22
339	1606	1	−3.81	−3.65	0.16
182	1604	1	−3.78	−3.61	0.17
290	1554	1	−3.39	−3.24	0.15
53	1469	1	−3.49	−3.35	0.15
127	1466	1	−3.18	−3.03	0.15
115	1461	1	−3.18	−2.99	0.19
263	1455	1	−3.70	−3.52	0.18
162	1427	1	−3.57	−3.42	0.16
349	1398	1	−3.24	−3.05	0.20
302	1394	1	−3.48	−3.33	0.16
325	1385	1	−3.64	−3.48	0.16
188	1385	1	−3.79	−3.60	0.19
52	1384	1	−3.95	−3.82	0.13
157	1308	1	−3.76	−3.60	0.16
217	1276	1	−3.20	−3.02	0.18
135	1267	1	−3.59	−3.44	0.15
262	1151	1	−3.60	−3.45	0.15
332	1134	1	−3.74	−3.57	0.17
337	1122	1	−3.39	−3.24	0.15

Continued on next page

Supplementary Table 28: Energetic and electronic properties for $[Cu_8Zn_3](Cp^*)_3(Mes)_4CO_2$ complexes obtained with PBE+TS/light-tier1 level and light SCF parameters: configuration number (i), relative total energy (ΔE_{tot}), total magnetic moment (m_{tot}), HOMO energy (ε_H), LUMO energy (ε_L), and LUMO-HOMO energy gap (E_g).

i	ΔE_{tot} (meV)	m_{tot} (μ_B)	ε_H (eV)	ε_L (eV)	E_g (eV)
237	1108	1	−3.64	−3.48	0.15
151	1099	1	−3.65	−3.47	0.17
75	1087	1	−3.56	−3.34	0.22
314	1066	1	−3.40	−3.23	0.17
306	1030	1	−3.83	−3.63	0.20
348	979	1	−3.14	−2.95	0.19
311	938	1	−3.12	−2.93	0.20
51	913	1	−3.19	−3.03	0.16
307	901	1	−3.85	−3.67	0.18
310	719	1	−3.67	−3.51	0.16
321	392	1	−3.41	−3.24	0.17
312	366	1	−3.06	−2.90	0.16
309	214	1	−3.07	−2.90	0.17
316	0	1	−3.08	−2.92	0.17

Supplementary Table 29: Optimized and filtered representative structures of $[Cu_8Zn_3](Cp^*)_3(Mes)_4CO_2$ complexes obtained with PBE+TS/light-tier1 level and light SCF parameters. The relative total energy (ΔE_{tot}) is depicted above each structure.

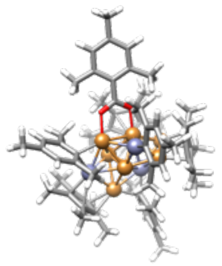	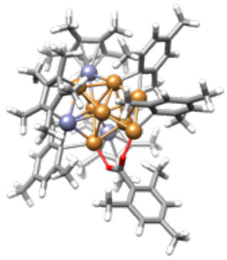	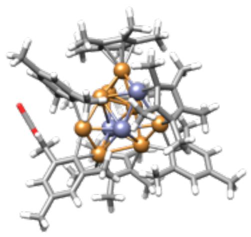
0 meV	214 meV	913 meV

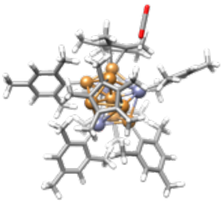

1.31 eV

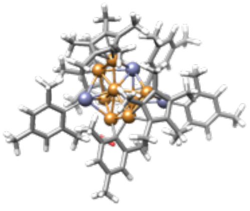

1.70 eV

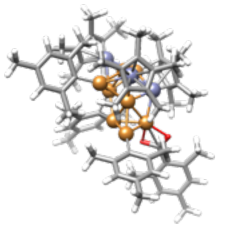

1.82 eV

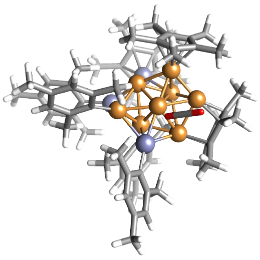

1.85 eV

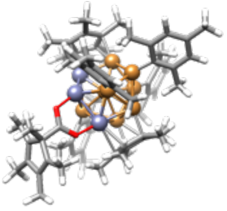

1.87 eV

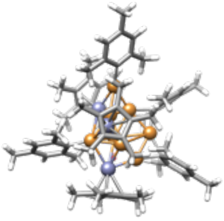

1.90 eV

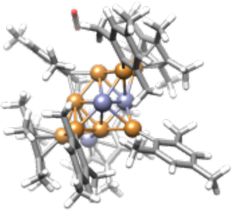

1.94 eV

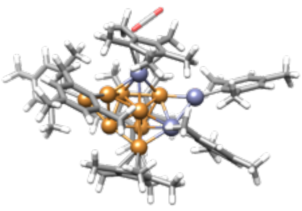

1.97 eV

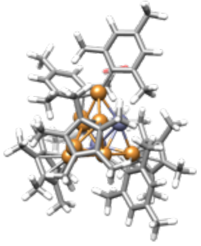

1.98 eV

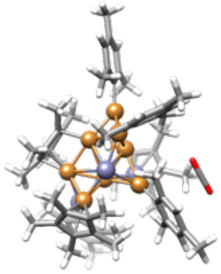

2.00 eV

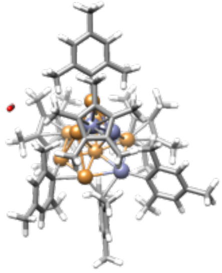

2.06 eV

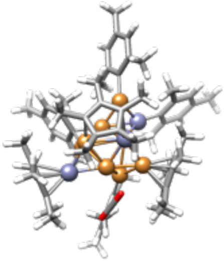

2.09 eV

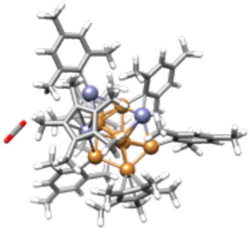

2.15 eV

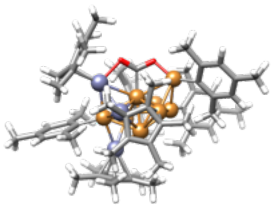

2.15 eV

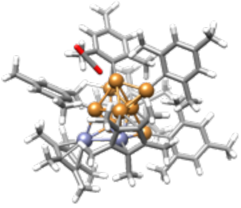

2.32 eV

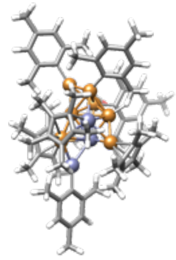

2.33 eV

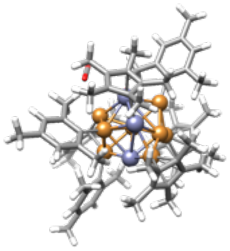

2.39 eV

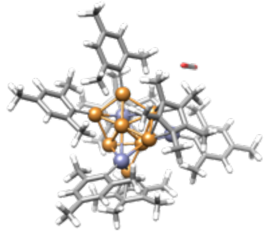

2.78 eV

2.7.6 $[Cu_8Zn_3](Cp^*)_4(Mes)_3H$ Complexes

Supplementary Table 30: Energetic and electronic properties for $[Cu_8Zn_3](Cp^*)_4(Mes)_3H$ complexes obtained with PBE+TS/light-tier1 level and light SCF parameters: configuration number (i), relative total energy (ΔE_{tot}), total magnetic moment (m_{tot}), HOMO energy (ε_H), LUMO energy (ε_L), and LUMO-HOMO energy gap (E_g).

i	ΔE_{tot} (meV)	m_{tot} (μ_B)	ε_H (eV)	ε_L (eV)	E_g (eV)
183	6846	2	−3.90	−3.59	0.31
191	6038	0	−3.99	−3.23	0.77
263	5476	0	−3.97	−3.82	0.15
134	5231	0	−3.70	−3.47	0.23
138	5082	0	−3.90	−3.70	0.20
71	4996	0	−3.70	−3.31	0.40
102	4673	0	−3.91	−3.03	0.87
88	4616	0	−3.85	−3.70	0.15
15	4429	0	−4.46	−3.42	1.04
256	4426	0	−3.82	−3.49	0.33
117	4395	0	−3.81	−3.60	0.21
32	4382	0	−4.12	−3.34	0.78
91	4339	0	−3.72	−3.55	0.17
19	4139	0	−4.06	−2.83	1.23

Continued on next page

Supplementary Table 30: Energetic and electronic properties for $[Cu_8Zn_3](Cp^*)_4(Mes)_3H$ complexes obtained with PBE+TS/light-tier1 level and light SCF parameters: configuration number (i), relative total energy (ΔE_{tot}), total magnetic moment (m_{tot}), HOMO energy (ε_H), LUMO energy (ε_L), and LUMO-HOMO energy gap (E_g).

i	ΔE_{tot} (meV)	m_{tot} (μ_B)	ε_H (eV)	ε_L (eV)	E_g (eV)
295	4133	0	−3.42	−3.23	0.19
37	4051	0	−3.94	−3.78	0.15
42	4044	0	−4.03	−2.84	1.20
246	4028	0	−4.17	−3.19	0.98
153	3862	0	−3.96	−3.12	0.84
104	3808	0	−3.75	−3.05	0.70
202	3802	0	−3.79	−3.06	0.74
293	3752	0	−3.58	−3.42	0.17
103	3730	2	−3.44	−3.27	0.17
285	3675	0	−3.60	−3.41	0.19
196	3660	0	−4.36	−4.21	0.14
74	3581	0	−3.80	−3.61	0.18
10	3578	0	−4.03	−3.45	0.58
292	3576	0	−3.54	−3.42	0.13
150	3542	0	−3.65	−3.45	0.20
167	3518	0	−3.91	−3.74	0.17
259	3450	0	−3.67	−3.52	0.15
77	3421	0	−3.69	−3.09	0.60
14	3356	0	−3.47	−3.31	0.17
174	3248	0	−3.59	−3.44	0.15
243	3239	0	−3.35	−3.05	0.31
187	3195	0	−3.93	−3.77	0.16

Continued on next page

Supplementary Table 30: Energetic and electronic properties for $[Cu_8Zn_3](Cp^*)_4(Mes)_3H$ complexes obtained with PBE+TS/light-tier1 level and light SCF parameters: configuration number (i), relative total energy (ΔE_{tot}), total magnetic moment (m_{tot}), HOMO energy (ε_H), LUMO energy (ε_L), and LUMO-HOMO energy gap (E_g).

i	ΔE_{tot} (meV)	m_{tot} (μ_B)	ε_H (eV)	ε_L (eV)	E_g (eV)
110	3191	0	−3.68	−3.25	0.44
296	3174	0	−3.94	−3.75	0.18
165	3168	0	−3.18	−3.04	0.14
120	3144	0	−3.81	−3.71	0.10
170	3137	0	−4.04	−3.38	0.67
137	3124	0	−3.78	−3.57	0.21
51	3102	0	−4.13	−3.95	0.18
203	3077	0	−3.22	−3.11	0.11
241	3075	0	−3.59	−3.42	0.17
255	3068	2	−3.98	−3.86	0.12
79	2958	0	−3.89	−3.20	0.69
298	2936	2	−3.50	−3.34	0.16
156	2930	0	−3.61	−3.48	0.13
242	2920	0	−3.54	−3.05	0.49
82	2843	0	−3.99	−3.38	0.61
215	2833	0	−4.11	−2.98	1.13
80	2798	0	−3.82	−3.62	0.20
101	2775	0	−3.80	−3.62	0.18
271	2702	0	−3.87	−3.05	0.83
121	2655	0	−3.72	−3.51	0.21
222	2639	0	−4.02	−2.77	1.25
189	2634	0	−4.01	−2.97	1.04

Continued on next page

Supplementary Table 30: Energetic and electronic properties for $[Cu_8Zn_3](Cp^*)_4(Mes)_3H$ complexes obtained with PBE+TS/light-tier1 level and light SCF parameters: configuration number (i), relative total energy (ΔE_{tot}), total magnetic moment (m_{tot}), HOMO energy (ε_H), LUMO energy (ε_L), and LUMO-HOMO energy gap (E_g).

i	ΔE_{tot} (meV)	m_{tot} (μ_B)	ε_H (eV)	ε_L (eV)	E_g (eV)
221	2626	0	−3.99	−3.10	0.89
123	2624	0	−3.62	−3.01	0.61
126	2604	0	−4.10	−3.11	0.99
35	2591	0	−3.64	−2.72	0.92
276	2581	0	−3.93	−2.80	1.13
313	2431	0	−4.49	−3.57	0.92
25	2430	0	−3.48	−3.30	0.18
232	2424	0	−4.14	−3.64	0.50
319	2409	0	−4.16	−3.28	0.88
249	2323	0	−3.78	−2.73	1.05
317	2307	0	−4.29	−3.42	0.87
129	2303	0	−4.09	−3.22	0.87
192	2288	0	−4.07	−2.87	1.21
198	2272	0	−3.92	−3.74	0.18
125	2270	0	−4.06	−3.23	0.83
305	2243	0	−3.75	−3.13	0.62
154	2224	0	−4.04	−3.23	0.80
152	2213	0	−4.09	−3.01	1.08
274	2199	0	−4.05	−3.30	0.75
226	2196	0	−4.16	−3.07	1.09
267	2172	0	−4.14	−3.12	1.01
52	2152	0	−3.90	−3.20	0.70

Continued on next page

Supplementary Table 30: Energetic and electronic properties for $[Cu_8Zn_3](Cp^*)_4(Mes)_3H$ complexes obtained with PBE+TS/light-tier1 level and light SCF parameters: configuration number (i), relative total energy (ΔE_{tot}), total magnetic moment (m_{tot}), HOMO energy (ε_H), LUMO energy (ε_L), and LUMO-HOMO energy gap (E_g).

i	ΔE_{tot} (meV)	m_{tot} (μ_B)	ε_H (eV)	ε_L (eV)	E_g (eV)
114	2147	0	−4.13	−3.37	0.76
320	2125	0	−4.11	−3.61	0.51
132	2124	0	−3.93	−3.51	0.42
147	2113	0	−3.59	−3.03	0.56
236	2100	0	−4.13	−3.10	1.03
244	2083	0	−4.20	−2.98	1.23
176	2063	0	−4.18	−3.30	0.88
149	2048	0	−4.12	−3.35	0.77
185	2024	0	−3.78	−2.73	1.05
195	2013	0	−4.04	−3.08	0.96
12	2008	0	−3.71	−3.58	0.14
97	1980	0	−4.11	−2.97	1.15
297	1965	0	−4.00	−2.59	1.40
53	1962	0	−4.29	−3.63	0.66
224	1944	0	−4.09	−3.21	0.88
252	1943	0	−4.05	−3.49	0.56
4	1900	0	−3.61	−3.32	0.29
34	1891	0	−3.89	−2.97	0.92
66	1833	0	−3.84	−2.95	0.89
155	1825	0	−4.05	−3.19	0.86
310	1821	0	−4.45	−3.45	1.00
8	1813	0	−3.83	−2.60	1.24

Continued on next page

Supplementary Table 30: Energetic and electronic properties for $[Cu_8Zn_3](Cp^*)_4(Mes)_3H$ complexes obtained with PBE+TS/light-tier1 level and light SCF parameters: configuration number (i), relative total energy (ΔE_{tot}), total magnetic moment (m_{tot}), HOMO energy (ε_H), LUMO energy (ε_L), and LUMO-HOMO energy gap (E_g).

i	ΔE_{tot} (meV)	m_{tot} (μ_B)	ε_H (eV)	ε_L (eV)	E_g (eV)
46	1810	0	−3.80	−2.81	0.99
314	1807	0	−4.27	−3.08	1.19
268	1784	0	−4.22	−3.38	0.84
321	1774	0	−4.24	−2.99	1.24
261	1741	0	−3.94	−3.56	0.38
9	1731	0	−3.44	−3.32	0.12
127	1712	0	−3.94	−3.08	0.86
257	1689	0	−4.27	−3.04	1.24
143	1683	0	−4.10	−3.18	0.91
163	1676	0	−4.25	−3.13	1.12
61	1670	0	−4.16	−3.32	0.83
164	1665	0	−3.86	−3.34	0.52
288	1648	0	−3.78	−2.77	1.00
161	1644	0	−3.80	−3.08	0.72
300	1641	0	−4.16	−2.90	1.26
139	1625	0	−4.25	−3.01	1.24
287	1613	0	−3.98	−3.47	0.51
227	1604	0	−4.08	−3.26	0.82
124	1592	0	−4.20	−3.10	1.10
99	1586	0	−4.30	−2.66	1.64
159	1580	0	−3.90	−2.90	1.00
23	1570	0	−3.88	−2.97	0.91

Continued on next page

Supplementary Table 30: Energetic and electronic properties for $[Cu_8Zn_3](Cp^*)_4(Mes)_3H$ complexes obtained with PBE+TS/light-tier1 level and light SCF parameters: configuration number (i), relative total energy (ΔE_{tot}), total magnetic moment (m_{tot}), HOMO energy (ε_H), LUMO energy (ε_L), and LUMO-HOMO energy gap (E_g).

i	ΔE_{tot} (meV)	m_{tot} (μ_B)	ε_H (eV)	ε_L (eV)	E_g (eV)
48	1547	0	−3.87	−2.75	1.12
190	1513	0	−4.02	−3.17	0.85
18	1508	0	−3.70	−3.22	0.47
316	1505	0	−4.31	−3.49	0.82
307	1505	0	−3.70	−2.94	0.76
225	1499	0	−4.04	−3.22	0.82
72	1496	0	−3.80	−3.02	0.78
245	1492	0	−4.23	−3.19	1.04
266	1456	0	−3.98	−2.97	1.01
131	1445	0	−4.13	−3.28	0.86
108	1408	0	−3.95	−2.88	1.07
118	1384	0	−3.80	−3.10	0.70
304	1358	0	−4.07	−3.16	0.91
5	1350	0	−4.09	−3.10	0.99
107	1336	0	−4.29	−3.30	0.99
201	1308	0	−4.31	−3.13	1.19
247	1286	0	−3.89	−3.07	0.82
27	1241	0	−3.81	−2.84	0.96
264	1210	0	−3.77	−2.74	1.04
223	1194	0	−3.77	−3.02	0.75
173	1193	0	−3.94	−3.28	0.66
39	1174	0	−4.01	−2.90	1.11

Continued on next page

Supplementary Table 30: Energetic and electronic properties for $[Cu_8Zn_3](Cp^*)_4(Mes)_3H$ complexes obtained with PBE+TS/light-tier1 level and light SCF parameters: configuration number (i), relative total energy (ΔE_{tot}), total magnetic moment (m_{tot}), HOMO energy (ε_H), LUMO energy (ε_L), and LUMO-HOMO energy gap (E_g).

i	ΔE_{tot} (meV)	m_{tot} (μ_B)	ε_H (eV)	ε_L (eV)	E_g (eV)
81	1168	0	−3.94	−3.01	0.93
303	1150	0	−3.74	−3.11	0.63
177	1141	0	−4.38	−3.11	1.27
83	1111	0	−3.95	−3.06	0.89
148	1101	0	−4.08	−2.76	1.32
106	1100	0	−3.78	−2.97	0.81
168	1074	0	−3.99	−2.89	1.11
308	1073	0	−3.97	−3.07	0.89
70	1066	0	−4.03	−3.05	0.98
279	1006	0	−4.03	−3.05	0.98
50	963	0	−4.14	−2.96	1.18
318	939	0	−4.08	−2.91	1.18
265	935	0	−4.00	−3.38	0.61
181	897	0	−3.83	−2.58	1.25
199	890	0	−3.92	−2.95	0.98
49	885	0	−3.91	−3.57	0.34
92	871	0	−4.26	−2.83	1.43
128	866	0	−4.16	−2.97	1.20
228	817	0	−4.06	−2.73	1.33
93	794	0	−3.84	−3.01	0.83
95	757	0	−3.79	−2.96	0.83
45	727	0	−3.69	−2.95	0.74

Continued on next page

Supplementary Table 30: Energetic and electronic properties for $[Cu_8Zn_3](Cp^*)_4(Mes)_3H$ complexes obtained with PBE+TS/light-tier1 level and light SCF parameters: configuration number (i), relative total energy (ΔE_{tot}), total magnetic moment (m_{tot}), HOMO energy (ε_H), LUMO energy (ε_L), and LUMO-HOMO energy gap (E_g).

i	ΔE_{tot} (meV)	m_{tot} (μ_B)	ε_H (eV)	ε_L (eV)	E_g (eV)
282	717	0	−4.13	−2.79	1.35
315	632	0	−4.42	−3.22	1.20
301	601	0	−4.30	−3.05	1.25
119	573	0	−3.97	−2.97	1.00
302	550	0	−4.28	−3.01	1.27
175	512	0	−4.02	−2.62	1.40
63	482	0	−3.81	−2.64	1.17
213	414	0	−3.74	−2.91	0.83
309	376	0	−4.04	−2.61	1.42
109	266	0	−3.76	−2.62	1.14
306	0	0	−3.92	−2.74	1.18

Supplementary Table 31: Optimized and filtered representative structures of $[Cu_8Zn_3](Cp^*)_4(Mes)_3H$ complexes obtained with PBE+TS/light-tier1 level and light SCF parameters. The relative total energy (ΔE_{tot}) is depicted above each structure.

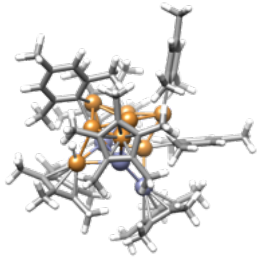

0 meV

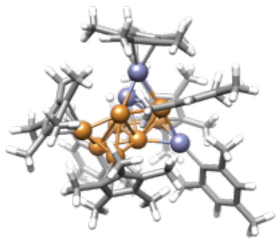

266 meV

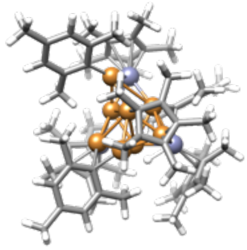

376 meV

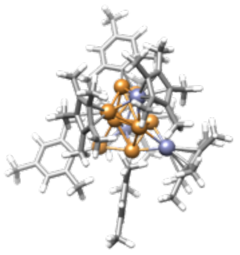
550 meV

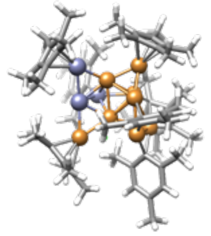
757 meV

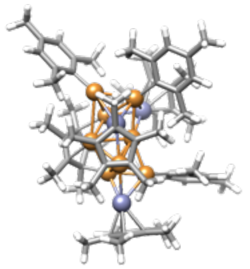
939 meV

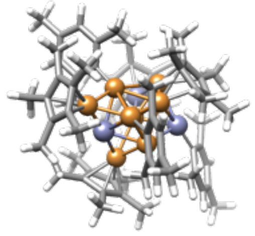
1.10 eV

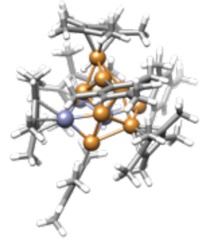
1.14 eV

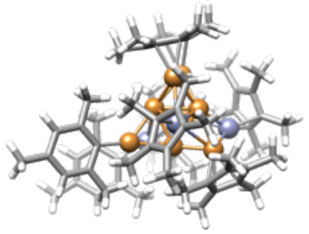
1.17 eV

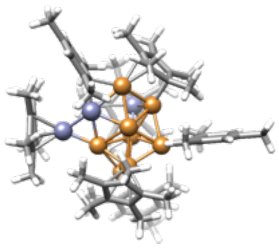
1.19 eV

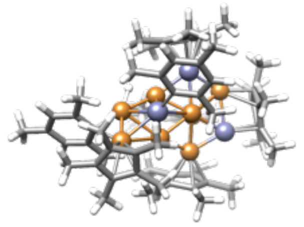
1.24 eV

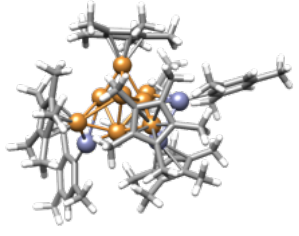
1.41 eV

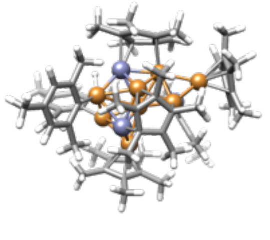
1.50 eV

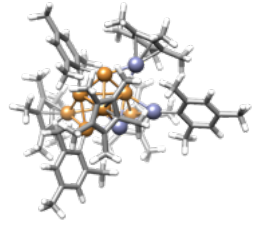
1.61 eV

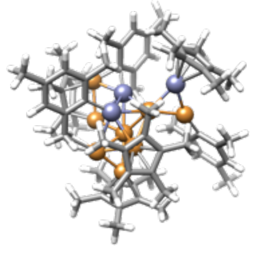
1.65 eV

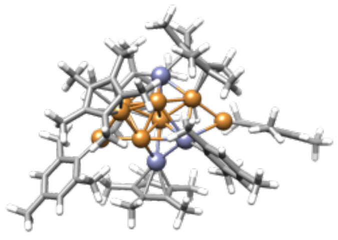
1.68 eV

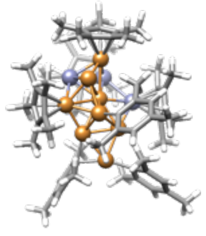
1.69 eV

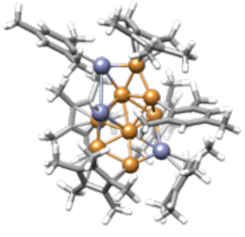
1.94 eV

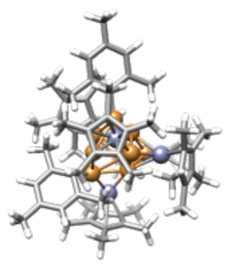

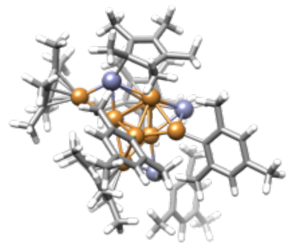

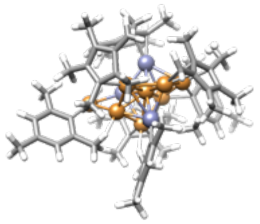

2.12 eV	2.15 eV	2.24 eV

2.7.7 $[Cu_9Zn_7](Cp^*)_6(Hex)_3H_3$ Complexes

Supplementary Table 32: Energetic and electronic properties for $[Cu_9Zn_7](Cp^*)_6(Hex)_3H_3$ complexes obtained with PBE+TS/light-tier1 level and light SCF parameters: configuration number (i), relative total energy (ΔE_{tot}), total magnetic moment (m_{tot}), HOMO energy (ε_H), LUMO energy (ε_L), and LUMO-HOMO energy gap (E_g).

i	ΔE_{tot} (meV)	m_{tot} (μ_B)	ε_H (eV)	ε_L (eV)	E_g (eV)
105	9805	2	−3.65	−3.43	0.22
4	9804	2	−3.15	−2.95	0.21
193	9542	2	−3.82	−3.54	0.28
108	9494	2	−3.90	−3.76	0.14
241	9073	2	−3.35	−3.17	0.18
282	8981	0	−3.70	−3.12	0.57
253	8962	0	−3.48	−3.31	0.17
205	8937	0	−3.74	−3.54	0.20
43	8677	2	−3.67	−3.56	0.12
142	8576	2	−3.70	−3.46	0.24
281	8544	2	−3.83	−3.65	0.18
162	8405	0	−3.51	−3.35	0.17
273	8276	0	−3.64	−3.14	0.50
236	8268	2	−3.72	−3.58	0.14

Continued on next page

Supplementary Table 32: Energetic and electronic properties for $[Cu_9Zn_7](Cp^*)_6(Hex)_3H_3$ complexes obtained with PBE+TS/light-tier1 level and light SCF parameters: configuration number (i), relative total energy (ΔE_{tot}), total magnetic moment (m_{tot}), HOMO energy (ε_H), LUMO energy (ε_L), and LUMO-HOMO energy gap (E_g).

i	ΔE_{tot} (meV)	m_{tot} (μ_B)	ε_H (eV)	ε_L (eV)	E_g (eV)
102	8259	2	−3.71	−3.47	0.23
221	8234	2	−3.51	−3.43	0.08
115	7416	2	−3.55	−3.38	0.17
52	7379	2	−3.35	−3.22	0.13
145	7355	2	−3.65	−3.51	0.13
144	7315	2	−3.81	−3.57	0.24
206	7247	0	−3.74	−3.23	0.51
53	7138	2	−3.77	−3.52	0.25
21	7116	0	−3.29	−2.87	0.42
242	7085	2	−3.65	−3.48	0.17
186	6973	2	−3.69	−3.68	0.02
101	6855	0	−3.42	−2.99	0.43
19	6571	0	−3.86	−2.99	0.87
201	6353	2	−3.48	−3.33	0.15
12	6293	0	−3.44	−3.07	0.37
55	6207	0	−3.73	−2.89	0.84
124	6136	0	−3.83	−3.33	0.50
294	6076	2	−3.66	−3.52	0.15
152	5919	0	−3.61	−2.81	0.80
313	5584	0	−3.85	−3.21	0.64
275	5509	0	−3.74	−2.85	0.89
2	5465	0	−3.79	−2.86	0.93

Continued on next page

Supplementary Table 32: Energetic and electronic properties for $[Cu_9Zn_7](Cp^*)_6(Hex)_3H_3$ complexes obtained with PBE+TS/light-tier1 level and light SCF parameters: configuration number (i), relative total energy (ΔE_{tot}), total magnetic moment (m_{tot}), HOMO energy (ε_H), LUMO energy (ε_L), and LUMO-HOMO energy gap (E_g).

i	ΔE_{tot} (meV)	m_{tot} (μ_B)	ε_H (eV)	ε_L (eV)	E_g (eV)
214	5433	0	−3.59	−2.84	0.75
324	5402	0	−3.84	−3.01	0.83
320	5299	0	−3.90	−3.04	0.86
196	4993	0	−3.62	−2.77	0.85
284	4885	0	−3.71	−3.00	0.71
93	4707	0	−3.64	−2.93	0.71
31	4705	0	−3.74	−2.92	0.82
181	4303	0	−3.61	−2.79	0.82
312	4199	0	−3.63	−3.14	0.49
306	4092	0	−3.81	−2.82	0.99
311	4076	0	−3.98	−2.83	1.15
132	4009	0	−3.68	−2.85	0.83
310	3956	0	−3.85	−2.88	0.98
163	3831	0	−3.68	−3.01	0.68
315	3601	0	−3.82	−2.76	1.07
318	3557	0	−3.77	−2.93	0.83
319	3334	0	−3.58	−2.83	0.75
322	3274	0	−3.55	−2.65	0.90
302	3128	0	−3.91	−2.79	1.12
314	3110	0	−3.77	−2.95	0.82
309	3070	0	−3.82	−3.03	0.80
305	2953	0	−3.87	−2.84	1.03

Continued on next page

Supplementary Table 32: Energetic and electronic properties for $[Cu_9Zn_7](Cp^*)_6(Hex)_3H_3$ complexes obtained with PBE+TS/light-tier1 level and light SCF parameters: configuration number (i), relative total energy (ΔE_{tot}), total magnetic moment (m_{tot}), HOMO energy (ε_H), LUMO energy (ε_L), and LUMO-HOMO energy gap (E_g).

i	ΔE_{tot} (meV)	m_{tot} (μ_B)	ε_H (eV)	ε_L (eV)	E_g (eV)
307	2854	0	−3.76	−2.76	1.00
321	2732	0	−3.60	−2.78	0.82
323	2606	0	−3.43	−2.87	0.56
316	2174	0	−3.67	−2.87	0.80
317	2126	0	−3.80	−3.06	0.74
328	1338	0	−3.61	−2.59	1.02
326	1081	0	−3.51	−2.70	0.81
327	1069	0	−3.69	−2.53	1.16
329	1028	0	−3.51	−2.65	0.87
330	986	0	−3.45	−2.67	0.78
331	934	0	−3.37	−2.44	0.93
335	379	0	−3.46	−2.62	0.83
332	319	0	−3.35	−2.42	0.93
334	231	0	−3.34	−2.56	0.78
333	226	0	−3.36	−2.69	0.66
336	0	0	−3.29	−2.65	0.65

Supplementary Table 33: Optimized and filtered representative structures of $[Cu_9Zn_7](Cp^*)_6(Hex)_3H_3$ complexes obtained with PBE/light-tier1 level and light SCF parameters. The relative total energy (ΔE_{tot}) is depicted above each structure.

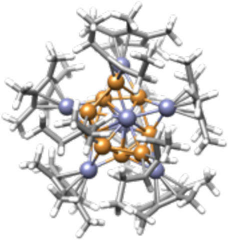

0 meV

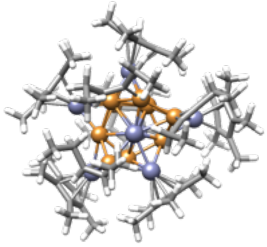

231 meV

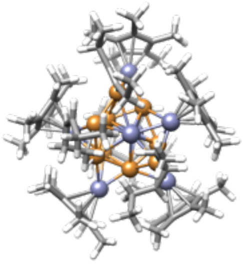

319 meV

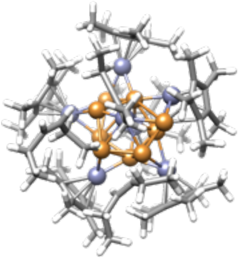

1.07 eV

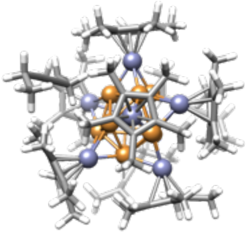

2.12 eV

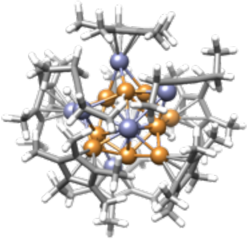

3.80 eV

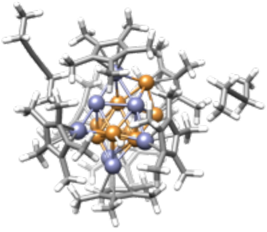

4.00 eV

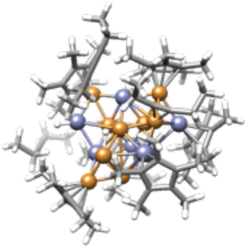

4.70 eV

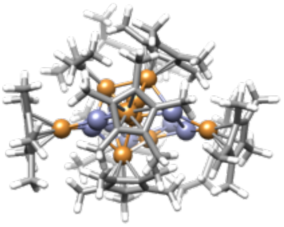

4.71 eV

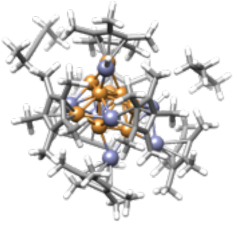

4.88 eV

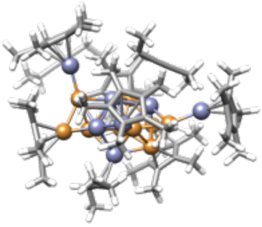

4.997 eV

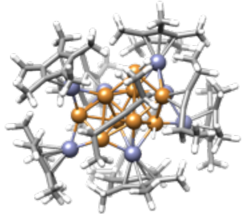

5.30 eV

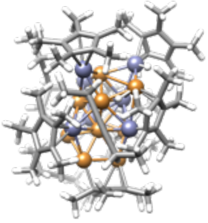

5.46 eV

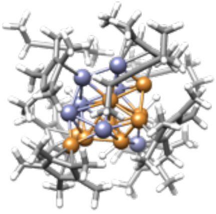

5.51 eV

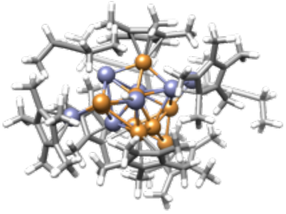

6.14 eV

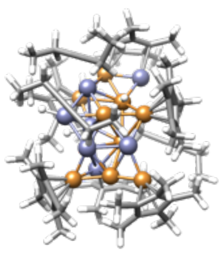

6.21 eV

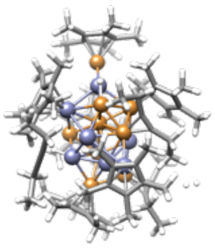

6.29 eV

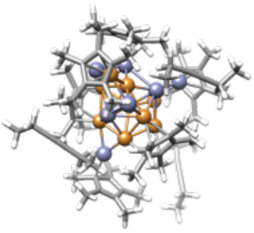

6.57 eV

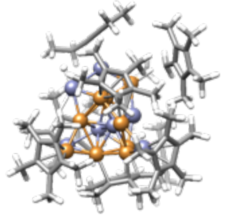

7.12 eV

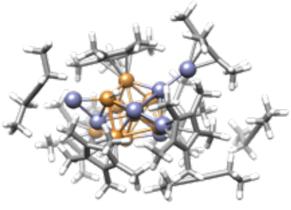

7.38 eV

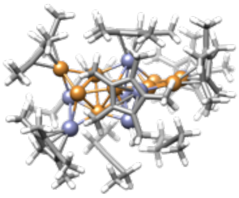

8.27 eV

2.7.8 $[Cu_{11}Zn_6](Cp^*)_8(CO_2)_2(HCO_2)$ Complexes

Supplementary Table 34: Energetic and electronic properties for $[Cu_{11}Zn_6](Cp^*)_8(HCO_2)(CO_2)_2$ complexes obtained with PBE+TS/light-tier1 level and light SCF parameters: configuration number (i), relative total energy (ΔE_{tot}), total magnetic moment (m_{tot}), HOMO energy (ε_H), LUMO energy (ε_L), and LUMO-HOMO energy gap (E_g).

i	ΔE_{tot} (meV)	m_{tot} (μ_B)	ε_H (eV)	ε_L (eV)	E_g (eV)
61	6360	1	−1.52	−1.51	0.01
195	6008	1	−1.76	−1.12	0.63
31	5989	1	−1.48	−1.44	0.04
204	5923	1	−1.50	−1.48	0.02
157	5842	1	−1.58	−1.57	0.02
214	5766	1	−1.02	−0.88	0.14
16	5583	1	−1.56	−1.52	0.04
252	5072	1	−1.52	−1.27	0.25
102	4859	1	−1.13	−1.00	0.13
184	4685	1	−1.17	−0.99	0.17

Continued on next page

Supplementary Table 34: Energetic and electronic properties for $[Cu_{11}Zn_6](Cp^*)_8(HCO_2)(CO_2)_2$ complexes obtained with PBE+TS/light-tier1 level and light SCF parameters: configuration number (i), relative total energy (ΔE_{tot}), total magnetic moment (m_{tot}), HOMO energy (ε_H), LUMO energy (ε_L), and LUMO-HOMO energy gap (E_g).

i	ΔE_{tot} (meV)	m_{tot} (μ_B)	ε_H (eV)	ε_L (eV)	E_g (eV)
41	4450	1	−1.81	−1.67	0.14
193	4353	1	−1.48	−1.13	0.35
283	4347	1	−1.95	−1.89	0.06
203	4344	1	−1.54	−1.37	0.17
25	4206	1	−1.29	−1.20	0.09
103	4182	1	−1.61	−1.59	0.02
162	4143	1	−1.50	−1.44	0.06
244	4049	1	−1.44	−1.37	0.07
29	4047	1	−1.85	−1.63	0.21
165	4032	1	−1.55	−1.53	0.02
262	3849	1	−1.70	−1.62	0.08
18	3842	1	−1.55	−1.53	0.02
153	3695	1	−1.84	−1.57	0.27
36	3629	1	−1.70	−1.68	0.02
20	3602	1	−1.41	−1.37	0.04
113	3594	1	−1.76	−1.70	0.06
93	3502	1	−1.15	−1.14	0.01
234	3455	1	−1.68	−1.65	0.03
28	3455	1	−1.35	−1.24	0.11
331	3429	1	−2.09	−1.99	0.11
206	3428	1	−1.62	−1.50	0.12
288	3407	3	−1.73	−1.58	0.14

Continued on next page

Supplementary Table 34: Energetic and electronic properties for $[Cu_{11}Zn_6](Cp^*)_8(HCO_2)(CO_2)_2$ complexes obtained with PBE+TS/light-tier1 level and light SCF parameters: configuration number (i), relative total energy (ΔE_{tot}), total magnetic moment (m_{tot}), HOMO energy (ε_H), LUMO energy (ε_L), and LUMO-HOMO energy gap (E_g).

i	ΔE_{tot} (meV)	m_{tot} (μ_B)	ε_H (eV)	ε_L (eV)	E_g (eV)
212	3387	1	−1.62	−1.44	0.18
211	3186	1	−1.55	−1.44	0.11
82	3177	1	−1.40	−1.30	0.10
174	3170	1	−1.90	−1.89	0.01
32	3060	1	−1.52	−1.48	0.04
143	3059	1	−1.82	−1.48	0.34
21	3055	1	−1.55	−1.46	0.09
43	2995	1	−1.63	−1.61	0.03
258	2981	1	−1.18	−1.17	0.01
285	2921	1	−1.75	−1.68	0.07
232	2910	1	−1.65	−1.63	0.02
161	2906	1	−1.60	−1.53	0.07
290	2878	1	−1.68	−1.60	0.08
224	2853	1	−1.97	−1.84	0.14
22	2835	1	−1.67	−1.65	0.02
154	2648	1	−1.67	−1.54	0.13
132	2599	1	−1.50	−1.48	0.02
268	2585	1	−1.62	−1.46	0.16
172	2578	1	−1.64	−1.53	0.11
282	2517	1	−1.13	−1.01	0.11
241	2491	1	−1.58	−1.37	0.21
295	2482	1	−1.46	−1.42	0.04

Continued on next page

Supplementary Table 34: Energetic and electronic properties for $[Cu_{11}Zn_6](Cp^*)_8(HCO_2)(CO_2)_2$ complexes obtained with PBE+TS/light-tier1 level and light SCF parameters: configuration number (i), relative total energy (ΔE_{tot}), total magnetic moment (m_{tot}), HOMO energy (ε_H), LUMO energy (ε_L), and LUMO-HOMO energy gap (E_g).

i	ΔE_{tot} (meV)	m_{tot} (μ_B)	ε_H (eV)	ε_L (eV)	E_g (eV)
306	2457	1	−1.74	−1.60	0.14
257	2428	1	−1.76	−1.66	0.10
259	2371	1	−1.61	−1.48	0.13
238	2367	1	−1.68	−1.66	0.02
191	2270	1	−1.65	−1.46	0.19
305	2183	1	−1.75	−1.60	0.15
13	2160	1	−1.34	−1.24	0.10
284	2150	1	−1.19	−1.06	0.13
37	2088	1	−1.73	−1.69	0.04
303	2013	1	−1.66	−1.55	0.11
319	1980	1	−1.41	−1.29	0.11
49	1877	1	−1.52	−1.41	0.11
26	1866	1	−1.76	−1.64	0.12
318	1840	1	−1.57	−1.45	0.12
221	1763	1	−1.51	−1.46	0.05
309	1717	1	−1.32	−1.20	0.11
46	1712	1	−1.65	−1.55	0.10
307	1674	1	−1.70	−1.59	0.11
225	1621	1	−1.53	−1.40	0.12
301	1597	1	−1.47	−1.38	0.10
121	1520	1	−1.26	−1.14	0.12
312	1459	1	−1.57	−1.45	0.11

Continued on next page

Supplementary Table 34: Energetic and electronic properties for $[Cu_{11}Zn_6](Cp^*)_8(HCO_2)(CO_2)_2$ complexes obtained with PBE+TS/light-tier1 level and light SCF parameters: configuration number (i), relative total energy (ΔE_{tot}), total magnetic moment (m_{tot}), HOMO energy (ε_H), LUMO energy (ε_L), and LUMO-HOMO energy gap (E_g).

i	ΔE_{tot} (meV)	m_{tot} (μ_B)	ε_H (eV)	ε_L (eV)	E_g (eV)
223	1426	1	−1.50	−1.39	0.12
327	1417	1	−1.53	−1.43	0.10
329	1324	1	−1.54	−1.42	0.12
325	1217	1	−1.57	−1.42	0.14
311	1211	1	−1.44	−1.33	0.11
315	1125	1	−1.45	−1.33	0.13
333	1096	1	−1.68	−1.57	0.11
308	1003	1	−1.39	−1.27	0.12
323	756	1	−1.42	−1.32	0.10
326	689	1	−1.46	−1.34	0.12
328	385	1	−1.33	−1.20	0.13
332	318	1	−1.57	−1.45	0.11
321	281	1	−1.54	−1.43	0.11
330	0	1	−1.27	−1.13	0.14

Supplementary Table 35: Optimized and filtered representative structures of $[Cu_{11}Zn_6](Cp^*)_8(CO_2)_2(HCO_2)$ complexes obtained with PBE/light-tier1 level and light SCF parameters. The relative total energy (ΔE_{tot}) is depicted above each structure.

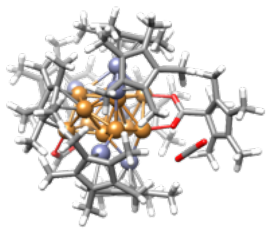	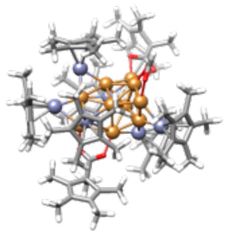	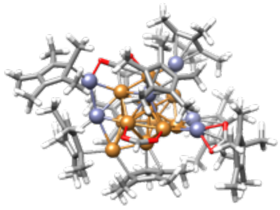
0 meV	385 meV	689 meV

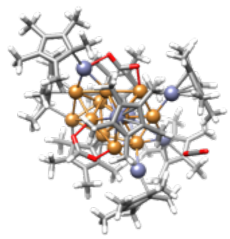

1.00 eV

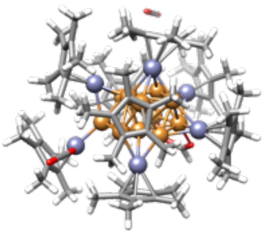

1.32 eV

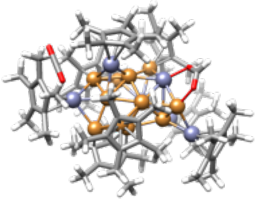

1.43 eV

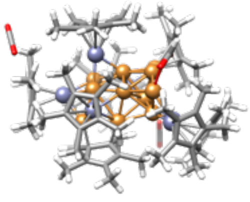

1.46 eV

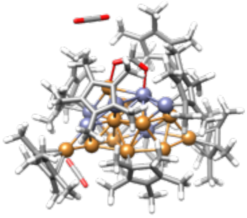

1.52 eV

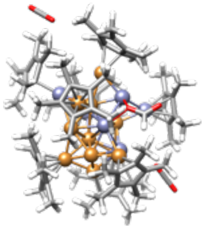

1.62 eV

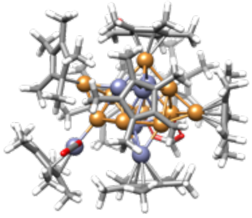

1.71 eV

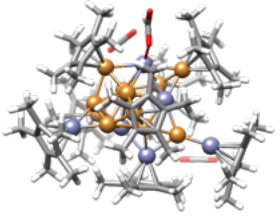

1.87 eV

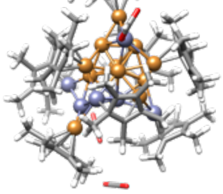

1.88 eV

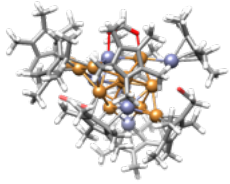

2.16 eV

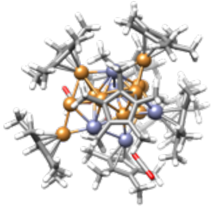

2.27 eV

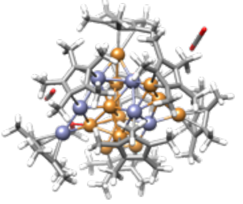

2.58 eV

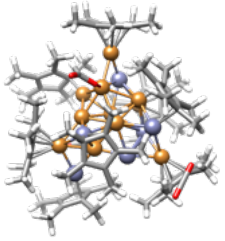

2.58 eV

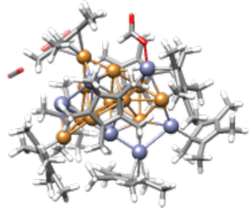

3.18 eV

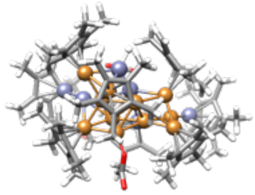

3.18 eV

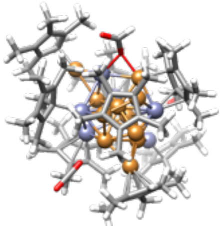

3.43 eV

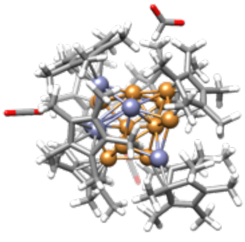

4.35 eV

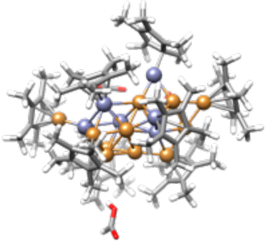

5.07 eV

2.7.9 $[Cu_8Al_6](Cp^*)_6$ Complexes

Supplementary Table 36: Energetic and electronic properties for $[Cu_8Al_6](Cp^*)_6$ complexes obtained with PBE+TS/light-tier1 level and light SCF parameters: configuration number (i), relative total energy (ΔE_{tot}), total magnetic moment (m_{tot}), HOMO energy (ε_H), LUMO energy (ε_L), and LUMO-HOMO energy gap (E_g).

i	ΔE_{tot} (meV)	m_{tot} (μ_B)	ε_H (eV)	ε_L (eV)	E_g (eV)
193	7749	0	−3.37	−3.05	0.33
55	6715	0	−3.26	−2.70	0.56
227	6615	0	−3.18	−2.53	0.64
188	6102	0	−3.12	−2.57	0.55
82	5917	0	−3.21	−2.98	0.23
13	5839	0	−3.05	−2.67	0.38
216	5673	0	−3.48	−3.04	0.44
36	5452	0	−3.34	−2.59	0.75
102	5417	0	−3.32	−2.58	0.73
262	5173	0	−3.05	−2.42	0.63
6	5164	0	−3.46	−2.82	0.64
75	5152	0	−3.39	−2.80	0.59
45	5125	0	−3.26	−2.72	0.54
105	5019	0	−3.10	−2.39	0.70

Continued on next page

Supplementary Table 36: Energetic and electronic properties for $[Cu_8Al_6](Cp^*)_6$ complexes obtained with PBE+TS/light-tier1 level and light SCF parameters: configuration number (i), relative total energy (ΔE_{tot}), total magnetic moment (m_{tot}), HOMO energy (ε_H), LUMO energy (ε_L), and LUMO-HOMO energy gap (E_g).

i	ΔE_{tot} (meV)	m_{tot} (μ_B)	ε_H (eV)	ε_L (eV)	E_g (eV)
276	4975	0	−3.21	−2.68	0.53
101	4902	0	−3.31	−2.97	0.34
134	4901	0	−3.19	−2.59	0.59
56	4887	0	−3.05	−2.47	0.58
278	4755	0	−3.50	−2.76	0.74
20	4717	0	−3.05	−2.65	0.40
61	4711	0	−3.44	−2.47	0.97
51	4600	0	−3.36	−2.77	0.59
180	4532	0	−3.19	−2.40	0.80
100	4506	0	−3.43	−2.77	0.66
57	4457	0	−3.24	−2.64	0.60
158	4415	0	−3.29	−2.57	0.72
201	4387	0	−3.19	−2.51	0.67
235	4358	0	−3.38	−2.98	0.40
202	4340	0	−3.23	−2.52	0.70
62	4262	0	−3.28	−2.43	0.85
171	4241	0	−3.33	−2.58	0.75
198	4238	0	−3.12	−2.68	0.44
14	4205	0	−3.54	−2.58	0.96
297	4203	0	−3.55	−2.89	0.66
311	4173	0	−2.94	−2.49	0.45
261	4157	0	−3.48	−2.67	0.81

Continued on next page

Supplementary Table 36: Energetic and electronic properties for $[Cu_8Al_6](Cp^*)_6$ complexes obtained with PBE+TS/light-tier1 level and light SCF parameters: configuration number (i), relative total energy (ΔE_{tot}), total magnetic moment (m_{tot}), HOMO energy (ε_H), LUMO energy (ε_L), and LUMO-HOMO energy gap (E_g).

i	ΔE_{tot} (meV)	m_{tot} (μ_B)	ε_H (eV)	ε_L (eV)	E_g (eV)
175	4066	0	−3.34	−2.62	0.71
48	4065	0	−3.26	−2.93	0.33
189	4021	0	−3.49	−2.62	0.87
190	4019	0	−2.93	−2.57	0.36
124	4004	0	−3.03	−2.64	0.39
108	4000	0	−3.31	−2.63	0.68
3	3966	0	−3.39	−2.36	1.03
248	3955	0	−3.18	−2.35	0.83
211	3927	0	−3.46	−2.93	0.53
196	3920	0	−3.26	−2.81	0.45
185	3914	0	−3.17	−2.92	0.26
296	3870	0	−3.24	−2.71	0.53
181	3862	0	−3.25	−2.61	0.64
68	3832	0	−3.39	−2.57	0.82
147	3794	0	−2.93	−2.62	0.31
139	3780	0	−3.23	−2.56	0.68
294	3765	0	−3.56	−2.98	0.58
83	3762	0	−3.04	−2.60	0.44
246	3745	0	−3.39	−2.29	1.10
179	3734	0	−3.59	−2.62	0.97
113	3729	0	−3.46	−2.70	0.75
312	3718	0	−2.94	−2.41	0.53

Continued on next page

Supplementary Table 36: Energetic and electronic properties for $[Cu_8Al_6](Cp^*)_6$ complexes obtained with PBE+TS/light-tier1 level and light SCF parameters: configuration number (i), relative total energy (ΔE_{tot}), total magnetic moment (m_{tot}), HOMO energy (ε_H), LUMO energy (ε_L), and LUMO-HOMO energy gap (E_g).

i	ΔE_{tot} (meV)	m_{tot} (μ_B)	ε_H (eV)	ε_L (eV)	E_g (eV)
95	3717	0	−3.52	−2.70	0.82
264	3702	0	−3.11	−2.47	0.64
149	3666	0	−3.32	−2.51	0.81
265	3625	0	−3.46	−2.78	0.68
131	3611	0	−3.48	−2.92	0.56
16	3534	0	−3.10	−2.38	0.72
126	3526	0	−3.28	−2.50	0.78
168	3513	0	−3.43	−2.89	0.53
274	3483	0	−3.29	−2.63	0.65
325	3472	0	−3.31	−2.98	0.33
266	3471	0	−3.61	−2.81	0.80
215	3465	0	−3.52	−2.65	0.87
281	3456	0	−3.32	−2.68	0.64
118	3448	0	−3.00	−2.42	0.58
191	3423	0	−3.37	−2.84	0.53
243	3417	0	−3.28	−2.46	0.82
161	3410	0	−3.35	−2.93	0.42
41	3399	0	−3.65	−2.68	0.97
44	3360	0	−3.57	−2.63	0.95
213	3347	0	−3.39	−2.71	0.68
169	3343	0	−3.50	−2.50	1.00
295	3340	0	−3.18	−2.63	0.55

Continued on next page

Supplementary Table 36: Energetic and electronic properties for $[Cu_8Al_6](Cp^*)_6$ complexes obtained with PBE+TS/light-tier1 level and light SCF parameters: configuration number (i), relative total energy (ΔE_{tot}), total magnetic moment (m_{tot}), HOMO energy (ε_H), LUMO energy (ε_L), and LUMO-HOMO energy gap (E_g).

i	ΔE_{tot} (meV)	m_{tot} (μ_B)	ε_H (eV)	ε_L (eV)	E_g (eV)
28	3310	0	−3.54	−2.81	0.72
289	3298	0	−3.55	−2.71	0.84
221	3291	0	−3.15	−2.64	0.51
199	3258	0	−3.01	−2.19	0.82
99	3255	0	−3.15	−2.62	0.52
129	3243	0	−3.49	−2.74	0.75
73	3223	0	−3.40	−2.69	0.71
222	3223	0	−3.01	−2.32	0.69
29	3209	0	−3.62	−2.58	1.04
220	3193	0	−2.91	−2.29	0.63
174	3155	0	−3.44	−2.77	0.67
208	3142	0	−3.39	−2.79	0.59
109	3139	0	−3.00	−2.39	0.61
77	3130	0	−3.52	−2.82	0.69
200	3129	0	−3.60	−2.82	0.78
292	3121	0	−3.10	−2.62	0.48
275	3119	0	−3.54	−2.99	0.56
42	3104	0	−3.11	−2.10	1.01
123	3102	0	−3.20	−2.38	0.82
89	3094	0	−3.40	−2.82	0.58
231	3092	0	−3.42	−2.68	0.74
97	3070	0	−3.04	−2.51	0.53

Continued on next page

Supplementary Table 36: Energetic and electronic properties for $[Cu_8Al_6](Cp^*)_6$ complexes obtained with PBE+TS/light-tier1 level and light SCF parameters: configuration number (i), relative total energy (ΔE_{tot}), total magnetic moment (m_{tot}), HOMO energy (ε_H), LUMO energy (ε_L), and LUMO-HOMO energy gap (E_g).

i	ΔE_{tot} (meV)	m_{tot} (μ_B)	ε_H (eV)	ε_L (eV)	E_g (eV)
273	3048	0	−3.33	−2.49	0.84
114	3047	0	−3.39	−2.65	0.74
250	3025	0	−3.29	−2.64	0.65
122	3018	0	−3.26	−2.75	0.51
67	3015	0	−3.17	−2.66	0.50
98	3013	0	−3.36	−2.29	1.07
271	2981	0	−3.41	−2.82	0.59
214	2977	0	−3.54	−2.47	1.06
219	2971	0	−3.25	−2.74	0.51
288	2960	0	−3.47	−2.47	1.01
186	2947	0	−3.52	−2.65	0.87
277	2944	0	−3.27	−2.62	0.65
52	2944	0	−3.54	−2.67	0.87
323	2910	0	−3.28	−2.59	0.69
163	2888	0	−3.24	−2.64	0.59
263	2876	0	−3.43	−2.61	0.82
120	2865	0	−3.26	−2.75	0.51
166	2843	0	−3.40	−2.47	0.92
115	2826	0	−3.48	−2.92	0.56
284	2744	0	−3.34	−2.50	0.84
91	2739	0	−3.31	−2.60	0.71
207	2735	0	−3.46	−2.63	0.83

Continued on next page

Supplementary Table 36: Energetic and electronic properties for $[Cu_8Al_6](Cp^*)_6$ complexes obtained with PBE+TS/light-tier1 level and light SCF parameters: configuration number (i), relative total energy (ΔE_{tot}), total magnetic moment (m_{tot}), HOMO energy (ε_H), LUMO energy (ε_L), and LUMO-HOMO energy gap (E_g).

i	ΔE_{tot} (meV)	m_{tot} (μ_B)	ε_H (eV)	ε_L (eV)	E_g (eV)
25	2711	0	−3.26	−2.41	0.86
140	2693	0	−3.36	−2.57	0.79
92	2654	0	−3.16	−2.62	0.54
252	2651	0	−3.60	−2.37	1.23
249	2625	0	−3.28	−2.31	0.97
197	2623	0	−3.27	−2.17	1.09
69	2595	0	−3.31	−2.53	0.78
245	2593	0	−3.54	−2.42	1.13
72	2576	0	−3.36	−2.56	0.79
244	2549	0	−3.31	−2.22	1.09
125	2542	0	−3.18	−2.60	0.58
217	2525	0	−3.26	−2.63	0.62
194	2500	0	−3.63	−2.69	0.94
241	2500	0	−3.35	−2.57	0.78
177	2496	0	−3.46	−2.89	0.57
43	2479	0	−3.39	−2.98	0.41
18	2474	0	−3.15	−2.60	0.55
286	2472	0	−3.46	−2.51	0.95
150	2455	0	−3.27	−2.23	1.04
327	2449	0	−3.15	−2.58	0.57
121	2443	0	−3.21	−2.56	0.65
256	2435	0	−3.27	−2.64	0.63

Continued on next page

Supplementary Table 36: Energetic and electronic properties for $[Cu_8Al_6](Cp^*)_6$ complexes obtained with PBE+TS/light-tier1 level and light SCF parameters: configuration number (i), relative total energy (ΔE_{tot}), total magnetic moment (m_{tot}), HOMO energy (ε_H), LUMO energy (ε_L), and LUMO-HOMO energy gap (E_g).

i	ΔE_{tot} (meV)	m_{tot} (μ_B)	ε_H (eV)	ε_L (eV)	E_g (eV)
2	2432	0	−3.71	−2.40	1.32
184	2427	0	−3.33	−2.64	0.69
155	2416	0	−3.51	−2.49	1.03
80	2408	0	−3.52	−2.32	1.20
63	2393	0	−3.26	−2.14	1.12
267	2387	0	−3.31	−2.48	0.83
310	2386	0	−3.21	−2.30	0.91
1	2385	0	−3.21	−2.35	0.86
225	2361	0	−3.10	−2.62	0.48
164	2335	0	−3.28	−2.37	0.91
151	2331	0	−3.53	−2.37	1.16
128	2330	0	−3.46	−2.39	1.07
26	2328	0	−3.41	−2.33	1.08
137	2323	0	−3.63	−2.29	1.33
71	2292	0	−3.42	−2.20	1.21
127	2285	0	−3.42	−2.54	0.88
272	2282	0	−3.31	−2.45	0.86
309	2259	0	−3.35	−2.24	1.11
54	2259	0	−3.48	−2.44	1.05
22	2258	0	−3.50	−2.40	1.10
142	2218	0	−3.40	−2.45	0.96
232	2205	0	−3.23	−2.11	1.12

Continued on next page

Supplementary Table 36: Energetic and electronic properties for $[Cu_8Al_6](Cp^*)_6$ complexes obtained with PBE+TS/light-tier1 level and light SCF parameters: configuration number (i), relative total energy (ΔE_{tot}), total magnetic moment (m_{tot}), HOMO energy (ε_H), LUMO energy (ε_L), and LUMO-HOMO energy gap (E_g).

i	ΔE_{tot} (meV)	m_{tot} (μ_B)	ε_H (eV)	ε_L (eV)	E_g (eV)
291	2184	0	−3.38	−2.55	0.84
182	2181	0	−3.57	−2.69	0.88
141	2179	0	−3.22	−2.18	1.04
233	2158	0	−3.28	−2.18	1.10
170	2154	0	−3.07	−2.14	0.93
298	2151	0	−3.56	−2.13	1.43
31	2143	0	−3.38	−2.20	1.18
10	2140	0	−3.35	−2.55	0.80
253	2129	0	−3.17	−2.51	0.65
117	2091	0	−3.30	−2.31	0.99
328	2078	0	−2.94	−2.15	0.80
226	2068	0	−3.39	−2.49	0.90
322	2064	0	−3.47	−2.47	1.00
47	2060	0	−2.87	−2.30	0.57
324	2023	0	−2.79	−2.42	0.37
5	1991	0	−3.52	−2.85	0.67
318	1945	0	−3.10	−1.82	1.28
315	1940	0	−3.18	−2.53	0.64
17	1916	0	−3.24	−2.34	0.90
283	1912	0	−3.35	−2.23	1.12
132	1900	0	−3.33	−2.12	1.22
280	1892	0	−3.38	−2.23	1.16

Continued on next page

Supplementary Table 36: Energetic and electronic properties for $[Cu_8Al_6](Cp^*)_6$ complexes obtained with PBE+TS/light-tier1 level and light SCF parameters: configuration number (i), relative total energy (ΔE_{tot}), total magnetic moment (m_{tot}), HOMO energy (ε_H), LUMO energy (ε_L), and LUMO-HOMO energy gap (E_g).

i	ΔE_{tot} (meV)	m_{tot} (μ_B)	ε_H (eV)	ε_L (eV)	E_g (eV)
64	1867	0	−3.44	−2.26	1.18
32	1866	0	−2.95	−2.17	0.78
160	1866	0	−3.19	−2.25	0.95
205	1866	0	−3.61	−2.48	1.13
228	1849	0	−3.21	−2.11	1.10
234	1848	0	−3.70	−2.23	1.47
173	1824	0	−3.54	−2.46	1.09
254	1807	0	−3.45	−2.48	0.97
165	1795	0	−3.54	−2.33	1.20
304	1744	0	−3.14	−2.36	0.78
34	1710	0	−3.40	−2.22	1.18
11	1697	0	−3.53	−2.31	1.21
53	1681	0	−3.29	−2.47	0.82
178	1678	0	−3.22	−2.26	0.96
136	1657	0	−3.26	−2.21	1.05
247	1604	0	−3.42	−2.35	1.07
316	1601	0	−3.25	−2.02	1.23
258	1581	0	−3.24	−2.39	0.86
112	1578	0	−3.35	−2.09	1.26
203	1573	0	−3.52	−2.51	1.01
293	1564	0	−3.35	−1.89	1.45
153	1536	0	−3.31	−2.24	1.07

Continued on next page

Supplementary Table 36: Energetic and electronic properties for $[Cu_8Al_6](Cp^*)_6$ complexes obtained with PBE+TS/light-tier1 level and light SCF parameters: configuration number (i), relative total energy (ΔE_{tot}), total magnetic moment (m_{tot}), HOMO energy (ε_H), LUMO energy (ε_L), and LUMO-HOMO energy gap (E_g).

i	ΔE_{tot} (meV)	m_{tot} (μ_B)	ε_H (eV)	ε_L (eV)	E_g (eV)
183	1501	0	−3.38	−2.30	1.07
76	1487	0	−3.30	−2.52	0.78
135	1470	0	−3.50	−2.32	1.18
329	1469	0	−3.17	−2.42	0.75
148	1457	0	−3.17	−2.06	1.11
27	1441	0	−3.33	−2.26	1.07
326	1437	0	−3.03	−1.97	1.06
145	1383	0	−3.24	−2.11	1.13
172	1365	0	−3.47	−2.66	0.81
79	1357	0	−3.25	−2.02	1.23
224	1323	0	−3.42	−2.24	1.19
212	1202	0	−3.35	−2.00	1.35
317	1190	0	−3.15	−1.86	1.29
7	1185	0	−3.43	−2.27	1.16
218	939	0	−3.25	−2.12	1.13
242	885	0	−3.21	−2.42	0.79
60	713	0	−3.24	−1.96	1.28
96	466	0	−3.20	−2.24	0.96
308	461	0	−3.10	−1.86	1.24
162	224	0	−3.15	−1.94	1.22
306	197	0	−2.97	−1.97	1.00
301	184	0	−3.29	−1.72	1.57

Continued on next page

Supplementary Table 36: Energetic and electronic properties for $[Cu_8Al_6](Cp^*)_6$ complexes obtained with PBE+TS/light-tier1 level and light SCF parameters: configuration number (i), relative total energy (ΔE_{tot}), total magnetic moment (m_{tot}), HOMO energy (ε_H), LUMO energy (ε_L), and LUMO-HOMO energy gap (E_g).

i	ΔE_{tot} (meV)	m_{tot} (μ_B)	ε_H (eV)	ε_L (eV)	E_g (eV)
330	96	0	−3.15	−1.92	1.24
303	0	0	−3.19	−1.76	1.43

Supplementary Table 37: Optimized and filtered representative structures of $[Cu_8Al_6](Cp^*)_6$ complexes obtained with PBE+TS/light-tier1 level and light SCF parameters. The relative total energy (ΔE_{tot}) is depicted above each structure.

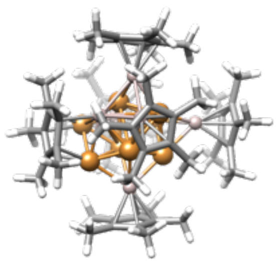
0 meV

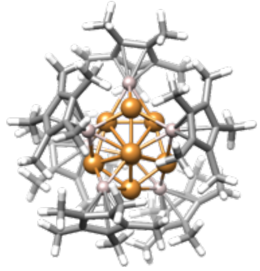
96 meV

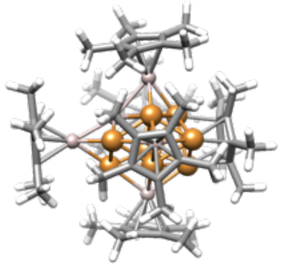
224 meV

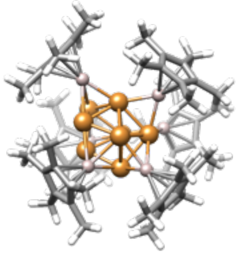
1.19 eV

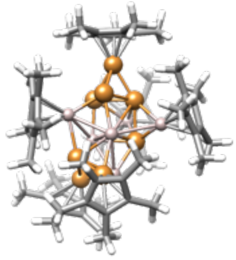
1.57 eV

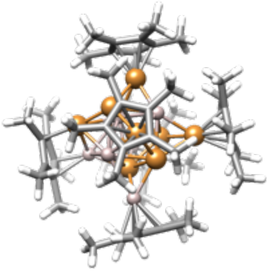
1.71 eV

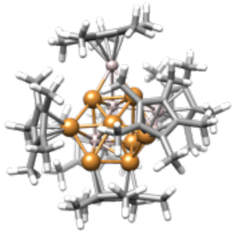
1.87 eV

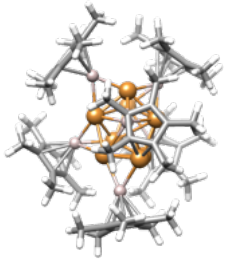
2.08 eV

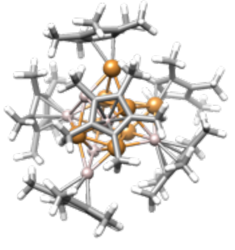
2.33 eV

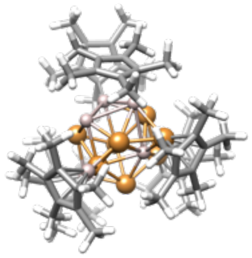

2.43 eV

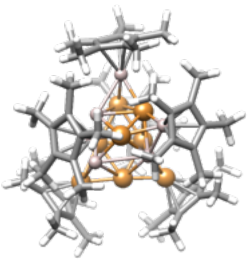

2.46 eV

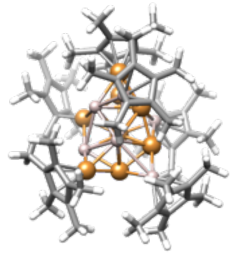

2.73 eV

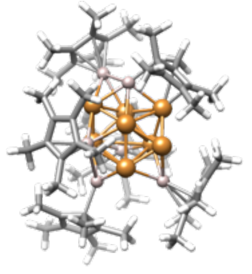

2.91 eV

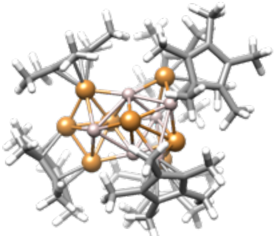

2.94 eV

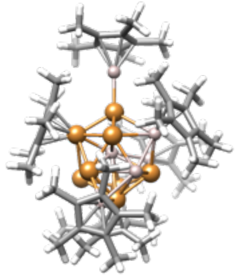

3.07 eV

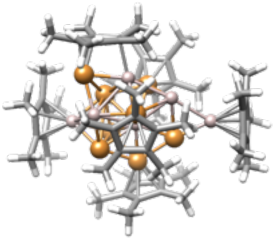

3.12 eV

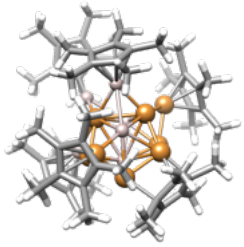

3.13 eV

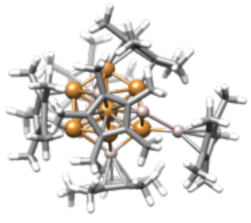

3.34 eV

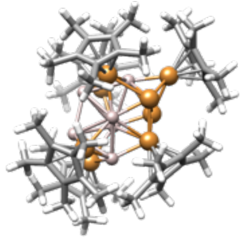

3.47 eV

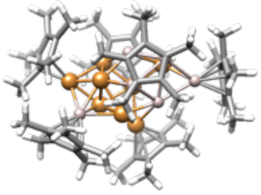

4.16 eV

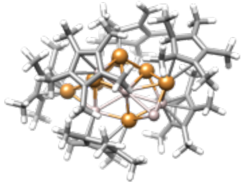

4.46 eV

2.7.10 $[Ni_7Ga_6](Cp^*)_6$ Complexes

Supplementary Table 38: Energetic and electronic properties for $[Ni_7Ga_6](Cp^*)_6$ complexes obtained with PBE+TS/light-tier1 level and light SCF parameters: configuration number (i), relative total energy (ΔE_{tot}), total magnetic moment (m_{tot}), HOMO energy (ε_H), LUMO energy (ε_L), and LUMO-HOMO energy gap (E_g).

i	ΔE_{tot} (meV)	m_{tot} (μ_B)	ε_H (eV)	ε_L (eV)	E_g (eV)
261	9370	2	−3.29	−2.94	0.35
274	8736	2	−3.30	−3.12	0.18
137	8585	0	−3.29	−2.99	0.30
6	8283	2	−3.16	−2.97	0.19
111	8232	2	−3.47	−3.28	0.19
73	8175	2	−3.11	−2.88	0.23
62	8053	4	−3.30	−3.19	0.10
89	7997	2	−3.32	−3.15	0.17
233	7916	2	−3.40	−3.24	0.16
112	7711	0	−3.13	−2.65	0.48
257	7626	0	−3.31	−2.89	0.42
167	7577	2	−3.08	−2.95	0.13
146	7320	2	−3.29	−2.94	0.35
158	7061	2	−3.25	−3.01	0.24
267	6954	2	−3.17	−3.04	0.14
292	6905	0	−3.20	−2.64	0.57
156	6893	2	−3.29	−3.16	0.14
109	6862	2	−3.32	−3.06	0.26
229	6834	0	−3.43	−3.19	0.24
303	6813	0	−3.16	−2.98	0.18
171	6714	2	−3.28	−3.01	0.27
214	6661	0	−3.40	−2.89	0.51

Continued on next page

Supplementary Table 38: Energetic and electronic properties for $[Ni_7Ga_6](Cp^*)_6$ complexes obtained with PBE+TS/light-tier1 level and light SCF parameters: configuration number (i), relative total energy (ΔE_{tot}), total magnetic moment (m_{tot}), HOMO energy (ε_H), LUMO energy (ε_L), and LUMO-HOMO energy gap (E_g).

i	ΔE_{tot} (meV)	m_{tot} (μ_B)	ε_H (eV)	ε_L (eV)	E_g (eV)
66	6655	2	−3.24	−3.10	0.13
59	6608	2	−3.30	−3.19	0.11
52	6593	2	−3.15	−2.99	0.16
308	6524	2	−3.15	−2.97	0.18
23	6524	0	−3.28	−3.02	0.26
8	6519	0	−3.27	−3.12	0.15
3	6500	2	−3.28	−3.04	0.24
29	6484	0	−3.02	−2.62	0.40
311	6471	2	−3.06	−2.90	0.16
118	6454	0	−3.34	−2.92	0.42
187	6431	2	−3.31	−3.09	0.22
144	6399	2	−3.24	−2.96	0.28
150	6359	0	−3.33	−2.97	0.37
24	6293	2	−3.29	−3.06	0.23
175	6292	2	−3.39	−3.10	0.29
163	6266	2	−3.17	−3.00	0.17
305	6266	0	−3.14	−2.86	0.28
280	6223	2	−3.32	−3.15	0.17
128	6205	2	−3.15	−2.95	0.19
192	6182	0	−3.21	−2.89	0.33
139	6170	0	−3.33	−2.68	0.66
25	6127	0	−3.21	−2.76	0.45

Continued on next page

Supplementary Table 38: Energetic and electronic properties for $[Ni_7Ga_6](Cp^*)_6$ complexes obtained with PBE+TS/light-tier1 level and light SCF parameters: configuration number (i), relative total energy (ΔE_{tot}), total magnetic moment (m_{tot}), HOMO energy (ε_H), LUMO energy (ε_L), and LUMO-HOMO energy gap (E_g).

i	ΔE_{tot} (meV)	m_{tot} (μ_B)	ε_H (eV)	ε_L (eV)	E_g (eV)
301	6123	0	−3.10	−2.80	0.30
272	6119	2	−3.11	−2.86	0.26
234	6112	2	−3.27	−3.02	0.25
209	6095	2	−3.26	−3.02	0.24
18	6086	2	−3.23	−3.12	0.11
242	6070	0	−3.40	−2.70	0.71
159	6043	2	−3.19	−2.93	0.27
86	6002	0	−3.39	−3.11	0.27
186	5995	2	−2.92	−2.68	0.24
231	5961	0	−3.15	−2.79	0.36
165	5917	2	−3.23	−3.09	0.14
297	5898	0	−3.42	−2.98	0.44
122	5860	2	−3.33	−2.96	0.37
199	5780	2	−3.31	−3.15	0.16
41	5776	2	−3.09	−2.84	0.25
17	5760	2	−3.10	−2.88	0.22
85	5750	2	−3.39	−3.18	0.21
100	5744	0	−3.20	−2.68	0.52
153	5713	2	−3.20	−3.01	0.19
135	5710	0	−3.19	−2.73	0.46
241	5704	2	−3.01	−2.86	0.15
185	5704	2	−3.18	−2.92	0.27

Continued on next page

Supplementary Table 38: Energetic and electronic properties for $[Ni_7Ga_6](Cp^*)_6$ complexes obtained with PBE+TS/light-tier1 level and light SCF parameters: configuration number (i), relative total energy (ΔE_{tot}), total magnetic moment (m_{tot}), HOMO energy (ε_H), LUMO energy (ε_L), and LUMO-HOMO energy gap (E_g).

i	ΔE_{tot} (meV)	m_{tot} (μ_B)	ε_H (eV)	ε_L (eV)	E_g (eV)
244	5677	2	−3.23	−3.08	0.14
281	5654	2	−3.27	−3.07	0.20
61	5645	0	−3.36	−3.09	0.26
284	5625	0	−3.30	−2.75	0.56
309	5624	2	−3.25	−2.98	0.27
93	5608	2	−3.30	−3.16	0.14
253	5594	0	−3.22	−2.55	0.66
288	5591	0	−3.40	−2.72	0.68
271	5575	2	−3.21	−3.00	0.21
239	5566	2	−3.18	−2.86	0.32
42	5550	2	−3.17	−2.84	0.33
101	5532	0	−3.16	−2.87	0.29
63	5444	0	−3.02	−2.66	0.36
240	5442	0	−3.37	−3.07	0.30
38	5399	2	−3.25	−2.99	0.26
249	5305	4	−3.22	−3.07	0.15
169	5289	0	−3.32	−2.84	0.49
53	5273	0	−3.14	−2.72	0.42
102	5264	0	−3.24	−2.99	0.25
237	5255	2	−3.13	−2.95	0.18
12	5253	2	−3.27	−3.08	0.19
252	5250	0	−3.12	−2.86	0.27

Continued on next page

Supplementary Table 38: Energetic and electronic properties for $[Ni_7Ga_6](Cp^*)_6$ complexes obtained with PBE+TS/light-tier1 level and light SCF parameters: configuration number (i), relative total energy (ΔE_{tot}), total magnetic moment (m_{tot}), HOMO energy (ε_H), LUMO energy (ε_L), and LUMO-HOMO energy gap (E_g).

i	ΔE_{tot} (meV)	m_{tot} (μ_B)	ε_H (eV)	ε_L (eV)	E_g (eV)
226	5236	2	−3.07	−2.79	0.28
121	5211	2	−3.36	−3.18	0.18
217	5194	2	−3.17	−2.95	0.23
149	5189	2	−3.18	−3.07	0.11
204	5185	2	−3.30	−3.09	0.21
299	5183	2	−3.31	−3.16	0.15
183	5157	2	−3.42	−3.13	0.28
83	5150	2	−3.20	−3.02	0.17
191	5131	0	−3.29	−3.05	0.24
58	5128	2	−3.26	−2.97	0.29
134	5124	0	−3.33	−2.86	0.48
221	5101	0	−3.09	−2.68	0.42
224	5083	2	−3.22	−3.00	0.22
104	5077	0	−3.09	−2.86	0.23
76	5066	2	−3.10	−2.99	0.11
14	5065	2	−3.29	−3.03	0.25
65	5053	2	−3.22	−3.07	0.15
263	5048	2	−3.21	−3.02	0.19
219	5005	2	−3.26	−3.02	0.23
256	4963	2	−3.38	−3.19	0.19
72	4949	2	−3.16	−2.85	0.31
225	4937	2	−3.14	−2.92	0.21

Continued on next page

Supplementary Table 38: Energetic and electronic properties for $[Ni_7Ga_6](Cp^*)_6$ complexes obtained with PBE+TS/light-tier1 level and light SCF parameters: configuration number (i), relative total energy (ΔE_{tot}), total magnetic moment (m_{tot}), HOMO energy (ε_H), LUMO energy (ε_L), and LUMO-HOMO energy gap (E_g).

i	ΔE_{tot} (meV)	m_{tot} (μ_B)	ε_H (eV)	ε_L (eV)	E_g (eV)
194	4928	2	−3.12	−2.75	0.36
120	4919	2	−3.21	−2.95	0.26
33	4908	2	−3.01	−2.82	0.19
46	4848	2	−3.09	−2.98	0.12
57	4846	2	−2.97	−2.78	0.19
49	4821	0	−2.99	−2.66	0.33
114	4813	2	−3.08	−2.88	0.20
181	4807	0	−3.18	−2.65	0.54
290	4797	2	−3.22	−3.02	0.21
283	4795	0	−3.26	−2.70	0.56
166	4766	2	−3.11	−2.97	0.14
55	4748	2	−3.16	−2.89	0.28
238	4711	0	−3.02	−2.82	0.20
35	4690	0	−3.28	−2.72	0.55
202	4672	0	−3.31	−2.85	0.46
230	4651	2	−3.34	−3.14	0.20
7	4638	2	−3.10	−2.89	0.22
140	4553	0	−3.12	−2.99	0.13
235	4527	2	−3.37	−3.24	0.14
92	4513	0	−3.11	−2.78	0.33
232	4513	2	−3.24	−3.06	0.18
132	4506	2	−3.34	−3.08	0.26

Continued on next page

Supplementary Table 38: Energetic and electronic properties for $[Ni_7Ga_6](Cp^*)_6$ complexes obtained with PBE+TS/light-tier1 level and light SCF parameters: configuration number (i), relative total energy (ΔE_{tot}), total magnetic moment (m_{tot}), HOMO energy (ε_H), LUMO energy (ε_L), and LUMO-HOMO energy gap (E_g).

i	ΔE_{tot} (meV)	m_{tot} (μ_B)	ε_H (eV)	ε_L (eV)	E_g (eV)
172	4502	2	−3.15	−3.01	0.14
320	4500	0	−3.35	−2.97	0.38
1	4481	0	−3.28	−2.67	0.61
126	4465	0	−3.14	−2.64	0.50
177	4458	2	−3.09	−2.95	0.14
259	4375	2	−2.99	−2.81	0.18
21	4348	0	−3.28	−3.05	0.23
84	4314	0	−3.27	−2.59	0.68
173	4276	0	−3.29	−3.11	0.18
131	4255	2	−3.12	−2.97	0.15
220	4203	2	−3.29	−3.02	0.27
164	4197	2	−3.01	−2.93	0.08
60	4187	2	−3.33	−3.09	0.23
312	4176	2	−2.98	−2.76	0.21
228	4133	2	−3.13	−2.84	0.29
74	4034	0	−3.17	−2.77	0.40
266	3940	0	−3.02	−2.68	0.34
176	3933	0	−3.35	−2.88	0.47
15	3931	0	−3.22	−2.91	0.31
179	3926	2	−3.11	−2.99	0.12
313	3921	2	−3.21	−2.96	0.25
248	3905	2	−3.11	−2.83	0.29

Continued on next page

Supplementary Table 38: Energetic and electronic properties for $[Ni_7Ga_6](Cp^*)_6$ complexes obtained with PBE+TS/light-tier1 level and light SCF parameters: configuration number (i), relative total energy (ΔE_{tot}), total magnetic moment (m_{tot}), HOMO energy (ε_H), LUMO energy (ε_L), and LUMO-HOMO energy gap (E_g).

i	ΔE_{tot} (meV)	m_{tot} (μ_B)	ε_H (eV)	ε_L (eV)	E_g (eV)
180	3852	2	−3.18	−3.04	0.14
141	3819	0	−3.28	−2.84	0.44
82	3778	2	−3.03	−2.95	0.09
282	3740	2	−3.13	−2.84	0.29
145	3596	0	−3.30	−2.72	0.58
254	3359	2	−2.98	−2.88	0.10
88	3079	0	−3.32	−2.69	0.63
316	2807	0	−3.22	−2.68	0.53
193	2727	0	−3.00	−2.70	0.30
315	2494	0	−3.34	−2.79	0.55
302	2394	0	−3.05	−2.82	0.23
307	2369	0	−3.13	−2.58	0.55
318	2196	2	−3.09	−2.79	0.30
306	2101	0	−3.11	−2.62	0.50
5	1173	0	−3.31	−2.68	0.63
304	0	0	−3.26	−2.62	0.64

Supplementary Table 39: Optimized and filtered representative structures of $[Ni_7Ga_6](Cp^*)_6$ complexes obtained with PBE+TS/light-tier1 level and light SCF parameters. The relative total energy (ΔE_{tot}) is depicted above each structure.

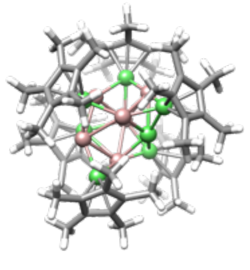

0 meV

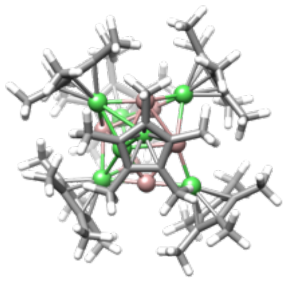

1.17 eV

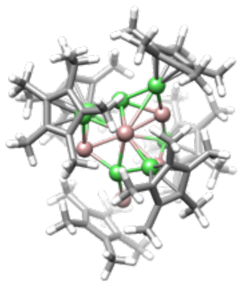

2.20 eV

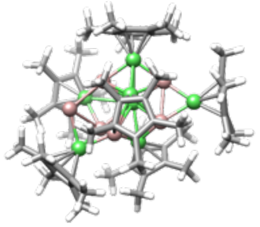

2.49 eV

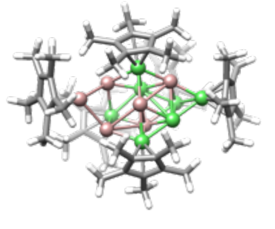

3.92 eV

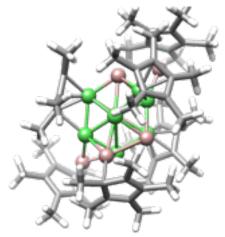

4.20 eV

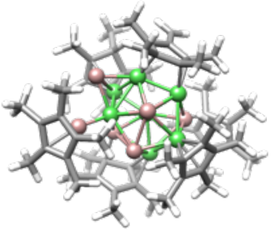

4.31 eV

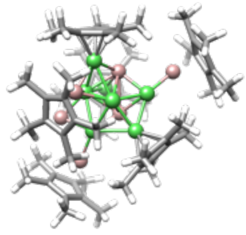

4.37 eV

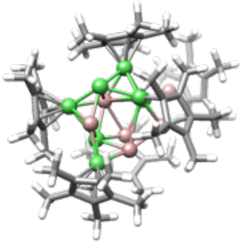

4.46 eV

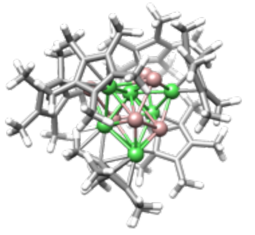

4.48 eV

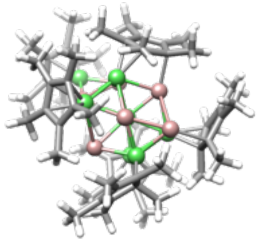

4.50 eV

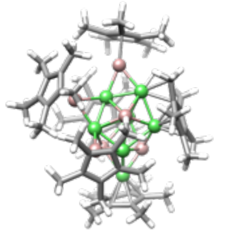

4.85 eV

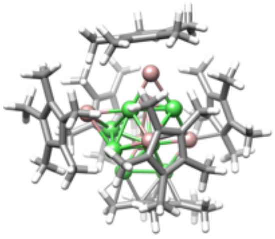

5.15 eV

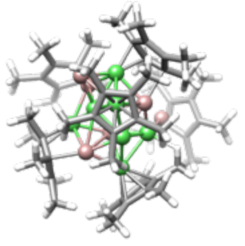

5.19 eV

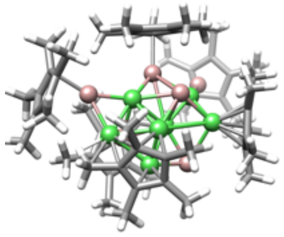

5.55 eV

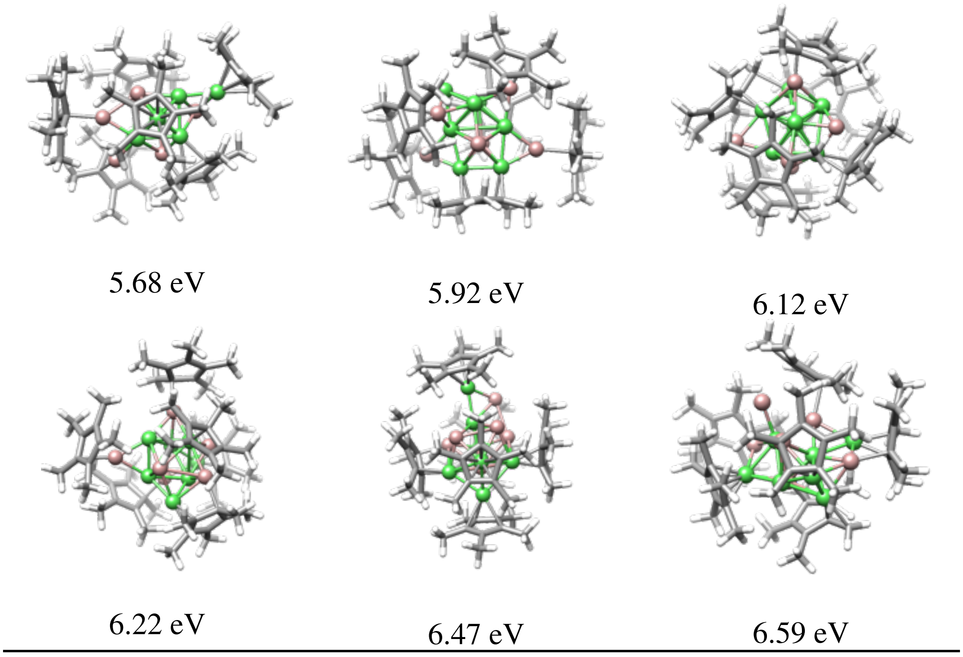

References

1 Rondina, G. G.; Da Silva, J. L. F. Revised Basin-Hopping Monte Carlo Algorithm for Structure Optimization of Clusters and Nanoparticles. *J. Chem. Inf. Model.* **2013**, *53*, 2282–2298, DOI: `10.1021/ci400224z`.

2 Cha, S.-H. Comprehensive Survey on Distance/Similarity Measures Between Probability Density Functions. *Int. J. Math. Models Methods Appl. Sci.* **2007**, *1*, 300–307.

3 Weßing, J.; Ganesamoorthy, C.; Kahlal, S.; Marchal, R.; Gemel, C.; Cador, O.; Da Silva, A. C. H.; Da Silva, J. L. F.; Saillard, J.-Y.; Fischer, R. A. The Mackay-type Cluster $[Cu_{43}Al_{12}](Cp^*)_{12}$: Open-shell 67-electron Superatom With Emerging Metal-like Electronic Structure. *Angew. Chem. Int. Ed.* **2018**, *57*, 14630–14634, DOI: `10.1002/anie.201806039`.

4 Morais, F. O.; Andriani, K. F.; Silva, J. L. F. D. Investigation of the Stability Mechanisms of Eight-Atom Binary Metal Clusters Using DFT Calculations and k-means Clustering Algorithm. **2021**, *61*, 3411–3420, DOI: `10.1021/acs.jcim.1c00253`.

5 de Mendonça, J. P. A.; Calderan, F. V.; Lourenço, T. C.; Quiles, M. G.; Da Silva, J.

L. F. Theoretical Framework Based on Molecular Dynamics and Data Mining Analyses for the Study of Potential Energy Surfaces of Finite-Size Particles. *Journal of Chemical Information and Modeling* **2022**, *62*, 5503–5512, DOI: 10.1021/acs.jcim.2c00957, PMID: 36302503.

6 Kresse, G.; Hafner, J. *Ab initio* Molecular Dynamics for Open-Shell Transition Metals. *Phys. Rev. B* **1993**, *48*, 13115–13118, DOI: 10.1103/physrevb.48.13115.

7 Kresse, G.; Furthmüller, J. Efficient Iterative Schemes For *Ab Initio* Total-Energy Calculations Using a Plane-Wave Basis set. *Phys. Rev. B* **1996**, *54*, 11169–11186, DOI: 10.1103/physrevb.54.11169.

8 te Velde, G.; Bickelhaupt, F. M.; Baerends, E. J.; Fonseca Guerra, C.; van Gisbergen, S. J. A.; Snijders, J. G.; Ziegler, T. Chemistry with ADF. *J. Comput. Chem.* **2001**, *22*, 931–967, DOI: 10.1002/jcc.1056.

9 Barca, G. M. J.; Bertoni, C.; Carrington, L.; Datta, D.; De Silva, N.; Deustua, J. E.; Fedorov, D. G.; Gour, J. R.; Gunina, A. O.; Guidez, E.; Harville, T.; Irle, S.; Ivanic, J.; Kowalski, K.; Leang, S. S.; Li, H.; Li, W.; Lutz, J. J.; Magoulas, I.; Mato, J.; Mironov, V.; Nakata, H.; Pham, B. Q.; Piecuch, P.; Poole, D.; Pruitt, S. R.; Rendell, A. P.; Roskop, L. B.; Ruedenberg, K.; Sattasathuchana, T.; Schmidt, M. W.; Shen, J.; Slipchenko, L.; Sosonkina, M.; Sundriyal, V.; Tiwari, A.; Galvez Vallejo, J. L.; Westheimer, B.; Wloch, M.; Xu, P.; Zahariev, F.; Gordon, M. S. Recent developments in the general atomic and molecular electronic structure system. *The Journal of Chemical Physics* **2020**, *152*, 154102, DOI: 10.1063/5.0005188.

10 Frisch, M. J.; Trucks, G. W.; Schlegel, H. B.; Scuseria, G. E.; Robb, M. A.; Cheeseman, J. R.; Scalmani, G.; Barone, V.; Petersson, G. A.; Nakatsuji, H.; Li, X.; Caricato, M.; Marenich, A. V.; Bloino, J.; Janesko, B. G.; Gomperts, R.; Mennucci, B.; Hratchian, H. P.; Ortiz, J. V.; Izmaylov, A. F.; Sonnenberg, J. L.; Williams-Young, D.; Ding, F.; Lipparini, F.; Egidi, F.; Goings, J.; Peng, B.; Petrone, A.; Henderson, T.; Ranasinghe, D.; Zakrzewski, V. G.; Gao, J.; Rega, N.; Zheng, G.; Liang, W.; Hada, M.; Ehara, M.; Toyota, K.;

Fukuda, R.; Hasegawa, J.; Ishida, M.; Nakajima, T.; Honda, Y.; Kitao, O.; Nakai, H.; Vreven, T.; Throssell, K.; Montgomery, J. A., Jr.; Peralta, J. E.; Ogliaro, F.; Bearpark, M. J.; Heyd, J. J.; Brothers, E. N.; Kudin, K. N.; Staroverov, V. N.; Keith, T. A.; Kobayashi, R.; Normand, J.; Raghavachari, K.; Rendell, A. P.; Burant, J. C.; Iyengar, S. S.; Tomasi, J.; Cossi, M.; Millam, J. M.; Klene, M.; Adamo, C.; Cammi, R.; Ochterski, J. W.; Martin, R. L.; Morokuma, K.; Farkas, O.; Foresman, J. B.; Fox, D. J. Gaussian~16 Revision C.01. 2016; Gaussian Inc. Wallingford CT.

11 Blum, V.; Gehrke, R.; Hanke, F.; Havu, P.; Havu, V.; Ren, X.; Reuter, K.; Scheffler, M. *Ab initio* Molecular Simulations With Numeric Atom-Centered Orbitals. *Comput. Phys. Commun.* **2009**, *180*, 2175–2196, DOI: 10.1016/j.cpc.2009.06.022.

12 Havu, V.; Blum, V.; Havu, P.; Scheffler, M. Efficient Integration for all-Electron Electronic Structure Calculation Using Numeric Basis Functions. *J. Comput. Phys.* **2009**, *228*, 8367–8379, DOI: 10.1016/j.jcp.2009.08.008.

13 Cai, W.; Feng, Y.; Shao, X.; Pan, Z. Optimization of Lennard-Jones atomic clusters. *Journal of Molecular Structure: THEOCHEM* **2002**, *579*, 229–234, DOI: https://doi.org/10.1016/S0166-1280(01)00730-8.

14 Jain, A. K.; Murty, M. N.; Flynn, P. J. Data Clustering: A Review. *ACM Comput. Surv.* **1999**, *31*, 264–323, DOI: 10.1145/331499.331504.

15 Rupp, M.; Tkatchenko, A.; Müller, K.-R.; von Lilienfeld, O. A. Fast and Accurate Modeling of Molecular Atomization Energies with Machine Learning. *Physiscal Review Letters* **2012**, *108*, 058301, DOI: 10.1103/PhysRevLett.108.058301.

16 Rousseeuw, P. J. Silhouettes: A graphical aid to the interpretation and validation of cluster analysis. *Journal of computational and applied mathematics* **1987**, *20*, 53–65.

17 Gonzalez, A. Measurement of Areas on a Sphere Using Fibonacci and Latitude-Longitude Lattices. *Mathematical Geosciences* **2010**, *42*, 49–64, DOI: 10.1007/s11004-009-9257-x.

18 Perdew, J. P.; Burke, K.; Ernzerhof, M. Generalized Gradient Approximation Made Simple. *Phys. Rev. Lett.* **1996**, *77*, 3865–3868, DOI: 10.1103/physrevlett.77.3865.

19 Tkatchenko, A.; Scheffler, M. Accurate Molecular Van Der Waals Interactions from Ground-State Electron Density and Free-Atom Reference Data. *Phys. Rev. Lett.* **2009**, *102*, 073005, DOI: 10.1103/physrevlett.102.073005.

20 Averill, F. W. An Efficient Numerical Multicenter Basis Set for Molecular Orbital Calculations: Application to $FeCl_4$. *J. Chem. Phys.* **1973**, *59*, 6412–6418, DOI: 10.1063/1.1680020.

21 van Lenthe, E.; Snijders, J. G.; Baerends, E. J. The Zero-Order Regular Approximation for Relativistic Effects: The Effect of Spin–Orbit Coupling in Closed Shell Molecules. *J. of Chem. Phys.* **1996**, *105*, 6505–6516, DOI: 10.1063/1.472460.

22 Chaves, A. S.; Piotrowski, M. J.; Da Silva, J. L. F. Evolution of the Structural, Energetic, and Electronic properties of the 3*d*, 4*d*, and 5*d* Transition-Metal Clusters (30 TM_n Systems for $n = 2 - 15$): A Density Functional Theory Investigation. *Phys. Chem. Chem. Phys.* **2017**, *19*, 15484–15502, DOI: 10.1039/c7cp02240a.
